# Genome-Based Taxonomic Classification of *Bacteroidetes*

**DOI:** 10.3389/fmicb.2016.02003

**Published:** 2016-12-20

**Authors:** Richard L. Hahnke, Jan P. Meier-Kolthoff, Marina García-López, Supratim Mukherjee, Marcel Huntemann, Natalia N. Ivanova, Tanja Woyke, Nikos C. Kyrpides, Hans-Peter Klenk, Markus Göker

**Affiliations:** ^1^Department of Microorganisms, Leibniz Institute DSMZ–German Collection of Microorganisms and Cell Cultures Braunschweig, Germany; ^2^Department of Energy Joint Genome Institute (DOE JGI) Walnut Creek, CA, USA; ^3^Department of Biological Sciences, Faculty of Science, King Abdulaziz University Jeddah, Saudi Arabia; ^4^School of Biology, Newcastle University Newcastle upon Tyne, UK

**Keywords:** G+C content, genome BLAST distance phylogeny, gliding motility, gut microbiome, marine microbiology, one thousand microbial genomes project, phylogenetic classification, *Bacteroidaeota-Rhodothermaeota-Balneolaeota-Chlorobaeota* superphylum

## Abstract

The bacterial phylum *Bacteroidetes*, characterized by a distinct gliding motility, occurs in a broad variety of ecosystems, habitats, life styles, and physiologies. Accordingly, taxonomic classification of the phylum, based on a limited number of features, proved difficult and controversial in the past, for example, when decisions were based on unresolved phylogenetic trees of the 16S rRNA gene sequence. Here we use a large collection of type-strain genomes from *Bacteroidetes* and closely related phyla for assessing their taxonomy based on the principles of phylogenetic classification and trees inferred from genome-scale data. No significant conflict between 16S rRNA gene and whole-genome phylogenetic analysis is found, whereas many but not all of the involved taxa are supported as monophyletic groups, particularly in the genome-scale trees. Phenotypic and phylogenomic features support the separation of *Balneolaceae* as new phylum *Balneolaeota* from *Rhodothermaeota* and of *Saprospiraceae* as new class *Saprospiria* from *Chitinophagia*. *Epilithonimonas* is nested within the older genus *Chryseobacterium* and without significant phenotypic differences; thus merging the two genera is proposed. Similarly, *Vitellibacter* is proposed to be included in *Aequorivita*. *Flexibacter* is confirmed as being heterogeneous and dissected, yielding six distinct genera. *Hallella seregens* is a later heterotypic synonym of *Prevotella dentalis*. Compared to values directly calculated from genome sequences, the G+C content mentioned in many species descriptions is too imprecise; moreover, corrected G+C content values have a significantly better fit to the phylogeny. Corresponding emendations of species descriptions are provided where necessary. Whereas most observed conflict with the current classification of *Bacteroidetes* is already visible in 16S rRNA gene trees, as expected whole-genome phylogenies are much better resolved.

## Introduction

*Bacteroidetes* comprise bacteria widespread in the biosphere and isolated from many distinct habitats, including temperate, tropical and polar ecosystems (Krieg et al., 2010; Thomas et al., 2011). *Bacteroides* are anaerobic and mostly found in the gastrointestinal tract of animals and humans and, besides *Firmicutes*, even dominates the gut microflora of mammals (Smith et al., 2006; Ley et al., 2009; Thomas et al., 2011). Several *Cytophagia* were cultured from marine habitats, whereas *Cytophaga* was found in soils and *Cyclobacteriaceae* in (hyper-)saline environments only (Krieg et al., 2010). *Flavobacteriaceae* have colonized many different ecosystems such as soils, sediment, freshwater, brackish water, and seawater in temperate, tropical, and polar ecosystems (Bernardet, 2011). Some *Flavobacteriaceae* are even pathogenic for humans, other mammals, freshwater fish or marine fish. However, *Blattabacteriaceae* are endosymbiotic bacteria (e.g., in termites and cockroaches), whereas *Cryomorphaceae* mostly live in cold, marine environments (Bowman et al., 2003). The wide variety of habitats reflects the importance of *Bacteroidetes* in biogeochemical processes. For instance, aquatic, terrestrial and gut *Bacteroidetes* are well known for their functional specialization on the decomposition of peptides and polysaccharides (Kirchman, 2002; Bowman, 2006; Thomas et al., 2011; Fernández-Gómez et al., 2013). This feature is accompanied by a great number and diversity of carbohydrate-active enzymes (Cantarel et al., 2009) in *Bacteroidetes* genomes (Fernández-Gómez et al., 2013). The corresponding genes cluster together with TonB-dependent transporters in polysaccharide-utilization loci (Martens et al., 2009; Sonnenburg et al., 2010; Fernández-Gómez et al., 2013). Genome sequences are expected to support investigating the evolutionary relationship of gut *Bacteroidetes* and the diet of their hosts, facilitated by lateral gene (carbohydrate-active enzymes) and gene cluster (polysaccharide utilization loci) transfer between environmental and gut *Bacteroidetes* (Thomas et al., 2011).

*Bacteroidetes* are Gram-stain-negative, chemo-organotrophic rods that do not form endospores and are either non-motile or motile by gliding (Woese, 1987; Paster et al., 1994). Before the phylum was called *Bacteroidetes* (Krieg et al., 2010), it had been referred to as *Cytophaga-Flavobacteria-Bacteroides* (Paster et al., 1994; Woese, 1987). The phylum comprises the classes *Bacteroidia, Cytophagia, Flavobacteriia*, and *Sphingobacteriia* (Krieg et al., 2010). Recently, the name *Bacteroidaeota* was proposed for this phylum by including the rank phylum in the International Code of Nomenclature of Prokaryotes (Oren et al., 2015). As *Cytophaga, Flexibacter*, and *Flavobacterium* have many phenotypic characteristics in common, their differentiation used to be based on the presence or absence of gliding motility (Bernardet et al., 1996). However, gliding motility is a common feature of many *Bacteroidetes* genera (McBride and Zhu, 2013). *Cytophaga* and *Flexibacter* were also delineated based on cell morphology, the G+C content as well as the habitats they were isolated from Reichenbach (1989c). The anaerobic *Bacteroidia* used to be considered separate from the aerobic groups such as *Flavobacteriia* and *Cytophagia*, but 16S rRNA gene sequencing clarified their interconnections (Paster et al., 1985; Weisburg et al., 1985; Woese, 1987). Despite its usefulness in resolving such taxonomic questions, the 16S rRNA gene contains only a limited number of characters and thus usually yields only partially resolved phylogenies, i.e., trees with many statistically unsupported branches (Klenk and Göker, 2010; Breider et al., 2014). Recently, Munoz et al. (2016) revised the phylogeny of *Bacteroidetes* and removed the *incertae sedis* taxa from the *Balneola* group and *Rhodothermaceae* (*Bacteroidetes* Order II. *incertae sedis*) from the *Bacteroidetes* as the novel phylum *Rhodothermaeota*. Nevertheless, many unsatisfactory aspects of *Bacteroidetes* classification might still persist.

Indeed, only monophyletic taxa can be accepted in a taxonomic classification because its purpose is to summarize the phylogeny of the classified organisms (Hennig, 1965; Wiley and Lieberman, 2011), and genome-scale data are more promising than single genes, or multi-locus sequence analysis restricted to a low number of genes, to identify monophyletic and non-monophyletic groups with high confidence (Klenk and Göker, 2010). The phenomenal increase in the number of publicly available whole-genome sequences further demands a genome-based classification system in today's genomic era.

The genomic G+C content, i.e., the proportion of cytosines and guanines among all nucleotides in the genome, is one of the most frequently used taxonomic markers in microbiology (Mesbah et al., 1989; Rosselló-Mora and Amann, 2001). Within *Bacteroidetes*, it is strongly recommended to include the G+C content especially when describing every species of *Flavobacteriaceae* (Bernardet et al., 2002). The rapid progress in sequencing technology (Liu et al., 2012; Mavromatis et al., 2012) allows not only for inferring genome-scale phylogenies (Klenk and Göker, 2010; Meier-Kolthoff et al., 2014a) but also for replacing traditional methods that indirectly determine the G+C content (Mesbah et al., 1989; Moreira et al., 2011) by calculating it directly from highly accurate genome sequences. For this reason, literature claims that the variation of the G+C content within bacterial species is at most 3 mol% (Mesbah et al., 1989) or even up to 5 mol% (Rosselló-Mora and Amann, 2001) can be attributed to the imprecision of traditional methods (Meier-Kolthoff et al., 2014c). On the other hand, within-species variation is at most 1% when both species boundaries (Meier-Kolthoff et al., 2013a) and G+C contents are determined from genome sequences (Meier-Kolthoff et al., 2014c). These inconsistencies call for correcting species descriptions that include a conventionally determined G+C content value that differs by more than 1% from the value calculated from the genome sequence of the type strain (Meier-Kolthoff et al., 2014c; Riedel et al., 2014). Apparently the same holds if a range of G+C values was provided whose lower or upper bound deviates by more than 1% from the directly calculated G+C value, but a species description should also be restricted if a range of G+C values was provided that, unrealistically, exceeds 1%. Taxa of higher rank should accordingly be emended if their presumed range of G+C values turns out to be in conflict with the information from genome sequences (Scheuner et al., 2014).

The Genomic Encyclopedia of *Bacteria* and *Archaea* (GEBA) pilot phase as well as the One Thousand Microbial Genomes phase 1 (KMG-1) projects (Kyrpides et al., 2014) are ideal data sources for genome-scale taxonomic reasoning because these projects aimed at filling the genomic gaps in the bacterial and archaeal branches of the tree of life (Göker and Klenk, 2013) and included only type strains with a certified origin from a culture collection and thus a verifiable history. Using the *Bacteroidetes* genome sequences from these projects, we here address the following questions: (i) What is the relationship between the phylogenomic trees and the proposed taxonomic classifications or the 16S rRNA gene phylogenies? (ii) Which taxa need to be revised because they are evidently non-monophyletic? (iii) Which taxon descriptions that lack G+C values should be augmented with information from genome sequences? (iv) Which taxon descriptions deviate from G+C content values calculated from genome sequences and should now be emended accordingly? (v) Does the correction of G+C values improve their fit to the phylogeny?

## Materials and methods

The *Bacteroidetes* (ingroup), *Chlorobi, Planctomycetes*, and *Verrucomicrobia* (outgroup) type-strain genomes originating from the GEBA pilot phase and the KMG-1 (Kyrpides et al., 2014) project were downloaded from IMG (Markowitz et al., 2009) and augmented with additional type-strain genomes of taxonomic interest deposited in INSDC. The complete list is found in Supplementary Table 1. Genome-scale phylogenies were inferred from whole proteomes using the high-throughput version (Meier-Kolthoff et al., 2014a) of the Genome BLAST Distance Phylogeny (GBDP) approach (Henz et al., 2005; Auch et al., 2006). BLAST+ (v2.2.30) (Camacho et al., 2009) was run in BLASTP mode with default parameters except for an *e*-value filter of 10^−8^ (Meier-Kolthoff et al., 2014a). The greedy-with-trimming algorithm, which conducts a correction for non-orthologous hits, was applied in conjunction with formula *d*_5_, which relates the (weighted) number of identities within BLAST hits (high-scoring segment pairs) to the overall length of these hits and thus is unaffected by incomplete genome sequencing, and subjected to 100 pseudo-bootstrap replicates (Meier-Kolthoff et al., 2013a, 2014a). Phylogenetic trees were inferred from the original and pseudo-bootstrapped intergenomic distance matrices using FastME (Lefort et al., 2015), and tree and support values visualized using ITOL (Letunic and Bork, 2011). Where species affiliations had to be clarified, digital DNA:DNA hybridization was conducted with the recommended settings of the Genome-To-Genome Distance Calculator (GGDC) version 2.1 (Meier-Kolthoff et al., 2013a).

Comprehensive, aligned, near full-length 16S rRNA gene data for *Bacteroidetes* and the outgroup phyla were taken from version s123 of the All-Species Living Tree Project (LTP) (Yarza et al., 2008). Sequences of species of interest missing from LTP s123 (see Supplementary File 2) were added to the alignment using POA (Lee et al., 2002). Trees were inferred from the alignment with RAxML (Stamatakis, 2014) under the maximum-likelihood (ML) criterion in conjunction with the GTR-CAT model, fast bootstrapping, bootstopping (Pattengale et al., 2010) and subsequent search for the best tree, and with TNT (Goloboff et al., 2008) under the maximum-parsimony criterion (MP); here, new-technology search for the best tree was conducted as well as 1000 bootstrapping replicates in conjunction with tree-bisection-and-reconnection branch swapping and one random sequence addition replicate per bootstrap replicate. Further ML and MP trees were inferred in the same way but using the branches of the GBDP tree with ≥95% support as backbone constraint. Finally, 16S rRNA gene trees reduced to genome-sequenced strains were inferred.

All trees were compared to the current classification used in LTP version s123, which was cleaned from inconsistencies, such as mismatches between species and genus names, and subsequently modified manually in the case of taxonomic arrangements that could not be confirmed in the literature, of validly published synonyms that were in better agreement to the phylogenies, and of missing taxa such as the family for *Saccharicrinis* (Yang et al., 2014). The full list of changes is described in Supplementary File 2. Whether taxa were monophyletic, paraphyletic or polyphyletic (Farris, 1974; Wood, 1994) was determined using program code developed at DSMZ, which reports, in the case of taxa appearing non-monophyletic in a phylogeny, the highest support of all branches that conflict with the monophyly of that taxon as support against it (that can be displayed as negative support). In a rooted tree, these conflicting branches are the ones connected to subtrees that contain some but not all representatives of the taxon, as well as representatives of other taxa.

The (trivial) calculation of the G+C content from genome sequences was done as in a previous study (Meier-Kolthoff et al., 2014c) and by the GGDC server version 2.1. The changes in the fit between phylogeny and G+C content data when switching from conventionally calculated values to G+C counts from genome sequences was assessed by calculating their MP score with TNT (Goloboff et al., 2008), which allows for treating continuous characters as such (Goloboff et al., 2006). The data were rescaled to fit in the range between 0 and 65 as necessary for TNT and exported using the opm package (Vaas et al., 2013) for R (R Development Core Team, 2015); species without a literature G+C value were deactivated. To address phylogenetic uncertainty, the difference was re-calculated for each pseudo-bootstrap tree, and the resulting set of differences tested for its difference from zero using a *t*-test and Wilcoxon signed rank test as implemented in R. Because some G+C contents are given as ranges in the literature, we alternatively assessed minima, averages and maxima of the ranges, combined with the single G+C values from the other sources. For each species, only the most recent emendation that included a G+C content value was considered. Visualization was done with ggplot2 (Wickham, 2009). We further investigated the effect of incomplete genome sequencing on the calculation of G+C content values using the simulation techniques from our earlier study (Auch et al., 2010); details are provided in Supplementary File 3.

## Results

### G+C content and phylogeny

**Figure 1:**
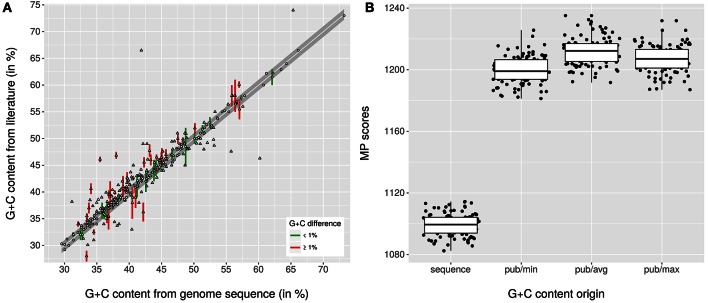
**Relationship between G+C content values from species descriptions and those calculated from the genome sequences**. **(A)** scatter plot showing the relationship between G+C content values calculated from the genome sequences and those found in the respective last revision in the literature, mostly calculated traditionally. The gray band indicates the boundary of 1% deviation, green color (when ranges were provided) and circles indicate values within that range, red color and triangles indicate values outside that range. **(B)** box plots showing the MP scores calculated with TNT from the rescaled G+C content values of distinct origin (pub, published; min, avg and max refer to whether the lower, average or upper value was used when a range was given) in conjunction with the 100 pseudo-bootstrapped GBDP trees. The values calculated from the genome sequences yield a significantly lower MP score (and thus a significantly better fit to the tree) than the ones from the respective last emendation in the literature.

Figure 1A shows the relationship between conventionally determined G+C content values and those calculated from the genome sequences; Supplementary Table 1 contains all collected data. A total of 161 species descriptions could be confirmed regarding the G+C content, for 45 it was obvious that they should be restricted because the provided G+C content range was unrealistically broad, 119 deviated by more than 1% from the genome sequence, and 24 lacked a G+C content range or value altogether. The MP scores of the conventionally determined and genome-sequence based G+C contents are shown in the Figure 1B. As confirmed by all tests conducted (α <0.001), it was obvious that the corrected G+C content values display a significantly better fit to the phylogeny than the original ones, and that this progress can be detected even though a large number of the G+C content values needed no correction. The results are in agreement with the simulations to assess the affect of incomplete genome sequencing on the calculation of the G+C content, which showed that the expected deviation from the real value is significantly below 0.1% for the given sequencing quality (Supplementary File 3).

**Figure 2:**
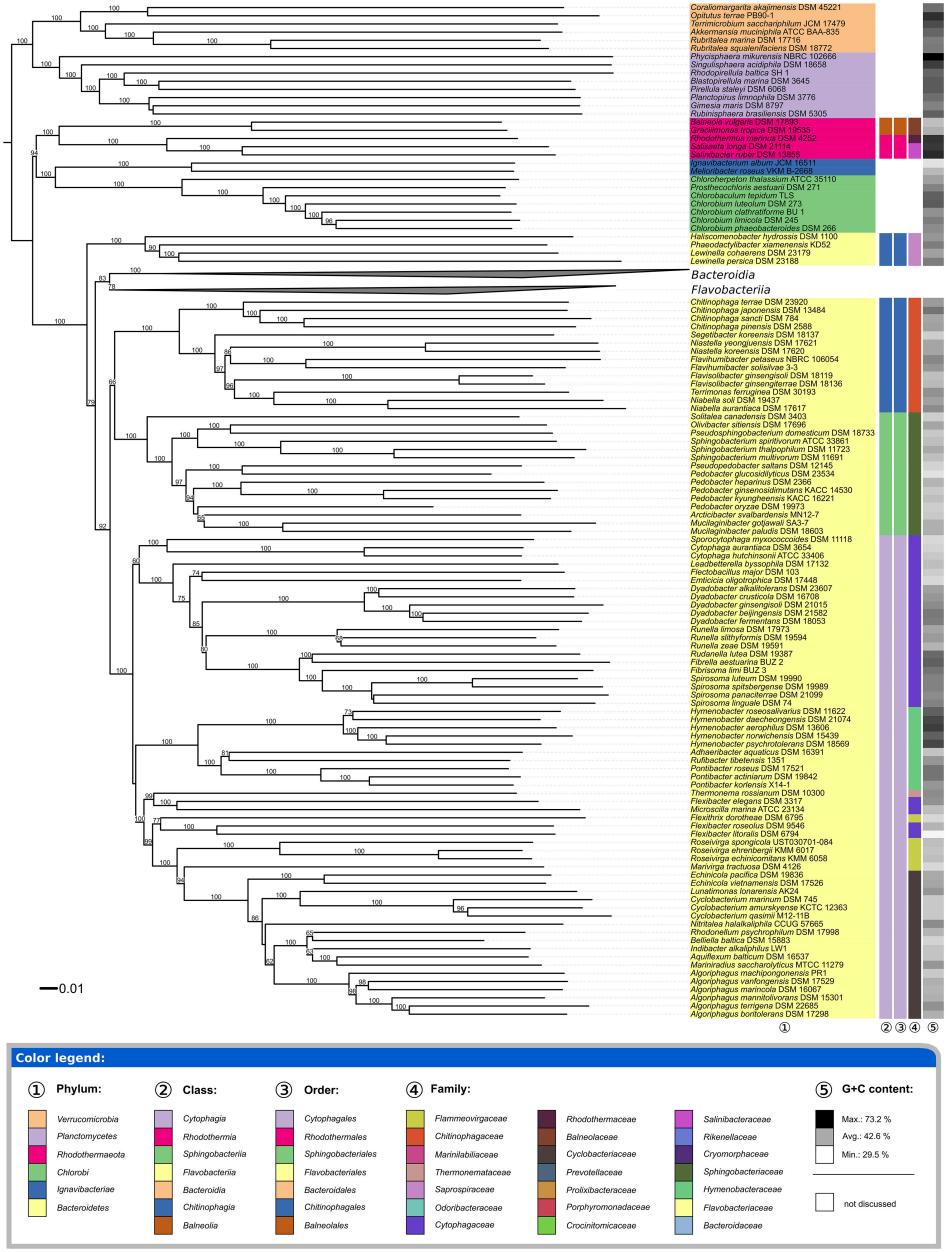
**First part of the phylogenomic tree inferred with GBDP**. Tree inferred with FastME from GBDP distances calculated from whole proteomes. The numbers above branches are GBDP pseudo-bootstrap support values from 100 replications. Tip colors indicate the phylum, colors to the right of the ingroup tips indicate, from left to right, class, order and family (see the embedded legend for details). Gray scale on the very right indicates the exact G+C content as calculated from the genome sequences. The *Bacteroidia* and *Flavobacteriia* parts of the tree, which have been collapsed here, are shown in Figures 3, 4.

**Figure 3:**
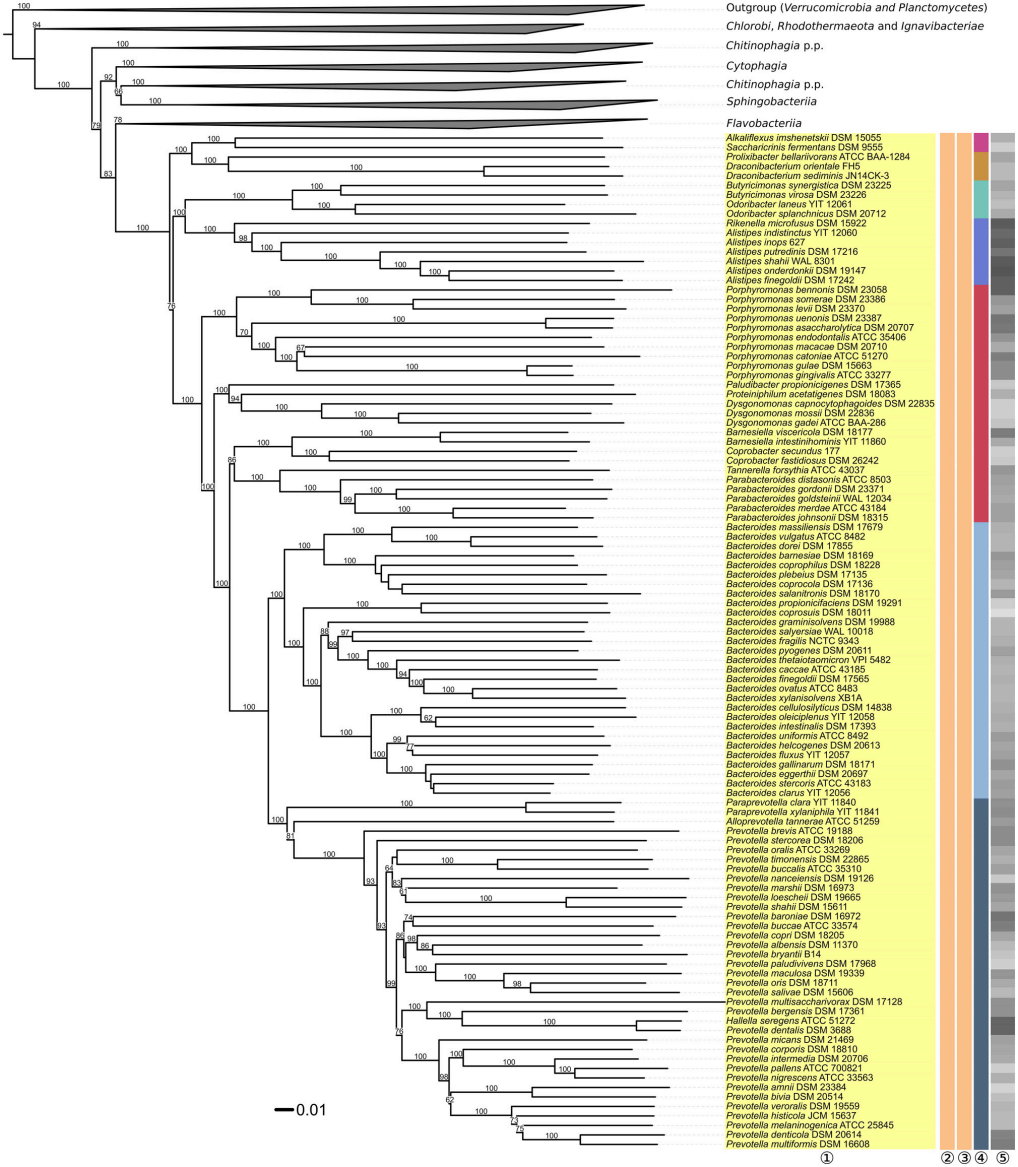
**Second part of the phylogenomic tree inferred with GBDP**. Tree inferred with FastME from GBDP distances calculated from whole proteomes. The numbers above branches are GBDP pseudo-bootstrap support values from 100 replications. Tip colors indicate the phylum, colors to the right of the ingroup tips indicate, from left to right, class, order and family (see the legend embedded in Figure 2 for details). Gray scale on the very right indicates the exact G+C content as calculated from the genome sequences. The non-*Bacteroidia* parts of the tree, which have been collapsed here, are shown in Figures 2, 4.

**Figure 4:**
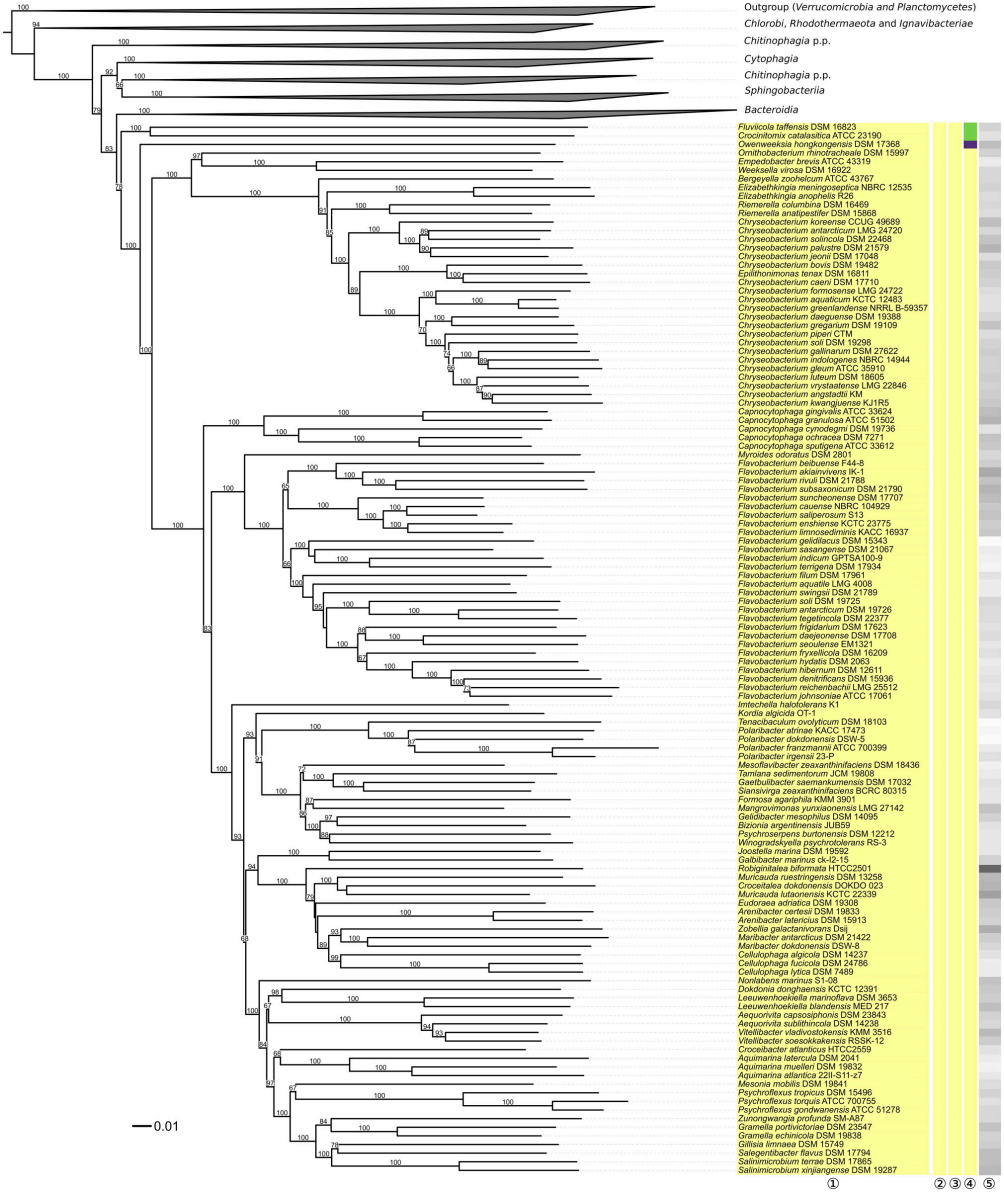
**Third part of the phylogenomic tree inferred with GBDP**. Tree inferred with FastME from GBDP distances calculated from whole proteomes. The numbers above branches are GBDP pseudo-bootstrap support values from 100 replications. Tip colors indicate the phylum, colors to the right of the ingroup tips indicate, from left to right, class, order and family (see the legend embedded in Figure 2 for details). Gray scale on the very right indicates the exact G+C content as calculated from the genome sequences. The non-*Flavobacteriia* parts of the tree, which have been collapsed here, are shown in Figures 2, 3.

**Figure 5:**
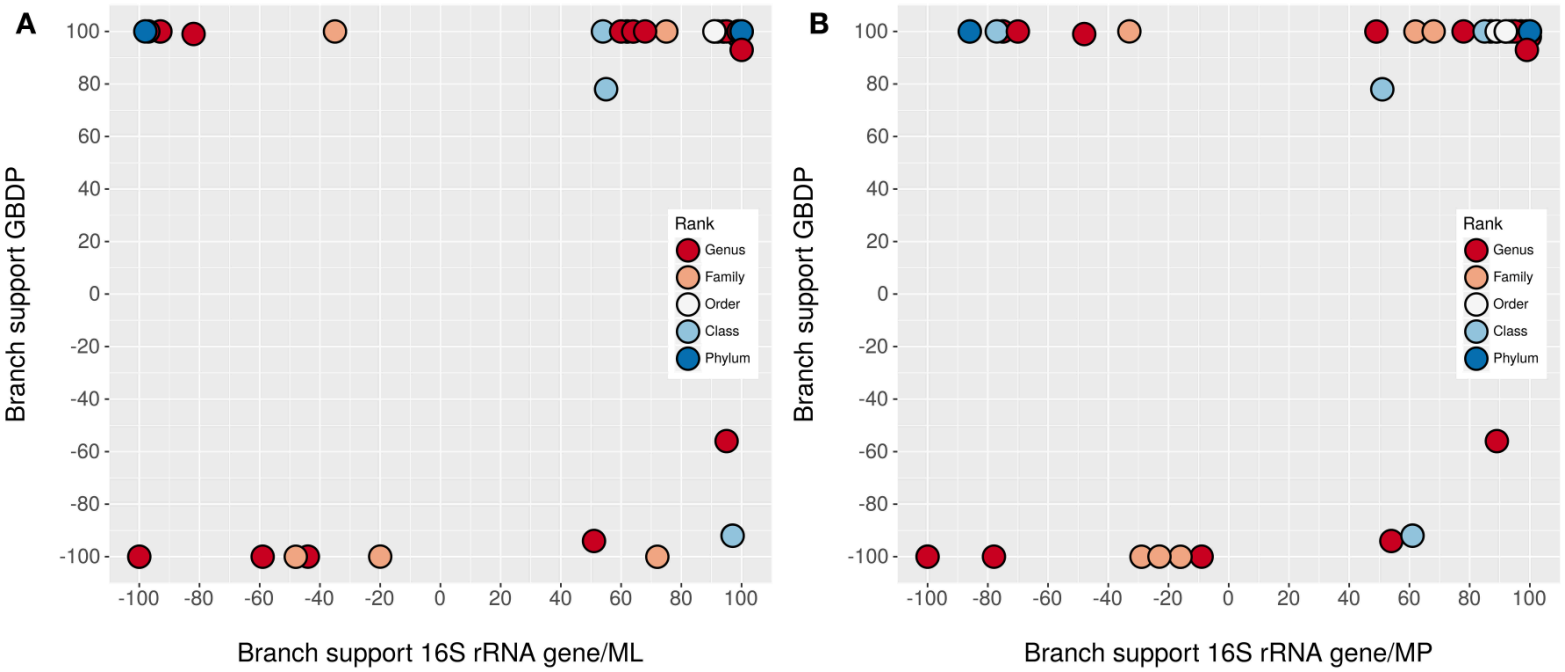
**Comparison of branch support**. Shown are the negative or positive branch support values for each taxon inferred from whole genomes with GBDP in comparison to those inferred from 16S rRNA gene sequences with ML **(A)** and MP **(B)**. The colors indicate the taxonomic rank. The upper right corner contains the taxa significantly supported by all methods, the lower left corner those significantly opposed by all methods. The other two corners, which would indicate a significant conflict between the methods, are empty. Jitter was used to avoid overplotting.

The underlying GDBP tree is shown in Figures 2–4 together with branch support values, taxonomic annotations and genomic G+C content indicators. Figure 5 compares the positive or negative support values for each taxon implicit in the GBDP (average support over all branches, 92.00%), 16S rRNA gene ML (69.47%), and 16S rRNA gene MP (67.25%) trees. The whole-genome tree supports more taxa than the 16S rRNA gene but also yields conflict in few cases where the 16S rRNA gene phylogeny is inconclusive, whereas several taxa are strongly supported as non-monophyletic by all approaches. However, in contrast to the classification, no well-supported (Taylor and Piel, 2004) discrepancies between the GBDP tree and the 16S rRNA gene trees were detected, as evident from the empty upper left and lower right corners in Figure 5. The unconstrained comprehensive 16S rRNA gene ML and MP trees (UCT) as well as the backbone-constrained ones (CCT) are included in Supplementary File 4. Whereas the majority of taxa appeared monophyletic in our analyses, a couple of discrepancies between the phylogenomic tree and the classification were observed, which we report herein in decreasing order of taxonomic rank involved, along with according suggestions for reclassifications.

### Phyla, classes, and orders

All phyla appeared as monophyletic in the GBDP tree (Figure 2). Given their separation from *Bacteroidetes*, the *Balneolia* and *Rhodothermia* could be assigned to a single phylum, *Rhodothermaeota* (Munoz et al., 2016). However, the 16S rRNA gene alone provides no support for their sister-group relationship (albeit no significant conflict), and the two groups strongly differ in their genomic G+C content (Figure 2), as previously noted (Urios et al., 2006); none of the remaining phyla show a comparable discrepancy in G+C values. Exactly the same reasons argue against an inclusion of the two groups in the phylum *Chlorobi* (Figure 2). Moreover, there are considerable phenotypic and habitat differences between the *Balneolia* and *Rhodothermia* (Table 1). The *Balneolia* are mesophilic, whereas *Rhodothermia* are mostly either strongly thermophilic (Alfredsson et al., 1988) or extremely halophilic (Antón et al., 2002). Carotenoids were only reported from *Rhodothermia* (Makhdoumi-Kakhki et al., 2012) but not from the *Balneolia*. For these reasons, the *Balneolia* and *Rhodothermia* should not only be removed from *Bacteroidetes* but also classified in two separate phyla, *Rhodothermaeota* (Munoz et al., 2016) and *Balneolaeota* (this study), based on the priority of the respective genus names. A comparable situation has been solved recently for the *Ignavibacteriaceae* (Iino et al., 2010) and *Melioribacteraceae* (Podosokorskaya et al., 2013). These taxa have been separated from the *Chlorobi* as a new phylum *Ignavibacteria* (Podosokorskaya et al., 2013) based on their physiological distinction and their large phylogenetic distance to *Chlorobi*.

**Table 1:** **Relevant phenotypic and genomic features of the *Balneola* clade and remaining *Rhodothermaeota***.

**Species**	**G+C content**	**Motility**	**Pigments**	**Colony color**	**Growth temperature**	**NaCl (w/v)**	**Major polar lipids**	**Polar lipid types**	**Major fatty acids**
*Aliifodinibius roseus*	49	None	?	Rose red	28 (42)	6–10 (4–20)	DPG, PC, PE, PL, 3L, GL	PL, AL, GL, L	iso-C_15:0_, anteiso-C_15:0_, summed feature 3 (C_16:1_ ω6c and/or C_16:1_ω7c), summed feature (iso-C_17:1_ ω9c and/or C_16:0_ 10-methyl)
*Aliifodinibius sediminis*	48.4	None	?	Salmon pink	28 (45)	6-10 (4-16)	DPG, PC, PE, PL, 3L, GL	PL, AL, GL, L	iso-C_15:0_, anteiso-C_15:0_, summed feature 3 (C_16:1_ ω6c and/or C_16:1_ ω7c), summed feature (iso-C_17:1_ ω9c and/or C_16:0_ 10-methyl)
*Balneola alkaliphila*	39	None	No flexirubin	Pale yellow; pale orange	25 (37)	3 (1–8)	DPG, PE, PG, GL, AL, L	PL, AL, GL, L	iso-C_13:0_, iso-C_15:0_, summed feature 3 (C_16:1_ ω6c and/or C_16:1_ ω7c)
*Balneola vulgaris*	42	Flagella	No flexirubin	Orange	30 (40)	2 (0–5)	DPG, PE, PG, GL, L	PL, AL, GL, L	iso-C_13:0_, iso-C_14:0_, iso-C_15:0_, summed feature (C_16:1_ ω6c and/or C_16:1_ ω7c)
*Fodinibius salinus*	43	None	?	Pink	37 (45)	10–15 (4–23)	DPG, PC, PE, PL, GL, AL	PL, AL, GL	iso-C_15:0_, C_16:1_ ω6c/C_16:1_ ω7c, summed feature (iso-C_17:1_ ω9c and/or C_16:0_ 10-methyl)
*Gracilimonas mengyeensis*	47.2	None	?	Red	28 (37)	5–8 (2–15)	DPG, PC, 2PL, GL	PL, AL, GL	iso-C_15:0_, anteiso-C_15:0_, summed feature (iso-C_17:1_ ω9c and/or C_16:0_ 10-methyl)
*Gracilimonas rosea*	43.2	None	No flexirubin	Pink	35 (40)	3.5–5 (1–20)	DPG, PE, PG, PL, 2GL, 2L	PL, AL, GL, L	iso-C_15:0_, iso-C_17:1_ ω9c, summed feature 3 (C_16:1_ ω7c and/or iso-C_15:0_ 2-OH)
*Gracilimonas tropica*	42.7	None	No flexirubin	Orange	35 (40)	3–6 (1–20)	DPG, PE, PG, PL, GL, 2L	PL, AL, GL, L	iso-C_15:0_, iso-C_17:1_ ω9c, summed feature 3 (C_16:1_ ω7c and/or iso-C_15:0_ 2-OH), anteiso-C_15:0_
*Rhodothermus marinus*	65	None	Carotenoids	Reddish	65 (77)	2 (1–6)	DPG, PE	PL, AL, GL	iso-C_16:0_, iso-C_17:0_, anteiso-C_15:0_, anteiso-C_17:0_
*Rhodothermus profundi*	60.9	None	No carotenoids	Cream	70 (80)	2–3 (1–5)	DPG, PE	PL, AL, GL	iso-C_16:0_, iso-C_17:0_, anteiso-C_15:0_, anteiso-C_17:0_
*Rubricoccus marinus*	68.9	None	?	Reddish	20–30 (37)	2 (1–5)	DPG, PE, PG, 2L	PL, AL, L	iso-C_17:1_ ω9c, C_17:1_ ω8c, iso-_C17:0_, C_16:0_
*Rubrivirga marina*	64.8-65.8	None	?	Pale-red	25–30 (37)	? (1–5)	DPG, PE, PG, PL, 2L	PL, AL, L	iso-C_15:0_, iso-C_17:0_, summed feature 3 (C_16:1_ ω6c and/or C_16:1_ ω7c), summed feature 4 (iso-C_17:1_ ω9c and/or C_16:0_ 10-methyl)
*Salinibacter iranicus*	64.8	None	Carotenoids	Red	37 (50)	18 (12–30)	DPG, PC, 3L, AL, 3GL	PL, AL, GL, L	iso-C_15:0_, C_18:1_ ω7c, summed feature 3 (C_16:1_ ω7c and/or iso-C_15:0_ 2-OH)
*Salinibacter luteus*	65.6	None	Carotenoids	Orange	37 (50)	18 (12–30)	DPG, PC, 3L, AL, 3GL	PL, AL, GL, L	iso-C_15:0_, C_18:1_ ω7c, summed feature 3 (C_16:1_ ω7c and/or iso-C_15:0_ 2-OH)
*Salinibacter ruber*	66.5	Flagella	Carotenoids	Red	37–47 (52)	23 (15–30)	DPG, PC, PE, 3GL, SL (halocapnine)	PL, AL, GL, SL	iso-C_15:0_, C_18:1_ ω7c, summed feature 3 (C_16:1_ ω7c and/or iso-C_15:0_ 2-OH)
*Salisaeta longa*	62.9	None	Carotenoids	Red	37–46 (50)	10–12 (5–20)	DPG, PC, PE, GL, 2SL (halocapnine)	PL, AL, GL, SL	iso-C_15:0_, summed feature 3 (C_16:1_ ω6c and/or C_16:1_ ω7c)

*All data are from the original descriptions (Alfredsson et al., 1988; Antón et al., 2002; Choi et al., 2009; Vaisman and Oren, 2009; Marteinsson et al., 2010; Park et al., 2011, 2013; Urios et al., 2006, 2008; Makhdoumi-Kakhki et al., 2012; Wang et al., 2012, 2013a,b; Cho et al., 2013) except for the polar lipids of Salinibacter ruber (Corcelli et al., 2004) and Salisaeta longa (Baronio et al., 2010). Question marks indicate missing information. Values in parentheses are maxima; those before parentheses are the optimal values or ranges. The abbreviations of the polar lipids are: AL, unidentified aminolipid; GL, unidentified glycolipid; L, unidentified polar lipid; PL, unidentified phospholipid; SL, unidentified sulfonolipid*.

The *Balneolaeota* (Munoz et al., 2016) and *Rhodothermaeota* (as suggested here) differ from *Bacteroidetes* by a considerable number of phenotypic characters. The large amount of phospholipids [diphosphatidylglycerol (DPG), phosphatidylethanolamine (PE), phosphatidylglycerol (PG) and phosphatidylcholine (PC)] found in *Rhodothermus* (and its relatives) is unusual for aerobic *Bacteroidetes* (Nolan et al., 2009). Polar lipids of *Bacteroides* comprise largely PE and small amounts of PG and phosphatidylserine (Wardle et al., 1996), whereas polar lipids of the phylum *Bacteroidetes* usually comprise significant amounts of amide-linked lipids (aminolipids) rather than ester-linked polar lipids (Bernardet, 2010). The most significant difference between the *Balneolaeota* and *Rhodothermaeota* on the one hand and *Bacteroidetes* on the other hand can be attributed to the composition of fatty acids. Characteristic fatty acids of *Bacteroidetes* are 2-hydroxy and 3-hydroxy fatty acids, predominantly iso-C_17:0_ 3-OH, iso-C_15:0_ 3-OH and iso-C_16:0_ 3-OH (Mayberry, 1980; Krieg et al., 2010), whereas strains of the *Balneolaeota* and *Rhodothermaeota* do not possess 2-hydroxy and 3-hydroxy fatty acids (Table 1). Flagella in conjunction with motility were observed in *Balneola vulgaris* (Urios et al., 2006) and *Salinibacter ruber* (Antón et al., 2002) but are unusual in *Bacteroidetes*; conversely, neither gliding motility nor flexirubin, a pigment common in *Bacteroidetes* (Krieg et al., 2010), were reported for the *Balneolaeota* and *Rhodothermaeota*. We suggest according emendations of taxon descriptions.

With the *Balneolaeota* and *Rhodothermaeota* removed from *Bacteroidetes*, the classes and orders of *Bacteroidetes* appeared as monophyletic in the GBDP phylogeny (Figure 2) with the sole exception of *Chitinophagia* and *Chitinophagales*, which were shown as paraphyletic due to the early branching of *Haliscomenobacter, Phaeodactylibacter*, and *Lewinella* (*Saprospiraceae*). Three branches with weak to moderate (79, 92, and 66%) support, respectively, would need to be wrong to obtain monophyletic *Chitinophagia* and *Chitinophagales*. The UCT and CCT show a distinct picture with a monophyletic *Chitinophagia and Chitinophagales* with 94–99% support under ML and <60–79% support under MP. Thus, regarding the monophyly criterion, it might or might not be adequate to place *Saprospiraceae* in *Chitinophagales* according to our analyses. The monophyly of the family itself, including the type genus *Saprospira*, is supported by the 16S rRNA gene trees with 95–100%. Thus, given its uncertain position relative to the remaining *Chitinophagia* and *Chitinophagales*, the taxonomic placement of *Saprospiraceae* should be reconsidered.

The family *Saprospiraceae* has been proposed first in Bergey's manual (Garrity and Holt, 2001) and was suggested to represent a sister lineage, without confirmatory evidence, to the family *Sphingobacteriaceae*. *Saprospiraceae* was later on placed in *Chitinophagales* but without relevant branch support (Munoz et al., 2016). In contrast to both *Sphingobacteriaceae* and *Chitinophagaceae, Saprospiraceae* are long rods (up to 5 μm) that form long filaments (up to 500 μm) and do not possess sphingophospholipids. Moreover, the 16S rRNA gene trees indicate that the group comprising both *Saprospiraceae* and *Chitinophagaceae* (which is not even monophyletic in Figure 2) is quite divergent. Based on these results, we propose to classify the family *Saprospiraceae* into the new order *Saprospirales*, of the new class *Saprospiria*. Moreover, some noticeable phenotypical characteristics in agreement with the branching order (Figure 2, Supplementary File 4) call for a split of the family *Saprospiraceae* into three families (*Saprospiraceae, Lewinellaceae*, and *Haliscomenobacteraceae*). *Lewinella, Saprospira* and *Aureispira* exhibit gliding motility, but only *Saprospira* and *Aureispira* form helical filaments (Krieg et al., 2012). In contrast, *Haliscomenobacter, Phaeodactylibacter*, and *Portibacter* are non-motile straight filaments.

A noticeable feature of the current classification of *Bacteroidetes* is that each class contains only a single order and thus the classification into orders provides no additional information. As this is not an issue of non-monophyly, we suggest addressing it once an even more comprehensive genome-scale phylogeny might help making more sense from these Linnaean ranks.

### Families

The recently revised (Munoz et al., 2016) *Rhodothermales* families appeared as monophyletic (Figure 2) but turned out to differ significantly by some key physiological characteristics. *Rhodothermus* (*Rhodothermaceae*) tolerates up to 6% NaCl and 70°C (Table 1). In contrast, *Salinibacter* and *Salisaeta* (*Salinibacteraceae*) thrive in salt lakes and crystallizer ponds and grow at maximum temperatures of 50°C in medium with at least 5% NaCl and up to saturation (Table 1). Additionally, the polar lipids of *Rhodothermus* comprise mainly DPG and PE whereas *Salinibacter, Salinivenus*, and *Salisaeta* additionally contain PC, glycolipids and halocapnines, while *Rubricoccus* and *Rubrivirga* (*Rubricoccaceae*) contain PG, but no glycolipids (Table 1). *Rhodothermus* mainly displays saturated fatty acids, whereas *Salinibacter, Salinivenus*, and *Salisaeta* contain unsaturated and C_18_ fatty acids (Table 1). We thus suggest according emendations.

The families within *Sphingobacteriales* appeared monophyletic throughout with maximum support in the GBDP tree (Figure 2). Within *Cytophagales*, the situation seems more complex. *Cytophagaceae* were not shown as monophyletic, since strong conflicting support was present regarding the positioning of *Flexibacter* and *Microscilla*, which are currently placed in that family (Nakagawa, 2011b) but here *Flexibacter* appeared problematic *per se* and thus is discussed below in more detail.

*Flexithrix* is placed apart from the remaining *Flammeovirgaceae* (*Marivirga, Roseivirga*) with 99% support in the GBDP analysis. *Flexithrix and Rapidithrix* formed a clade with reasonable bootstrap support (always >90%) in the UCT and CCT. *Marivirga* and *Roseivirga* did not form a clade either, with a conflicting branch supported by 94% in the GBDP tree. Because of their overall lower resolution, the 16S rRNA gene trees do not indicate in which of these three distinct groups of *Flammeovirgaceae* its type genus, *Flammeovirga*, is placed. For this reason, further revisions of *Flammeovirgaceae* have to be postponed until more genome sequences are available. Based on phylogenetic results (Nakagawa et al., 2002) and a polyphasic approach (Nedashkovskaya et al., 2010), the genus *Marivirga* was proposed to comprise of *M. sericea* and *M. tractuosa* (Nedashkovskaya et al., 2010). However, the physiological differentiation in that study only included *Flammeovirgaceae* genera (*Fabibacter, Fulvivirga, Marinoscillum, Reichenbachiella, Roseivirga*), and *Thermonema lapsum* (*Flammeovirgaceae*) was used as an outgroup for the 16S rRNA gene sequence-based phylogeny. A similar set of strains was investigated in the description of the two other *Marivirga* species, *M. lumbricoides* (Xu et al., 2015) and *M. atlantica* (Lin C.-Y. et al., 2015). Thus, the strain samplings in those earlier studies did not allow for detecting a closer affiliation of *Marivirga* to another family, which might explain the conflicting result obtained here.

Within *Bacteroidia, Porphyromonadaceae* appeared paraphyletic with maximum support against their monophyly in the GBDP analysis (Figure 3), as all other genera were placed in clades distinct from the one harboring *Porphyromonas*. According to the GBDP phylogeny, three families should be separated from *Porphyromonadaceae*, each corresponding to a maximally support clade. A revision of the family is currently already conducted (Hugenholtz, pers. comm.), hence no taxonomic consequences will be undertaken in our study.

The recent removal of *Butyricimonas* and *Odoribacter* from *Porphyromonadaceae* to place them in the new family *Odoribacteraceae* (Munoz et al., 2016) also requires some attention. The features of the genera that used to be included in *Porphyromonadaceae* have been reviewed in 2009 (Sakamoto et al., 2009); the statements below also extend to the more recently added genera *Falsiporphyromonas* (Wagener et al., 2014), *Macellibacteroides* (Jabari et al., 2012), and *Petrimonas* (Grabowski et al., 2005). (An issue with *Falsiporphyromonas* and *Macellibacteroides* that is beyond the scope of the current study is that these genera are nested with strong support within *Porphyromonas* and *Parabacteroides*, respectively; see Supplementary File 4.) *Butyricimonas* and *Odoribacter* (Hardham et al., 2008) differ from each other, but not clearly from the other genera, by their major metabolic end products; however, this feature does not unambiguously differentiate between the other genera either. *Butyricimonas* and *Odoribacter* differ from all other genera except *Porphyromonas* by containing iso-C_15:0_ as major fatty acid, with a low ratio of anteiso-C_15:0_ to iso-C_15:0_. *Butyricimonas* and *Odoribacter* differ from *Falsiporphyromonas* and *Porphyromonas* by their fermentative metabolism (Sakamoto et al., 2009) and partially by their lack of pigments (Nagai et al., 2010). To reflect this distribution of phenotypic features, we propose to emend the family *Odoribacteraceae* accordingly.

### Genera

Six genera were found to be non-monophyletic in the phylogenomic analysis. *Pedobacter* (*Sphingobacteriaceae*) appeared paraphyletic because *Pseudopedobacter saltans* was placed as sister group of *Pedobacter glucosidilyticus* with 100% support and *Pedobacter oryzae* as sister group of *Arcticibacter svalbardensis* and *Mucilaginibacter paludis* with <50% support (Figure 2). The UCT and CCT also showed *Pedobacter* distributed over several clades but without significant support against its monophyly. Due to the size of the genera *Mucilaginibacter* and *Pedobacter* we suggest sampling more genomes from these groups prior to drawing taxonomic conclusions.

*Flexibacter* (*Cytophagaceae*) appeared paraphyletic with high confidence in the phylogenomic tree because the clade comprising *F. litoralis* and *F. roseolus* (separated by long branches) occurred in clades with 77 and 99% support together with other genera, to the exclusion of *F. elegans*, which formed the sister group of *Microscilla marina* with maximum support (Figure 2). A paraphyletic *Flexibacter* also occurred in the UCT and CCT, with strong support against its monophyly, as *F. elegans* was also highly supported as sister group of *Microscilla marina*. *F. ruber* formed the sister group of that clade without significant support, whereas *Flexibacter flexilis* Soriano 1945, the type species of the genus (Nakagawa, 2011c), was indeed placed within the *Cytophagaceae*, with *Arcicella, Flectobacillus*, and *Pseudarcicella* as most closely related genera, without much support. *Flexibacter litoralis, F. polymorphus*, and *F. roseolus* formed a clade with reasonable bootstrap support (>90% in the CCT), which was placed in an uncertain position. Apart from tree topology, the branch lengths in the whole-genome and 16S rRNA gene tree indicated that the *Flexibacter* species are too divergent to be placed in a single genus. Additionally, *Microscilla marina* and *F. elegans* appear to be too divergent to be placed in a single genus (Figure 2).

Since its description, eleven of 17 originally supposed *Flexibacter* species were reclassified into a variety of genera (*Chitinophaga, Flavobacterium, Marivirga, Solitalea*, and *Tenacibaculum*), but it was emphasized that *Flexibacter* is still heterogeneous and should be subdivided based on additional molecular taxonomic data (Nakagawa, 2011c). Furthermore, this author mentioned that the type species, *Flecibacter flexilis*, is isolated from all other species of the genus and that *Flexibacter* should be restricted to it. The current sole distinction between the definitions of *Microscilla* and *Flexibacter* is their habitat (marine vs. terrestrial) (Reichenbach, 1989a), whereas *F. elegans* showed the same morphology and physiology as *M. marina* in previous studies (Lewin, 1969); *F. roseolus* differed from *M. marina* regarding its pigmentation and *F. ruber* by its ability to digest starch. *F. flexilis* differs from *M. marina* and *F. elegans* regarding cell length and H_2_S production, whereas the other species differ from *F. flexilis* regarding pigmentation, cell length and in the case of *F. roseolus* also H_2_S production. Thus, the *Flexibacter* species are approximately equidistant from each other regarding morphology and physiology. This also holds for *Flexibacter litoralis* and *F. polymorphus*; whereas they form sister groups with high support, their level of divergence is higher than between many other pairs of genera, as obvious from the branch lengths in the trees (Figures 2, 3; Supplementary File 4), and their morphology and physiology also differs (Lewin, 1969, 1974). Thus, based on phylogenetic position (Supplementary File 4) and phenotype, we propose to classify *F. elegans, F. litoralis, F. polymorphus, F. roseolus*, and *F. ruber* into the new genera *Eisenibacter, Bernardetia, Garritya, Hugenholtzia*, and *Thermoflexibacter*, respectively. Further, we propose the new family *Microscillaceae* to accomodate *Bernardetia* and *Eisenibacter* as well as the new family *Bernardetiaceae* to accommodate *Bernardetia, Hugenholtzia* and tentatively also *Garritya*.

Within *Prevotellaceae, Prevotella* (Shah and Collins, 1990; Willems and Collins, 1995a; Sakamoto and Ohkuma, 2012) appeared paraphyletic because *Hallella seregens* (Moore and Moore, 1994) was included within, placed as sister group of *P. dentalis* with strong support (Figure 3). This problem was also obvious in 16S rRNA gene data and was indeed noted two decades ago (Willems and Collins, 1995a). These authors refrained from taxonomic consequences, however, because they observed a 16S rRNA gene similarity of 99.8% between the type strains of *H. seregens* and *P. dentalis*; hence it could not be excluded that the two species are heterotypic synonyms (Meier-Kolthoff et al., 2013b). Furthermore, the G+C values calculated from the genomes of *H. seregens* and *P. dentalis* are almost identical, with 56.0 and 55.9%, respectively, even though this is not currently reflected in the species descriptions. Digital DNA:DNA hybridization between the genome sequences of *H. seregens* ATCC 51272^T^ and *P. dentalis* DSM 3688^T^ conducted with the recommended settings of the GGDC yielded 87.40 ± 2.36% DDH similarity and thus indicated identical species, even identical subspecies (Meier-Kolthoff et al., 2014b). We conclude that *H. seregens* is a later heterotypic synonym of *P. dentalis*.

Within *Flavobacteriaceae, Chryseobacterium* appeared paraphyletic because *Epilithonimonas tenax* was clearly nested within a well-supported branch of 21 *Chryseobacterium* species (Figure 4). Whereas support was maximal in the phylogenomic analysis, the UCT and CCT showed *Epilithonimonas* nested within a paraphyletic *Chryseobacterium*, too, but without any branch support. In the original description of *Epilithonimonas* (O'Sullivan et al., 2006) only 75% bootstrap support was obtained for the separation of *Epilithonimonas* from *Chryseobacterium*, based on a simplistic evolutionary model (Felsenstein, 2004). The most recent description of an *Epilithonimonas* species (Ge et al., 2015), still based on that model, showed moderate support for *Epilithonimonas* monophyly but none for *Chryseobacterium* monophyly; hence 16S rRNA gene analyses did not rule out that *Epilithonimonas* is nested within *Chryseobacterium*, as revealed by phylogenomic analysis. As evident from Figure 4, retaining *Epilithonimonas* by including certain *Chryseobacterium* species would require the establishment of a third genus for at least the upper branch including six *Chryseobacterium species* (*C. koreense, C. antarcticum, C. solincola, C. palustre, C. jeonii, C. bovis*) to obtain monophyletic groups. The solution to merge the two genera is thus more conservative. Moreover, the overall divergence of the clade comprising the two genera is lower than the divergence of other clades comprising only a single genus (Figures 2–4). *Chryseobacterium* has priority over *Epilithonimonas* (Vandamme et al., 1994). *Chryseobacterium* was recently emended because some summed features of MIDI-system fatty-acids profiles appeared to have frequently been misinterpreted in the literature, and a specific pattern of polar lipids remained mostly unnoticed (Montero-Calasanz et al., 2014). In detail, iso-C_15:0_ 2-OH is present in *Chryseobacterium* as part of summed feature 3 or 4 instead of C_16:1_ω7t, C_16:1_ω6c and C_16:1_ω7c; iso-C_17:1_ω7c is present instead of iso-C_17:1_ω9c; and the major polar lipids contain three common unknown lipids and two common unknown aminolipids.

Thus, the question arises whether the reported differences regarding the lipid profiles between *Epilithonimonas* and the description of *Chryseobacterium* can be traced back to misidentified fatty acids or were real differences. Table 2 shows the potentially conflicting phenotypic features. The only real difference in fatty-acid profiles appeared to be the presence of iso-C_16:0_ 3-OH, which was not explicitly mentioned for the genus *Chryseobacterium* (Montero-Calasanz et al., 2014). However, it was reported for a variety of *Chryseobacterium* species (Hugo et al., 2003; Kämpfer et al., 2003, 2011; Li et al., 2003; Kim et al., 2005; Shen et al., 2005; Young et al., 2005; Park et al., 2006; Behrendt et al., 2007, 2008; Hantsis-Zacharov and Halpern, 2007; Quan et al., 2007; Vaneechoutte et al., 2007; Szoboszlay et al., 2008; Ilardi et al., 2009; Benmalek et al., 2010; Pires et al., 2010; Wu et al., 2013) in concentrations up to 9% and thus does not constitute a real difference either. Additionally, anteiso-C_15:0_ as reported for *Epilithonimonas* is not mentioned in the description of *Chryseobacterium* but known to occur in the genus from traces to up to 8.2% (Montero-Calasanz et al., 2014). The polar-lipid patterns of *Epilithonimonas* also fit very well to that revision of *Chryseobacterium* (Table 2); the sole exception is *E. psychrophila*, for which only phosphatidylethanolamine and a single unknown lipid were reported; given its well-supported position within a core group of four *Epilithonimonas* species and the general faintness of the published thin-layer chromatogram (Ge et al., 2015), this can hardly be regarded as an argument against the unification of the genera. Moreover, the two genera have overlapping habitats and other interesting features in common, such as the production of indole-3-acetic acid for promoting plant growth (Montero-Calasanz et al., 2014; Hoang et al., 2015). Thus, the three physiological features that conflict between the original description of *Chryseobacterium* (Vandamme et al., 1994) and some or all of the *Epilithonimonas* species rather call for an emendation of *Chryseobacterium*.

**Table 2:** **Comparison of relevant phenotypic features between *Epilithonimonas* species and the description of *Chryseobacterium***.

**Feature**	**Maybe provided as**	***Chryseobacterium***	***E. tenax***	***E. lactis***	***E. ginsengisoli***	***E. xixisoli***
iso-C_15:0_		Major	(20)	23.7	17.9	29.6
iso-C_15:0_ 2-OH	summed feature 3	Major	(21.1^*^)	26.7^*^	33.1^**^	23^*^
iso-C_17:0_ 3-OH		Major	(9.8)	10	10	(4.6)
iso-C_17:1_ω7c	iso-C_17:1_ω9c	Major	(1.0^**^)	(1.1^**^)	(0.0)	(<1.0^**^)
anteiso-C_15:0_		Traces/major	(14.5)	(8.2)	(9.3)	12.9
iso-C_16:0_ 3-OH		Missing/traces	(1.9)	(1.5)	(1.4)	(2.7)
Phosphatidylethanolamine		Major	?	?	Present	Major
Unknown lipids		Three major	?	?	One to five	Four major, five in total
Unknown aminolipids		Two major	?	?	three to six	Three major
Growth at 30°C		Yes	Yes	Yes	Yes	Yes
Oxidation of glycerol		Yes	No	No	?	No
Oxidation of trehalose		Yes	Yes	Yes	?	No

*The table includes all features potentially regarded as differing between the description of Chryseobacterium and the Epilithonimonas species. A single star indicates slightly misinterpreted summed features according to the corrected description of Chryseobacterium (Montero-Calasanz et al., 2014); two stars indicate more strongly misinterpreted summed features. Values in parentheses are those not included in the respective species descriptions (O'Sullivan et al., 2006; Shakéd et al., 2010; Feng et al., 2014; Ge et al., 2015; Hoang et al., 2015). Physiological data for Chryseobacterium are from its original description (Vandamme et al., 1994). Question marks indicate missing information*.

*Muricauda* (*Flavobacteriaceae*) appeared paraphyletic in the phylogenomic tree because *Croceitalea dokdonensis* was placed as sister group of *M. lutaonensis*. Support was weak (<60%), however, and in the UCT and CCT *Croceitalea* and *Muricauda* appear as separate groups with moderate support. Thus, even though in the description of the moderate thermophile *M. lutaonens* the genus *Croceitalea* was not considered (Arun et al., 2009), our analyses do not indicate a need to revise the classification of the two genera.

*Aequorivita* (*Flavobacteriaceae*) also appeared paraphyletic in the phylogenomic tree because two *Vitellibacter* species were placed as sister group of *Aequorivita sublithincola* with 94% support. In the UCT and CCT the clade comprising both genera is maximally supported, but within the clade the two genera are intermixed without much support. The genomic divergence of the clade comprising both genera appears as lower than the divergences of many clades that contain only a single genus (Figure 3). When the genus *Vitellibacter* was introduced (Nedashkovskaya et al., 2003), type strains from the previously suggested genus *Aequorivita* (Bowman and Nichols, 2002) were not considered. The phenotypic features reported in the literature (Bowman and Nichols, 2002; Nedashkovskaya et al., 2003; Bowman, 2006; Park et al., 2009, 2014; Kim et al., 2010; Liu et al., 2013; Lin S. Y. et al., 2015; Rajasabapathy et al., 2015) for the species of two genera considerably overlap. The only stable difference is whether the cells are oxidase-positive or negative, but this alone can hardly justify two separate genera. For this reason, we propose to place *Vitellibacter* in *Aequorivita* and to emend the description of *Aequorivita* accordingly.

## Discussion

Our phylogenetic analysis revealed much agreement between genome-scale data and the current classification of *Bacteroidetes*, particularly due to the most recent revision (Munoz et al., 2016), but also a number of instances which still call for reclassifications or emendations of taxon descriptions. Prokaryotic taxonomy is not “written by taxonomists for taxonomists” (Cowan, 1971) but directly influences all microbiological disciplines. In microbial ecology, the name of a given taxon gets linked to observed characteristics of its described representatives and, further, to its inferred function in the environment. For example, in the *Bacteroidetes*-*Chlorobi* “superphylum,” *Chlorobi* (Garrity and Holt, 2001), now *Chlorobaeota* (Oren et al., 2015), are known as the obligate anaerobic green sulfur bacteria, whereas *Bacteroidetes*, now *Bacteroidaeota* (Oren et al., 2015), are known as polymer-decomposing microorganisms in the environment and the intestinal tract (Thomas et al., 2011). Currently *Halomonadaceae*, some *Bacteroidetes* (*Rhodothermaceae, Salinibacter*) and *Chlostridia* are considered as halotolerant or halophilic *Bacteria* (Oren, 2002, 2008; Bakermans, 2015). The reclassifications of the *Rhodothermia* and the *Balneolia* into separated phyla suggest that some *Bacteroidetes* remain thermotolerant (e.g., *Schleiferia* and *Thermonema*, growth at 30–60°C) or halotolerant [e.g., *Anaerophaga, Fabibacter*, and *Dyadobacter*, growth at 1–12% NaCl (w/v)] (Krieg et al., 2010), but not thermophilic (*Rhodothermus*, growth at 50–70°C) or halophilic [*Salinibacter*, growth at 15–30% NaCl (w/v)].

In other areas such as medicine and industry, the risk classification of a prokaryotic species to cause infectious diseases is based on its taxonomic classification (ABAS, 2015; ABSA, 2016). Given the peculiar ecological preferences of particularly *Rhodothermaeota and Balneolaeota*, but also of other groups reclassified here, it is likely that ecological studies using metagenomic binning or similar techniques will benefit from the further improved classification. Studies that do not distinguish between *Rhodothermaeota, Balneolaeota*, and *Bacteroidetes* may insufficiently describe microbial compositions of environmental habitats and perhaps make misleading assumptions on environmental conditions (Tsiamis et al., 2008; Baati et al., 2010; Çınar and Mutlu, 2016). A more profound effect of the proposed reclassifications might be on a potentially misleading proposal of an origin for horizontal-gene-transfer (Nelson-Sathi et al., 2015). In contrast, the CARD-FISH probes CF968 (Acinas et al., 2015) and CFB319 (Manz et al., 1996) will not hybridize with 16S rRNA gene sequences of *Rhodothermaeota* and the *Balneolaeota* (both probes have two mismatches), and thus environmental studies using this probe are not influenced by the mentioned reclassifications.

Our analysis has shown that a large proportion of G+C content values in *Bacteroidetes* species descriptions needs to be corrected. The according emendations proposed below are numerous but by no means excessive. Indeed, we do not propose to correct all G+C content values whose precision or accuracy could be improved but only those that are too imprecise or inaccurate given that the within-species deviation in G+C content is at most 1% (Meier-Kolthoff et al., 2014c). G+C content values provided in species descriptions that deviate more than 1% from the value calculated from the genome sequence, or display a range of more than 1%, obscure species affiliations and should be corrected (Riedel et al., 2014). Emendations of higher taxa regarding their G+C content have also been conducted (Scheuner et al., 2014) but we abstain from accordingly redefining *Bacteroidetes* genera here because we opine that more type-strain genome sequences per genus would be needed. Moreover, the emendation of *Flavobacterium suncheonense* has recently been conducted (Panschin et al., 2016) and needs not be considered here. For the emendations conducted below we round all G+C content values to a single decimal place to accommodate for incompletely sequenced genomes (see Supplementary File 3 for details).

Whereas conflict with the monophyly of several taxa was obvious in our genome-scale analysis, cases where a taxon was strongly supported in the proteome tree and strongly in conflict with the 16S rRNA gene, or vice versa, were not observed (Figure 5). This is crucial since the promise of phylogenomics is to yield more strongly resolved trees (which fully succeeded in this study), but this might in theory also increase the topological conflict between analyses based on distinct large sets of characters or distinct phylogenetic methods (Jeffroy et al., 2006; Klenk and Göker, 2010). For instance, horizontal gene transfer is known as a cause of topological conflict between analyses of single genes and has even been be used to argue against hierarchical classification (Bapteste and Boucher, 2009; Klenk and Göker, 2010). However, adding more genes, up to virtually all genes available in a genome, increases support in phylogenomic analyses (Breider et al., 2014), indicating that there is a strong hierarchical signal. In contrast, selection of few genes (or any number of characters not on the same order of magnitude as the overall number of genomic characters) can hardly be called genome-scale and also depends on assumptions about the relative suitability of genes for the analysis (Lienau and DeSalle, 2009; Klenk and Göker, 2010). Whole-genome methods such as GBDP avoid this issue and have the potential to yield a truly genome-based classification, but then the question arises how to avoid overestimating phylogenetic confidence (Taylor and Piel, 2004). Bootstrapping entire genes instead of single alignment positions was suggested to reduce conflict and provide more realistic support values in phylogenomic analysis (Siddall, 2010). GBDP pseudo-bootstrapping in conjunction with the greedy-with-trimming algorithm (Meier-Kolthoff et al., 2014a) is indeed conceptually closer to this partition bootstrap than to standard bootstrapping. Compared to analyses of concatenated alignments of orthologs, GBDP yielded the same topology but slightly lower support values (Riley et al., 2016). This might explain why we did not identify significant conflict with the 16S rRNA gene in this study.

We neither identified any significant conflict between phenotypic information and reclassifications suggested by genome-scale trees; rather, these called for a distinct interpretation of the available information, or highlighted known problems that just had not yet been taxonomically fixed. Many of the taxonomic problems had also been visible in the 16S rRNA gene sequence analysis in the past, provided a sufficient taxon sampling had been used. For instance, sampling *Chlorobi* was also necessary in previous (Munoz et al., 2016) and the current phylogenetic analyses to fully recognize the problematic placement of the *Balneola* clade and *Rhodothermaceae*. In other cases, missing genomes did not hinder recognizing taxonomic problems. For instance, whereas the genome sequence of the type strain of the type species of *Flexibacter* was not available to us, the need to split the genus into several genera is already visible in the unconstrained 16S rRNA gene analysis. Moreover, applying a backbone constraint is a valuable means for augmenting comprehensible sampled single-gene data with information from analyses of more genes but fewer organisms (Liu et al., 2015).

The high resolution provided by whole-genome phylogenies and the apparent lack of conflicts with data from other sources renders genome-based approaches quite promising for shaping the future of *Bacteroidetes* taxonomy. This holds even though the present study led to a number of changes in taxonomic opinion, listed below in the order of decreasing Linnaean ranks and with proposed names first, followed by the emendations. We additionally propose to rephrase the “*Bacteroidetes-Chlorobi* superphylum” to “*Bacteroidaeota-Rhodothermaeota-Balneolaeota-Chlorobaeota* (BRBC) superphylum,” considering the recent proposal to include the phylum rank in taxonomy (Oren et al., 2015), and considering the new phyla *Rhodothermaeota* and *Balneolaeota* as part of the former “*Bacteroidetes-Chlorobi* superphylum” (Figure 2).

### Taxonomic consequences

#### Description of *Balneolaeota*, phyl. nov.

Bal.ne.o.lae.o'ta (N.L. fem. n. *Balneola*, type genus of the type order of the phylum, -*aeota* ending to denote phylum; N.L. neut. pl. n. *Balneolaeeota*, the phylum of the class *Balneolia*).

The description is the same as for the class *Balneolia*.

This phylum is described on the basis of 16S rRNA gene and whole-genome phylogenetic analysis. The type (and currently sole) order of the phylum is *Balneolales*. The phylum currently contains a sole class, *Balneolia*.

#### Emended description of *Rhodothermaeota*, Munoz et al. 2016

The description is the same as given by Munoz et al. (2016) with the following modification. Cells are aerobic. The phylum has been additionally circumscribed on the basis of whole-genome phylogenetic analysis.

This change was necessary due to the removal of *Balneolia* from the phylum *Rhodothermaeota*.

#### Emended description of *Balneolia* Munoz et al. 2016

The description is the same as given by Munoz et al. (2016) with the following modification. The class *Balneolia* is part of the phylum *Balneolaeota* and has been additionally circumscribed on the basis of whole-genome phylogenetic analysis.

This change was necessary due to the removal of *Balneolia* from the phylum *Rhodothermaeota*. The description is the same as for the order *Balneolales*.

#### Emended description of *Cytophagia* Nakagawa 2012

The description is the same as given by Nakagawa (2011a) with the following modification. Flagella are not found.

This change was necessary due to the removal of *Balneola* from *Cytophagia*.

#### Description of *Saprospiria*, class. nov.

Sa.pros.pi'ri.a (N.L. fem. n. *Saprospira*, type genus of the type order of the class, -*ia* ending to denote a class; N.L. fem. pl. n. *Saprospiria*, the class of the order *Saprospirales*).

The description is the same as for the order *Saprospirales*.

This class is described on the basis of 16S rRNA gene and whole-genome phylogenetic analysis. The type and currently sole order of the class is *Saprospirales*.

#### Description of *Saprospirales*, ord. nov.

Sa.pro.spi.ra'les (N.L. fem. n. *Saprospira*, type genus of the order, suff. -*ales* ending to denote order; N.L. fem. pl. n. *Saprospirales*, the order of the genus *Saprospira*).

The description is the same as for the family *Saprospiraceae*.

This order is described on the basis of 16S rRNA gene and whole-genome phylogenetic analysis. The type genus is *Saprospira*. The currently sole family of the order is *Saprospiraceae*.

#### Emended description of *Chitinophagales* Munoz et al. 2016

The description is as given by Munoz et al. (2016), with the following changes. Currently encompasses only the family *Chitinophagaceae*. This change was necessary due to the removal of *Saprospiraceae* from the order *Chitinophagales*.

#### Description of *Lewinellaceae*, fam. nov.

Le.wi.nel.la'ce.ae (L. fem. n. *Lewinella* type genus of the family; -aceae ending to denote a family; N.L. fem. pl. n. *Lewinellaceae* the *Lewinella* family).

Cells are ensheathed, Gram-stain negative flexible rods (up to 3 μm) that form long filaments (up to 150 μm) and are motile by gliding. Typical fatty acids are iso-C_15:0_, iso-C_15:1_, iso-C_17:0_ 3-OH and summed feature 3 (either C_16:1_ ω7c/C_16:1_ ω6c or C_16:1_ ω7c/iso-C_15:0_ 2-OH). Seawater is required for growth. Flexirubin-type pigments are not produced. Carotenoid-type pigments are produced. The respiratory quinone is MK-7. The genomic G+C content is around 45–53%.

This family belongs to the phylum *Bacteroidetes*, order *Saprospirales* ord. nov., class *Saprospiria* class. nov., and currently comprises only the type genus, *Lewinella*.

#### Description of *Haliscomenobacteraceae*, fam. nov.

Ha.lis.co.me.no.bac.te.ra'ce.ae (L. fem. n. *Haliscomenobacter* type genus of the family; -aceae ending to denote a family; N.L. fem. pl. n. *Haliscomenobacteraceae* the *Haliscomenobacter* family).

Cells are Gram-stain negative, non-motile long rods (up to 5 μm) that form long needle-like filaments (up to 300 μm). Some are enclosed by a narrow, hardly visible hyaline sheath (*Haliscomenobacter*). Typical fatty acids are iso-C_15:0_, summed feature 3 (either C_16:1_ ω7c/C_16:1_ ω6c or C_16:1_ ω7c/iso-C_15:0_ 2-OH) and either iso-C_17:0_ 3-OH or iso-C_15:0_ 3-OH. Flexirubin-type pigments are not produced. Carotenoid-type pigments are produced. The respiratory quinone is MK-7. Cells are oxidase-positive. The genomic G+C content is around 47–54%.

This family belongs to the phylum *Bacteroidetes*, order *Saprospirales* ord. nov., class *Saprospiria* class. nov., and currently comprises the genera *Haliscomenobacter* (the type genus), *Phaeodactylibacter* and *Portibacter*.

#### Description of *Microscillaceae*, fam. nov.

Mi.cros.cil.la'ce.ae (L. fem. n. *Microscilla* type genus of the family; -aceae ending to denote a family; N.L. fem. pl. n. *Microscillaceae* the *Microscilla* family).

Cells are Gram-stain negative, chemoorganotrophs and strict aerobes. Cells are flexible threads motile by gliding. Gelatin is degraded, but not agar, alginate, or carboxymethyl cellulose. The major respiratory quinone is MK-7. The genomic G+C content is around 40–50%.

This family belongs to the phylum *Bacteroidetes*, order *Cytophagales*, class *Cytophagia*, and comprises currently the genera *Microscilla* (the type genus) and *Eisenibacter* gen. nov.

#### Description of *Bernardetiaceae*, fam. nov.

Ber.nar.de.ti.a'ce.ae (L. fem. n. *Bernardetia* type genus of the family; -aceae ending to denote a family; N.L. fem. pl. n. *Bernardetiaceae* the *Bernardetia* family).

Cells are Gram-stain negative, chemoorganotrophs and strict aerobes that are motile by gliding. Threads are formed, with and without cross-walls. Carotenoids, saproxanthin or flexixanthin, are produced. Oxidase activity is present, catalase activity is absent. Gelatin is hydrolyzed, but neither cellulose nor agar. The major respiratory quinone is MK-7. The genomic G+C content is 29–38%.

This family belongs to the phylum *Bacteroidetes*, order *Cytophagales*, class *Cytophagia*, and currently comprises the genera *Bernardetia* (the type genus), *Hugenholtzia*, and tentatively also *Garritya*.

#### Emended description of *Balneolaceae* Munoz et al. 2016

The description is the same as given by Munoz et al. (2016) with the following modification.

Cells are non-motile or motile by means of flagella. The dominant fatty acids are iso-C_15:0_ and other non-hydroxy branched-chain fatty acids. Major polar lipids are diphosphatidylglycerol, phosphatidylethanolamine and either phosphatidylglycerol (*Balneola, Gracilimonas*) or phosphatidylcholine (*Aliifodinibius, Fodinibius*). The major menaquinone is MK-7. The genomic G+C content varies around 45%.

#### Emended description of *Salinibacteraceae* Munoz et al. 2016

The description is the same as given by Munoz et al. (2016) with the following modification.

Major polar lipids are diphosphatidylglycerol (cardiolipid) and diphosphatidylcholin. Some species possess halocapnines. The major fatty acids are iso-C_15:0_, C_18:1_ ω7c, summed feature 3 (C_16:1_ ω6c and/or C_16:1_ ω7c).

#### Emended description of *Rhodothermaceae* Ludwig et al. 2012

The description is the same as given by Ludwig et al. (2012) with the following modification.

Major polar lipids are diphosphatidylglycerol, phosphatidylethanolamine and phosphatidylglycerol. The major fatty acids are iso-C_16:0_, iso-C_17:0_, anteiso-C_17:0_. The genomic G+C content varies around 65%.

#### Emended description of *Odoribacteraceae* Munoz et al. 2016

The description is the same as given by Munoz et al. (2016) with the following modification.

Cells are non-motile. Metabolism fermentative, major end products are diverse organic acids. Major menaquinones are MK-9 and MK-10. Major fatty acid iso-C_15:0_, with a low ratio of anteiso-C_15:0_ to iso-C_15:0_. The genomic G+C content varies around 40–50%.

#### Emended description of *Saprospiraceae* Krieg et al. 2012

The description is as given by Krieg et al. (2012), with the following changes.

Cells are long rods (up to 3.5 μm) that form long helical filaments (up to 500 μm) and are motile by gliding. NaCl is required for growth and some can be tolerate NaCl at a concentration of up to 9% (w/v). Cytochrome oxidase and catalase activities are variable. Flexirubin-type pigments are not produced. Carotenoid-type pigments are produced. The respiratory quinone is MK-7. The genomic G+C content is around 33–48%.

This family belongs to the phylum *Bacteroidetes*, order *Saprospirales* ord. nov., class *Saprospiria* class. nov., and currently comprises the genera *Saprospira* (the type genus) and *Aureispira*.

#### Description of *Bernardetia*, gen. nov.

Ber.nar.de'ti.a (N.L. masc. n. *Bernardet* named after Jean-François Bernardet, researcher at INRA Research Center, Jouy-en-Josas, France, and chairman of ICSP subcommittee on the taxonomy of aerobic *Bacteroidetes*; N.L. fem. n. *Bernardetia* a genus named after Jean-François Bernardet).

On the basis of 16S rRNA gene sequence analysis, the genus represents a separate branch within the order *Cytophagales*. With moderate support it forms a clade together with *Hugenholtzia* gen. nov. and perhaps also *Garritya* gen. nov. but the three type species, respectively, are comparatively distant from each other in terms of sequence divergence, morphology and physiology. The type species of the genus is *Bernardetia litoralis* comb. nov.

#### Description of *Bernardetia litoralis*, comb. nov.

B. li.to.ra'lis (L. masc. adj. *litoralis*, belonging to the sea shore).

Basonym: *Flexibacter litoralis* Lewin 1969

The description is the same as for *Flexibacter litoralis* (Lewin, 1969). The type strain is ATCC 23117 = DSM 6794.

#### Description of *Garritya*, gen. nov.

Gar.ri'ty.a (N.L. masc. n. *Garrity* named after George M. Garrity, professor at Michigan State University, East Lansing, MI, USA, the former editor of Bergey's manual and current chief editor of Standards in Genomic Sciences; N.L. fem. n. *Garritya* a genus named after George M. Garrity).

The description is the same as for the species *Garritya polymorpha*, comb. nov., as its known features, while scarce, already differentiate at the genus level.

On the basis of 16S rRNA gene sequence and phylogenomic analysis, the genus represents a separate branch within the order *Cytophagales* with an uncertain affiliation to a family. It potentially forms a clade together with *Bernardetia* gen. nov. and *Hugenholtzia* gen. nov. but the three type species, respectively, are comparatively distant from each other in terms of sequence divergence, morphology and physiology. The type (and currently sole) species of the genus is *Garritya polymorpha*.

#### Description of *Garritya polymorpha*, comb. nov.

G. po.ly.mor'pha (N.L. fem. adj. *polymorpha*, variable in form).

Basonym: *Flexibacter polymorphus* Lewin 1974

The description is the same as for *Flexibacter polymorphus* (Lewin, 1974). The type strain is ATCC 27820 = DSM 9678.

#### Description of *Hugenholtzia*, gen. nov.

Hu.gen.hol'tzi.a (N.L. masc. n. *Hugenholtz* named after Philip Hugenholtz, Professor at University of Queensland, Brisbane, Qld, Australia, who played a decisive role in starting the GEBA project; N.L. fem. n. *Hugenholtzia* a genus named after Philip Hugenholtz).

The description is the same as for the species *Hugenholtzia roseola*, comb. nov., as its known features, while scarce, already differentiate at the genus level.

On the basis of 16S rRNA gene sequence and phylogenomic analysis, the genus represents a separate branch within the order *Cytophagales*. With moderate support it forms a clade together with *Bernardetia* gen. nov. and perhaps also *Garritya* gen. nov. but the three type species, respectively, are comparatively distant from each other in terms of sequence divergence, morphology and physiology. The type species of the genus is *Hugenholtzia roseola*.

#### Description of *Hugenholtzia roseola*, comb. nov.

H. ro.se'o.la (N.L. fem. dim. adj. *roseola*, with a rosy shading).

Basonym: *Flexibacter roseolus* Lewin 1969

The description is the same as for *Flexibacter roseolus* (Lewin, 1969) with the following restriction. The genomic G+C content is 42.2%. The type strain is ATCC 23088 = DSM 9546.

#### Description of *Thermoflexibacter*, gen. nov.

Ther.mo.fle.xi.bac'ter (Gr. adj. thermos hot; L. part. adj. flexus bent, winding; N.L. masc. n. bacter from Gr. neut. n. baktron little stick or rod; N.L. masc. n. *Thermoflexibacter* a thermophilic flexible rod. The type strain GEY was isolated from a Geysir in Iceland, can grow in freshwater medium of up to 45°C and forms very long threads of more than 50 μm length.

The description is the same as for the species *Thermoflexibacter ruber*, comb. nov., as its known features, while scarce, already differentiate at the genus level.

On the basis of 16S rRNA gene sequence analysis as well as previously published physiological and morphological data, the genus represents a branch of uncertain affiliation within the order *Cytophagales*. The 16S rRNA gene quite weakly supports a sister-group relationship of this genus and the new family *Microscillaceae*. The type (and currently sole) species of the genus is *Thermoflexibacter ruber*.

#### Description of *Thermoflexibacter ruber*, comb. nov.

T. ru'bra (L. fem. adj. *rubra*, red).

Basonym: *Flexibacter ruber* Lewin 1969

The description is the same as for *Flexibacter ruber* (Lewin, 1969). The type strain is GEY = ATCC 23103 = DSM 9560.

#### Description of *Eisenibacter*, gen. nov.

Ei.se.ni.bac'ter (N.L. masc. n. *Eisen* named after Jonathan A. Eisen, professor at University of California, Davis, CA, USA, who played a decisive role in starting the GEBA project; N.L. masc. n. *bacter* a rod; N.L. masc. n. *Eisenibacter* a rod named after Jonathan A. Eisen).

The description is the same as for the species *Eisenibacter elegans*, comb. nov., as its known features, while scarce, already differentiate at the genus level.

On the basis of 16S rRNA gene sequence analysis as well as previously published physiological and morphological data, the genus represents a separate branch within the order *Cytophagales*. The 16S rRNA gene strongly supports a sister-group relationship of this genus and *Microscilla*. The type (and currently sole) species of the genus is *Eisenibacter elegans*.

#### Description of *Eisenibacter elegans*, comb. nov.

E. e'le.gans (L. masc. adj. elegans refined, fashionable, elegant).

Basonym: *Flexibacter elegans* (ex Lewin 1969, non Soriano, 1945) Reichenbach 1989b, 2067^AL^

The description is the same as for *Flexibacter elegans* (Reichenbach, 1989a). The type strain is ATCC 23112 = DSM 3317 = JCM 21159 = LMG 10750 = NBRC 15055 = Lewin NZ-1.

#### Emended description of *Aequorivita* (Bowman and Nichols 2002) Park et al. 2009

The description is as given by Park et al. (2009) with the following modifications. Gliding motility is present or absent, flexirubin production is variable, oxidase reaction is positive or negative. The genomic G+C content varies around 37%.

This change was necessary due to the inclusion of the *Vitellibacter* species in *Aequorivita*.

#### Description of *Aequorivita aestuarii*, comb. nov.

A. aes.tu.a'ri.i (L. gen. n. *aestuarii*, of a tidal flat).

Basonym: *Vitellibacter aestuarii* Kim et al. 2010

The description is the same as for *Vitellibacter aestuarii* (Kim et al., 2010). The type strain is JC2436 = IMSNU 14137 = KACC 13727.

#### Description of *Aequorivita echinoideorum*, comb. nov.

A. e.chi.no.i.de.o'rum (N.L. gen. n. *echinoideorum*, of *Echinoidea*, sea urchins).

Basonym: *Vitellibacter echinoideorum* Lin et al. 2015

The description is the same as for *Vitellibacter echinoideorum* (Lin S. Y. et al., 2015). The type strain is CC-CZW007 = BCRC 80886 = JCM 30378.

#### Description of *Aequorivita nionensis*, comb. nov.

A. ni.o.nen'sis (N.L. adj. *nionensis*, derived from the acronym of the National Institute of Oceanography, NIO).

Basonym: *Vitellibacter nionensis* Rajasabapathy et al., 2015

The description is the same as for *Vitellibacter nionensis* (Rajasabapathy et al., 2015). The type strain is VBW088 = KCTC 32420 = MCC 2354.

#### Description of *Aequorivita soesokkakensis*, comb. nov.

A. soe.sok.ka.ken'sis (N.L. adj. *soesokkakensis*, pertaining to the Soesokkak area)

Basonym: *Vitellibacter soesokkakensis* Park et al., 2014

The description is the same as for *Vitellibacter soesokkakensis* (Park et al., 2014) with the following modification. The genomic G+C content of the type strain is 37.8%. The type strain is RSSK-12 = KCTC 32536 = CECT 8398.

#### Description of *Aequorivita vladivostokensis*, comb. nov.

A. vla.di.vos.to.ken'sis (N.L. adj. vladivostokensis, pertaining to the city of Vladivostok)

Basonym: *Vitellibacter vladivostokensis* Nedashkovskaya et al. 2003

The description is the same as for *Vitellibacter vladivostokensis* (Nedashkovskaya et al., 2003). The type strain is KMM 3516 = NBRC 16718.

#### Emended description of *Aliifodinibius* Wang et al. 2013

The description is the same as given by Wang et al. (2013a) with the following modification. The genus *Aliifodinibius* is a member of the phylum *Balneolaeota*.

This change was necessary due to the removal of *Aliifodinibius* from *Rhodothermaeota*.

#### Emended description of *Chryseobacterium* (Vandamme et al. 1994) Montero-Calasanz et al. 2014

The description is as given by Montero-Calasanz et al. (2014) with the following modification. Almost all strains grow at 30°C. Most, but not all, strains oxidize glycerol and trehalose.

On the basis of phylogenomic analysis and a re-assessment of 16S rRNA gene sequence analyses and phenotypic features published earlier, the genus *Epilithonimonas* should be included in *Chryseobacterium*.

#### Description of *Chryseobacterium ginsengiterrae*, nom. nov.

C. gin.sen.gi.ter'rae (N.L. n. *ginsengum* ginseng; L. n. *terra* soil; N.L. gen. n. *ginsengiterrae* of soil from a ginseng field).

Basonym: *Epilithonimonas ginsengisoli* Hoang et al. 2015 (the name *Chryseobacterium ginsengisoli* has already been validly published, hence a new epithet must be chosen to avoid homonyms)

The description is the same as for *Epilithonimonas ginsengisoli* (Hoang et al., 2015) with the following modification. Summed feature 3 should be interpreted as iso-C_15:0_ 2-OH (Montero-Calasanz et al., 2014). The type strain is DCY78 = JCM 19896 = KCTC 32174.

#### Description of *Chryseobacterium halperniae*, nom. nov.

C. hal.per'ni.ae (N.L. gen. n. *halperniae* of Halpern, named after Malka Halpern, Professor at University of Haifa, Haifa, Israel, whose team isolated *E. lactis*).

Basonym: *Epilithonimonas lactis* Shakéd et al. 2010 (the name *Chryseobacterium lactis* has already been validly published, hence a new epithet must be chosen to avoid homonyms)

The description is the same as for *Epilithonimonas lactis* (Shakéd et al., 2010). The type strain is DSM 19921 = H1 = LMG 24401.

#### Description of *Chryseobacterium psychrotolerans*, comb. nov.

C. psy.chro.to'le.rans. (Gr. adj. *psychros* cold; L. pres. part. *tolerans* tolerating; N.L. part. adj. *psychrotolerans* cold-tolerating).

Basonym: *Epilithonimonas psychrotolerans* Ge et al. 2015

The description is the same as for *Epilithonimonas psychrotolerans* (Ge et al., 2015) with the following modification. Summed feature 3 should be interpreted as iso-C_15:0_ 2-OH (Montero-Calasanz et al., 2014). The type strain is CCTCC AB 207182 = NRRL B-51307 = TSBY 57.

#### Description of *Chryseobacterium tenax*, comb. nov.

C. te'nax (L. fem. adj. *tenax* sticky, holding firm).

Basonym: *Epilithonimonas tenax* O'Sullivan et al. 2006

The description is the same as for *Epilithonimonas tenax* (O'Sullivan et al., 2006). Lipids were not mentioned in the original description and thus the published corrections for *Chryseobacterium* (Montero-Calasanz et al., 2014) do not apply. The type strain is DSM 16811 = EP105 = NCIMB 14026.

#### Description of *Chryseobacterium xixisoli*, comb. nov.

C. xi.xi.so'li (N.L. n. *xixi* of Xixi, a geographical name; L. gen. n. *soli* of soil; N.L. fem. gen. n. *xixisoli* of soil from Xixi).

Basonym: *Epilithonimonas xixisoli* Feng et al. 2014

The description is the same as for *Epilithonimonas xixisoli* (Feng et al., 2014). The type strain is CGMCC 1.12802 = NBRC 110387 = S31.

#### Emended description of *Flexibacter Soriano* 1945

The description is the same as for the species *Flexibacter flexilis*, as its known features, while scarce, already differentiate at the genus level.

On the basis of 16S rRNA gene sequence analysis as well as previously published physiological and morphological data, the type species of the genus represents a separate branch within the order *Cytophagales*, and must be separated from all other species formerly classified in *Flexibacter*. The description of the genus must accordingly be restricted.

#### Emended description of *Akkermansia muciniphila* Derrien et al. 2004

The description is as given by Derrien et al. (2004) with the following modification. The genomic G+C content of the type strain is 55.8%.

#### Emended description of *Algoriphagus marincola* (Yoon et al. 2004) Nedashkovskaya et al. 2007

The description is as given by Nedashkovskaya et al. (2007b) with the following modification. The genomic G+C content of the type strain is 41.9%.

#### Emended description of *Algoriphagus terrigena* Yoon et al. 2006

The description is as given by Yoon et al. (2006) with the following modification. The genomic G+C content of the type strain is 47.8%.

#### Emended description of *Alistipes putredinis* (Weinberg et al. 1937) Rautio et al. 2003

The description is as given by Rautio et al. (2003) with the following modification. The genomic G+C content of the type strain is 53.3%.

#### Emended description of *Alistipes shahii* Song et al. 2006

The description is as given by Song et al. (2006) with the following modification. The genomic G+C content of the type strain is 57.6%.

#### Emended description of *Alkaliflexus imshenetskii* Zhilina et al. 2005

The description is as given by Zhilina et al. (2004) with the following restriction. The genomic G+C content of the type strain is 42.7%.

#### Emended description of *Alloprevotella tannerae* (Moore et al. 1994) Downes et al. 2013

The description is as given by Downes et al. (2013) with the following modification. The genomic G+C content of the type strain is 46.6%.

#### Emended description of *Arcticibacter svalbardensis* Prasad et al. 2013

The description is as given by Prasad et al. (2013) with the following restriction. The genomic G+C content of the type strain is 38.2%.

#### Emended description of *Arenibacter latericius* Ivanova et al. 2001 emend. Nedashkovskaya et al. 2006

The description is as given by Nedashkovskaya et al. (2006c) with the following modification. The genomic G+C content of the type strain is 36.8%.

#### Emended description of *Bacteroides caccae* Johnson et al. 1986

The description is as given by Johnson et al. (1986) with the following modification. The genomic G+C content of the type strain is 41.9%.

#### Emended description of *Bacteroides cellulosilyticus* Robert et al. 2007

The description is as given by Robert et al. (2007) with the following modification. The genomic G+C content of the type strain is 42.7%.

#### Emended description of *Bacteroides coprophilus* Hayashi et al. 2007

The description is as given by Hayashi et al. (2007a) with the following modification. The genomic G+C content of the type strain is 45.7%.

#### Emended description of *Bacteroides coprosuis* Whitehead et al. 2005

The description is as given by Whitehead et al. (2005) with the following modification. The genomic G+C content of the type strain is 35.0%.

The genome sequence-derived G+C content was reported earlier (Land et al., 2011) but no taxonomic consequences were drawn.

#### Emended description of *Bacteroides dorei* Bakir et al. 2006

The description is as given by Bakir et al. (2006b) with the following modification. The genomic G+C content of the type strain is 42.0%.

#### Emended description of *Bacteroides eggerthii* Holdeman and Moore 1974

The description is as given by Holdeman and Moore (1974) with the following modification. The genomic G+C content of the type strain is 44.6%.

#### Emended description of *Bacteroides fragilis* (Veillon and Zuber 1898) Castellani and Chalmers 1919

The description is as given by Castellani and Chalmers (1919) with the following addition. The genomic G+C content of the type strain is 43.1%.

#### Emended description of *Bacteroides graminisolvens* Nishiyama et al. 2009

The description is as given by Nishiyama et al. (2009) with the following modification. The genomic G+C content of the type strain is 41.5%.

#### Emended description of *Bacteroides helcogenes* Benno et al. 1983

The description is as given by Benno et al. (1983) with the following modification. The genomic G+C content of the type strain is 44.7%.

The genome sequence-derived G+C content was reported earlier (Pati et al., 2011c) but no taxonomic consequences were drawn.

#### Emended description of *Bacteroides intestinalis* Bakir et al. 2006

The description is as given by Bakir et al. (2006a) with the following modification. The genomic G+C content of the type strain is 42.7%.

#### Emended description of *Bacteroides massiliensis* Fenner et al. 2005

The description is as given by Fenner et al. (2005) with the following modification. The genomic G+C content of the type strain is 42.7%.

#### Emended description of *Bacteroides ovatus* Eggerth and Gagnon 1932

The description is as given by Eggerth and Gagnon (1932) with the following addition. The genomic G+C content of the type strain is 41.9%.

#### Emended description of *Bacteroides propionicifaciens* Ueki et al. 2008

The description is as given by Ueki et al. (2008) with the following restriction. The genomic G+C content of the type strain is 38.0%.

#### Emended description of *Bacteroides thetaiotaomicron* (Distaso 1912) Castellani and Chalmers 1919

The description is as given by Castellani and Chalmers (1919) with the following addition. The genomic G+C content of the type strain is 42.9%.

#### Emended description of *Bacteroides uniformis* Eggerth and Gagnon 1932

The description is as given by Eggerth and Gagnon (1932) with the following addition. The genomic G+C content of the type strain is 46.4%.

#### Emended description of *Bacteroides vulgatus* Eggerth and Gagnon 1932

The description is as given by Eggerth and Gagnon (1932) with the following addition. The genomic G+C content of the type strain is 42.2%.

#### Emended description of *Balneola vulgaris* Urios et al. 2006

The description is as given by Urios et al. (2006) with the following modification. The genomic G+C content of the type strain is 39.8%.

#### Emended description of *Barnesiella intestinihominis* Morotomi et al. 2008

The description is as given by Morotomi et al. (2008) with the following modification. The genomic G+C content of the type strain is 43.9%.

#### Emended description of *Belliella baltica* Brettar et al. 2004

The description is as given by Brettar et al. (2004) with the following modification. The genomic G+C content of the type strain is 36.8%.

#### Emended description of *Bergeyella zoohelcum* (Holmes et al. 1987) Vandamme et al. 1994

The description is as given by Vandamme et al. (1994) with the following restriction. The genomic G+C content of the type strain is 36.1%.

#### Emended description of *Blastopirellula marina* (Schlesner 1987) Schlesner et al. 2004

The description is as given by Schlesner et al. (2004) with the following restriction. The genomic G+C content of the type strain is 57.0%.

#### Emended description of *Butyricimonas synergistica* Sakamoto et al. 2009 emend. Sakamoto et al. 2014

The description is as given by Sakamoto et al. (2014) with the following restriction. The genomic G+C content of the type strain is 44.8%.

#### Emended description of *Butyricimonas virosa* Sakamoto et al. 2009 emend. Sakamoto et al. 2014

The description is as given by Sakamoto et al. (2014) with the following restriction. The genomic G+C content of the type strain is 42.3%.

#### Emended description of *Capnocytophaga gingivalis* Leadbetter et al. 1982 emend. London et al. 1985

The description is as given by London et al. (1985) with the following restriction. The genomic G+C content of the type strain is 40.5%.

#### Emended description of *Capnocytophaga sputigena* Leadbetter et al. 1982

The description is as given by Leadbetter et al. (1979) with the following modification. The genomic G+C content of the type strain is 38.4%.

#### Emended description of *Cellulophaga algicola* Bowman 2000

The description is as given by Bowman (2000) with the following restriction. The genomic G+C content of the type strain is 33.8%.

The genome sequence-derived G+C content was reported earlier (Abt et al., 2011a) but no taxonomic consequences were drawn.

#### Emended description of *Cellulophaga lytica* (Lewin 1969) Johansen et al. 1999

The description is as given by Johansen et al. (1999) with the following modification. The genomic G+C content of the type strain is 32.1%.

The genome sequence-derived G+C content was reported earlier (Pati et al., 2011a) but no taxonomic consequences were drawn.

#### Emended description of *Chitinophaga japonensis* (Fujita et al. 1997) Kämpfer et al. 2006

The description is as given by Kämpfer et al. (2006) with the following modification. The genomic G+C content of the type strain is 53.0%.

#### Emended description of *Chitinophaga pinensis* Sangkhobol and Skerman 1981

The description is as given by Sangkhobol and Skerman (1981) with the following addition. The genomic G+C content of the type strain is 45.2%.

The genome sequence-derived G+C content was reported earlier (Glavina Del Rio et al., 2010) but no taxonomic consequences were drawn.

#### Emended description of *Chitinophaga sancti* (Lewin 1969) Kämpfer et al. 2006

The description is as given by Kämpfer et al. (2006) with the following modification. The genomic G+C content of the type strain is 44.2%.

#### Emended description of *Chlorobium limicola* Nadson 1906 emend. Imhoff 2003

The description is as given by Imhoff (2003) with the following restriction. The genomic G+C content of the type strain is 51.3%.

#### Emended description of *Chlorobium phaeobacteroides* Pfennig 1968 emend. Imhoff 2003

The description is as given by Imhoff (2003) with the following modification. The genomic G+C content of the type strain is 48.4%.

#### Emended description of *Chloroherpeton thalassium* Gibson et al. 1985

The description is as given by Gibson et al. (1984) with the following restriction. The genomic G+C content of the type strain is 45.0%.

#### Emended description of *Chryseobacterium antarcticum* (Yi et al. 2005) Kämpfer et al. 2009

The description is as given by Kämpfer et al. (2009a) with the following modification. The genomic G+C content of the type strain is 36.1%.

#### Emended description of *Chryseobacterium aquaticum* Kim et al. 2008

The description is as given by Kim et al. (2008) with the following modification. The genomic G+C content of the type strain is 33.9%.

#### Emended description of *Chryseobacterium caeni* Quan et al. 2007

The description is as given by Quan et al. (2007) with the following modification. The genomic G+C content of the type strain is 36.6%.

#### Emended description of *Chryseobacterium formosense* Young et al. 2005

The description is as given by Young et al. (2005) with the following addition. The genomic G+C content of the type strain is 34.8%.

#### Emended description of *Chryseobacterium gallinarum* Kämpfer et al. 2014

The description is as given by Kämpfer et al. (2014) with the following addition. The genomic G+C content of the type strain is 37.3%.

#### Emended description of *Chryseobacterium gleum* (Holmes et al. 1984) Vandamme et al. 1994 emend. Montero-Calasanz et al. 2014

The description is as given by Montero-Calasanz et al. (2014) with the following restriction. The genomic G+C content of the type strain is 36.8%.

#### Emended description of *Chryseobacterium greenlandense* Loveland-Curtze et al. 2009

The description is as given by Loveland-Curtze et al. (2009) with the following restriction. The genomic G+C content of the type strain is 34.1%.

#### Emended description of *Chryseobacterium jeonii* (Yi et al. 2005) Kämpfer et al. 2009

The description is as given by Kämpfer et al. (2009a) with the following modification. The genomic G+C content of the type strain is 34.9%.

#### Emended description of *Chryseobacterium koreense* (Kim et al. 2004) Kämpfer et al. 2009

The description is as given by Kämpfer et al. (2009b) with the following modification. The genomic G+C content of the type strain is 40.1%.

#### Emended description of *Chryseobacterium kwangjuense* Sang et al. 2013

The description is as given by Sang et al. (2013) with the following modification. The genomic G+C content of the type strain is 38.5%.

#### Emended description of *Chryseobacterium luteum* Behrendt et al. 2007 emend. Montero-Calasanz et al. 2014

The description is as given by Montero-Calasanz et al. (2014) with the following addition. The genomic G+C content of the type strain is 37.3%.

#### Emended description of *Chryseobacterium palustre* Pires et al. 2010

The description is as given by Pires et al. (2010) with the following modification. The genomic G+C content of the type strain is 41.5%.

#### Emended description of *Chryseobacterium piperi* Strahan et al. 2011

The description is as given by Strahan et al. (2011) with the following modification. The genomic G+C content of the type strain is 35.2%.

#### Emended description of *Chryseobacterium soli* Weon et al. 2008

The description is as given by Weon et al. (2008) with the following modification. The genomic G+C content of the type strain is 36.4%.

#### Emended description of *Chryseobacterium solincola* Benmalek et al. 2010

The description is as given by Benmalek et al. (2010) with the following modification. The genomic G+C content of the type strain is 39.5%.

#### Emended description of *Croceitalea dokdonensis* Lee et al. 2008

The description is as given by Lee et al. (2008) with the following modification. The genomic G+C content of the type strain is 41.9%.

#### Emended description of *Cyclobacterium amurskyense* Nedashkovskaya et al. 2005

The description is as given by Nedashkovskaya et al. (2005a) with the following modification. The genomic G+C content of the type strain is 38.3%.

#### Emended description of *Cyclobacterium marinum* (Raj 1976) Raj and Maloy 1990

The description is as given by Raj and Maloy (1990) with the following modification. The genomic G+C content of the type strain is 38.1%.

#### Emended description of *Cyclobacterium qasimii* Shivaji et al. 2012

The description is as given by Shivaji et al. (2012) with the following modification. The genomic G+C content of the type strain is 38.8%.

#### Emended description of *Cytophaga aurantiaca* (ex Winogradsky 1929) Reichenbach 1989

The description is as given by Reichenbach (1989b) with the following restriction. The genomic G+C content of the type strain is 37.1%.

#### Emended description of *Cytophaga hutchinsonii* Winogradsky 1929

The description is as given by Winogradsky (1929) with the following addition. The genomic G+C content of the type strain is 38.8%.

#### Emended description of *Dyadobacter beijingensis* Dong et al. 2007

The description is as given by Dong et al. (2007) with the following modification. The genomic G+C content of the type strain is 52.1%.

#### Emended description of *Dyadobacter crusticola* Reddy and Garcia-Pichel 2005

The description is as given by Reddy and Garcia-Pichel (2005) with the following modification. The genomic G+C content of the type strain is 46.7%.

#### Emended description of *Dyadobacter fermentans* Chelius and Triplett 2000

The description is as given by Chelius and Triplett (2000) with the following modification. The genomic G+C content of the type strain is 51.5%.

The genome sequence-derived G+C content was reported earlier (Lang et al., 2009) but no taxonomic consequences were drawn.

#### Emended description of *Dyadobacter ginsengisoli* Liu et al. 2006

The description is as given by Liu et al. (2006) with the following modification. The genomic G+C content of the type strain is 49.0%.

#### Emended description of *Dysgonomonas gadei* Hofstad et al. 2000

The description is as given by Hofstad et al. (2000) with the following addition. The genomic G+C content of the type strain is 39.6%.

#### Emended description of *Dysgonomonas mossii* Lawson et al. 2002

The description is as given by Lawson et al. (2002) with the following modification. The genomic G+C content of the type strain is 37.5%.

#### Emended description of *Echinicola pacifica* Nedashkovskaya et al. 2006

The description is as given by Nedashkovskaya et al. (2006a) with the following modification. The genomic G+C content of the type strain is 43.8%.

#### Emended description of *Echinicola vietnamensis* Nedashkovskaya et al. 2007

The description is as given by Nedashkovskaya et al. (2007a) with the following modification. The genomic G+C content of the type strain is 44.8%.

#### Emended description of *Elizabethkingia anophelis* Kämpfer et al. 2011 emend. Kämpfer et al. 2015

The description is as given by Kämpfer et al. (2015) with the following addition. The genomic G+C content of the type strain is 35.4%.

#### Emended description of *Empedobacter brevis* (Holmes and Owen 1982) Vandamme et al. 1994 emend. Zhang et al. 2014

The description is as given by Zhang et al. (2014) with the following restriction. The genomic G+C content of the type strain is 32.8%.

#### Emended description of *Emticicia oligotrophica* Saha and Chakrabarti 2006

The description is as given by Saha and Chakrabarti (2006) with the following modification. The genomic G+C content of the type strain is 35.6%.

#### Emended description of *Flavihumibacter petaseus* Zhang et al. 2010 emend. Zhang et al. 2013

The description is as given by Zhang et al. (2013) with the following modification. The genomic G+C content of the type strain is 49.3%.

#### Emended description of *Flavihumibacter solisilvae* Lee et al. 2014

The description is as given by Lee et al. (2014) with the following modification. The genomic G+C content of the type strain is 47.0%.

#### Emended description of *Flavisolibacter ginsengisoli* Yoon and Im 2007

The description is as given by Yoon and Im (2007) with the following modification. The genomic G+C content of the type strain is 40.6%.

#### Emended description of *Flavisolibacter ginsengiterrae* Yoon and Im 2007

The description is as given by Yoon and Im (2007) with the following modification. The genomic G+C content of the type strain is 41.3%.

#### Emended description of *Flavobacterium antarcticum* Yi et al. 2005

The description is as given by Yi et al. (2005) with the following modification. The genomic G+C content of the type strain is 35.0%.

#### Emended description of *Flavobacterium aquatile* (Frankland and Frankland 1889) Bergey et al. 1923 emend. Sheu et al. 2013

The description is as given by Sheu et al. (2013) with the following addition. The genomic G+C content of the type strain is 32.2%.

#### Emended description of *Flavobacterium enshiense* Dong et al. 2013

The description is as given by Dong et al. (2013a) with the following modification. The genomic G+C content of the type strain is 37.7%.

#### Emended description of *Flavobacterium fryxellicola* Van Trappen et al. 2005

The description is as given by Van Trappen *et al*. (Van Trappen et al., 2005) with the following modification. The genomic G+C content of the type strain is 34.6%.

#### Emended description of *Flavobacterium hydatis* Bernardet et al., 1996

The description is as given by Bernardet et al. (1996) with the following modification. The genomic G+C content of the type strain is 32.7%.

#### Emended description of *Flavobacterium saliperosum* Wang et al. 2006 emend. Dong et al. 2013

The description is as given by Dong et al. (2013a) with the following modification. The genomic G+C content of the type strain is 39.6%.

#### Emended description of *Flavobacterium subsaxonicum* Ali et al. 2009 emend. Dong et al. 2013

The description is as given by Dong et al. (2013b) with the following modification. The genomic G+C content of the type strain is 41.6%.

#### Emended description of *Flavobacterium terrigena* Yoon et al. 2007 emend. Fujii et al. 2014

The description is as given by Fujii et al. (2014) with the following modification. The genomic G+C content of the type strain is 31.2%.

#### Emended description of *Flectobacillus major* (Gromov 1963) Larkin et al. 1977

The description is as given by Larkin et al. (1977) with the following modification. The genomic G+C content of the type strain is 37.8%.

#### Emended description of *Flexibacter roseolus* Lewin 1969

The description is as given by Lewin (1969) with the following restriction. The genomic G+C content of the type strain is 42.2%.

#### Emended description of *Formosa agariphila* Nedashkovskaya et al. 2006

The description is as given by Nedashkovskaya et al. (2006b) with the following modification. The genomic G+C content of the type strain is 33.5%.

#### Emended description of *Gelidibacter mesophilus* Macián et al. 2002

The description is as given by Macián et al. (2002) with the following restriction. The genomic G+C content of the type strain is 36.9%.

#### Emended description of *Gillisia limnaea* Van Trappen et al. 2004

The description is as given by Van Trappen et al. (2004) with the following restriction. The genomic G+C content of the type strain is 37.6%.

The genome sequence-derived G+C content was reported earlier (Riedel et al., 2012a) but no taxonomic consequences were drawn.

#### Emended description of *Haliscomenobacter hydrossis* Van Veen et al. 1973

The description is as given by van Veen et al. (1973) with the following addition. The genomic G+C content of the type strain is 47.1%.

The genome sequence-derived G+C content was reported earlier (Daligault et al., 2011) but no taxonomic consequences were drawn.

#### Emended description of *Hallella seregens* Moore and Moore 1994

The description is as given by Moore and Moore (1994) with the following modification. The genomic G+C content of the type strain is 56.0%.

#### Emended description of *Hymenobacter aerophilus* Buczolits et al. 2002

The description is as given by Buczolits et al. (2002) with the following restriction. The genomic G+C content of the type strain is 62.1%.

#### Emended description of *Hymenobacter norwichensis* Buczolits et al. 2006

The description is as given by Buczolits et al. (2006) with the following addition. The genomic G+C content of the type strain is 56.4%.

#### Emended description of *Hymenobacter roseosalivarius* Hirsch et al. 1999

The description is as given Hirsch et al. (1998) with the following restriction. The genomic G+C content of the type strain is 56.4%.

#### Emended description of *Imtechella halotolerans* Surendra et al. 2012

The description is as given by Surendra et al. (2012) with the following modification. The genomic G+C content of the type strain is 35.5%.

#### Emended description of *Indibacter alkaliphilus* Kumar et al. 2010

The description is as given by Kumar et al. (2010) with the following restriction. The genomic G+C content of the type strain is 39.7%.

#### Emended description of *Joostella marina* Quan et al. 2008

The description is as given by Quan et al. (2008) with the following modification. The genomic G+C content of the type strain is 33.6%.

The genome sequence-derived G+C content was reported earlier (Stackebrandt et al., 2013) but no taxonomic consequences were drawn.

#### Emended description of *Leadbetterella byssophila* Weon et al. 2005

The description is as given by Weon et al. (2005) with the following modification. The genomic G+C content of the type strain is 40.4%.

The genome sequence-derived G+C content was reported earlier (Abt et al., 2011b) but no taxonomic consequences were drawn.

#### Emended description of *Leeuwenhoekiella blandensis* Pinhassi et al. 2006

The description is as given by Pinhassi et al. (2006) with the following modification. The genomic G+C content of the type strain is 39.8%.

#### Emended description of *Lewinella persica* (Lewin 1970) Sly et al. 1998 emend. Khan et al. 2007

The description is as given by Khan et al. (2007) with the following modification. The genomic G+C content of the type strain is 51.6%.

#### Emended description of *Mariniradius saccharolyticus* Bhumika et al. 2013

The description is as given by Bhumika et al. (2013) with the following modification. The genomic G+C content of the type strain is 46.7%.

#### Emended description of *Microscilla marina* (Pringsheim 1951) Lewin 1969

The description is as given by Lewin (1969) with the following modification. The genomic G+C content of the type strain is 40.6%.

#### Emended description of *Mucilaginibacter paludis* Pankratov et al. 2007

The description is as given by Pankratov et al. (2007) with the following modification. The genomic G+C content of the type strain is 42.9%.

#### Emended description of *Muricauda lutaonensis* Arun et al. 2009

The description is as given by Arun et al. (2009) with the following restriction. The genomic G+C content of the type strain is 45.0%.

#### Emended description of *Myroides odoratus* (Stutzer 1929) Vancanneyt et al. 1996

The description is as given by Vancanneyt et al. (1996) with the following restriction. The genomic G+C content of the type strain is 35.8%.

#### Emended description of *Niabella aurantiaca* Kim et al. 2007

The description is as given by Kim et al. (2007) with the following modification. The genomic G+C content of the type strain is 48.6%.

#### Emended description of *Nonlabens marinus* Park et al. 2013 emend. Kwon et al. 2014

The description is as given by Kwon et al. (2014) with the following modification. The genomic G+C content of the type strain is 39.7%.

#### Emended description of *Odoribacter laneus* Nagai et al. 2010

The description is as given by Nagai et al. (2010) with the following modification. The genomic G+C content of the type strain is 40.5%.

#### Emended description of *Odoribacter splanchnicus* (Werner et al. 1975) Hardham et al. 2008

The description is as given by Hardham et al. (2008) with the following addition. The genomic G+C content of the type strain is 43.4%.

The genome sequence-derived G+C content was reported earlier (Göker et al., 2011) but no taxonomic consequences were drawn.

#### Emended description of *Opitutus terrae* Chin et al. 2001

The description is as given by Chin et al. (2001) with the following modification. The genomic G+C content of the type strain is 65.3%.

#### Emended description of *Owenweeksia hongkongensis* Lau et al. 2005 emend. Zhou et al. 2013

The description is as given by Zhou et al. (2013) with the following modification. The genomic G+C content of the type strain is 40.2%.

The genome sequence-derived G+C content was reported earlier (Riedel et al., 2012b) but no taxonomic consequences were drawn.

#### Emended description of *Parabacteroides distasonis* (Eggerth and Gagnon 1932) Sakamoto and Benno 2006

The description is as given by Sakamoto and Benno (2006) with the following modification. The genomic G+C content of the type strain is 45.1%.

#### Emended description of *Parabacteroides johnsonii* Sakamoto et al. 2007

The description is as given by Sakamoto et al. (2007) with the following modification. The genomic G+C content of the type strain is 45.2%.

#### Emended description of *Parabacteroides merdae* (Johnson et al. 1986) Sakamoto and Benno 2006

The description is as given by Sakamoto and Benno (2006) with the following modification. The genomic G+C content of the type strain is 45.3%.

#### Emended description of *Pedobacter glucosidilyticus* Luo et al. 2010 emend. Zhou et al. 2012

The description is as given by Zhou et al. (2012) with the following modification. The genomic G+C content of the type strain is 34.4%.

#### Emended description of *Pedobacter kyungheensis* Yang et al. 2015

The description is as given by Yang et al. (2012) with the following modification. The genomic G+C content of the type strain is 40.5%.

#### Emended description of *Pirellula staleyi* (Schlesner and Hirsch 1984) Schlesner and Hirsch 1987

The description is as given by Schlesner and Hirsch (1987) with the following addition. The genomic G+C content of the type strain is 57.5%.

The genome sequence-derived G+C content was reported earlier (Clum et al., 2009) but no taxonomic consequences were drawn.

#### Emended description of *Polaribacter franzmannii* Gosink et al. 1998

The description is as given by Gosink et al. (1998) with the following restriction. The genomic G+C content of the type strain is 32.5%.

#### Emended description of *Polaribacter irgensii* Gosink et al. 1998 emend. Kim et al. 2013

The description is as given by Kim et al. (2013) with the following modification. The genomic G+C content of the type strain is 34.5%.

#### Emended description of *Pontibacter actiniarum* Nedashkovskaya et al. 2005

The description is as given by Nedashkovskaya et al. (2005c) with the following modification. The genomic G+C content of the type strain is 53.1%.

#### Emended description of *Porphyromonas asaccharolytica* (Holdeman and Moore 1970) Shah and Collins 1988

The description is as given by Shah and Collins (1988) with the following restriction. The genomic G+C content of the type strain is 52.5%.

#### Emended description of *Porphyromonas bennonis* Summanen et al. 2009

The description is as given by Summanen et al. (2009) with the following modification. The genomic G+C content of the type strain is 56.3%.

#### Emended description of *Porphyromonas catoniae* (Moore and Moore 1994) Willems and Collins 1995

The description is as given by Willems and Collins (1995b) with the following modification. The genomic G+C content of the type strain is 51.0%.

#### Emended description of *Porphyromonas endodontalis* (Van Steenbergen et al. 1984) Shah and Collins 1988

The description is as given by Shah and Collins (1988) with the following restriction. The genomic G+C content of the type strain is 47.5%.

#### Emended description of *Porphyromonas gingivalis* (Coykendall et al. 1980) Shah and Collins 1988

The description is as given by Shah and Collins (1988) with the following modification. The genomic G+C content of the type strain is 48.4%.

#### Emended description of *Porphyromonas gulae* Fournier et al. 2001

The description is as given by Fournier et al. (2001) with the following modification. The genomic G+C content of the type strain is 48.6%.

#### Emended description of *Porphyromonas levii* (Johnson and Holdeman 1983) Shah et al. 1995

The description is as given by Shah et al. (1995) with the following restriction. The genomic G+C content of the type strain is 45.7%.

#### Emended description of *Porphyromonas somerae* Summanen et al. 2006

The description is as given by Summanen et al. (2005) with the following addition. The genomic G+C content of the type strain is 47.1%.

#### Emended description of *Prevotella albensis* Avgustin et al. 1997

The description is as given by Avgustin et al. (1997) with the following restriction. The genomic G+C content of the type strain is 41.2%.

#### Emended description of *Prevotella amnii* Lawson et al. 2008

The description is as given by Lawson et al. (2008) with the following addition. The genomic G+C content of the type strain is 36.6%.

#### Emended description of *Prevotella baroniae* Downes et al. 2005

The description is as given by Downes et al. (2005) with the following modification. The genomic G+C content of the type strain is 53.0%.

#### Emended description of *Prevotella brevis* (Bryant et al. 1958) Avgustin et al. 1997

The description is as given by Avgustin et al. (1997) with the following restriction. The genomic G+C content of the type strain is 48.7%.

#### Emended description of *Prevotella bryantii* Avgustin et al. 1997

The description is as given by Avgustin et al. (1997) with the following restriction. The genomic G+C content of the type strain is 39.1%.

#### Emended description of *Prevotella buccae* (Holdeman et al. 1982) Shah and Collins 1990

The description is as given by Shah and Collins (1990) with the following modification. The genomic G+C content of the type strain is 51.0%.

#### Emended description of *Prevotella copri* Hayashi et al. 2007

The description is as given by Hayashi et al. (2007b) with the following restriction. The genomic G+C content of the type strain is 44.8%.

#### Emended description of *Prevotella corporis* (Johnson and Holdeman 1983) Shah and Collins 1990

The description is as given by Shah and Collins (1990) with the following restriction. The genomic G+C content of the type strain is 44.1%.

#### Emended description of *Prevotella dentalis* (Haapasalo et al. 1986) Willems and Collins 1995

The description is as given by Willems and Collins (1995a) with the following restriction. The genomic G+C content of the type strain is 55.9%.

#### Emended description of *Prevotella denticola* (Shah and Collins 1982) Shah and Collins 1990 emend. Wu et al. 1992

The description is as given by Wu et al. (1992) with the following restriction. The genomic G+C content of the type strain is 50.1%.

#### Emended description of *Prevotella histicola* Downes et al. 2008

The description is as given by Downes et al. (2008) with the following modification. The genomic G+C content of the type strain is 41.2%.

#### Emended description of *Prevotella intermedia* (Holdeman and Moore 1970) Shah and Collins 1990

The description is as given by Shah and Collins (1990) with the following restriction. The genomic G+C content of the type strain is 43.3%.

#### Emended description of *Prevotella loescheii* (Holdeman and Johnson 1982) Shah and Collins 1990 emend. Wu et al. 1992

The description is as given by Wu et al. (1992) with the following restriction. The genomic G+C content of the type strain is 46.6%.

#### Emended description of *Prevotella marshii* Downes et al. 2005

The description is as given by Downes et al. (2005) with the following modification. The genomic G+C content of the type strain is 47.5%.

#### Emended description of *Prevotella melaninogenica* (Oliver and Wherry 1921) Wu et al. 1992

The description is as given by Wu et al. (1992) with the following addition. The genomic G+C content of the type strain is 41.0%.

#### Emended description of *Prevotella multisaccharivorax* Sakamoto et al. 2005

The description is as given by Sakamoto et al. (2005) with the following modification. The genomic G+C content of the type strain is 48.3%.

The genome sequence-derived G+C content was reported earlier (Pati et al., 2011b) but no taxonomic consequences were drawn.

#### Emended description of *Prevotella nanceiensis* Alauzet et al. 2007

The description is as given by Alauzet et al. (2007) with the following modification. The genomic G+C content of the type strain is 38.4%.

#### Emended description of *Prevotella nigrescens* Shah and Gharbia 1992

The description is as given by Shah and Gharbia (1992) with the following restriction. The genomic G+C content of the type strain is 42.6%.

#### Emended description of *Prevotella oralis* (Loesche et al. 1964) Shah and Collins 1990

The description is as given by Shah and Collins (1990) with the following addition. The genomic G+C content of the type strain is 44.5%.

#### Emended description of *Prevotella oris* (Holdeman et al. 1982) Shah and Collins 1990

The description is as given by Shah and Collins (1990) with the following restriction. The genomic G+C content of the type strain is 43.8%.

#### Emended description of *Prevotella pallens* Könönen et al., 1998

The description is as given by Könönen et al. (1998) with the following addition. The genomic G+C content of the type strain is 37.4%.

#### Emended description of *Prevotella paludivivens* Ueki et al. 2007

The description is as given by Ueki et al. (2007) with the following modification. The genomic G+C content of the type strain is 37.3%.

#### Emended description of *Prevotella timonensis* Glazunova et al. 2007

The description is as given by Glazunova et al. (2007) with the following addition. The genomic G+C content of the type strain is 42.4%.

#### Emended description of *Prosthecochloris aestuarii* Gorlenko 1970 emend. Imhoff 2003

The description is as given by Imhoff (2003) with the following addition. The genomic G+C content of the type strain is 50.1%.

#### Emended description of *Proteiniphilum acetatigenes* Chen and Dong 2005

The description is as given by Chen and Dong (2005) with the following restriction. The genomic G+C content of the type strain is 43.1%.

#### Emended description of *Pseudopedobacter saltans* (Steyn et al. 1998) Cao et al. 2014

The description is as given by Cao et al. (2014) with the following restriction. The genomic G+C content of the type strain is 36.6%.

#### Emended description of *Pseudosphingobacterium domesticum* Vaz-Moreira et al. 2007

The description is as given by Vaz-Moreira et al. (2007) with the following modification. The genomic G+C content of the type strain is 38.9%.

#### Emended description of *Psychroflexus gondwanensis* (Dobson et al. 1993) Bowman et al. 1999

The description is as given by Bowman et al. (1998) with the following modification. The genomic G+C content of the type strain is 35.8%.

#### Emended description of *Psychroflexus torquis* Bowman et al. 1999

The description is as given by Bowman et al. (1998) with the following modification. The genomic G+C content of the type strain is 34.5%.

#### Emended description of *Psychroflexus tropicus* Donachie et al. 2004

The description is as given by Donachie et al. (2004) with the following restriction. The genomic G+C content of the type strain is 36.5%.

#### Emended description of *Psychroserpens burtonensis* Bowman et al. 1997

The description is as given by Bowman et al. (1997) with the following restriction. The genomic G+C content of the type strain is 33.4%.

#### Emended description of *Rhodonellum psychrophilum* Schmidt et al. 2006

The description is as given by Schmidt et al. (2006) with the following modification. The genomic G+C content of the type strain is 41.8%.

#### Emended description of *Rikenella microfusus* (Kaneuchi and Mitsuoka 1978) Collins et al. 1985

The description is as given by Collins et al. (1985) with the following restriction. The genomic G+C content of the type strain is 57.0%.

#### Emended description of *Robiginitalea biformata* Cho and Giovannoni 2004

The description is as given by Cho and Giovannoni (2004) with the following modification. The genomic G+C content of the type strain is 55.3%.

#### Emended description of *Roseivirga echinicomitans* Nedashkovskaya et al. 2005

The description is as given by Nedashkovskaya et al. (2005b) with the following modification. The genomic G+C content of the type strain is 40.0%.

#### Emended description of *Roseivirga ehrenbergii* Nedashkovskaya et al. 2005 emend. Nedashkovskaya et al. 2008

The description is as given by Nedashkovskaya et al. (2008) with the following modification. The genomic G+C content of the type strain is 39.3%.

#### Emended description of *Roseivirga spongicola* Lau et al. 2006

The description is as given by Lau et al. (2006) with the following modification. The genomic G+C content of the type strain is 40.2%.

#### Emended description of *Runella limosa* Ryu et al. 2006

The description is as given by Ryu et al. (2006) with the following modification. The genomic G+C content of the type strain is 44.3%.

#### Emended description of *Runella slithyformis* Larkin and Williams 1978

The description is as given by Larkin and Williams (1978) with the following modification. The genomic G+C content of the type strain is 46.6%.

The genome sequence-derived G+C content was reported earlier (Copeland et al., 2012) but no taxonomic consequences were drawn.

#### Emended description of *Runella zeae* Chelius et al. 2002

The description is as given by Chelius et al. (2002) with the following modification. The genomic G+C content of the type strain is 42.1%.

#### Emended description of *Salegentibacter flavus* Ivanova et al. 2006

The description is as given by Ivanova et al. (2006) with the following modification. The genomic G+C content of the type strain is 39.8%.

#### Emended description of *Salinimicrobium terrae* Chen et al. 2008

The description is as given by Chen et al. (2008) with the following modification. The genomic G+C content of the type strain is 40.4%.

#### Emended description of *Segetibacter koreensis* An et al. 2007

The description is as given by An et al. (2007) with the following modification. The genomic G+C content of the type strain is 37.4%.

#### Emended description of *Sphingobacterium multivorum* (Holmes et al. 1981) Yabuuchi et al. 1983

The description is as given by Yabuuchi et al. (1983) with the following modification. The genomic G+C content of the type strain is 39.9%.

#### Emended description of *Sphingobacterium thalpophilum* (Holmes et al. 1983) Takeuchi and Yokota 1993

The description is as given by Takeuchi and Yokota (1992) with the following modification. The genomic G+C content of the type strain is 43.6%.

#### Emended description of *Spirosoma linguale* Migula 1894

The description is as given by Migula (1894) with the following restriction. The genomic G+C content of the type strain is 50.1%.

The genome sequence-derived G+C content was reported earlier (Lail et al., 2010) but no taxonomic consequences were drawn.

#### Emended description of *Spirosoma panaciterrae* Ten et al. 2009

The description is as given by Ten et al. (2009) with the following modification. The genomic G+C content of the type strain is 48.9%.

#### Emended description of *Spirosoma spitsbergense* Finster et al. 2009

The description is as given by Finster et al. (2009) with the following modification. The genomic G+C content of the type strain is 50.4%.

#### Emended description of *Sporocytophaga myxococcoides* (Krzemieniewska 1933) Stanier 1940

The description is as given by Stanier (1940) with the following addition. The genomic G+C content of the type strain is 36.2%.

#### Emended description of *Tamlana sedimentorum* Romanenko et al. 2014

The description is as given by Romanenko et al. (2014) with the following modification. The genomic G+C content of the type strain is 32.9%.

#### Emended description of *Terrimicrobium sacchariphilum* Qiu et al. 2014

The description is as given by Qiu et al. (2014) with the following modification. The genomic G+C content of the type strain is 60.2%.

#### Emended description of *Thermonema rossianum* Nobre et al. 1997

The description is as given by Nobre et al. (Tenreiro et al., 1997) with the following modification. The genomic G+C content of the type strain is 48.8%.

#### Emended description of *Weeksella virosa* Holmes et al. 1987 emend. Zhang et al. 2014

The description is as given by Zhang et al. (2014) with the following modification. The genomic G+C content of the type strain is 35.9%.

The genome sequence-derived G+C content was reported earlier (Lang et al., 2011) but no taxonomic consequences were drawn.

#### Emended description of *Winogradskyella psychrotolerans* Begum et al. 2013

The description is as given by Begum et al. (2013) with the following restriction. The genomic G+C content of the type strain is 33.5%.

## Author contributions

HK prepared genomic DNA. TW sequenced the genomes. MH, NI, NK, and SM annotated the genomes. JM and MG phylogenetically analyzed the data. MGL and MG collected the G+C content information. RH, MGL, and MG collected the phenotypic information. RH, JM, MGL, and MG interpreted the results. All authors read and approved the final manuscript.

## Funding

This work was performed under the auspices of the US Department of Energy's Office of Science, Biological and Environmental Research Program, and by the University of California, Lawrence Berkeley National Laboratory under contract No. DE-AC02-05CH11231. RH was supported by the German Bundesministerium für Ernährung und Landwirtschaft, grant No. 22016812 for Brian J. Tindall.

### Conflict of interest statement

The authors declare that the research was conducted in the absence of any commercial or financial relationships that could be construed as a potential conflict of interest.
