# Corrigendum: Genome-Based Taxonomic Classification of *Bacteroidetes*

**DOI:** 10.3389/fmicb.2018.00304

**Published:** 2018-02-21

**Authors:** Richard L. Hahnke, Jan P. Meier-Kolthoff, Marina García-López, Supratim Mukherjee, Marcel Huntemann, Natalia N. Ivanova, Tanja Woyke, Nikos C. Kyrpides, Hans-Peter Klenk, Markus Göker

**Affiliations:** ^1^Department of Microorganisms, Leibniz Institute DSMZ – German Collection of Microorganisms and Cell Cultures, Braunschweig, Germany; ^2^Department of Energy Joint Genome Institute (DOE JGI), Walnut Creek, CA, United States; ^3^Department of Biological Sciences, Faculty of Science, King Abdulaziz University, Jeddah, Saudi Arabia; ^4^School of Biology, Newcastle University, Newcastle upon Tyne, United Kingdom

**Keywords:** G+C content, genome BLAST distance phylogeny, gliding motility, gut microbiome, marine microbiology, one thousand microbial genomes project, phylogenetic classification, Bacteroidaeota-Rhodothermaeota-Balneolaeota-Chlorobaeota superphylum

In the Taxonomic Consequences section of the Discussion chapter of the original article the type of the newly proposed class *Saprospiria* was incorrectly indicated (and in conflict with the etymology paragraph) within the description of the class. A correction has accordingly been made to Discussion, Taxonomic Consequences, Description of *Saprospiria*, class. nov.:

**Description of *Saprospiria*, class. nov**.

Sa.pros.pi'ri.a (N.L. fem. n. *Saprospira*, type genus of the type order of the class, -*ia* ending to denote a class; N.L. fem. pl. n. *Saprospiria*, the class of the order *Saprospirales*).

The description is the same as for the order *Saprospirales*.

This class is described on the basis of 16S rRNA gene and whole-genome phylogenetic analysis. The type and currently sole order of the class is *Saprospirales*.

The authors apologize for this error and state that this does not change the scientific conclusions of the original article in any way.

The original article has been updated.

## Conflict of interest statement

The authors declare that the research was conducted in the absence of any commercial or financial relationships that could be construed as a potential conflict of interest.

