# Supplementary material for: Genome-Based Taxonomic Classification of Bacteroidetes
Source: Front Microbiol. 2016 Dec 20;7:2003. doi: 10.3389/fmicb.2016.02003 (PMC5167729; doi:10.3389/fmicb.2016.02003)
Supplement: Supplementary file 2 [file DataSheet2.pdf]

Supplementary file S2

Changes to the LTP classification

Rhodonellum was listed in the LTP s123 classification (Yarza et al., 2008), which is derived from LPSN (Euzéby, 1997), as belonging to *Cytophagaceae*. In the description of the genus *Rhodonellum* (Schmidt et al., 2006) it was placed within the family *"Flexibacteraceae"*. According to a later study (Ying et al., 2006), its most closely related genera are *Algoriphagus*, *Aquiflexum*, *Belliella*, *Chimaereicella, Cyclobacterium* and *Hongiella*; among those, *Chimaereicella* and *Hongiella* have been reclassified into *Algoriphagus* (Nedashkovskaya et al., 2007). Accordingly, *Algoriphagus*, *Aquiflexum*, *Belliella*, *Cyclobacterium*, *Echinicola* and *Rhodonellum* have been assigned to *Cyclobacteriaceae* (Nedashkovskaya and Ludwig, 2011; Euzéby, 2012).

An affiliation to a family for *Saccharicrinis* was missing in the LTP s123 classification but provided in the literature (Yang et al., 2014). *Thermonema* was listed in the LTP s123 classification as belonging to *Flammeovirgaceae*. But according to the literature (Nedashkovskaya and Ludwig, 2011a) *Flammeovirgaceae* comprise *Flexithrix*, *Fabibacter*, *Flammeovirga*, *Persicobacter*, *Reichenbachiella* and *Roseivirga*, whereas *Thermonema* is classified as incertae sedis (The editorial board, 2011).

The closely related genera *Aliifodinibius*, *Balneola, Fodinibius*, and *Gracilimonas* are listed in LTP/LPSN under the family *Chitinophagaceae*. The family *Chitinophagaceae* originally comprised the genera *Chitinophaga* and *Terrimonas* (Ii, 2010). The new genus *Balneola* was described as belonging to the family *Crenotrichaceae* (Urios et al., 2006). However, the genus *Crenothrix* was later on assigned to *Methylococcales* (Stoecker et al., 2006). Thus, all other *Crenotrichaceae* genera were then considered as *Sphingobacteriales* incertae sedis (Urios et al., 2008). Much like *Balneola*, the genera *Fodinibius* (Wang et al., 2012), *Aliifodinibius* (Wang et al., 2013) and *Gracilimonas* (Choi et al., 2009) were classified as *Sphingobacteriales* incertae sedis (class *Cytophagia*) (Nakagawa, 2011a; Euzéby, 2012). Therefore it remains unclear why these genera have been placed into the family *Chitinophagaceae* by LTP/LPSN.

A couple of species were not included in our comprehensive *Bacteroidetes* 16S rRNA gene tree because in contrast to the LTP/LPSN classification they are not taxonomically classified in *Bacteroidetes* any more. *Anaerorhabdus* with its sole species *A. furcosa* is listed as belonging to *Bacteroidaceae*, whereas a recent monography (Rosenberg et al., 2013) assigns it to *Erysipelotrichaceae* (*Firmicutes*) because if its phylogenetic placement in 16S rRNA gene trees. *Acetomicrobium faecale* has been reclassified into the genus *Caldicoprobacter* (*Firmicutes*) as *C. faecalis* (Ben

Hania et al., 2015). The remaining (and type) species in *Acetomicrobium*, *A. flavidum*, is placed within *Synergistetes* in 16S rRNA gene trees (Yarza et al., 2008), thus *Acetomicrobium* should not be classified as within *Bacteroidaceae* as in the LTP/LPSN classification but outside the *Bacteroidetes*. *Bacteroides cellulosolvens* should not separately be listed in the LTP/LPSN classification because it is a homotypic synonym of *Pseudobacteroides cellulosolvens* (*Firmicutes*) (Horino et al., 2014). Similarly, *Flexibacter aurantiacus* is a homotypic synonym of *Flavobacterium johnsoniae* (Bernardet et al., 1996).

Flavobacterium oceanosedimentum (*Flavobacteriaceae*) has been reclassified as *Curtobacterium oceanosedimentum* (*Actinobacteria*) (Kim et al., 2009) but this name has not been regarded as validly published. For *Bacteroides coagulans*, *B. galacturonicus* and *B. pectinophilus* new combinations have not yet been proposed but phylogenetically they are not only placed outside *Bacteroides* but even outside *Bacteroidetes* (Yarza et al., 2008). For this reason, they were not included in our comprehensive *Bacteroidetes* 16S rRNA gene tree either.

Prolixibacteraceae comprise the genera *Prolixibacter*, *Sunxiuqinia* and *Mangrovibacterium* (Huang et al., 2014). *Prolixibacteraceae* are an earlier heterotypic synonym of *Draconibacteriaceae* (Du et al., 2014) and thus comprise additionally the genera *Draconibacterium*, *Mariniphaga* and *Meniscus* (Iino et al., 2014). The genus *Marinifilum* belongs to the family *Marinifilaceae* (order *Bacteroidales*) (Iino et al., 2014).

A couple of sequences of interest were missing from the LTP s123 release, including the type species of the genus *Thermonema*, *T. lapsum* (HE582775) (Hudson et al., 1989); *Saprospira grandis* (M58795); and *Epilithonimonas psychrotolerans* (DQ173014). Moreover, AB078062 is incorrect a sequence of the type strain of *Flexibacter roseolus*; AB078063 should be chosen instead (Nakagawa et al., 2002).

Ignavibacterium (family *Ignavibacteriaceae*, order *Ignavibacteriales*, class *Ignavibacteria*) was first placed in the phylum *Chlorobi* (Iino et al., 2010). However, with the description of the new genus *Melioribacter* within the new family *Melioribacteraceae*, the class *Ignavibacteria* was placed into the new phylum *Ignavibacteriae* (Oren and Garrity, 2016; Podosokorskaya et al., 2013).

The new families *Catalimonadaceae* (comprising the genera *Catalinimonas*, '*Porifericola*' and '*Tunicatimonas*') and *Mooreiaceae* (comprising the genus *Mooreia*) were recently suggested (Choi et al., 2013). This resulted into a split of the family *Flammeovirgaceae* into the core *Flammeovirgaceae* (comprising the genera *Flammeovirga*, *Perexilibacter*, *Limibacter*, *Sediminitomix*, *Rapidithrix*, *Flexithrix*, *Persicobacter* and *Aureibacter*) and a taxonomically unresolved branch (comprising the

genera *Cesiribacter*, *Marivirga*, *Roseivirga*, *Fabibacter*, *Reichenbachia*, *Fulvivirga* and *Marinoscillum*) (Choi et al., 2013).

A variety of changes to the classification were made in a recent study (Munoz et al., 2016) using 16S rRNA gene and MLSA phylogenies (for which no branch support was presented). *Butyricimonas* and *Odoribacter* were placed in a family of their own, *Odoribacteraceae*, whereas *Crocinitomix* and *Fluviicola* were placed in the new family *Crocinitomicaceae* together with *Brumimicrobium Lishizhenia*, *Salinirepens* and *Wandonia*. *Adhaeribacter*, *Hymenobacter*, *Nibribacter, Pontibacter* and *Rufibacter* were placed in the new family *Hymenobacteraceae* but *Aureibacter*, *Fulvitalea* and *Persicobacter* in *Persicobacteraceae*. For *Thermonema* the new family *Thermonemataceae* was introduced, and for the *Chitinophagaceae* and *Saprospiraceae* the new order *Chitinophagales* in the new class *Chitinophagia*. The genera *Persicobacter*, *Aureibacter* and *Fulvitalea* were moved into the new family *Persicobacteraceae*. Finally, the new phylum *Rhodothermaeota* was established, comprising the classes *Balneolia* and *Rhodothermia* with the sole orders *Balneolales* and *Rhodothermales*, respectively. *Balneolaceae* were introduced to harbor *Aliifodinibius*, *Balneola, Fodinibius* and *Gracilimonas*, *Salinibacteraceae* to comprise *Salinibacter*, *Salisaeta* and *Salinivenus* (a genus newly introduced for *Salinibacter iranicus* and *S. luteus*), and *Rubricoccaceae* to contain *Rubricoccus* and *Rubrivirga*, thus restricting *Rhodothermaceae* to *Rhodothermus*.

Other taxonomic notes

The genus *Flexithrix* is listed in LPSN in the family *Flammeovirgaceae*. This is consistent with Bergey's Manual of Systematic Bacteriology (Nedashkovskaya and Ludwig, 2011a). The family *Flammeovirgaceae* (Nedashkovskaya and Ludwig, 2011a) comprises the genera *Flexithrix* as well as *Fabibacter*, *Flammeovirga*, *Persicobacter*, *Reichenbachiella* and *Roseivirga* (Nedashkovskaya and Ludwig, 2011a). *Flexithrix dorotheae* Lewin 1970 (Approved Lists 1980) is an earlier heterotypic synonym of *Flexibacter aggregans* (Lewin 1969) Leadbetter 1974 (Approved Lists 1980) (Hosoya and Yokota, 2007). *Flexithrix dorotheae* is the type species of the genus and the only type train in the genus. See notes on "Flexibacter" below.

The genus *Marivirga* is listed in LPSN in the family *Flammeovirgaceae*. The family *Flammeovirgaceae* comprises the genera *Flexithrix* as well as *Fabibacter*, *Flammeovirga*, *Persicobacter*, *Reichenbachiella* and *Roseivirga* (Nedashkovskaya and Ludwig, 2011a). Based on the results of Nakagawa et al. (Nakagawa et al., 2002) and a polyphasic approach by Nedashkovskaya et al. (Nedashkovskaya et al., 2010), the genus *Marivirga* was proposed comprising *Marivirga tractuosa* (Flexibacter tractuosus

(Lewin 1969) Leadbetter 1974) and *Marivirga sericea* (“*Microscilla sericea*” Lewin 1969) (Nedashkovskaya and Ludwig, 2011a).

Owenweeksia hongkongensis (Lau et al., 2005; Zhou et al., 2013) is listed in LPSN under the family *Cryomorphaceae*. This is consistent with Bergey’s Manual of Systematic Bacteriology (Nedashkovskaya and Ludwig, 2011a). The family *Cryomorphaceae* (Bowman, 2011) comprises the genera *Brumimicrobium*, *Crocinitomix*, *Cryomorpha*, *Fluviicola*, *Lishizhenia*, *Owenweeksia* (Bowman, 2011; Lee et al., 2010), as well as the genera *Phaeocystidibacter* (Zhou et al., 2013), *Salinirepens* (Muramatsu et al., 2012), *Luteibaculum* (Shahina et al., 2013) and *Wandonia* (Lee et al., 2010; Muramatsu et al., 2012.

The genus *Flexibacter* is listed in LPSN in the family *Cytophagaceae*. This is consistent with Bergey’s Manual of Systematic Bacteriology (Nedashkovskaya and Ludwig, 2011a). The family *Cytophagaceae* comprises the genera *Flexibacter* as well as *Adhaeribacter*, *Arcicella*, *Cytophaga*, *Dyadobacter*, *Effluviibacter*, *Emticicia*, *Flectobacillus*, *Hymenobacter*, *Larkinella*, *Leadbetterella*, *Meniscus*, *Microscilla*, *Pontibacter*, *Runella*, *Spirosoma* and *Sporocytophaga* (Nakagawa, 2011b).

Recently the family *Rhodothermaceae* (Ludwig W, Euzeby J, 2011; Euzéby, 2012) was validly named (incertae sedis, class *Cytophagia*), comprising the genera *Rhodothermus* and *Salinibacter* (Oren and Garrity, 2015), as well as the genera *Rubricoccus* (Park et al., 2011), *Rubrivirga* (Park et al., 2013) and *Salisaeta* (Vaisman and Oren, 2009).

References

Bernardet, J.-F., Segers, P., Vancanneyt, M., Berthe, F., Kersters, K., and Vandamme, P. (1996). Cutting a gordian knot: emended classification and description of the genus *Flavobacterium*, emended description of the family *Flavobacteriaceae*, and proposal of *Flavobacterium hydatis* nom. nov. (Basonymasonym, *Cytophaga*. *Int. J. Syst. Bacteriol.* 46, 128–148. doi:10.1099/00207713-46-1-128.

Bowman, J. (2011). “Family III. Cryomorphaceae Bowman, Nichols and Gibson 2003, 1353VP.,” in *Bergey’s Manual of Systematic Bacteriology, 2nd edition, vol. 4. The Bacteroidetes, Spirochaetes, Tenericutes (Mollicutes), Acidobacteria, Fibrobacteres, Fusobacteria, Dictyoglomi, Gemmatimonadetes, Lentisphaerae, Verrucomicrobia, Chlamydiae, and Planctom*, ed. W. W. Krieg NR, Staley JT, Brown DR, Hedlund BP, Paster BJ, Ward NL, Ludwig W (New York: Springer), 322.

Choi, D. H., Zhang, G. I., Noh, J. H., Kim, W. S., and Cho, B. C. (2009). Gracilimonas tropica gen. nov., sp. nov., isolated from a Synechococcus culture. *Int. J. Syst. Evol. Microbiol.* 59, 1167–1172. doi:10.1099/ijs.0.005512-0.

Choi, E. J., Beatty, D. S., Paul, L. A., Fenical, W., and Jensen, P. R. (2013). Mooreia alkaloidigena gen. nov., sp. nov. and Catalinimonas alkaloidigena gen. nov., sp. nov., alkaloid-producing

marine bacteria in the proposed families Mooreiaceae fam. nov. and Catalimonadaceae fam. nov. in the phylum Bacteroidetes. *Int. J. Syst. Evol. Microbiol.* 63, 1219–1228. doi:10.1099/ijs.0.043752-0.

Du, Z. J., Wang, Y., Dunlap, C., Rooney, A. P., and Chen, G. J. (2014). *Draconibacterium orientale* gen. nov., sp. nov., isolated from two distinct marine environments, and proposal of *Draconibacteriaceae* fam. nov. *Int. J. Syst. Evol. Microbiol.* 64, 1690–1696. doi:10.1099/ijs.0.056812-0.

Euzéby, J. P. (1997). List of Bacterial Names with Standing in Nomenclature: a folder available on the Internet. *Int. J. Syst. Bacteriol.* 47, 590–2. Available at: http://www.ncbi.nlm.nih.gov/pubmed/9103655.

Ben Hania, W., Fardeau, M.-L., Cayol, J.-L., Bouanane-Darenfed, A., and Ollivier, B. (2015). Reclassification of Acetomicrobium faecale as Caldicoprobacter faecalis comb. nov. *Int. J. Syst. Evol. Microbiol.* 65, 3286–3288. doi:10.1099/ijsem.0.000409.

Horino, H., Fujita, T., and Tonouchi, A. (2014). Description of Anaerobacterium chartisolvens gen. nov., sp. nov., an obligately anaerobic bacterium from Clostridium rRNA cluster III isolated from soil of a Japanese rice field, and reclassification of Bacteroides cellulosolvens Murray et al. 1984 as Pse. *Int. J. Syst. Evol. Microbiol.* 64, 1296–1303. doi:10.1099/ijs.0.059378-0.

Hosoya, S., and Yokota, A. (2007). Reclassification of Flexibacter aggregans (Lewin 1969) Leadbetter 1974 as a later heterotypic synonym of Flexithrix dorotheae Lewin 1970. *Int. J. Syst. Evol. Microbiol.* 57, 1086–1088. doi:10.1099/ijs.0.64798-0.

Huang, X. F., Liu, Y. J., Dong, J. De, Qu, L. Y., Zhang, Y. Y., Wang, F. Z., et al. (2014). *Mangrovibacterium diazotrophicum* gen. nov., sp. nov., a nitrogen-fixing bacterium isolated from a mangrove sediment, and proposal of *Prolixibacteraceae* fam. nov. *Int. J. Syst. Evol. Microbiol.* 64, 875–881. doi:10.1099/ijs.0.052779-0.

Hudson, J. A., Schofield, K. M., Morgan, H. W., and Daniel, R. M. (1989). Thermonema lapsum gen. nov., sp. nov., a thermophilic gliding bacterium. *Int. J. Syst. Bacteriol.* 39, 485–487. Available at: http://www.scopus.com/inward/record.url?eid=2-s2.0-0024451276&partnerID=40&md5=a1d5a94d53f6c9ba14bd32b9eff291dd.

Ii, F. (2010). Family II. Chitinophagaceae fam. nov. Kämpfer, Lodders and Falsen 2010. *Bergey's Man. Syst. Bacteriol. 2nd Ed. vol. 4. Bacteroidetes, Spirochaetes, Tenericutes (Mollicutes), Acidobacteria, Fibrobacteres, Fusobacteria, Dictyoglomi, Gemmatimonadetes, Lentisphaerae, Verrucomicrobia, Chlamydiae, Planctom*, 2462–2466.

Iino, T., Mori, K., Itoh, T., Kudo, T., Suzuki, K. I., and Ohkuma, M. (2014). Description of *Mariniphaga anaerophila* gen. nov., sp. nov., A facultatively aerobic marine bacterium isolated from tidal flat sediment, reclassification of the *Draconibacteriaceae* as a later heterotypic synonym of the <i>Prolixibacteraceae</. *Int. J. Syst. Evol. Microbiol.* 64, 3660–3667. doi:10.1099/ijs.0.066274-0.

Iino, T., Mori, K., Uchino, Y., Nakagawa, T., Harayama, S., and Suzuki, K. I. (2010). *Ignavibacterium album* gen. nov., sp. nov., a moderately thermophilic anaerobic bacterium

isolated from microbial mats at a terrestrial hot spring and proposal of *Ignavibacteria* classis nov., for a novel lineage at the periphery of green sulf. *Int. J. Syst. Evol. Microbiol.* 60, 1376–1382. doi:10.1099/ijs.0.012484-0.

Kim, B. K., Kim, O. S., Moon, E. Y., and Chun, J. (2009). Proposal to transfer Flavobacterium oceanosedimentum Carty and Litchfield 1978 to the genus Curtobacterium as Curtobacterium oceanosedimentum comb. nov. *FEMS Microbiol. Lett.* 296, 137–141. doi:10.1111/j.1574-6968.2009.01628.x.

Lau, K. W. K., Ng, C. Y. M., Ren, J., Lau, S. C. L., Qian, P. Y., Wong, P. K., et al. (2005). *Owenweeksia hongkongensis* gen. nov., sp. nov., a novel marine bacterium of the phylum "*Bacteroidetes*." *Int. J. Syst. Evol. Microbiol.* 55, 1051–1057. doi:10.1099/ijs.0.63155-0.

Lee, D. H., Choi, E. K., Moon, S. R., Ahn, S., Lee, Y. S., Jung, J. S., et al. (2010). Wandonia haliotis gen. nov., sp. nov., a marine bacterium of the family Cryomorphaceae, phylum Bacteroidetes. *Int. J. Syst. Evol. Microbiol.* 60, 510–514. doi:10.1099/ijs.0.012674-0.

Ludwig, W., Euzeby, J., and Whitman, W. B. (2011). "Family I. Rhodothermaaceae fam. Nov. Kämpfer, Lodders and Falsen 2010," in *Bergey's Manual of Systematic Bacteriology, 2nd edition, vol. 4. The Bacteroidetes, Spirochaetes, Tenericutes (Mollicutes), Acidobacteria, Fibrobacteres, Fusobacteria, Dictyoglomi, Gemmatimonadetes, Lentisphaerae, Verrucomicrobia, Chlamydiae, and Planctom*, eds. N. R. Krieg, J. T. Staley, D. R. Brown, B. P. Hedlund, B. J. Paster, N. L. Ward, et al. (New York: Springer), 457.

Munoz, R., Rosselló-Móra, R., and Amann, R. (2016). Revised phylogeny of *Bacteroidetes* and proposal of sixteen new taxa and two new combinations including *Rhodothermaeota* phyl. nov. *Syst. Appl. Microbiol.* doi:10.1016/j.syapm.2016.04.004.

Muramatsu, Y., Takahashi, M., Kamakura, Y., Suzuki, K. ichiro, and Nakagawa, Y. (2012). Salinirepens amamiensis gen. nov., sp. nov., a member of the family cryomorphaceae isolated from seawater, and emended descriptions of the genera fluviicola and Wandonia. *Int. J. Syst. Evol. Microbiol.* 62, 2235–2240. doi:10.1099/ijs.0.032029-0.

Nakagawa, Y. (2011a). "Class IV. Cytophagia class. nov.," in *Bergey's Manual of Systematic Bacteriology, 2nd edition, vol. 4. The Bacteroidetes, Spirochaetes, Tenericutes (Mollicutes), Acidobacteria, Fibrobacteres, Fusobacteria, Dictyoglomi, Gemmatimonadetes, Lentisphaerae, Verrucomicrobia, Chlamydiae, and Planctom*, eds. N. R. Krieg, J. T. Staley, D. R. Brown, B. P. Hedlund, B. J. Paster, N. L. Ward, et al. (New York: Springer), 370.

Nakagawa, Y. (2011b). "Family I. Cytophagaceae Stanier 1940, 630AL," in *Bergey's Manual of Systematic Bacteriology, 2nd edition, vol. 4. The Bacteroidetes, Spirochaetes, Tenericutes (Mollicutes), Acidobacteria, Fibrobacteres, Fusobacteria, Dictyoglomi, Gemmatimonadetes, Lentisphaerae, Verrucomicrobia, Chlamydiae, and Planctom*, eds. N. R. Krieg, J. T. Staley, D. R. Brown, B. P. Hedlund, B. J. Paster, N. L. Ward, et al. (New York: Springer), 371.

Nakagawa, Y., Sakane, T., Suzuki, M., and Hatano, K. (2002). Phylogenetic structure of the genera *Flexibacter*, *Flexithrix*, and *Microscilla* deduced from 16S rRNA sequence analysis. *J. Gen. Appl. Microbiol.* 48, 155–165. doi:10.2323/jgam.48.155.

Nedashkovskaya, O. I. I., and Ludwig, W. (2011a). “Family III. *Flammeovirgaceae* fam. nov.,” in *Bergey’s Manual of Systematic Bacteriology, 2nd edition, vol. 4. The Bacteroidetes, Spirochaetes, Tenericutes (Mollicutes), Acidobacteria, Fibrobacteres, Fusobacteria, Dictyoglomi, Gemmatimonadetes, Lentisphaerae, Verrucomicrobia, Chlamydiae, and Planctom*, eds. N. R. Krieg, J. T. Staley, D. R. Brown, B. P. Hedlund, B. J. Paster, N. L. Ward, et al. (New York: Springer), 442.

Nedashkovskaya, O. I., Kim, S. B., Kwon, K. K., Shin, D. S., Luo, X., Kim, S. J., et al. (2007). Proposal of *Algoriphagus vanfongensis* sp. nov., transfer of members of the genera *Hongiella* Yi and Chun 2004 emend. Nedashkovskaya *et al.* 2004 and *Chimaereicella* Tiago *et al.* 2006 to the genus *Algoriphagus*, and em. *Int. J. Syst. Evol. Microbiol.* 57, 1988–1994. doi:10.1099/ijs.0.65073-0.

Nedashkovskaya, O. I., and Ludwig, W. (2011b). “Family II. *Cyclobacteriaceae* fam. nov.,” in *Bergey’s Manual of Systematic Bacteriology, 2nd edition, vol. 4. The Bacteroidetes, Spirochaetes, Tenericutes (Mollicutes), Acidobacteria, Fibrobacteres, Fusobacteria, Dictyoglomi, Gemmatimonadetes, Lentisphaerae, Verrucomicrobia, Chlamydiae, and Planctom*, ed. W. B. Krieg, N.R., Staley, J.T., Brown, D.R., Hedlund, B.P., Paster, B.J., Ward, N.L., Ludwig, W. and Whitman (New York: Springer), 423.

Nedashkovskaya, O. I., Vancanneyt, M., Kim, S. B., and Bae, K. S. (2010). Reclassification of *Flexibacter tractuosus* (Lewin 1969) Leadbetter 1974 and “*Microscilla sericea*” Lewin 1969 in the genus *Marivirga* gen. nov. as *Marivirga tractuosa* comb. nov. and *Marivirga sericea* nom. rev., comb. nov. *Int. J. Syst. Evol. Microbiol.* 60, 1858–1863. doi:10.1099/ijs.0.016121-0.

Oren, A., and Garrity, G. M. (2015). List of new names and new combinations previously effectively, but not validly, published. *Int. J. Syst. Evol. Microbiol.* 65, 2777–2783. doi:10.1099/ijsem.0.000464.

Oren, A., and Garrity, G. M. (2016). List of new names and new combinations previously effectively, but not validly, published. *Int. J. Syst. Evol. Microbiol.* 66, 1–3. doi:10.1099/ijsem.0.000737.

Park, S., Song, J., Yoshizawa, S., Choi, A., Cho, J. C., and Kogure, K. (2013). Rubrivirga marina gen. nov., sp. nov., a member of the family Rhodothermaceae isolated from deep seawater. *Int. J. Syst. Evol. Microbiol.* 63, 2229–2233. doi:10.1099/ijs.0.046318-0.

Park, S., Yoshizawa, S., Kogure, K., and Yokota, A. (2011). Rubricoccus marinus gen. nov., sp. nov., of the family “Rhodothermaceae”, isolated from seawater. *Int. J. Syst. Evol. Microbiol.* 61, 2069–2072. doi:10.1099/ijs.0.026294-0.

Podosokorskaya, O. A., Kadnikov, V. V., Gavrilov, S. N., Mardanov, A. V., Merkel, A. Y., Karnachuk, O. V., et al. (2013). Characterization of *Melioribacter roseus* gen. nov., sp. nov., a novel facultatively anaerobic thermophilic cellulolytic bacterium from the class *Ignavibacteria*, and a proposal of a novel bacterial phylum *Ignavibacteriae*. *Environ. Microbiol.* 15, 1759–1771. doi:10.1111/1462-2920.12067.

Rosenberg, E., DeLong, E. F., Thompson, F., Lory, S., and Stackebrandt, E. (2013). *The prokaryotes: Prokaryotic biology and symbiotic associations*. Fourth Edi. , eds. E. Rosenberg,

E. F. DeLong, S. Lory, E. Stackebrandt, and F. Thompson Berlin: Springer doi:10.1007/978-3-642-30194-0.

Schmidt, M., Priemé, A., and Stougaard, P. (2006). *Rhodonellum psychrophilum* gen. nov., sp. nov., a novel psychrophilic and alkaliphilic bacterium of the phylum *Bacteroidetes* isolated from Greenland. *Int. J. Syst. Evol. Microbiol.* 56, 2887–2892. doi:10.1099/ijs.0.64450-0.

Shahina, M., Hameed, A., Lin, S. Y., Lai, W. A., Liu, Y. C., Hsu, Y. H., et al. (2013). Luteibaculum oceani gen. nov., sp. nov., a carotenoid-producing, lipolytic bacterium isolated from surface seawater, and emended description of the genus Owenweeksia Lau et al. 2005. *Int. J. Syst. Evol. Microbiol.* 63, 4765–4770. doi:10.1099/ijs.0.054635-0.

Stoecker, K., Bendinger, B., Schöning, B., Nielsen, P. H., Nielsen, J. L., Baranyi, C., et al. (2006). Cohn's Crenothrix is a filamentous methane oxidizer with an unusual methane monooxygenase. *Proc. Natl. Acad. Sci. U. S. A.* 103, 2363–2367. doi:10.1073/pnas.0506361103.

The editorial board (2011). "Genus I. Thermonema Hudson, Schofield, Morgan and Daniel 1989, 487VP," in *Bergey's Manual of Systematic Bacteriology, 2nd edition, vol. 4. The Bacteroidetes, Spirochaetes, Tenericutes (Mollicutes), Acidobacteria, Fibrobacteres, Fusobacteria, Dictyoglomi, Gemmatimonadetes, Lentisphaerae, Verrucomicrobia, Chlamydiae, and Planctom*, eds. N. R. Krieg, J. T. Staley, D. R. Brown, B. P. Hedlund, B. J. Paster, N. L. Ward, et al. (New York: Springer), 465–467.

Urios, L., Agogué, H., Lesongeur, F., Stackebrandt, E., and Lebaron, P. (2006). *Balneola vulgaris* gen. nov., sp. nov., a member of the phylum Bacteroidetes from the north-western Mediterranean Sea. *Int. J. Syst. Evol. Microbiol.* 56, 1883–7. doi:10.1099/ijs.0.64285-0.

Urios, L., Intertaglia, L., Lesongeur, F., and Lebaron, P. (2008). Balneola alkaliphila sp. nov., a marine bacterium isolated from the Mediterranean Sea. *Int. J. Syst. Evol. Microbiol.* 58, 1288–1291. doi:10.1099/ijs.0.65555-0.

Vaisman, N., and Oren, A. (2009). Salisaeta longa gen. nov., sp. nov., a red, halophilic member of the Bacteroidetes. *Int. J. Syst. Evol. Microbiol.* 59, 2571–2574. doi:10.1099/ijs.0.010892-0.

Wang, Y.-X., Liu, J.-H., Xiao, W., Zhang, X.-X., Li, Y.-Q., Lai, Y.-H., et al. (2012). Fodinibius salinus gen. nov., sp. nov., a moderately halophilic bacterium isolated from a salt mine. *Int. J. Syst. Evol. Microbiol.* 62, 390–6. doi:10.1099/ijs.0.025502-0.

Wang, Y. X., Liu, J. H., Xiao, W., Ma, X. L., Lai, Y. H., Li, Z. Y., et al. (2013). Aliifodinibius roseus gen. nov., sp. nov., and Aliifodinibius sediminis sp. nov., two moderately halophilic bacteria isolated from salt mine samples. *Int. J. Syst. Evol. Microbiol.* 63, 2907–2913. doi:10.1099/ijs.0.043869-0.

Yang, S.-H., Seo, H.-S., Woo, J.-H., Oh, H.-M., Jang, H., Lee, J.-H., et al. (2014). *Carboxylicivirga* gen. nov. in the family *Marinilabiliaceae* with two novel species, *Carboxylicivirga mesophila* sp. nov. and *Carboxylicivirga taeanensis* sp. nov., and reclassification of *Cytophaga fermentans* as <i>Sacchari. *Int. J. Syst. Evol. Microbiol.* 64, 1351–1358. doi:10.1099/ijs.0.053462-0.

Yarza, P., Richter, M., Peplies, J., Euzeby, J., Amann, R., Schleifer, K.-H., et al. (2008). The All-Species Living Tree project: a 16S rRNA-based phylogenetic tree of all sequenced type strains. *Syst. Appl. Microbiol.* 31, 241–250. doi:10.1016/j.syapm.2008.07.001.

Ying, J. Y., Wang, B. J., Yang, S. S., and Liu, S. J. (2006). Cyclobacterium lianum sp. nov., a marine bacterium isolated from sediment of an oilfield in the South China Sea, and emended description of genus Cyclobacterium. *Int. J. Syst. Evol. Microbiol.* 56, 2927–2930. doi:10.1099/ijs.0.64510-0.

Zhou, Y., Su, J., Lai, Q., Li, X., Yang, X., Dong, P., et al. (2013). *Phaeocystidibacter luteus* gen. nov., sp. nov., a member of the family *Cryomorphaceae* isolated from the marine alga *Phaeocystis globosa*, and emended description of *Owenweeksia hongkongensis*. *Int. J. Syst. Evol. Microbiol.* 63, 1143–1148. doi:10.1099/ijs.0.030254-0.
