# Supplementary material for: Genome-Based Taxonomic Classification of Bacteroidetes
Source: Front Microbiol. 2016 Dec 20;7:2003. doi: 10.3389/fmicb.2016.02003 (PMC5167729; doi:10.3389/fmicb.2016.02003)
Supplement: Supplementary file 3 [file DataSheet3.pdf]

Effect of incomplete genome sequencing on G+C content calculation
Supplementary file to “Hahnke, R.L., Meier-Kolthoff, J.P., García-López, M., Mukherjee, S., Huntemann, M., Ivanova, N., Woyke, T., C, N. Kyrpides, Hans-Peter Klenk, Markus Göker. Genome-based Taxonomic Classification of *Bacteroidetes*”

Introduction

See main manuscript.

Material and Methods

Genomes completely sequenced in the course of the GEBA and KMG-1 projects were obtained for a total of 122 type strains of species of *Bacteria* or *Archaea* (for the complete list see the appendix below), which cover a wide range of G+C content values (25.7%-74.4%). Artificial incomplete genomes were generated using the methods developed earlier [1] based on the Lander-Waterman formula [2] and a read length of 700 bp. Target genome completeness was varied between 10% and 100% with a step width of 10%. For each combination of genome completeness and original genome, 10 random replicates were conducted. The G+C content for each resulting genome was calculated with scripts developed earlier [3] and the absolute deviation from the G+C content of the respective complete genome was recorded. The relationship between overall sequence length, completeness and number of gaps was also determined under these settings.

Results

The dependency of the absolute deviation of the G+C content calculation from the respective complete genome on the sequencing completeness is shown in Figure 1. When up to 10% of the genome sequence are missing the deviation in the G+C content calculation is always below 0.1% G+C and in most cases below 0.05% G+C, i.e. an order of magnitude below the maximum deviation observed between strains of the same species [3]. Even when only 20% of the genome sequence have been obtained, the majority of the deviations is still below 0.1% G+C. Figure 2 shows the relationship between overall genome size (of the completely sequenced genome) and the expected number of fragments from genome sequencing for a completeness of 90%.

Discussion

The results indicate that the deviation from the real G+C content value caused by incomplete genome sequencing is expected to be significantly lower than the deviation between (completely sequenced) genomes from strains of distinct species [3]. Given that even a genome completeness of only 90%, which amounts to numbers of fragments between 400 and 3500 for genome lengths between 1.24 and 10.47 Mbp (Figure 2), yields absolute deviations strictly below 0.1% G+C, the advise given in our earlier study [3] to round G+C derived from genome sequences to zero decimal places values might be regarded as too careful; a single decimal place can normally be provided, even in the case of incompletely sequenced genomes.

Figures

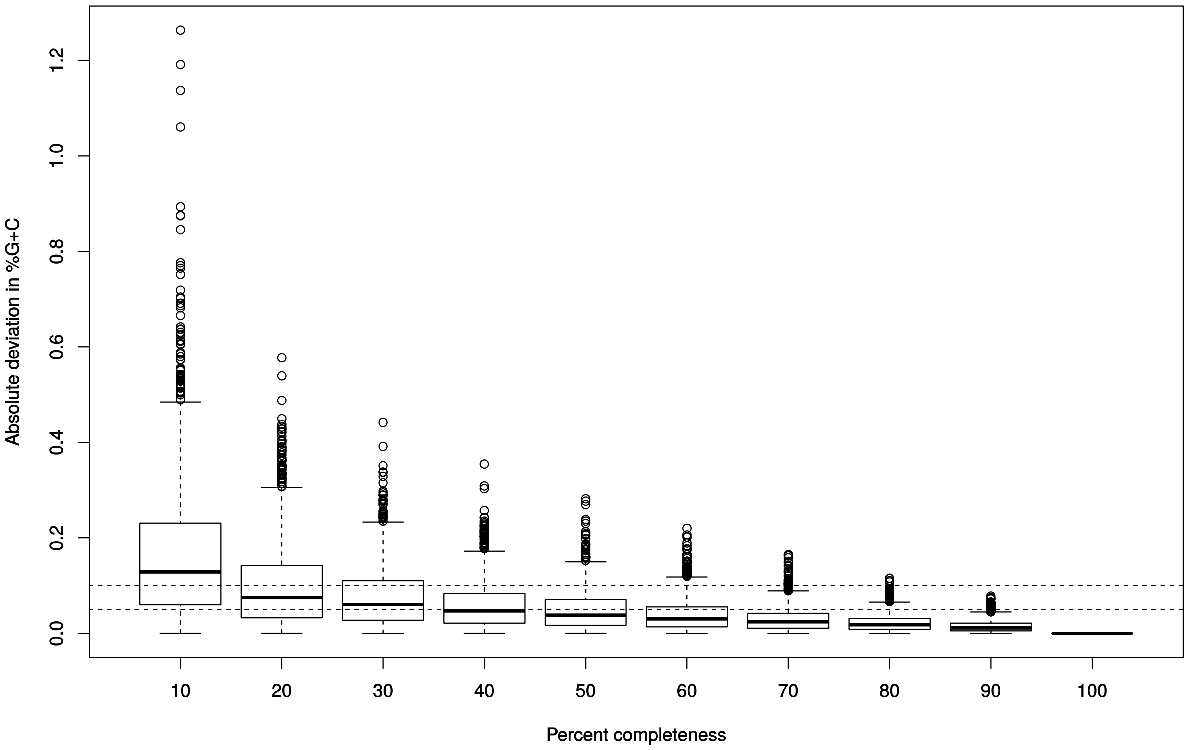

Figure 1. The dependency of the magnitude of error in G+C content calculation on sequencing completeness. The x axis is the percent completeness in genome sequencing, the y axis is the absolute deviation from the G+C content calculated from the respective complete genome. The horizontal lines correspond to a deviation of 0.1% G+C and 0.05% G+C, respectively.

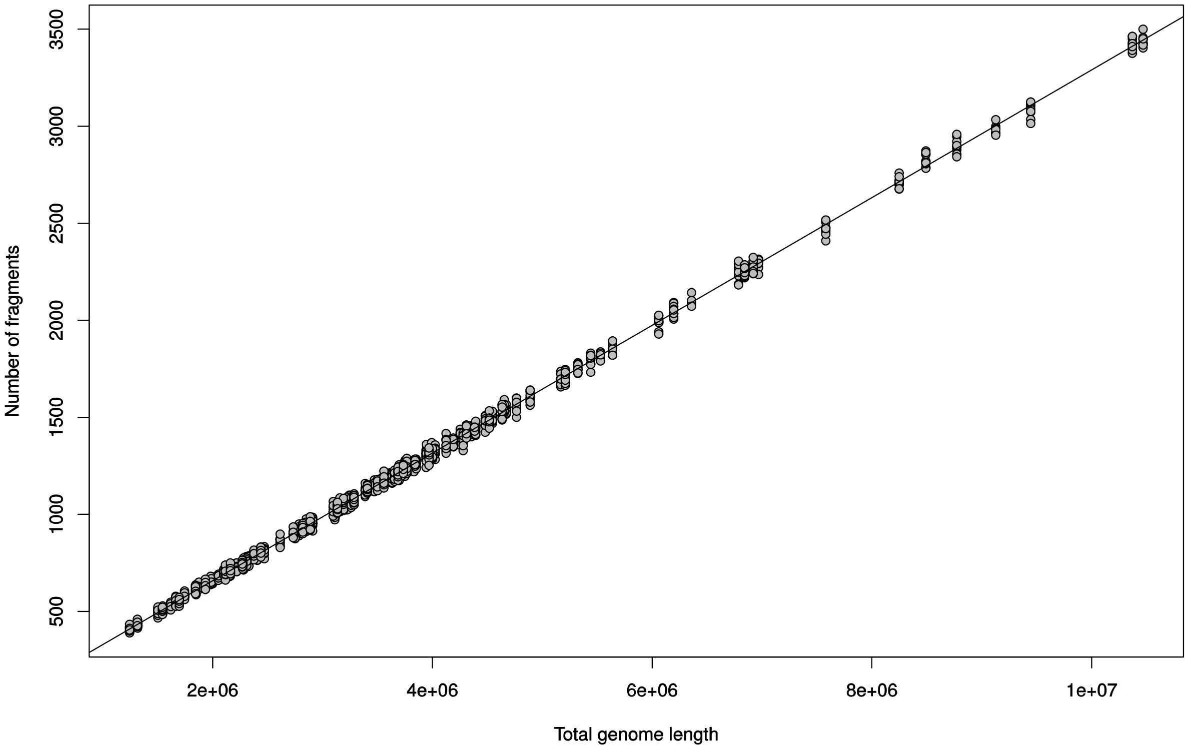

Figure 2. Dependency of the expected number of fragments from the total genome size of the completely sequenced genome for a genome completeness of 90%. The line represents a linear model of the data (slope, 0.00033; no y-intercept).

References

1. Auch AF, von Jan M, Klenk H-P, Göker M. Digital DNA-DNA hybridization for microbial species delineation by means of genome-to-genome sequence comparison. Stand Genomic Sci. 2010;2: 117.

2. Lander ES, Waterman MS. Genomic mapping by fingerprinting random clones: a mathematical analysis. Genomics. 1988;2: 231–239.

3. Meier-Kolthoff JP, Klenk H-P, Göker M. Taxonomic use of DNA G+C content and DNA-DNA hybridization in the genomic age. Int J Syst Evol Microbiol. 2014;64: 352–356. doi:10.1099/ijs.0.056994-0

Appendix: List of genomes used in this study

INSDC Accession IDs	Reference
CP002105	Sikorski, J., Lapidus, A., Lucas, S., Copeland, A., Glavina del Rio, T., Nolan, M., Tice, H., Cheng, J.-F., Han, J., Brambilla, E.-M., Pitluck, S., Liolios, K., Ivanova, N., Mavromatis, K., Mikhailova, N., Ovchinnikova, G., Pati, A., Goodwin, L.A.,

Chen, A., Palaniappan, K., Land, M., Hauser, L., Chang, Y.-J., Jeffries, C.D., Rohde, M., Göker, M., Spring, S., Woyke, T., Bristow, J., Eisen, J.A., Markowitz, V., Hugenholtz, P., Kyrpides, N.C., Klenk, H.-P. Complete genome sequence of Acetohalobium arabaticum type strain (Z-7288T). Standards in Genomic Sciences 3: 57-65, 2010.

CP001859 Chang, Y.-J., Pukall, R., Saunders, E., Lapidus, A., Copeland, A., Nolan, M., Glavina del Rio, T., Lucas, S., Chen, F., Tice, H., Cheng, J.-F., Han, J., Detter, J.C., Bruce, D., Goodwin, L.A., Pitluck, S., Mikhailova, N., Lykidis, A., Pati, A., Ivanova, N., Mavromatis, K., Chen, A., Palaniappan, K., Land, M., Hauser, L., Jeffries, C.D., Brettin, T., Rohde, M., Göker, M., Bristow, J., Eisen, J.A., Markowitz, V., Hugenholtz, P., Kyrpides, N.C., Klenk, H.-P. Complete genome sequence of Acidaminococcus fermentans type strain (VR4T). Standards in Genomic Sciences 3: 1-14, 2010.

CP001631 Clum, A., Nolan, M., Lang, E., Glavina del Rio, T., Tice, H., Copeland, A., Cheng, J.-F., Lucas, S., Chen, F., Bruce, D., Goodwin, L.A., Pitluck, S., Ivanova, N., Mavromatis, K., Mikhailova, N., Pati, A., Chen, A., Palaniappan, K., Göker, M., Spring, S., Land, M., Hauser, L., Chang, Y.-J., Jeffries, C.D., Chain, P., Bristow, J., Eisen, J.A., Markowitz, V., Hugenholtz, P., Kyrpides, N.C., Klenk, H.-P., Lapidus, A. Complete genome sequence of Acidimicrobium ferrooxidans type strain (ICPT). Standards in Genomic Sciences 1: 38-45, 2009.

CP001630 Land, M., Lapidus, A., Mayilraj, S., Chen, F., Copeland, A., Glavina del Rio, T., Nolan, M., Lucas, S., Tice, H., Cheng, J.-F., Chertkov, O., Bruce, D., Goodwin, L.A., Pitluck, S., Rohde, M., Göker, M., Pati, A., Ivanova, N., Mavromatis, K., Chen, A., Palaniappan, K., Hauser, L., Chang, Y.-J., Jeffries, C.D., Brettin, T., Detter, J.C., Han, J., Chain, P., Tindall, B.J., Bristow, J., Eisen, J.A., Markowitz, V., Hugenholtz, P., Kyrpides, N.C., Klenk, H.-P. Complete genome sequence of Actinosynnema mirum type strain (101T). Standards in Genomic Sciences 1: 46-53, 2009.

CP003274 Stackebrandt, E., Munk, A.C., Lapidus, A., Nolan, M., Lucas, S., Hammon, N., Deshpande, S., Cheng, J-F., Tapia, R., Goodwin, L.A., Pitluck, S., Liolios, K., Pagani, I., Ivanova, N., Huntemann, M., Pati, A., Chen, A., Palaniappan, K., Land, M., Hauser, L., Rohde, M., Gronow, S., Göker, M., Detter, J.C., Bristow, J., Eisen, J.A., Markowitz, V., Hugenholtz, P., Kyrpides, N.C., Klenk, H-P., Woyke, T. Complete genome sequence of the bile-resistant pigment-producing anaerobe Alistipes finegoldii type strain (AHN2437T). Standards in Genomic Sciences 8: 26-36, 2013.

CP001997 Chertkov, O., Sikorski, J., Brambilla, E.-M., Lapidus, A., Copeland, A., Glavina del Rio, T., Nolan, M., Lucas, S., Chen, F., Tice, H., Cheng, J.-F., Han, J., Detter, J.C., Woyke, T., Goodwin, L.A., Pitluck, S., Liolios, K., Ivanova, N., Mavromatis, K., Mikhailova, N., Pati, A., Chen, A., Palaniappan, K., Land, M., Hauser, L., Chang, Y.-J., Jeffries, C.D., Spring, S., Rohde, M., Göker, M., Bristow, J., Eisen, J.A., Markowitz, V., Hugenholtz, P., Kyrpides, N.C., Klenk, H.-P. Complete genome sequence of Aminobacterium colombiense type strain (ALA-1T). Standards in Genomic Sciences 2: 280-289, 2010.

CP003198 Mavromatis, K., Stackebrandt, E., Held, B., Lapidus, A., Nolan, M., Lucas, S., Hammon, N., Deshpande, S., Cheng, J-F., Tapia, R., Goodwin, L.A., Pitluck, S., Liolios, K., Pagani, I., Ivanova, N., Mikhailova, N., Huntemann, M., Pati, A., Chen, A., Palaniappan, K., Land, M., Rohde, M., Spring, S., Göker, M., Woyke, T., Detter, J.C., Bristow, J., Eisen, J.A., Markowitz, V., Hugenholtz, P., Klenk, H-P., Kyrpides, N.C. Complete genome sequence of the moderate thermophile Anaerobaculum

mobile type strain (NGAT). Standards in Genomic Sciences 8: 47-57, 2013.

CP001708; CP001709 — LaButti, K., Pukall, R., Steenblock, K., Glavina del Rio, T., Tice, H., Copeland, A., Cheng, J.-F., Lucas, S., Chen, F., Nolan, M., Bruce, D., Goodwin, L.A., Pitluck, S., Ivanova, N., Mavromatis, K., Ovchinnikova, G., Pati, A., Chen, A., Palaniappan, K., Land, M., Hauser, L., Chang, Y.-J., Jeffries, C.D., Chain, P., Saunders, E., Brettin, T., Detter, J.C., Han, J., Göker, M., Bristow, J., Eisen, J.A., Markowitz, V., Hugenholtz, P., Kyrpides, N.C., Klenk, H.-P., Lapidus, A. Complete genome sequence of Anaerococcus prevotii type strain (PC1T). Standards in Genomic Sciences 1: 159-165, 2009.

CP002045 — Yasawong, M., Teshima, H., Lapidus, A., Nolan, M., Lucas, S., Glavina del Rio, T., Tice, H., Cheng, J.-F., Bruce, D., Detter, J.C., Tapia, R., Han, J., Goodwin, L.A., Pitluck, S., Liolios, K., Ivanova, N., Mavromatis, K., Mikhailova, N., Pati, A., Chen, A., Palaniappan, K., Land, M., Hauser, L., Chang, Y.-J., Jeffries, C.D., Rohde, M., Sikorski, J., Pukall, R., Göker, M., Woyke, T., Bristow, J., Eisen, J.A., Markowitz, V., Hugenholtz, P., Kyrpides, N.C., Klenk, H.-P. Complete genome sequence of Arcanobacterium haemolyticum type strain (11018T). Standards in Genomic Sciences 3: 126-135, 2010.

CP001999 — Pati, A., Gronow, S., Lapidus, A., Copeland, A., Glavina del Rio, T., Nolan, M., Lucas, S., Tice, H., Cheng, J.-F., Han, J., Chertkov, O., Bruce, D., Tapia, R., Goodwin, L.A., Pitluck, S., Liolios, K., Ivanova, N., Mavromatis, K., Chen, A., Palaniappan, K., Land, M., Hauser, L., Chang, Y.-J., Jeffries, C.D., Detter, J.C., Rohde, M., Göker, M., Bristow, J., Eisen, J.A., Markowitz, V., Hugenholtz, P., Klenk, H.-P., Kyrpides, N.C. Complete genome sequence of Arcobacter nitrofigilis type strain (CIT). Standards in Genomic Sciences 2: 300-308, 2010.

CP001721 — Copeland, A., Sikorski, J., Lapidus, A., Nolan, M., Glavina del Rio, T., Lucas, S., Chen, F., Tice, H., Pitluck, S., Cheng, J.-F., Pukall, R., Chertkov, O., Brettin, T., Han, J., Detter, J.C., Kuske, C., Bruce, D., Goodwin, L.A., Ivanova, N., Mavromatis, K., Mikhailova, N., Chen, A., Palaniappan, K., Chain, P., Rohde, M., Göker, M., Bristow, J., Eisen, J.A., Markowitz, V., Hugenholtz, P., Kyrpides, N.C., Klenk, H.-P., Detter, J.C. Complete genome sequence of Atopobium parvulum type strain (IPP 1246T). Standards in Genomic Sciences 1: 166-173, 2009.

CP002352 — Pati, A., Gronow, S., Zeytun, A., Nolan, M., Lapidus, A., Nolan, M., Hammon, N., Deshpande, S., Cheng, J.-F., Tapia, R., Han, J., Goodwin, L.A., Pitluck, S., Liolios, K., Pagani, I., Ivanova, N., Mavromatis, K., Chen, A., Palaniappan, K., Land, M., Hauser, L., Chang, Y.-J., Jeffries, C.D., Detter, J.C., Brambilla, E.-M., Rohde, M., Göker, M., Woyke, T., Bristow, J., Eisen, J.A., Markowitz, V., Hugenholtz, P., Kyrpides, N.C., Klenk, H.-P., Lucas, S. Complete genome sequence of Bacteroides helcogenes type strain (P 36-108T). Standards in Genomic Sciences 4: 45-53, 2011.

CP002530; CP002531; CP002532; CP002533 — Gronow, S., Held, B., Lucas, S., Lapidus, A., Glavina del Rio, T., Nolan, M., Tice, H., Cheng, J.-F., Pitluck, S., Liolios, K., Pagani, I., Ivanova, N., Mavromatis, K., Pati, A., Tapia, R., Han, J., Goodwin, L.A., Chen, A., Palaniappan, K., Land, M., Hauser, L., Chang, Y.-J., Jeffries, C.D., Brambilla, E.-M., Welnitz, S., Rohde, M., Göker, M., Detter, J.C., Woyke, T., Bristow, J., Markowitz, V., Hugenholtz, P., Kyrpides, N.C., Klenk, H.-P., Eisen, J.A. Complete genome sequence of Bacteroides salanitronis type strain (BL78T). Standards in Genomic Sciences 4: 191-199, 2011.

CP001618 — Land, M., Pukall, R., Abt, B., Göker, M., Rohde, M., Glavina del Rio, T., Tice, H., Copeland, A., Cheng, J.-F., Lucas, S., Chen, F., Nolan, M., Bruce, D., Goodwin, L.A., Pitluck, S., Ivanova, N., Mavromatis, K., Ovchinnikova, G., Pati, A., Chen, A., Palaniappan, K., Hauser, L., Chang, Y.-J., Jeffries, C.D., Saunders, E., Brettin, T.,

Detter, J.C., Han, J., Chain, P., Bristow, J., Eisen, J.A., Markowitz, V., Hugenholtz, P., Kyrpides, N.C., Klenk, H.-P., Lapidus, A. Complete genome sequence of Beutenbergia cavernae type strain (HKI 0122T). Standards in Genomic Sciences 1: 21-28, 2009.

CP001643 Lapidus, A., Pukall, R., LaButti, K., Copeland, A., Glavina del Rio, T., Nolan, M., Chen, F., Lucas, S., Tice, H., Cheng, J.-F., Bruce, D., Goodwin, L.A., Pitluck, S., Rohde, M., Göker, M., Pati, A., Ivanova, N., Mavromatis, K., Chen, A., Palaniappan, K., D'haeseleer, P., Chain, P., Bristow, J., Eisen, J.A., Markowitz, V., Hugenholtz, P., Kyrpides, N.C., Klenk, H.-P. Complete genome sequence of Brachybacterium faecium type strain (Schefferle 6-10T). Standards in Genomic Sciences 1: 3-11, 2009.

CP001959 Pati, A., Sikorski, J., Gronow, S., Lapidus, A., Copeland, A., Glavina del Rio, T., Nolan, M., Lucas, S., Chen, F., Tice, H., Cheng, J.-F., Han, J., Detter, J.C., Bruce, D., Goodwin, L.A., Pitluck, S., Ivanova, N., Mavromatis, K., Mikhailova, N., Chen, A., Palaniappan, K., Land, M., Hauser, L., Chang, Y.-J., Jeffries, C.D., Chain, P., Spring, S., Rohde, M., Göker, M., Bristow, J., Eisen, J.A., Markowitz, V., Hugenholtz, P., Kyrpides, N.C., Klenk, H.-P. Complete genome sequence of Brachyspira murdochii type strain (56-150T). Standards in Genomic Sciences 2: 260-269, 2010.

CP002347; CP002348 Pitluck, S., Zeytun, A., Lapidus, A., Nolan, M., Lucas, S., Hammon, N., Deshpande, S., Cheng, J.-F., Tapia, R., Han, J., Goodwin, L.A., Pitluck, S., Liolios, K., Pagani, I., Ivanova, N., Mavromatis, K., Pati, A., Chen, A., Palaniappan, K., Hauser, L., Chang, Y.-J., Jeffries, C.D., Detter, J.C., Brambilla, E.-M., Ngatchou Djao, O.D., Rohde, M., Spring, S., Göker, M., Woyke, T., Bristow, J., Eisen, J.A., Markowitz, V., Hugenholtz, P., Kyrpides, N.C., Klenk, H.-P., Land, M. Complete genome sequence of Calditerrivibrio nitroreducens type strain (Yu37-1T). Standards in Genomic Sciences 4: 54-62, 2011.

CP001632 Mavromatis, K., Gronow, S., Saunders, E., Land, M., Lapidus, A., Copeland, A., Glavina del Rio, T., Nolan, M., Lucas, S., Chen, F., Tice, H., Cheng, J.-F., Bruce, D., Goodwin, L.A., Pitluck, S., Pati, A., Ivanova, N., Chen, A., Palaniappan, K., Chain, P., Hauser, L., Chang, Y.-J., Jeffries, C.D., Brettin, T., Detter, J.C., Han, J., Bristow, J., Göker, M., Rohde, M., Eisen, J.A., Markowitz, V., Kyrpides, N.C., Klenk, H.-P., Hugenholtz, P. Complete genome sequence of Capnocytophaga ochracea type strain (VPI 2845T). Standards in Genomic Sciences 1: 101-109, 2009.

CP001700 Copeland, A., Lapidus, A., Glavina del Rio, T., Nolan, M., Lucas, S., Chen, F., Tice, H., Cheng, J.-F., Bruce, D., Goodwin, L.A., Pitluck, S., Mikhailova, N., Pati, A., Ivanova, N., Mavromatis, K., Chen, A., Chain, P., Land, M., Hauser, L., Chang, Y.-J., Jeffries, C.D., Chertkov, O., Brettin, T., Detter, J.C., Han, J., Ali, Z., Tindall, B.J., Göker, M., Bristow, J., Eisen, J.A., Markowitz, V., Hugenholtz, P., Kyrpides, N.C., Klenk, H.-P. Complete genome sequence of Catenulispora acidiphila type strain (ID 139908T). Standards in Genomic Sciences 1: 119-128, 2009.

CP001964 Abt, B., Lucas, S., Lapidus, A., Pukall, R., Glavina del Rio, T., Nolan, M., Tice, H., Cheng, J.-F., Pitluck, S., Liolios, K., Ivanova, N., Mavromatis, K., Mikhailova, N., Ovchinnikova, G., Pati, A., Goodwin, L.A., Chen, A., Palaniappan, K., Land, M., Hauser, L., Chang, Y.-J., Jeffries, C.D., Rohde, M., Göker, M., Woyke, T., Bristow, J., Eisen, J.A., Markowitz, V., Hugenholtz, P., Kyrpides, N.C., Klenk, H.-P. Complete genome sequence of Cellulomonas flavigena type strain (134T). Standards in Genomic Sciences 3: 2-25, 2010.

CP002453 Abt, B., Lu, M., Misra, M., Han, J., Nolan, M., Lucas, S., Hammon, N., Deshpande,

S., Cheng, J.-F., Tapia, R., Goodwin, L.A., Pitluck, S., Liolios, K., Pagani, I., Ivanova, N., Mavromatis, K., Ovchinnikova, G., Pati, A., Chen, A., Palaniappan, K., Land, M., Hauser, L., Chang, Y.-J., Jeffries, C.D., Detter, J.C., Brambilla, E.-M., Rohde, M., Tindall, B.J., Göker, M., Woyke, T., Bristow, J., Eisen, J.A., Markowitz, V., Hugenholtz, P., Kyrpides, N.C., Klenk, H.-P., Lapidus, A. Complete genome sequence of Cellulophaga algicola type strain (IC166T). Standards in Genomic Sciences 4: 72-80, 2011.

CP002534 Pati, A., Abt, B., Teshima, H., Lu, M., Misra, M., Nolan, M., Lapidus, A., Lucas, S., Hammon, N., Deshpande, S., Cheng, J.-F., Tapia, R., Han, J., Goodwin, L.A., Pitluck, S., Liolios, K., Pagani, I., Mavromatis, K., Ovchinnikova, G., Chen, A., Palaniappan, K., Land, M., Hauser, L., Jeffries, C.D., Detter, J.C., Brambilla, E.-M., Kannan, K.P., Rohde, M., Spring, S., Göker, M., Woyke, T., Bristow, J., Eisen, J.A., Markowitz, V., Hugenholtz, P., Kyrpides, N.C., Klenk, H.-P., Ivanova, N. Complete genome sequence of Cellulophaga lytica type strain (LIM-21T). Standards in Genomic Sciences 4: 221-233, 2011.

CP001699 Glavina del Rio, T., Abt, B., Spring, S., Lapidus, A., Nolan, M., Tice, H., Copeland, A., Cheng, J.-F., Chen, F., Bruce, D., Goodwin, L.A., Pitluck, S., Ivanova, N., Mavromatis, K., Mikhailova, N., Pati, A., Chen, A., Palaniappan, K., Land, M., Hauser, L., Chang, Y.-J., Jeffries, C.D., Chain, P., Saunders, E., Detter, J.C., Brettin, T., Rohde, M., Göker, M., Bristow, J., Eisen, J.A., Markowitz, V., Hugenholtz, P., Kyrpides, N.C., Klenk, H.-P., Lucas, S. Complete genome sequence of Chitinophaga pinensis type strain (UQM 2034T). Standards in Genomic Sciences 2: 87-95, 2010.

CP000285 Copeland, A., O'Connor, K., Lucas, S., Lapidus, A., Barry, K.W., Detter, J.C., Glavina del Rio, T., Hammon, N., Dalin, E., Tice, H., Pitluck, S., Bruce, D., Goodwin, L.A., Han, J., Tapia, R., Saunders, E., Schmutz, J., Brettin, T., Larimer, F., Land, M., Hauser, L., Vargas, C., Nieto, J.J., Kyrpides, N.C., Ivanova, N., Göker, M., Klenk, H.-P., Csonka, L.N., Woyke, T. Complete genome sequence of the halophilic and highly halotolerant Chromohalobacter salexigens type strain (1H11T). Standards in Genomic Sciences 5: 379-388, 2011.

CP001854 Pukall, R., Lapidus, A., Glavina del Rio, T., Copeland, A., Tice, H., Cheng, J.-F., Lucas, S., Chen, F., Nolan, M., Bruce, D., Goodwin, L.A., Pitluck, S., Mavromatis, K., Ivanova, N., Ovchinnikova, G., Pati, A., Chen, A., Palaniappan, K., Land, M., Hauser, L., Chang, Y.-J., Jeffries, C.D., Chain, P., Meinecke, L., Sims, D., Brettin, T., Detter, J.C., Rohde, M., Göker, M., Bristow, J., Eisen, J.A., Markowitz, V., Kyrpides, N.C., Klenk, H.-P., Hugenholtz, P. Complete genome sequence of Conexibacter woesei type strain (ID131577T). Standards in Genomic Sciences 2: 212-219, 2010.

CP001998 Mavromatis, K., Abt, B., Brambilla, E.-M., Lapidus, A., Copeland, A., Deshpande, S., Nolan, M., Lucas, S., Tice, H., Cheng, J.-F., Han, J., Detter, J.C., Woyke, T., Goodwin, L.A., Pitluck, S., Held, B., Brettin, T., Tapia, R., Ivanova, N., Mikhailova, N., Pati, A., Liolios, K., Chen, A., Palaniappan, K., Land, M., Hauser, L., Chang, Y.-J., Jeffries, C.D., Rohde, M., Göker, M., Bristow, J., Eisen, J.A., Markowitz, V., Hugenholtz, P., Klenk, H.-P., Kyrpides, N.C. Complete genome sequence of Coraliomargarita akajimensis type strain (04OKA010-24T). Standards in Genomic Sciences 2: 290-299, 2010.

CP002628 Stackebrandt, E., Zeytun, A., Lapidus, A., Nolan, M., Lucas, S., Hammon, N., Deshpande, S., Cheng, J-F., Tapia, R., Goodwin, L.A., Pitluck, S., Liolios, K., Pagani, I., Ivanova, N., Mavromatis, K., Mikhailova, N., Huntemann, M., Pati, A., Chen, A., Palaniappan, K., Chang, Y-J., Land, M., Hauser, L., Rohde, M., Pukall, R., Göker, M., Detter, J.C., Woyke, T., Bristow, J., Eisen, J.A., Markowitz, V.,

Hugenholtz, P., Kyrpides, N.C., Klenk, H-P. Complete genome sequence of Coriobacterium glomerans type strain (PW2T) from the midgut of Pyrrhocoris apterus L. (red soldier bug). Standards in Genomic Sciences 8: 15-25, 2013.

CP001682 Mavromatis, K., Pukall, R., Rohde, C., Chen, F., Sims, D., Brettin, T., Kuske, C., Detter, J.C., Han, J., Lapidus, A., Copeland, A., Glavina del Rio, T., Nolan, M., Lucas, S., Tice, H., Cheng, J.-F., Bruce, D., Goodwin, L.A., Pitluck, S., Ovchinnikova, G., Pati, A., Ivanova, N., Chen, A., Palaniappan, K., Chain, P., D'haeseleer, P., Göker, M., Bristow, J., Eisen, J.A., Markowitz, V., Hugenholtz, P., Rohde, M., Klenk, H.-P., Kyrpides, N.C. Complete genome sequence of Cryptobacterium curtum type strain (12-3T). Standards in Genomic Sciences 1: 93-100, 2009.

CP002454 Pukall, R., Zeytun, A., Lucas, S., Lapidus, A., Hammon, N., Deshpande, S., Nolan, M., Cheng, J.-F., Pitluck, S., Liolios, K., Pagani, I., Mikhailova, N., Ivanova, N., Mavromatis, K., Pati, A., Tapia, R., Han, J., Goodwin, L.A., Chen, A., Palaniappan, K., Land, M., Hauser, L., Chang, Y.-J., Jeffries, C.D., Brambilla, E.-M., Rohde, M., Göker, M., Detter, J.C., Woyke, T., Bristow, J., Eisen, J.A., Markowitz, V., Hugenholtz, P., Kyrpides, N.C., Klenk, H.-P. Complete genome sequence of Deinococcus maricopensis type strain (LB-34T). Standards in Genomic Sciences 4: 163-172, 2011.

CP002536; CP002537; CP002538; CP002539; CP002540 Copeland, A., Zeytun, A., Yasawong, M., Nolan, M., Lucas, S., Hammon, N., Deshpande, S., Cheng, J.-F., Han, J., Tapia, R., Goodwin, L.A., Pitluck, S., Mavromatis, K., Liolios, K., Pagani, I., Ivanova, N., Mikhailova, N., Pati, A., Chen, A., Palaniappan, K., Land, M., Hauser, L., Jeffries, C.D., Brambilla, E.-M., Rohde, M., Sikorski, J., Pukall, R., Göker, M., Detter, J.C., Woyke, T., Bristow, J., Eisen, J.A., Markowitz, V., Hugenholtz, P., Kyrpides, N.C., Klenk, H.-P., Lapidus, A. Complete genome sequence of the orange-red pigmented, radioresistant Deinococcus proteolyticus type strain (MRPT). Standards in Genomic Sciences 6: 240-250, 2012.

CP001968 Kiss, H., Lang, E., Lapidus, A., Copeland, A., Nolan, M., Glavina del Rio, T., Chen, F., Lucas, S., Tice, H., Cheng, J.-F., Han, J., Goodwin, L.A., Pitluck, S., Liolios, K., Pati, A., Ivanova, N., Mavromatis, K., Chen, A., Palaniappan, K., Land, M., Hauser, L., Chang, Y.-J., Jeffries, C.D., Detter, J.C., Brettin, T., Spring, S., Rohde, M., Göker, M., Woyke, T., Bristow, J., Eisen, J.A., Markowitz, V., Hugenholtz, P., Kyrpides, N.C., Klenk, H.-P. Complete genome sequence of Denitrovibrio acetiphilus type strain (N2460T). Standards in Genomic Sciences 2: 270-279, 2010.

CP002085 Sun, H., Spring, S., Lapidus, A., Davenport, K., Glavina del Rio, T., Tice, H., Nolan, M., Copeland, A., Cheng, J.-F., Lucas, S., Tapia, R., Goodwin, L.A., Pitluck, S., Ivanova, N., Mavromatis, K., Ovchinnikova, G., Pati, A., Chen, A., Palaniappan, K., Hauser, L., Chang, Y.-J., Jeffries, C.D., Detter, J.C., Han, J., Rohde, M., Brambilla, E.-M., Göker, M., Woyke, T., Bristow, J., Eisen, J.A., Markowitz, V., Hugenholtz, P., Kyrpides, N.C., Land, M., Klenk, H.-P. Complete genome sequence of Desulfarculus baarsii type strain (2st14T). Standards in Genomic Sciences 3: 276-284, 2010 (doi:10.4056/sigs.124325).

CP002629 Göker, M., Teshima, H., Lapidus, A., Nolan, M., Lucas, S., Hammon, N., Deshpande, S., Cheng, J.-F., Tapia, R., Han, J., Goodwin, L.A., Pitluck, S., Huntemann, M., Liolios, K., Ivanova, N., Pagani, I., Mavromatis, K., Ovchinnikova, G., Pati, A., Chen, A., Palaniappan, K., Land, M., Hauser, L., Brambilla, E.-M., Rohde, M., Spring, S., Detter, J.C., Woyke, T., Bristow, J., Eisen, J.A., Markowitz, V., Hugenholtz, P., Kyrpides, N.C., Klenk, H.-P. Complete genome sequence of the acetate-degrading sulfate reducer Desulfobacca acetoxidans type strain (ASRB2T).

Standards in Genomic Sciences 4: 393-401, 2011.

CP002364	Pagani, I., Lapidus, A., Nolan, M., Lucas, S., Hammon, N., Deshpande, S., Cheng, J.-F., Chertkov, O., Davenport, K., Tapia, R., Han, J., Goodwin, L.A., Pitluck, S., Liolios, K., Mavromatis, K., Ivanova, N., Mikhailova, N., Pati, A., Chen, A., Palaniappan, K., Land, M., Hauser, L., Chang, Y.-J., Jeffries, C.D., Detter, J.C., Brambilla, E.-M., Kannan, K.P., Ngatchou Djao, O.D., Rohde, M., Pukall, R., Spring, S., Göker, M., Sikorski, J., Woyke, T., Bristow, J., Eisen, J.A., Markowitz, V., Hugenholtz, P., Kyrpides, N.C., Klenk, H.-P. Complete genome sequence of Desulfobulbus propionicus type strain (1pr3T). Standards in Genomic Sciences 4: 100-110, 2011.
CP001734; CP001735	Spring, S., Nolan, M., Lapidus, A., Glavina del Rio, T., Copeland, A., Tice, H., Cheng, J.-F., Lucas, S., Land, M., Chen, F., Bruce, D., Goodwin, L.A., Pitluck, S., Ivanova, N., Mavromatis, K., Mikhailova, N., Pati, A., Chen, A., Palaniappan, K., Hauser, L., Chang, Y.-J., Jeffries, C.D., Munk, A.C., Kiss, H., Han, J., Brettin, T., Detter, J.C., Schüler, E., Göker, M., Rohde, M., Bristow, J., Eisen, J.A., Markowitz, V., Hugenholtz, P., Kyrpides, N.C., Klenk, H.-P. Complete genome sequence of Desulfohalobium retbaense type strain (HR100T). Standards in Genomic Sciences 2: 38-48, 2010.
CP001629	Copeland, A., Spring, S., Göker, M., Schneider, S., Lapidus, A., Glavina del Rio, T., Tice, H., Cheng, J.-F., Lucas, S., Chen, F., Nolan, M., Bruce, D., Goodwin, L.A., Pitluck, S., Ivanova, N., Mavromatis, K., Ovchinnikova, G., Pati, A., Chen, A., Palaniappan, K., Land, M., Hauser, L., Chang, Y.-J., Jeffries, C.D., Meinecke, L., Sims, D., Brettin, T., Detter, J.C., Han, J., Chain, P., Bristow, J., Eisen, J.A., Markowitz, V., Hugenholtz, P., Klenk, H.-P., Kyrpides, N.C. Complete genome sequence of Desulfomicrobium baculatum type strain (XT). Standards in Genomic Sciences 1: 29-37, 2009.
CP001720	Spring, S., Lapidus, A., Schröder, M., Gleim, D., Sims, D., Meinecke, L., Glavina del Rio, T., Tice, H., Copeland, A., Cheng, J.-F., Lucas, S., Chen, F., Nolan, M., Bruce, D., Goodwin, L.A., Pitluck, S., Ivanova, N., Mavromatis, K., Mikhailova, N., Pati, A., Chen, A., Palaniappan, K., Land, M., Hauser, L., Chang, Y.-J., Jeffries, C.D., Chain, P., Saunders, E., Brettin, T., Detter, J.C., Göker, M., Bristow, J., Eisen, J.A., Markowitz, V., Hugenholtz, P., Kyrpides, N.C., Klenk, H.-P., Han, J. Complete genome sequence of Desulfotomaculum acetoxidans type strain (5575T). Standards in Genomic Sciences 1: 242-253, 2009.
CP002780	Spring, S., Visser, M., Lu, M., Copeland, A., Lapidus, A., Lucas, S., Cheng, J-F., Han, J., Tapia, R., Goodwin, L.A., Pitluck, S., Ivanova, N., Land, M., Hauser, L., Larimer, F., Rohde, M., Göker, M., Detter, J.C., Kyrpides, N.C., Woyke, T., Schaap, P., Plugge, C.M., Muyzer, G., Kuever, J., Pereira, I., Parshina, S.N., Berner-Latmani, R., Stams, A., Klenk, H-P. Complete genome sequence of the sulfate-reducing firmicute Desulfotomaculum ruminis type strain (DLT). Standards in Genomic Sciences 7: 304-319, 2012 (doi:10.4056/sigs.3226659).
CP002543	Göker, M., Daligault, H., Mwirichia, R., Lapidus, A., Lucas, S., Deshpande, S., Pagani, I., Tapia, R., Cheng, J.-F., Goodwin, L.A., Pitluck, S., Liolios, K., Ivanova, N., Mavromatis, K., Mikhailova, N., Pati, A., Chen, A., Palaniappan, K., Han, J., Land, M., Hauser, L., Pan, C., Brambilla, E.-M., Rohde, M., Spring, S., Sikorski, J., Wirth, R., Detter, J.C., Woyke, T., Bristow, J., Eisen, J.A., Markowitz, V., Hugenholtz, P., Kyrpides, N.C., Klenk, H.-P. Complete genome sequence of the thermophilic sulfur-reducer Desulfurobacterium thermolithotrophum type strain (BSAT) from a deep-sea hydrothermal vent. Standards in Genomic Sciences 5: 407-415, 2011.

CP002363 Wirth, R., Chertkov, O., Held, B., Lapidus, A., Nolan, M., Lucas, S., Hammon, N., Deshpande, S., Cheng, J.-F., Tapia, R., Han, J., Goodwin, L.A., Pitluck, S., Liolios, K., Pagani, I., Ivanova, N., Mavromatis, K., Mikhailova, N., Pati, A., Chen, A., Palaniappan, K., Land, M., Hauser, L., Chang, Y.-J., Jeffries, C.D., Bilek, Y., Hader, T., Rohde, M., Spring, S., Sikorski, J., Göker, M., Woyke, T., Bristow, J., Eisen, J.A., Markowitz, V., Hugenholtz, P., Kyrpides, N.C., Klenk, H.-P. Complete genome sequence of Desulfurococcus mucosus type strain (O7/1T). Standards in Genomic Sciences 4: 173-182, 2010.

CP001619 Lang, E., Lapidus, A., Chertkov, O., Brettin, T., Detter, J.C., Han, J., Copeland, A., Glavina del Rio, T., Nolan, M., Chen, F., Lucas, S., Tice, H., Cheng, J.-F., Land, M., Hauser, L., Bruce, D., Goodwin, L.A., Pitluck, S., Ovchinnikova, G., Pati, A., Ivanova, N., Mavromatis, K., Chen, A., Chain, P., Bristow, J., Eisen, J.A., Markowitz, V., Hugenholtz, P., Göker, M., Rohde, M., Kyrpides, N.C., Klenk, H.-P. Complete genome sequence of Dyadobacter fermentans type strain (NS114T). Standards in Genomic Sciences 1: 133-140, 2009.

CP001726 Saunders, E., Pukall, R., Abt, B., Lapidus, A., Glavina del Rio, T., Copeland, A., Tice, H., Cheng, J.-F., Lucas, S., Chen, F., Nolan, M., Bruce, D., Goodwin, L.A., Pitluck, S., Ivanova, N., Mavromatis, K., Ovchinnikova, G., Pati, A., Chen, A., Palaniappan, K., Land, M., Hauser, L., Chang, Y.-J., Jeffries, C.D., Chain, P., Meinecke, L., Sims, D., Brettin, T., Detter, J.C., Göker, M., Bristow, J., Eisen, J.A., Markowitz, V., Hugenholtz, P., Kyrpides, N.C., Klenk, H.-P., Han, J. Complete genome sequence of Eggerthella lenta type strain (IPP VPI 0255T). Standards in Genomic Sciences 1: 174-182, 2009.

CP002209 Nolan, M., Sikorski, J., Davenport, K., Lucas, S., Glavina del Rio, T., Tice, H., Cheng, J.-F., Goodwin, L.A., Pitluck, S., Liolios, K., Ivanova, N., Mavromatis, K., Ovchinnikova, G., Pati, A., Chen, A., Palaniappan, K., Land, M., Hauser, L., Chang, Y.-J., Jeffries, C.D., Tapia, R., Brettin, T., Detter, J.C., Han, J., Yasawong, M., Rohde, M., Tindall, B.J., Göker, M., Woyke, T., Bristow, J., Eisen, J.A., Markowitz, V., Hugenholtz, P., Kyrpides, N.C., Klenk, H.-P., Lapidus, A. Complete genome sequence of Ferrimonas balearica type strain (PATT). Standards in Genomic Sciences 3: 174-182, 2010.

CP002542 Woyke, T., Chertkov, O., Lapidus, A., Nolan, M., Lucas, S., Glavina del Rio, T., Tice, H., Cheng, J.-F., Tapia, R., Han, J., Goodwin, L.A., Pitluck, S., Liolios, K., Pagani, I., Ivanova, N., Huntemann, M., Mavromatis, K., Mikhailova, N., Pati, A., Chen, A., Palaniappan, K., Land, M., Hauser, L., Brambilla, E.-M., Rohde, M., Mwirichia, R., Sikorski, J., Tindall, B.J., Göker, M., Bristow, J., Eisen, J.A., Markowitz, V., Hugenholtz, P., Klenk, H.-P., Kyrpides, N.C. Complete genome sequence of the gliding freshwater bacterium Fluviicola taffensis type strain (RW262T). Standards in Genomic Sciences 5: 21-29, 2011.

CP001867 Ivanova, N., Sikorski, J., Jando, M., Munk, A.C., Lapidus, A., Glavina del Rio, T., Copeland, A., Tice, H., Cheng, J.-F., Lucas, S., Chen, F., Nolan, M., Bruce, D., Goodwin, L.A., Pitluck, S., Mavromatis, K., Mikhailova, N., Pati, A., Chen, A., Palaniappan, K., Land, M., Hauser, L., Chang, Y.-J., Jeffries, C.D., Meinecke, L., Brettin, T., Detter, J.C., Rohde, M., Göker, M., Bristow, J., Eisen, J.A., Markowitz, V., Hugenholtz, P., Kyrpides, N.C., Klenk, H.-P. Complete genome sequence of Geodermatophilus obscurus type strain (G-20T). Standards in Genomic Sciences 2: 158-167, 2010.

CP001802 Ivanova, N., Sikorski, J., Jando, M., Lapidus, A., Nolan, M., Lucas, S., Glavina del Rio, T., Tice, H., Copeland, A., Cheng, J.-F., Chen, F., Bruce, D., Goodwin, L.A., Pitluck, S., Mavromatis, K., Ovchinnikova, G., Pati, A., Chen, A., Palaniappan, K.,

Land, M., Hauser, L., Chang, Y.-J., Jeffries, C.D., Chain, P., Saunders, E., Han, J., Detter, J.C., Brettin, T., Rohde, M., Göker, M., Bristow, J., Eisen, J.A., Markowitz, V., Hugenholtz, P., Klenk, H.-P., Kyrpides, N.C. Complete genome sequence of Gordonia bronchialis type strain (3410T). Standards in Genomic Sciences 2: 19-28, 2010.

CP002175 Ivanova, N., Sikorski, J., Chertkov, O., Nolan, M., Lucas, S., Hammon, N., Deshpande, S., Cheng, J.-F., Tapia, R., Han, J., Goodwin, L.A., Pitluck, S., Huntemann, M., Liolios, K., Pagani, I., Mavromatis, K., Ovchinnikova, G., Pati, A., Chen, A., Palaniappan, K., Land, M., Hauser, L., Brambilla, E.-M., Kannan, K.P., Rohde, M., Tindall, B.J., Göker, M., Detter, J.C., Woyke, T., Bristow, J., Eisen, J.A., Markowitz, V., Hugenholtz, P., Kyrpides, N.C., Klenk, H.-P., Lapidus, A. Complete genome sequence of Halanaerobium praevalens type strain (GSLT). Standards in Genomic Sciences 4: 312-321, 2011.

CP001804 Ivanova, N., Daum, C., Lang, E., Abt, B., Kopitz, M., Saunders, E., Lapidus, A., Lucas, S., Glavina del Rio, T., Nolan, M., Tice, H., Copeland, A., Cheng, J.-F., Bruce, D., Goodwin, L.A., Pitluck, S., Ivanova, N., Chen, A., Palaniappan, K., Land, M., Hauser, L., Chang, Y.-J., Jeffries, C.D., Detter, J.C., Brettin, T., Rohde, M., Göker, M., Bristow, J., Eisen, J.A., Markowitz, V., Hugenholtz, P., Kyrpides, N.C., Klenk, H.-P., Lucas, S. Complete genome sequence of Haliangium ochraceum type strain (SMP-2T). Standards in Genomic Sciences 2: 96-106, 2010.

CP002691; CP002692; CP002693; CP002694 Daligault, H., Lapidus, A., Zeytun, A., Nolan, M., Lucas, S., Glavina del Rio, T., Tice, H., Cheng, J.-F., Tapia, R., Han, J., Goodwin, L.A., Pitluck, S., Liolios, K., Pagani, I., Ivanova, N., Huntemann, M., Mavromatis, K., Mikhailova, N., Pati, A., Chen, A., Palaniappan, K., Land, M., Hauser, L., Brambilla, E.-M., Rohde, M., Verbarg, S., Göker, M., Bristow, J., Eisen, J.A., Markowitz, V., Hugenholtz, P., Kyrpides, N.C., Klenk, H.-P., Woyke, T. Complete genome sequence of Haliscomenobacter hydrossis type strain (OT). Standards in Genomic Sciences 4: 352-360, 2011.

CP001690; CP001691; CP001692; CP001693; CP001694; CP001695 Malfatti, S., Tindall, B.J., Schneider, S., Fähnrich, R., Lapidus, A., LaButti, K., Copeland, A., Glavina del Rio, T., Nolan, M., Chen, F., Lucas, S., Tice, H., Cheng, J.-F., Bruce, D., Goodwin, L.A., Pitluck, S., Anderson, I.J., Pati, A., Ivanova, N., Mavromatis, K., Chen, A., Palaniappan, K., D'haeseleer, P., Göker, M., Bristow, J., Eisen, J.A., Markowitz, V., Hugenholtz, P., Kyrpides, N.C., Klenk, H.-P., Chain, P. Complete genome sequence of Halogeometricum borinquense type strain (PR3T). Standards in Genomic Sciences 1: 150-158, 2009.

CP001688 Tindall, B.J., Schneider, S., Lapidus, A., Copeland, A., Glavina del Rio, T., Nolan, M., Lucas, S., Chen, F., Tice, H., Cheng, J.-F., Saunders, E., Bruce, D., Goodwin, L.A., Pitluck, S., Mikhailova, N., Pati, A., Ivanova, N., Mavromatis, K., Chen, A., Palaniappan, K., Chain, P., Land, M., Hauser, L., Chang, Y.-J., Jeffries, C.D., Brettin, T., Han, J., Rohde, M., Göker, M., Bristow, J., Eisen, J.A., Markowitz, V., Hugenholtz, P., Klenk, H.-P., Kyrpides, N.C., Detter, J.C. Complete genome sequence of Halomicrobium mukohataei type strain (arg-2T). Standards in Genomic Sciences 1: 270-277, 2009.

CP001687 Anderson, I.J., Tindall, B.J., Göker, M., Lapidus, A., Nolan, M., Copeland, A., Glavina del Rio, T., Chen, F., Tice, H., Cheng, J.-F., Lucas, S., Chertkov, O., Bruce, D., Brettin, T., Detter, J.C., Han, J., Goodwin, L.A., Land, M., Hauser, L., Chang, Y.-J., Jeffries, C.D., Pitluck, S., Pati, A., Mavromatis, K., Ivanova, N., Ovchinnikova, G., Chen, A., Palaniappan, K., Chain, P., Rohde, M., Bristow, J., Eisen, J.A., Markowitz, V., Hugenholtz, P., Kyrpides, N.C., Klenk, H.-P. Complete genome sequence of Halorhabdus utahensis type strain (AX-2T). Standards in Genomic

Sciences 1: 218-225, 2009.

CP001860; CP001861; CP001862; CP001863; CP001864; CP001865; CP001866 — Saunders, E., Tindall, B.J., Fähnrich, R., Lapidus, A., Copeland, A., Glavina del Rio, T., Lucas, S., Chen, F., Tice, H., Cheng, J.-F., Han, J., Detter, J.C., Bruce, D., Goodwin, L.A., Chain, P., Pitluck, S., Pati, A., Ivanova, N., Mavromatis, K., Chen, A., Palaniappan, K., Land, M., Hauser, L., Chang, Y.-J., Jeffries, C.D., Brettin, T., Rohde, M., Göker, M., Bristow, J., Eisen, J.A., Markowitz, V., Hugenholtz, P., Klenk, H.-P., Kyrpides, N.C. Complete genome sequence of Haloterrigena turkmenica type strain (4kT). Standards in Genomic Sciences 2: 107-116, 2010.

CP000875; CP000876; CP000877 — Kiss, H., Nett, M., Domin, N., Martin, K., Maresca, J.A., Copeland, A., Lapidus, A., Lucas, S., Barry, K.W., Glavina del Rio, T., Dalin, E., Tice, H., Pitluck, S., Richardson, P., Bruce, D., Goodwin, L.A., Han, J., Detter, J.C., Schmutz, J., Brettin, T., Land, M., Hauser, L., Kyrpides, N.C., Ivanova, N., Göker, M., Woyke, T., Klenk, H.-P., Bryant, D.A. Complete genome sequence of the filamentous gliding predatory bacterium Herpetosiphon aurantiacus type strain (114-95T). Standards in Genomic Sciences 5: 356-370, 2011.

CP002606 — Huntemann, M., Lu, M., Nolan, M., Lapidus, A., Lucas, S., Hammon, N., Deshpande, S., Cheng, J.-F., Tapia, R., Han, J., Goodwin, L.A., Pitluck, S., Liolios, K., Pagani, I., Ivanova, N., Ovchinnikova, G., Pati, A., Chen, A., Palaniappan, K., Land, M., Hauser, L., Jeffries, C.D., Detter, J.C., Brambilla, E.-M., Rohde, M., Spring, S., Göker, M., Woyke, T., Bristow, J., Eisen, J.A., Markowitz, V., Hugenholtz, P., Kyrpides, N.C., Klenk, H.-P., Mavromatis, K. Complete genome sequence of Hippea maritima type strain (MH2T). Standards in Genomic Sciences 4: 303-311, 2011.

CP001678; CP001679 — Chertkov, O., Brown, P.J., Kysela, D.T., De Pedro, M.A., Lucas, S., Copeland, A., Lapidus, A., Glavina del Rio, T., Tice, H., Bruce, D., Goodwin, L.A., Pitluck, S., Detter, J.C., Han, J., Larimer, F., Chang, Y.-J., Jeffries, C.D., Land, M., Hauser, L., Kyrpides, N.C., Ivanova, N., Ovchinnikova, G., Tindall, B.J., Göker, M., Klenk, H.-P., Brun, Y.V. Complete genome sequence of Hirschia baltica type strain (IFAM 1418T). Standards in Genomic Sciences 5: 287-297, 2011.

CP002221 — Zeytun, A., Sikorski, J., Nolan, M., Lapidus, A., Lucas, S., Han, J., Tice, H., Cheng, J.-F., Tapia, R., Goodwin, L.A., Pitluck, S., Liolios, K., Ivanova, N., Mavromatis, K., Mikhailova, N., Ovchinnikova, G., Pati, A., Chen, A., Palaniappan, K., Ngatchou Djao, O.D., Land, M., Hauser, L., Chang, Y.-J., Jeffries, C.D., Han, J., Detter, J.C., Übler, S., Rohde, M., Tindall, B.J., Göker, M., Wirth, R., Woyke, T., Bristow, J., Eisen, J.A., Markowitz, V., Hugenholtz, P., Klenk, H.-P., Kyrpides, N.C. Complete genome sequence of Hydrogenobacter thermophilus type strain (TK-6). Standards in Genomic Sciences 4: 132-144, 2011.

CP002098 — Göker, M., Lapidus, A., Spring, S., Yasawong, M., Lucas, S., Glavina del Rio, T., Chen, F., Tice, H., Cheng, J.-F., Goodwin, L.A., Pitluck, S., Liolios, K., Ivanova, N., Mavromatis, K., Mikhailova, N., Pati, A., Chen, A., Palaniappan, K., Brambilla, E.-M., Land, M., Hauser, L., Chang, Y.-J., Jeffries, C.D., Brettin, T., Detter, J.C., Han, J., Rohde, M., Sikorski, J., Woyke, T., Bristow, J., Eisen, J.A., Markowitz, V., Hugenholtz, P., Kyrpides, N.C., Klenk, H.-P. Complete genome sequence of Ignisphaera aggregans type strain (AQ1.S1T). Standards in Genomic Sciences 3: 66-75, 2010.

CP002281; CP002282; CP002283 — Sikorski, J., Chertkov, O., Lapidus, A., Nolan, M., Lucas, S., Glavina del Rio, T., Tice, H., Cheng, J.-F., Tapia, R., Han, J., Goodwin, L.A., Pitluck, S., Liolios, K., Ivanova, N., Mavromatis, K., Mikhailova, N., Pati, A., Chen, A., Palaniappan, K., Land, M., Hauser, L., Chang, Y.-J., Jeffries, C.D., Brambilla, E.-M., Yasawong, M.,

Rohde, M., Pukall, R., Spring, S., Göker, M., Woyke, T., Bristow, J., Eisen, J.A., Markowitz, V., Hugenholtz, P., Kyrpides, N.C., Klenk, H.-P. Complete genome sequence of Ilyobacter polytropus type strain (CuHbu1). Standards in Genomic Sciences 3: 304-314, 2010 (doi:10.4056/sigs.1273360).

CP002343 Glavina del Rio, T., Chertkov, O., Yasawong, M., Lucas, S., Deshpande, S., Cheng, J.-F., Detter, J.C., Tapia, R., Han, J., Goodwin, L.A., Pitluck, S., Liolios, K., Ivanova, N., Mavromatis, K., Pati, A., Chen, A., Palaniappan, K., Land, M., Hauser, L., Chang, Y.-J., Jeffries, C.D., Rohde, M., Pukall, R., Sikorski, J., Göker, M., Woyke, T., Bristow, J., Eisen, J.A., Markowitz, V., Hugenholtz, P., Kyrpides, N.C., Klenk, H.-P., Lapidus, A. Complete genome sequence of Intrasporangium calvum type strain (7 KIP). Standards in Genomic Sciences 3: 294-303, 2010 (doi:10.4056/sigs.1263355).

CP002353; CP002354 Göker, M., Cleland, D., Saunders, E., Lapidus, A., Nolan, M., Lucas, S., Hammon, N., Deshpande, S., Cheng, J.-F., Tapia, R., Han, J., Goodwin, L.A., Pitluck, S., Liolios, K., Pagani, I., Ivanova, N., Mavromatis, K., Pati, A., Chen, A., Palaniappan, K., Land, M., Hauser, L., Chang, Y.-J., Jeffries, C.D., Detter, J.C., Beck, B., Woyke, T., Bristow, J., Eisen, J.A., Markowitz, V., Hugenholtz, P., Kyrpides, N.C., Klenk, H.-P. Complete genome sequence of Isosphaera pallida type strain (IS1BT). Standards in Genomic Sciences 4: 63-71, 2011.

CP001706 Pukall, R., Gehrich-Schröter, G., Lapidus, A., Nolan, M., Glavina del Rio, T., Lucas, S., Chen, F., Tice, H., Pitluck, S., Cheng, J.-F., Copeland, A., Saunders, E., Brettin, T., Detter, J.C., Bruce, D., Goodwin, L.A., Pati, A., Ivanova, N., Mavromatis, K., Ovchinnikova, G., Chen, A., Palaniappan, K., Land, M., Hauser, L., Chang, Y.-J., Jeffries, C.D., Chain, P., Göker, M., Bristow, J., Eisen, J.A., Markowitz, V., Hugenholtz, P., Kyrpides, N.C., Klenk, H.-P., Han, J. Complete genome sequence of Jonesia denitrificans type strain (Prevot 55134T). Standards in Genomic Sciences 1: 262-269, 2009.

CP001707 Han, J., Sikorski, J., Lapidus, A., Nolan, M., Glavina del Rio, T., Tice, H., Cheng, J.-F., Lucas, S., Chen, F., Copeland, A., Ivanova, N., Mavromatis, K., Ovchinnikova, G., Pati, A., Bruce, D., Goodwin, L.A., Pitluck, S., Chen, A., Palaniappan, K., Land, M., Hauser, L., Chang, Y.-J., Jeffries, C.D., Chain, P., Saunders, E., Brettin, T., Göker, M., Tindall, B.J., Bristow, J., Eisen, J.A., Markowitz, V., Hugenholtz, P., Kyrpides, N.C., Klenk, H.-P., Detter, J.C. Complete genome sequence of Kangiella koreensis type strain (SW-125T). Standards in Genomic Sciences 1: 226-233, 2009.

CP001736 Pukall, R., Lapidus, A., Glavina del Rio, T., Copeland, A., Tice, H., Cheng, J.-F., Lucas, S., Chen, F., Nolan, M., LaButti, K., Pati, A., Ivanova, N., Mavromatis, K., Mikhailova, N., Pitluck, S., Bruce, D., Goodwin, L.A., Land, M., Hauser, L., Chang, Y.-J., Jeffries, C.D., Chen, A., Palaniappan, K., Chain, P., Rohde, M., Göker, M., Bristow, J., Eisen, J.A., Markowitz, V., Hugenholtz, P., Kyrpides, N.C., Klenk, H.-P., Brettin, T. Complete genome sequence of Kribbella flavida type strain (IFO 14399T). Standards in Genomic Sciences 2: 186-193, 2010.

CP001686 Sims, D., Brettin, T., Detter, J.C., Han, J., Lapidus, A., Copeland, A., Glavina del Rio, T., Nolan, M., Chen, F., Lucas, S., Tice, H., Cheng, J.-F., Bruce, D., Goodwin, L.A., Pitluck, S., Ovchinnikova, G., Pati, A., Ivanova, N., Mavromatis, K., Chen, A., Palaniappan, K., D'haeseleer, P., Chain, P., Bristow, J., Eisen, J.A., Markowitz, V., Hugenholtz, P., Schneider, S., Göker, M., Pukall, R., Kyrpides, N.C., Klenk, H.-P. Complete genome sequence of Kytococcus sedentarius type strain (strain 541T). Standards in Genomic Sciences 1: 12-20, 2009.

CP001685 Ivanova, N., Gronow, S., Lapidus, A., Copeland, A., Glavina del Rio, T., Nolan, M.,

Lucas, S., Chen, F., Tice, H., Cheng, J.-F., Saunders, E., Bruce, D., Goodwin, L.A., Brettin, T., Detter, J.C., Han, J., Pitluck, S., Mikhailova, N., Pati, A., Mavromatis, K., Chen, A., Land, M., Hauser, L., Chang, Y.-J., Jeffries, C.D., Chain, P., Rohde, C., Göker, M., Bristow, J., Eisen, J.A., Markowitz, V., Hugenholtz, P., Kyrpides, N.C., Klenk, H.-P. Complete genome sequence of Leptotrichia buccalis type strain (C-1013-bT). Standards in Genomic Sciences 1: 126-132, 2009.

CP002360 Sikorski, J., Teshima, H., Nolan, M., Lucas, S., Hammon, N., Deshpande, S., Cheng, J.-F., Pitluck, S., Liolios, K., Pagani, I., Ivanova, N., Huntemann, M., Mavromatis, K., Ovchinnikova, G., Pati, A., Tapia, R., Han, J., Goodwin, L.A., Chen, A., Palaniappan, K., Land, M., Hauser, L., Ngatchou Djao, O.D., Rohde, M., Pukall, R., Spring, S., Abt, B., Göker, M., Detter, J.C., Woyke, T., Bristow, J., Markowitz, V., Hugenholtz, P., Eisen, J.A., Kyrpides, N.C., Klenk, H.-P., Lapidus, A. Complete genome sequence of Mahella australiensis type strain (50-1 BONT). Standards in Genomic Sciences 4: 331-341, 2011.

CP002630 Copeland, A., Gu, W., Yasawong, M., Lapidus, A., Lucas, S., Deshpande, S., Pagani, I., Tapia, R., Cheng, J.-F., Goodwin, L.A., Pitluck, S., Liolios, K., Ivanova, N., Mavromatis, K., Mikhailova, N., Pati, A., Chen, A., Palaniappan, K., Land, M., Pan, C., Brambilla, E.-M., Rohde, M., Tindall, B.J., Sikorski, J., Göker, M., Detter, J.C., Bristow, J., Eisen, J.A., Markowitz, V., Hugenholtz, P., Kyrpides, N.C., Klenk, H.-P., Woyke, T. Complete genome sequence of the aerobic, heterotroph Marinithermus hydrothermalis type strain (T1T) from a deep-sea hydrothermal vent chimney. Standards in Genomic Sciences 6: 21-30, 2012.

CP002349; CP002350 Pagani, I., Chertkov, O., Lapidus, A., Lucas, S., Glavina del Rio, T., Tice, H., Copeland, A., Cheng, J.-F., Nolan, M., Saunders, E., Pitluck, S., Held, B., Goodwin, L.A., Liolios, K., Ovchinnikova, G., Ivanova, N., Mavromatis, K., Pati, A., Chen, A., Palaniappan, K., Land, M., Hauser, L., Chang, Y.-J., Jeffries, C.D., Detter, J.C., Han, J., Tapia, R., Ngatchou Djao, O.D., Rohde, M., Göker, M., Spring, S., Woyke, T., Bristow, J., Eisen, J.A., Markowitz, V., Hugenholtz, P., Kyrpides, N.C., Klenk, H.-P. Complete genome sequence of Marivirga tractuosa type strain (H-43T). Standards in Genomic Sciences 4: 155-163, 2011.

CP001743 Tindall, B.J., Sikorski, J., Lucas, S., Glavina del Rio, T., Chen, F., Tice, H., Cheng, J.-F., Goodwin, L.A., Pitluck, S., Liolios, K., Ivanova, N., Mavromatis, K., Mikhailova, N., Ovchinnikova, G., Pati, A., Fähnrich, R., Chen, A., Palaniappan, K., Land, M., Hauser, L., Chang, Y.-J., Jeffries, C.D., Goltsmann, E., Rohde, M., Göker, M., Woyke, T., Bristow, J., Eisen, J.A., Markowitz, V., Hugenholtz, P., Kyrpides, N.C., Klenk, H.-P., Lapidus, A. Complete genome sequence of Meiothermus ruber type strain (21T). Standards in Genomic Sciences 3: 26-36, 2010.

CP002042; CP002043; CP002044 Sikorski, J., Tindall, B.J., Lucas, S., Copeland, A., Glavina del Rio, T., Nolan, M., Tice, H., Cheng, J.-F., Han, J., Pitluck, S., Liolios, K., Ivanova, N., Mavromatis, K., Mikhailova, N., Ovchinnikova, G., Pati, A., Goodwin, L.A., Chen, A., Palaniappan, K., Land, M., Hauser, L., Chang, Y.-J., Jeffries, C.D., Rohde, M., Göker, M., Woyke, T., Bristow, J., Eisen, J.A., Markowitz, V., Hugenholtz, P., Kyrpides, N.C., Klenk, H.-P., Lapidus, A. Complete genome sequence of Meiothermus silvanus type strain (VI-R2T). Standards in Genomic Sciences 3: 37-46, 2010.

CP002278 Anderson, I.J., Ngatchou Djao, O.D., Misra, M., Chertkov, O., Nolan, M., Lucas, S., Lapidus, A., Glavina del Rio, T., Tice, H., Cheng, J.-F., Tapia, R., Han, J., Goodwin, L.A., Pitluck, S., Liolios, K., Ivanova, N., Mavromatis, K., Mikhailova, N., Pati, A., Brambilla, E.-M., Chen, A., Palaniappan, K., Land, M., Hauser, L., Chang, Y.-J., Jeffries, C.D., Sikorski, J., Spring, S., Rohde, M., Eichinger, K., Huber, H., Wirth, R., Göker, M., Detter, J.C., Woyke, T., Bristow, J., Eisen, J.A., Markowitz, V.,

Hugenholtz, P., Klenk, H.-P., Kyrpides, N.C. Complete genome sequence of Methanothermus fervidus type strain (V24S). Standards in Genomic Sciences 3: 315-324, 2010 (doi:10.4056/sigs.1283367).

CP002999 Huntemann, M., Teshima, H., Lapidus, A., Nolan, M., Lucas, S., Hammon, N., Deshpande, S., Cheng, J.-F., Tapia, R., Goodwin, L.A., Pitluck, S., Liolios, K., Pagani, I., Ivanova, N., Mavromatis, K., Mikhailova, N., Pati, A., Chen, A., Palaniappan, K., Land, M., Hauser, L., Pan, C., Brambilla, E.-M., Rohde, M., Spring, S., Göker, M., Detter, J.C., Bristow, J., Eisen, J.A., Markowitz, V., Hugenholtz, P., Kyrpides, N.C., Klenk, H.-P., Woyke, T. Complete genome sequence of the facultatively anaerobic, appendaged bacterium Muricauda ruestringensis type strain (B1T). Standards in Genomic Sciences 6: 185-193, 2012.

CP001737 Tice, H., Mayilraj, S., Sims, D., Lapidus, A., Nolan, M., Lucas, S., Glavina del Rio, T., Copeland, A., Cheng, J.-F., Meinecke, L., Bruce, D., Goodwin, L.A., Pitluck, S., Ivanova, N., Mavromatis, K., Ovchinnikova, G., Pati, A., Chen, A., Palaniappan, K., Land, M., Hauser, L., Chang, Y.-J., Jeffries, C.D., Detter, J.C., Brettin, T., Rohde, M., Göker, M., Bristow, J., Eisen, J.A., Markowitz, V., Hugenholtz, P., Kyrpides, N.C., Klenk, H.-P., Chen, F. Complete genome sequence of Nakamurella multipartita type strain (Y-104T). Standards in Genomic Sciences 2: 168-175, 2010.

CP002452 Anderson, I.J., Sikorski, J., Zeytun, A., Nolan, M., Lapidus, A., Lucas, S., Hammon, N., Deshpande, S., Cheng, J.-F., Tapia, R., Han, J., Goodwin, L.A., Pitluck, S., Liolios, K., Pagani, I., Ivanova, N., Huntemann, M., Mavromatis, K., Ovchinnikova, G., Pati, A., Chen, A., Palaniappan, K., Land, M., Hauser, L., Brambilla, E.-M., Ngatchou Djao, O.D., Rohde, M., Tindall, B.J., Göker, M., Detter, J.C., Woyke, T., Bristow, J., Eisen, J.A., Markowitz, V., Hugenholtz, P., Klenk, H.-P., Kyrpides, N.C. Complete genome sequence of Nitratifractor salsuginis type strain (E9I37-1T). Standards in Genomic Sciences 4: 322-330, 2011.

CP002361; CP002362 Pati, A., Zhang, X., Lapidus, A., Nolan, M., Lucas, S., Glavina del Rio, T., Tice, H., Cheng, J.-F., Tapia, R., Han, J., Goodwin, L.A., Pitluck, S., Liolios, K., Pagani, I., Ivanova, N., Mavromatis, K., Chen, A., Palaniappan, K., Hauser, L., Jeffries, C.D., Brambilla, E.-M., Röhl, A., Mwirichia, R., Rohde, M., Tindall, B.J., Sikorski, J., Wirth, R., Göker, M., Woyke, T., Detter, J.C., Bristow, J., Eisen, J.A., Markowitz, V., Hugenholtz, P., Kyrpides, N.C., Klenk, H.-P., Land, M. Complete genome sequence of Oceanithermus profundus type strain (506T). Standards in Genomic Sciences 4: 210-220, 2011.

CP002544 Göker, M., Gronow, S., Zeytun, A., Nolan, M., Lucas, S., Lapidus, A., Hammon, N., Deshpande, S., Cheng, J.-F., Pitluck, S., Liolios, K., Pagani, I., Ivanova, N., Mavromatis, K., Ovchinnikova, G., Pati, A., Tapia, R., Han, J., Goodwin, L.A., Chen, A., Palaniappan, K., Land, M., Hauser, L., Jeffries, C.D., Brambilla, E.-M., Rohde, M., Detter, J.C., Woyke, T., Bristow, J., Markowitz, V., Hugenholtz, P., Eisen, J.A., Kyrpides, N.C., Klenk, H.-P. Complete genome sequence of Odoribacter splanchnicus type strain (1651/6T). Standards in Genomic Sciences 4: 200-209, 2011.

CP002106 Göker, M., Lucas, S., Yasawong, M., Glavina del Rio, T., Chen, F., Tice, H., Cheng, J.-F., Goodwin, L.A., Pitluck, S., Liolios, K., Ivanova, N., Mavromatis, K., Mikhailova, N., Pati, A., Chen, A., Palaniappan, K., Land, M., Hauser, L., Chang, Y.-J., Jeffries, C.D., Rohde, M., Sikorski, J., Pukall, R., Woyke, T., Bristow, J., Eisen, J.A., Markowitz, V., Hugenholtz, P., Kyrpides, N.C., Klenk, H.-P., Lapidus, A. Complete genome sequence of Olsenella uli type strain (VPI D76D-27CT). Standards in Genomic Sciences 3: 76-84, 2010.

CP002345 Gronow, S., Munk, A.C., Lapidus, A., Nolan, M., Lucas, S., Hammon, N., Deshpande, S., Cheng, J.-F., Tapia, R., Han, J., Goodwin, L.A., Pitluck, S., Liolios, K., Ivanova, N., Mavromatis, K., Mikhailova, N., Pati, A., Chen, A., Palaniappan, K., Land, M., Hauser, L., Chang, Y.-J., Jeffries, C.D., Brambilla, E.-M., Rohde, M., Göker, M., Detter, J.C., Woyke, T., Bristow, J., Eisen, J.A., Markowitz, V., Hugenholtz, P., Kyrpides, N.C., Klenk, H.-P. Complete genome sequence of Paludibacter propionicigenes type strain (WB4T). Standards in Genomic Sciences 4: 36-44, 2011.

CP001681 Han, J., Spring, S., Lapidus, A., Glavina del Rio, T., Tice, H., Copeland, A., Cheng, J.-F., Lucas, S., Chen, F., Nolan, M., Bruce, D., Goodwin, L.A., Pitluck, S., Ivanova, N., Mavromatis, K., Mikhailova, N., Pati, A., Chen, A., Palaniappan, K., Land, M., Hauser, L., Chang, Y.-J., Jeffries, C.D., Saunders, E., Chertkov, O., Brettin, T., Göker, M., Rohde, M., Bristow, J., Eisen, J.A., Markowitz, V., Hugenholtz, P., Kyrpides, N.C., Klenk, H.-P., Detter, J.C. Complete genome sequence of Pedobacter heparinus type strain (HIM 762-3T). Standards in Genomic Sciences 1: 54-62, 2009.

CP001848 Clum, A., Tindall, B.J., Sikorski, J., Lucas, S., Glavina del Rio, T., Nolan, M., Chen, F., Tice, H., Pitluck, S., Cheng, J.-F., Chertkov, O., Brettin, T., Han, J., Detter, J.C., Kuske, C., Bruce, D., Goodwin, L.A., Ovchinnikova, G., Pati, A., Ivanova, N., Mavromatis, K., Mikhailova, N., Chen, A., Palaniappan, K., Land, M., Hauser, L., Chang, Y.-J., Jeffries, C.D., Chain, P., Rohde, M., Göker, M., Bristow, J., Eisen, J.A., Markowitz, V., Hugenholtz, P., Kyrpides, N.C., Klenk, H.-P., Lapidus, A. Complete genome sequence of Pirellula staleyi type strain (ATCC 27377T). Standards in Genomic Sciences 1: 308-316, 2009.

CP002838 Göker, M., Nolan, M., Lucas, S., Hammon, N., Deshpande, S., Cheng, J.-F., Tapia, R., Han, J., Goodwin, L.A., Pitluck, S., Huntemann, M., Liolios, K., Ivanova, N., Pagani, I., Mavromatis, K., Ovchinnikova, G., Pati, A., Chen, A., Palaniappan, K., Land, M., Hauser, L., Brambilla, E.-M., Huber, H., Yasawong, M., Rohde, M., Spring, S., Abt, B., Sikorski, J., Wirth, R., Detter, J.C., Woyke, T., Bristow, J., Eisen, J.A., Markowitz, V., Hugenholtz, P., Kyrpides, N.C., Klenk, H.-P., Lapidus, A. Complete genome sequence of Pyrolobus fumarii type strain (1AT). Standards in Genomic Sciences 4: 381-392, 2011.

CP000230 Munk, A.C., Copeland, A., Lucas, S., Lapidus, A., Glavina del Rio, T., Barry, K.W., Detter, J.C., Hammon, N., Israni, S., Pitluck, S., Brettin, T., Bruce, D., Han, J., Tapia, R., Gilna, P., Schmutz, J., Larimer, F., Land, M., Kyrpides, N.C., Mavromatis, K., Richardson, P., Rohde, M., Göker, M., Klenk, H.-P., Zhang, X., Roberts, G.P., Reslewic, S., Schwartz, D.C. Complete genome sequence of Rhodospirillum rubrum type strain (S1T). Standards in Genomic Sciences 4: 293-302, 2011.

CP001807; CP001808 Nolan, M., Tindall, B.J., Pomrenke, H., Lapidus, A., Copeland, A., Glavina del Rio, T., Lucas, S., Chen, F., Tice, H., Cheng, J.-F., Saunders, E., Han, J., Bruce, D., Goodwin, L.A., Chain, P., Pitluck, S., Pati, A., Ivanova, N., Mavromatis, K., Chen, A., Palaniappan, K., Land, M., Hauser, L., Chang, Y.-J., Jeffries, C.D., Brettin, T., Göker, M., Bristow, J., Eisen, J.A., Markowitz, V., Hugenholtz, P., Kyrpides, N.C., Klenk, H.-P., Detter, J.C. Complete genome sequence of Rhodothermus marinus type strain (R-10T). Standards in Genomic Sciences 1: 283-291, 2009.

CP002346 Mavromatis, K., Lu, M., Misra, M., Lapidus, A., Nolan, M., Lucas, S., Hammon, N., Deshpande, S., Cheng, J.-F., Tapia, R., Han, J., Goodwin, L.A., Pitluck, S., Liolios, K., Pagani, I., Ivanova, N., Mikhailova, N., Pati, A., Chen, A., Palaniappan, K., Land, M., Hauser, L., Chang, Y.-J., Jeffries, C.D., Detter, J.C., Brambilla, E.-M., Rohde, M., Göker, M., Gronow, S., Woyke, T., Bristow, J., Eisen, J.A., Markowitz, V., Hugenholtz, P., Klenk, H.-P., Kyrpides, N.C. Complete genome sequence of

	Riemerella anatipestifer type strain (ATCC 11845T). Standards in Genomic Sciences 4: 145-155, 2011.
CP002859; CP002860; CP002861; CP002862; CP002863; CP002864	Copeland, A., Zhang, X., Misra, M., Lapidus, A., Nolan, M., Lucas, S., Deshpande, S., Cheng, J.-F., Tapia, R., Goodwin, L.A., Pitluck, S., Liolios, K., Pagani, I., Ivanova, N., Mikhailova, N., Pati, A., Chen, A., Palaniappan, K., Land, M., Hauser, L., Pan, C., Jeffries, C.D., Detter, J.C., Brambilla, E.-M., Rohde, M., Ngatchou Djao, O.D., Göker, M., Sikorski, J., Tindall, B.J., Woyke, T., Bristow, J., Eisen, J.A., Markowitz, V., Hugenholtz, P., Kyrpides, N.C., Klenk, H.-P., Mavromatis, K. Complete genome sequence of the aquatic bacterium Runella slithyformis type strain (LSU 4T). Standards in Genomic Sciences 6: 145-154, 2012.
CP001683	Pati, A., Sikorski, J., Nolan, M., Lapidus, A., Copeland, A., Glavina del Rio, T., Lucas, S., Chen, F., Tice, H., Pitluck, S., Cheng, J.-F., Chertkov, O., Brettin, T., Han, J., Detter, J.C., Kuske, C., Bruce, D., Goodwin, L.A., Chain, P., D'haeseleer, P., Chen, A., Ivanova, N., Mavromatis, K., Mikhailova, N., Rohde, M., Tindall, B.J., Göker, M., Bristow, J., Eisen, J.A., Markowitz, V., Hugenholtz, P., Kyrpides, N.C., Klenk, H.-P. Complete genome sequence of Saccharomonospora viridis type strain (P101T). Standards in Genomic Sciences 1: 141-149, 2009.
CP001819	Ivanova, N., Sikorski, J., Sims, D., Brettin, T., Detter, J.C., Han, J., Lapidus, A., Copeland, A., Glavina del Rio, T., Nolan, M., Chen, F., Lucas, S., Tice, H., Cheng, J.-F., Bruce, D., Goodwin, L.A., Pitluck, S., Pati, A., Mavromatis, K., Chen, A., Palaniappan, K., D'haeseleer, P., Chain, P., Bristow, J., Eisen, J.A., Markowitz, V., Hugenholtz, P., Göker, M., Pukall, R., Klenk, H.-P., Kyrpides, N.C. Complete genome sequence of Sanguibacter keddieii type strain (ST-74T). Standards in Genomic Sciences 1: 110-118, 2009.
CP001739; CP001740; CP001741	Harmon-Smith, M., Celia, L., Chertkov, O., Lapidus, A., Copeland, A., Nolan, M., Lucas, S., Tice, H., Cheng, J.-F., Han, J., Detter, J.C., Bruce, D., Goodwin, L.A., Pitluck, S., Pati, A., Liolios, K., Ivanova, N., Mavromatis, K., Mikhailova, N., Chen, A., Palaniappan, K., Land, M., Hauser, L., Chang, Y.-J., Jeffries, C.D., Brettin, T., Göker, M., Beck, B., Bristow, J., Eisen, J.A., Markowitz, V., Hugenholtz, P., Kyrpides, N.C., Klenk, H.-P., Chen, F. Complete genome sequence of Sebaldella termitidis type strain (NCTC 11300T). Standards in Genomic Sciences 2: 220-227, 2010.
CP001958	Sikorski, J., Lapidus, A., Copeland, A., Misra, M., Glavina del Rio, T., Nolan, M., Lucas, S., Chen, F., Tice, H., Cheng, J.-F., Jando, M., Schneider, S., Bruce, D., Goodwin, L.A., Pitluck, S., Liolios, K., Mikhailova, N., Pati, A., Ivanova, N., Mavromatis, K., Chen, A., Palaniappan, K., Chertkov, O., Land, M., Hauser, L., Chang, Y.-J., Jeffries, C.D., Brettin, T., Detter, J.C., Han, J., Rohde, M., Göker, M., Bristow, J., Eisen, J.A., Markowitz, V., Hugenholtz, P., Kyrpides, N.C., Klenk, H.-P. Complete genome sequence of Segniliparus rotundus type strain (CDC 1076T). Standards in Genomic Sciences 2: 203-211, 2010.
CP001684	Pukall, R., Lapidus, A., Nolan, M., Copeland, A., Glavina del Rio, T., Lucas, S., Chen, F., Tice, H., Cheng, J.-F., Chertkov, O., Bruce, D., Goodwin, L.A., Kuske, C., Brettin, T., Detter, J.C., Han, J., Pitluck, S., Pati, A., Mavromatis, K., Ivanova, N., Ovchinnikova, G., Chen, A., Palaniappan, K., Schneider, S., Rohde, M., Chain, P., D'haeseleer, P., Göker, M., Bristow, J., Eisen, J.A., Markowitz, V., Kyrpides, N.C., Klenk, H.-P., Hugenholtz, P. Complete genome sequence of Slackia heliotrinireducens type strain (RSH 1T). Standards in Genomic Sciences 1: 234-241, 2009.
CP001823;	Pati, A., LaButti, K., Pukall, R., Nolan, M., Glavina del Rio, T., Tice, H., Cheng, J.-

CP001824	F., Lucas, S., Chen, F., Copeland, A., Ivanova, N., Mavromatis, K., Mikhailova, N., Pitluck, S., Bruce, D., Goodwin, L.A., Land, M., Hauser, L., Chang, Y.-J., Jeffries, C.D., Chen, A., Palaniappan, K., Chain, P., Brettin, T., Sikorski, J., Rohde, M., Göker, M., Bristow, J., Eisen, J.A., Markowitz, V., Hugenholtz, P., Kyrpides, N.C., Klenk, H.-P., Lapidus, A. Complete genome sequence of Sphaerobacter thermophilus type strain (S 6022T). Standards in Genomic Sciences 2: 49-56, 2010.
CP003282	Liolios, K., Abt, B., Scheuner, C., Teshima, H., Held, B., Lapidus, A., Nolan, M., Lucas, S., Deshpande, S., Cheng, J.F., Tapia, R., Goodwin, L.A., Pitluck, S., Pagani, I., Ivanova, N., Mavromatis, K., Mikhailova, N., Huntemann, M., Pati, A., Chen, A., Palaniappan, K., Land, M., Rohde, M., Tindall, B.J., Detter, J.C., Göker, M., Bristow, J., Eisen, J.A., Markowitz, V., Hugenholtz, P., Woyke, T., Klenk, H.P., Klenk, H.P., Kyrpides, N.C. Complete genome sequence of the halophilic bacterium Spirochaeta africana type strain (Z-7692T) from the alkaline Lake Magadi in the East African Rift. Standards in Genomic Sciences 8: 165-176, 2013 (doi:10.4056/sigs.3607108).
CP002116	Mavromatis, K., Yasawong, M., Chertkov, O., Lapidus, A., Lucas, S., Nolan, M., Glavina del Rio, T., Tice, H., Cheng, J.-F., Pitluck, S., Liolios, K., Ivanova, N., Tapia, R., Han, J., Bruce, D., Goodwin, L.A., Pati, A., Chen, A., Palaniappan, K., Land, M., Hauser, L., Chang, Y.-J., Jeffries, C.D., Detter, J.C., Rohde, M., Brambilla, E.-M., Spring, S., Göker, M., Sikorski, J., Woyke, T., Bristow, J., Eisen, J.A., Markowitz, V., Hugenholtz, P., Klenk, H.-P., Kyrpides, N.C. Complete genome sequence of Spirochaeta smaragdinae type strain (SEBR 4228T). Standards in Genomic Sciences 3: 136-144, 2010.
CP001769; CP001770; CP001771; CP001772; CP001773; CP001774; CP001775; CP001776; CP001777	Lail, K., Sikorski, J., Saunders, E., Lapidus, A., Glavina del Rio, T., Copeland, A., Tice, H., Cheng, J.-F., Lucas, S., Nolan, M., Bruce, D., Goodwin, L.A., Pitluck, S., Ivanova, N., Mavromatis, K., Ovchinnikova, G., Pati, A., Chen, A., Palaniappan, K., Land, M., Hauser, L., Chang, Y.-J., Jeffries, C.D., Chain, P., Brettin, T., Detter, J.C., Schütze, A., Rohde, M., Tindall, B.J., Göker, M., Bristow, J., Eisen, J.A., Markowitz, V., Hugenholtz, P., Kyrpides, N.C., Klenk, H.-P., Chen, F. Complete genome sequence of Spirosoma linguale type strain (1T). Standards in Genomic Sciences 2: 176-185, 2010.
CP001778	Munk, A.C., Lapidus, A., Copeland, A., Jando, M., Mayilraj, S., Glavina del Rio, T., Nolan, M., Chen, F., Lucas, S., Tice, H., Cheng, J.-F., Han, J., Detter, J.C., Bruce, D., Goodwin, L.A., Chain, P., Pitluck, S., Göker, M., Ovchinnikova, G., Pati, A., Ivanova, N., Mavromatis, K., Chen, A., Palaniappan, K., Land, M., Hauser, L., Chang, Y.-J., Jeffries, C.D., Bristow, J., Eisen, J.A., Markowitz, V., Hugenholtz, P., Kyrpides, N.C., Klenk, H.-P. Complete genome sequence of Stackebrandtia nassauensis type strain (LLR-40K-21T). Standards in Genomic Sciences 1: 292-299, 2009.
CP002026	Kappler, U., Davenport, K., Beatson, S., Lucas, S., Lapidus, A., Copeland, A., Berry, K.W., Rio, T.G.D., Hammon, N., Dalin, E., Tice, H., Pitluck, S., Richardson, P., Bruce, D., Goodwin, L.A., Han, C., Tapia, R., Detter, J.C., Chang, Y-J., Jeffries, C.D., Land, M., Hauser, L., Kyrpides, N.C., Göker, M., Ivanova, N., Klenk, H.-P., Woyke, T. Complete genome sequence of the facultatively chemolithoautotrophic and methylotrophic alpha Proteobacterium Starkeya novella type strain (ATCC 8093T). Standards in Genomic Sciences Stand Genomic 7: 44-58, 2012 (doi:10.4056/sigs.3006378).
CP001779	Nolan, M., Gronow, S., Lapidus, A., Ivanova, N., Copeland, A., Lucas, S., Glavina del Rio, T., Chen, F., Tice, H., Pitluck, S., Cheng, J.-F., Sims, D., Meinecke, L.,

	Bruce, D., Goodwin, L.A., Brettin, T., Han, J., Detter, J.C., Ovchinnikova, G., Pati, A., Mavromatis, K., Mikhailova, N., Chen, A., Palaniappan, K., Land, M., Hauser, L., Chang, Y.-J., Jeffries, C.D., Rohde, M., Spröer, C., Göker, M., Bristow, J., Eisen, J.A., Markowitz, V., Hugenholtz, P., Kyrpides, N.C., Klenk, H.-P., Chain, P. Complete genome sequence of Streptobacillus moniliformis type strain (9901T). Standards in Genomic Sciences 2: 300-307, 2009.
CP001814; CP001815	Nolan, M., Sikorski, J., Jando, M., Lucas, S., Lapidus, A., Glavina del Rio, T., Chen, F., Tice, H., Pitluck, S., Cheng, J.-F., Chertkov, O., Sims, D., Meinecke, L., Brettin, T., Han, J., Detter, J.C., Bruce, D., Goodwin, L.A., Land, M., Hauser, L., Chang, Y.-J., Jeffries, C.D., Ivanova, N., Mavromatis, K., Mikhailova, N., Chen, A., Palaniappan, K., Chain, P., Rohde, M., Göker, M., Bristow, J., Eisen, J.A., Markowitz, V., Hugenholtz, P., Kyrpides, N.C., Klenk, H.-P. Complete genome sequence of Streptosporangium roseum type strain (NI 9100T). Standards in Genomic Sciences 2: 29-37, 2010.
CP003179; CP003180	Anderson, I.J., Chertkov, O., Chen, A., Saunders, E., Lapidus, A., Nolan, M., Lucas, S., Hammon, N., Deshpande, S., Cheng, J.-F., Han, J., Tapia, R., Goodwin, L.A., Pitluck, S., Liolios, K., Pagani, I., Ivanova, N., Mikhailova, N., Pati, A., Palaniappan, K., Land, M., Pan, C., Rohde, M., Pukall, R., Göker, M., Detter, J.C., Woyke, T., Bristow, J., Eisen, J.A., Markowitz, V., Hugenholtz, P., Kyrpides, N.C., Klenk, H.-P., Mavromatis, K. Complete genome sequence of the moderately thermophilic mineral-sulfide-oxidizing firmicute Sulfobacillus acidophilus type strain (NALT). Standards in Genomic Sciences 6: 293-303, 2012.
CP002355; CP002356; CP002357; CP002358; CP002359	Han, J., Kotsyurbenko, O., Chertkov, O., Held, B., Lapidus, A., Nolan, M., Lucas, S., Hammon, N., Deshpande, S., Cheng, J.-F., Tapia, R., Goodwin, L.A., Pitluck, S., Liolios, K., Pagani, I., Ivanova, N., Mavromatis, K., Mikhailova, N., Pati, A., Chen, A., Palaniappan, K., Land, M., Hauser, L., Chang, Y.-J., Jeffries, C.D., Brambilla, E.-M., Rohde, M., Spring, S., Sikorski, J., Göker, M., Woyke, T., Bristow, J., Eisen, J.A., Markowitz, V., Hugenholtz, P., Kyrpides, N.C., Klenk, H.-P., Detter, J.C. Complete genome sequence of the sulfur compounds oxidizing chemolithoautotroph Sulfuricurvum kujiense type strain (YK-1T). Standards in Genomic Sciences 6: 94-103, 2012.
CP002205	Sikorski, J., Munk, A.C., Lapidus, A., Ngatchou Djao, O.D., Lucas, S., Glavina del Rio, T., Nolan, M., Tice, H., Han, J., Cheng, J.-F., Tapia, R., Goodwin, L.A., Pitluck, S., Liolios, K., Ivanova, N., Mavromatis, K., Mikhailova, N., Pati, A., Sims, D., Meinecke, L., Brettin, T., Detter, J.C., Chen, A., Palaniappan, K., Land, M., Hauser, L., Chang, Y.-J., Jeffries, C.D., Rohde, M., Lang, E., Spring, S., Göker, M., Woyke, T., Bristow, J., Eisen, J.A., Markowitz, V., Hugenholtz, P., Kyrpides, N.C., Klenk, H.-P. Complete genome sequence of Sulfurimonas autotrophica type strain (OK10T). Standards in Genomic Sciences 3: 183-191, 2010.
CP001816	Sikorski, J., Lapidus, A., Copeland, A., Glavina del Rio, T., Nolan, M., Lucas, S., Chen, F., Tice, H., Cheng, J.-F., Saunders, E., Bruce, D., Goodwin, L.A., Pitluck, S., Mikhailova, N., Pati, A., Ivanova, N., Mavromatis, K., Chen, A., Palaniappan, K., Chain, P., Land, M., Hauser, L., Chang, Y.-J., Jeffries, C.D., Brettin, T., Detter, J.C., Han, J., Rohde, M., Lang, E., Spring, S., Göker, M., Bristow, J., Eisen, J.A., Markowitz, V., Hugenholtz, P., Kyrpides, N.C., Klenk, H.-P. Complete genome sequence of Sulfurospirillum deleyianum type strain (5175T). Standards in Genomic Sciences 2: 149-157, 2010.
CP002547	Han, J., Mwirichia, R., Chertkov, O., Held, B., Lapidus, A., Nolan, M., Lucas, S., Hammon, N., Deshpande, S., Cheng, J.-F., Tapia, R., Goodwin, L.A., Pitluck, S., Huntemann, M., Liolios, K., Ivanova, N., Pagani, I., Mavromatis, K., Ovchinnikova,

G., Pati, A., Chen, A., Palaniappan, K., Land, M., Hauser, L., Brambilla, E.-M., Rohde, M., Spring, S., Sikorski, J., Göker, M., Woyke, T., Bristow, J., Eisen, J.A., Markowitz, V., Hugenholtz, P., Kyrpides, N.C., Klenk, H.-P., Detter, J.C. Complete genome sequence of Syntrophobotulus glycolicus type strain (FlGlyRT). Standards in Genomic Sciences 4: 371-380, 2011.

CP002048 Ngatchou Djao, O.D., Zhang, X., Lucas, S., Lapidus, A., Glavina del Rio, T., Nolan, M., Tice, H., Cheng, J.-F., Han, J., Tapia, R., Goodwin, L.A., Pitluck, S., Liolios, K., Ivanova, N., Mavromatis, K., Mikhailova, N., Ovchinnikova, G., Pati, A., Brambilla, E.-M., Chen, A., Palaniappan, K., Land, M., Hauser, L., Chang, Y.-J., Jeffries, C.D., Rohde, M., Sikorski, J., Spring, S., Göker, M., Detter, J.C., Woyke, T., Bristow, J., Eisen, J.A., Markowitz, V., Hugenholtz, P., Kyrpides, N.C., Klenk, H.-P. Complete genome sequence of Syntrophothermus lipocalidus type strain (TGB-C1T). Standards in Genomic Sciences 3: 267-275, 2010 (doi:10.4056/sigs.1233249).

CP002344 Han, J., Gu, W., Zhang, X., Lapidus, A., Nolan, M., Copeland, A., Lucas, S., Glavina del Rio, T., Tice, H., Cheng, J.-F., Tapia, R., Goodwin, L.A., Pitluck, S., Pagani, I., Ivanova, N., Mavromatis, K., Mikhailova, N., Pati, A., Chen, A., Palaniappan, K., Land, M., Hauser, L., Chang, Y.-J., Jeffries, C.D., Schneider, S., Rohde, M., Göker, M., Pukall, R., Woyke, T., Bristow, J., Eisen, J.A., Markowitz, V., Hugenholtz, P., Kyrpides, N.C., Klenk, H.-P., Detter, J.C. Complete genome sequence of Thermaerobacter marianensis type strain (7p75a). Standards in Genomic Sciences 3: 337-345, 2010 (doi:10.4056/sigs.1373474).

CP001818 Chovatia, M., Sikorski, J., Schröder, M., Lapidus, A., Nolan, M., Tice, H., Glavina del Rio, T., Copeland, A., Cheng, J.-F., Lucas, S., Chen, F., Bruce, D., Goodwin, L.A., Pitluck, S., Ivanova, N., Mavromatis, K., Ovchinnikova, G., Pati, A., Chen, A., Palaniappan, K., Land, M., Hauser, L., Chang, Y.-J., Jeffries, C.D., Chain, P., Saunders, E., Detter, J.C., Brettin, T., Rohde, M., Göker, M., Spring, S., Bristow, J., Markowitz, V., Hugenholtz, P., Kyrpides, N.C., Klenk, H.-P., Eisen, J.A. Complete genome sequence of Thermanaerovibrio acidaminovorans type strain (Su883T). Standards in Genomic Sciences 1: 254-261, 2009.

CP001874 Liolios, K., Sikorski, J., Jando, M., Lapidus, A., Copeland, A., Glavina del Rio, T., Nolan, M., Lucas, S., Chen, F., Tice, H., Cheng, J.-F., Han, J., Woyke, T., Goodwin, L.A., Pitluck, S., Ivanova, N., Mavromatis, K., Mikhailova, N., Chertkov, O., Kuske, C., Chen, A., Palaniappan, K., Land, M., Hauser, L., Chang, Y.-J., Jeffries, C.D., Detter, J.C., Brettin, T., Chain, P., Rohde, M., Göker, M., Bristow, J., Eisen, J.A., Markowitz, V., Hugenholtz, P., Klenk, H.-P., Kyrpides, N.C. Complete genome sequence of Thermobispora bispora type strain (R51T). Standards in Genomic Sciences 2: 318-326, 2010.

CP001931 Wirth, R., Sikorski, J., Brambilla, E.-M., Misra, M., Lapidus, A., Copeland, A., Nolan, M., Lucas, S., Chen, F., Tice, H., Cheng, J.-F., Han, J., Detter, J.C., Tapia, R., Bruce, D., Goodwin, L.A., Pitluck, S., Pati, A., Anderson, I.J., Ivanova, N., Mavromatis, K., Mikhailova, N., Chen, A., Palaniappan, K., Bilek, Y., Hader, T., Land, M., Hauser, L., Chang, Y.-J., Jeffries, C.D., Tindall, B.J., Rohde, M., Göker, M., Bristow, J., Eisen, J.A., Markowitz, V., Hugenholtz, P., Kyrpides, N.C., Klenk, H.-P. Complete genome sequence of Thermocrinis albus type strain (HI 11/12T). Standards in Genomic Sciences 2: 194-202, 2010.

CP001738 Chertkov, O., Sikorski, J., Nolan, M., Lapidus, A., Lucas, S., Glavina del Rio, T., Tice, H., Cheng, J.-F., Goodwin, L.A., Pitluck, S., Liolios, K., Ivanova, N., Mavromatis, K., Mikhailova, N., Ovchinnikova, G., Pati, A., Chen, A., Palaniappan, K., Ngatchou Djao, O.D., Land, M., Hauser, L., Chang, Y.-J., Jeffries, C.D., Brettin, T., Han, J., Detter, J.C., Rohde, M., Göker, M., Woyke, T., Bristow, J., Eisen, J.A.,

Markowitz, V., Hugenholtz, P., Klenk, H.-P., Kyrpides, N.C. Complete genome sequence of Thermomonospora curvata type strain (B9). Standards in Genomic Sciences 4: 13-22, 2011.

CP002131 Pitluck, S., Yasawong, M., Munk, A.C., Nolan, M., Lapidus, A., Lucas, S., Glavina del Rio, T., Tice, H., Cheng, J.-F., Bruce, D., Detter, J.C., Tapia, R., Han, J., Goodwin, L.A., Liolios, K., Ivanova, N., Mavromatis, K., Mikhailova, N., Pati, A., Chen, A., Palaniappan, K., Land, M., Hauser, L., Chang, Y.-J., Jeffries, C.D., Rohde, M., Spring, S., Sikorski, J., Göker, M., Woyke, T., Bristow, J., Eisen, J.A., Markowitz, V., Hugenholtz, P., Kyrpides, N.C., Klenk, H.-P. Complete genome sequence of Thermosediminibacter oceani type strain (JW/IW-1228PT). Standards in Genomic Sciences 3: 108-116, 2010.

CP001939 Spring, S., Rachel, R., Lapidus, A., Davenport, K., Tice, H., Copeland, A., Cheng, J.-F., Lucas, S., Chen, F., Nolan, M., Bruce, D., Goodwin, L.A., Pitluck, S., Ivanova, N., Mavromatis, K., Ovchinnikova, G., Pati, A., Chen, A., Palaniappan, K., Land, M., Hauser, L., Chang, Y.-J., Jeffries, C.D., Brettin, T., Detter, J.C., Tapia, R., Han, J., Heimerl, T., Weikl, F., Brambilla, E.-M., Göker, M., Bristow, J., Eisen, J.A., Markowitz, V., Hugenholtz, P., Kyrpides, N.C., Klenk, H.-P. Complete genome sequence of Thermosphaera aggregans type strain (M11TLT). Standards in Genomic Sciences 2: 245-259, 2010.

CP002776 Kappler, U., Davenport, K.W., Beatson, S., Lapidus, A., Pan, C., Han, C., Montero-Calasanz, M.d.C., Land, M., Hauser, L., Rhode, M., Göker, M., Ivanova, N., Woyke, T., Klenk, H-P., Kyrpides, N.C. Complete genome sequence of the haloalkaliphilic, obligately chemolithoautotrophic thiosulfate and sulfide-oxidizing γ-proteobacterium Thioalkalimicrobium cyclicum type strain ALM 1 (DSM 14477T). Standards in Genomic Sciences, under review.

CP001616 Chertkov, O., Copeland, A., Lucas, S., Lapidus, A., Barry, K.W., Detter, J.C., Glavina del Rio, T., Hammon, N., Dalin, E., Tice, H., Pitluck, S., Richardson, P., Bruce, D., Goodwin, L.A., Han, J., Tapia, R., Saunders, E., Schmutz, J., Brettin, T., Larimer, F., Land, M., Hauser, L., Spring, S., Rohde, M., Kyrpides, N.C., Ivanova, N., Göker, M., Beller, H.R., Klenk, H.-P., Woyke, T. Complete genome sequence of Tolumonas auensis type strain (TA 4T). Standards in Genomic Sciences 5: 112-120, 2011.

CP002631 Han, J., Gronow, S., Teshima, H., Lapidus, A., Nolan, M., Lucas, S., Hammon, N., Deshpande, S., Cheng, J.-F., Zeytun, A., Tapia, R., Goodwin, L.A., Pitluck, S., Liolios, K., Pagani, I., Ivanova, N., Mavromatis, K., Mikhailova, N., Huntemann, M., Pati, A., Chen, A., Palaniappan, K., Land, M., Hauser, L., Brambilla, E.-M., Rohde, M., Göker, M., Woyke, T., Bristow, J., Eisen, J.A., Markowitz, V., Hugenholtz, P., Kyrpides, N.C., Klenk, H.-P., Detter, J.C. Complete genome sequence of Treponema succinifaciens type strain (6091T). Standards in Genomic Sciences 4: 361-370, 2011.

CP002049 Ivanova, N., Rohde, C., Munk, A.C., Nolan, M., Lucas, S., Glavina del Rio, T., Tice, H., Deshpande, S., Cheng, J.-F., Tapia, R., Han, J., Goodwin, L.A., Pitluck, S., Liolios, K., Mavromatis, K., Mikhailova, N., Pati, A., Chen, A., Palaniappan, K., Land, M., Hauser, L., Chang, Y.-J., Jeffries, C.D., Brambilla, E.-M., Rohde, M., Göker, M., Tindall, B.J., Woyke, T., Bristow, J., Eisen, J.A., Markowitz, V., Hugenholtz, P., Kyrpides, N.C., Klenk, H.-P., Lapidus, A. Complete genome sequence of Truepera radiovictrix type strain (RQ-24T). Standards in Genomic Sciences 4: 91-99, 2011.

CP001966; Munk, A.C., Lapidus, A., Lucas, S., Nolan, M., Tice, H., Cheng, J.-F., Glavina del

CP001967 Rio, T., Goodwin, L.A., Pitluck, S., Liolios, K., Huntemann, M., Ivanova, N., Mavromatis, K., Mikhailova, N., Pati, A., Chen, A., Palaniappan, K., Tapia, R., Han, J., Land, M., Hauser, L., Chang, Y.-J., Jeffries, C.D., Brettin, T., Yasawong, M., Brambilla, E.-M., Rohde, M., Sikorski, J., Göker, M., Detter, J.C., Woyke, T., Bristow, J., Eisen, J.A., Markowitz, V., Hugenholtz, P., Kyrpides, N.C., Klenk, H.-P. Complete genome sequence of Tsukamurella paurometabola type strain (no. 33T). Standards in Genomic Sciences 4: 342-351, 2011.

CP002959 Stackebrandt, E., Chertkov, O., Lapidus, A., Nolan, M., Lucas, S., Hammon, N., Deshpande, S., Cheng, J.F., Tapia, R., Goodwin, L.A., Pitluck, S., Liolios, K., Pagani, I., Ivanova, N., Mavromatis, K., Mikhailova, N., Huntemann, M., Pati, A., Chen, A., Palaniappan, K., Land, M., Pan, C., Rohde, M., Gronow, S., Göker, M., Detter, J.C., Bristow, J., Eisen, J.A., Markowitz, V., Hugenholtz, P., Woyke, T., Kyrpides, N.C., Klenk, H.P. Complete genome sequence of the free-living aerobic spirochete Turneriella parva type strain (HT). Standards in Genomic Sciences 8: 228-238, 2013 (doi:10.4056/sigs.3617113).

CP001820 Gronow, S., Welnitz, S., Lapidus, A., Nolan, M., Ivanova, N., Glavina del Rio, T., Copeland, A., Chen, F., Tice, H., Pitluck, S., Cheng, J.-F., Saunders, E., Brettin, T., Han, J., Detter, J.C., Bruce, D., Goodwin, L.A., Land, M., Hauser, L., Chang, Y.-J., Jeffries, C.D., Pati, A., Mavromatis, K., Mikhailova, N., Chen, A., Palaniappan, K., Chain, P., Rohde, M., Göker, M., Bristow, J., Eisen, J.A., Markowitz, V., Hugenholtz, P., Kyrpides, N.C., Klenk, H.-P., Lucas, S. Complete genome sequence of Veillonella parvula type strain (Te3T). Standards in Genomic Sciences 2: 57-65, 2010.

CP002100 Mavromatis, K., Sikorski, J., Pabst, E., Teshima, H., Lapidus, A., Lucas, S., Nolan, M., Glavina del Rio, T., Cheng, J.-F., Bruce, D., Goodwin, L.A., Pitluck, S., Liolios, K., Ivanova, N., Mikhailova, N., Pati, A., Chen, A., Palaniappan, K., Land, M., Hauser, L., Chang, Y.-J., Jeffries, C.D., Rohde, M., Spring, S., Göker, M., Wirth, R., Woyke, T., Bristow, J., Eisen, J.A., Markowitz, V., Hugenholtz, P., Klenk, H.-P., Kyrpides, N.C. Complete genome sequence of Vulcanisaeta distributa type strain (IC-017T). Standards in Genomic Sciences 3: 117-125, 2010.

CP002455 Lang, E., Teshima, H., Lucas, S., Lapidus, A., Hammon, N., Deshpande, S., Nolan, M., Cheng, J.-F., Pitluck, S., Liolios, K., Pagani, I., Mikhailova, N., Ivanova, N., Mavromatis, K., Pati, A., Tapia, R., Han, J., Goodwin, L.A., Chen, A., Palaniappan, K., Land, M., Hauser, L., Chang, Y.-J., Jeffries, C.D., Brambilla, E.-M., Kopitz, M., Rohde, M., Göker, M., Tindall, B.J., Detter, J.C., Woyke, T., Bristow, J., Eisen, J.A., Markowitz, V., Hugenholtz, P., Klenk, H.-P., Kyrpides, N.C. Complete genome sequence of Weeksella virosa type strain (9751T). Standards in Genomic Sciences 4: 81-90, 2011.

CP001821; CP001822 Foster, B., Pukall, R., Abt, B., Nolan, M., Glavina del Rio, T., Chen, F., Lucas, S., Tice, H., Pitluck, S., Cheng, J.-F., Chertkov, O., Brettin, T., Han, J., Detter, J.C., Bruce, D., Goodwin, L.A., Ivanova, N., Mavromatis, K., Pati, A., Mikhailova, N., Chen, A., Palaniappan, K., Land, M., Hauser, L., Chang, Y.-J., Jeffries, C.D., Chain, P., Rohde, M., Göker, M., Bristow, J., Eisen, J.A., Markowitz, V., Hugenholtz, P., Kyrpides, N.C., Klenk, H.-P., Lapidus, A. Complete genome sequence of Xylanimonas cellulosilytica type strain (XIL07T). Standards in Genomic Sciences 2: 1-8, 2010.
