# Supplementary material for: Genome-Based Taxonomic Classification of Bacteroidetes
Source: Front Microbiol. 2016 Dec 20;7:2003. doi: 10.3389/fmicb.2016.02003 (PMC5167729; doi:10.3389/fmicb.2016.02003)
Supplement: Supplementary file 4 [file DataSheet4.pdf]

Figure 1 - unconstrained comprehensive 16S rRNA gene ML and MP tree (UCT)

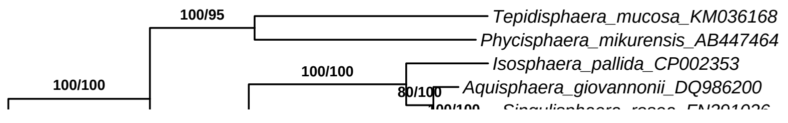
100/95
Tepidisphaera_mucosa_KM036168
Phycisphaera_mikurensis_AB447464
100/100
Isosphaera_pallida_CP002353
100/100
80/100
Aquisphaera_giovannonii_DQ986200

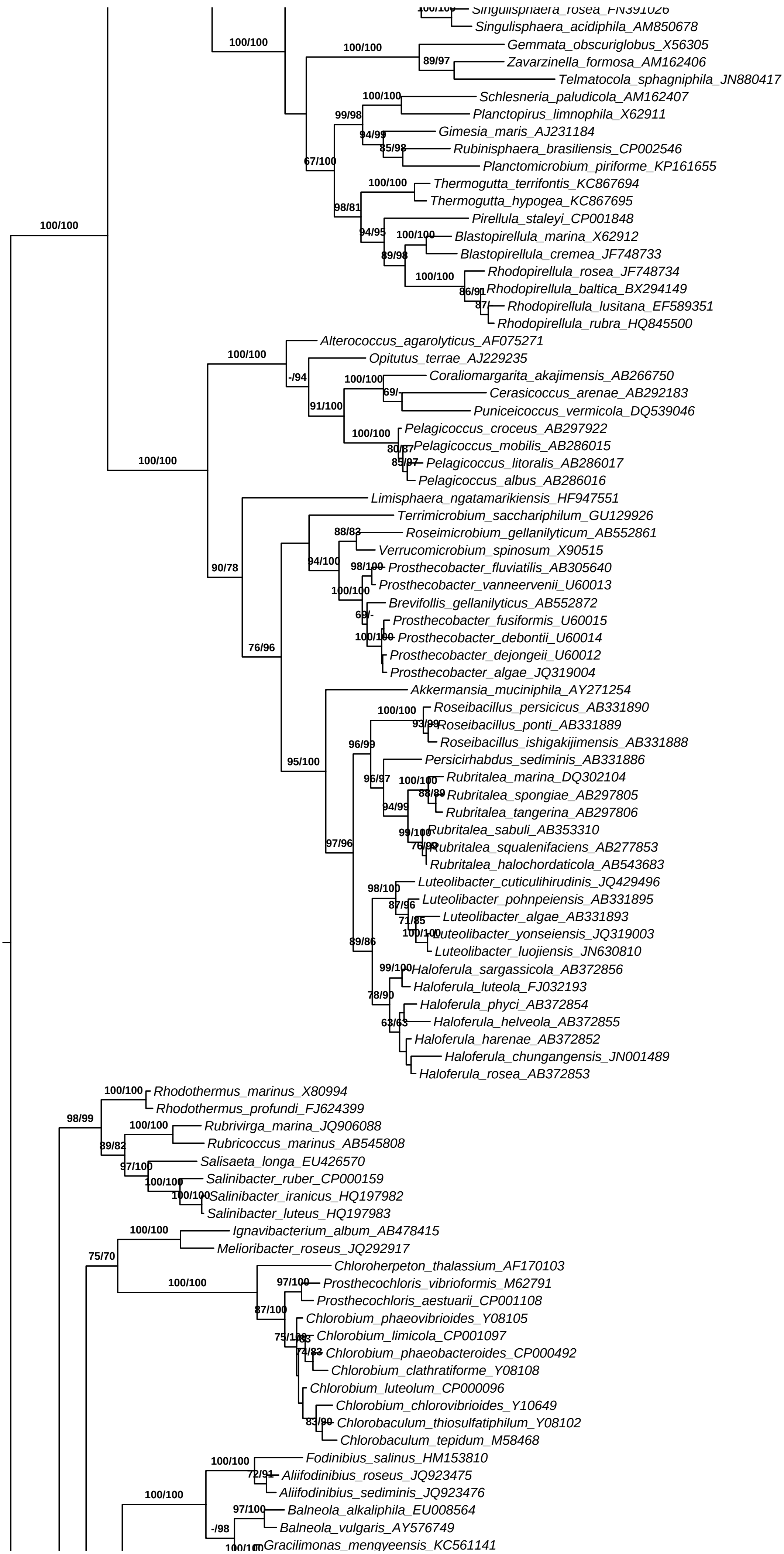
Singulisphaera_rosea_FN391026
Singulisphaera_acidiphila_AM850678
Gemmata_obscuriglobus_X56305
Zavarzinella_formosa_AM162406
Telmatocola_sphagniphila_JN880417
Schlesneria_paludicola_AM162407
Planctopirus_limnophila_X62911
Gimesia_maris_AJ231184
Rubinisphaera_brasiliensis_CP002546
Planctomicrobium_piriforme_KP161655
Thermogutta_terrifontis_KC867694
Thermogutta_hypogea_KC867695
Pirellula_staleyi_CP001848
Blastopirellula_marina_X62912
Blastopirellula_cremea_JF748733
Rhodopirellula_rosea_JF748734
Rhodopirellula_baltica_BX294149
Rhodopirellula_lusitana_EF589351
Rhodopirellula_rubra_HQ845500
Alterococcus_agarolyticus_AF075271
Opitutus_terrae_AJ229235
Coraliomargarita_akajimensis_AB266750
Cerasicoccus_arenae_AB292183
Puniceicoccus_vermicola_DQ539046
Pelagicoccus_croceus_AB297922
Pelagicoccus_mobilis_AB286015
Pelagicoccus_litoralis_AB286017
Pelagicoccus_albus_AB286016
Limisphaera_ngatamarikiensis_HF947551
Terrimicrobium_sacchariphilum_GU129926
Roseimicrobium_gellanilyticum_AB552861
Verrucomicrobium_spinosum_X90515
Prosthecobacter_fluviatilis_AB305640
Prosthecobacter_vanneervenii_U60013
Brevifollis_gellanilyticus_AB552872
Prosthecobacter_fusiformis_U60015
Prosthecobacter_debontii_U60014
Prosthecobacter_dejongeii_U60012
Prosthecobacter_algae_JQ319004
Akkermansia_muciniphila_AY271254
Roseibacillus_persicicus_AB331890
Roseibacillus_ponti_AB331889
Roseibacillus_ishigakijimensis_AB331888
Persicirhabdus_sediminis_AB331886
Rubritalea_marina_DQ302104
Rubritalea_spongiae_AB297805
Rubritalea_tangerina_AB297806
Rubritalea_sabuli_AB353310
Rubritalea_squalenifaciens_AB277853
Rubritalea_halochordaticola_AB543683
Luteolibacter_cuticulihirudinis_JQ429496
Luteolibacter_pohnpeiensis_AB331895
Luteolibacter_algae_AB331893
Luteolibacter_yonseiensis_JQ319003
Luteolibacter_luojiensis_JN630810
Haloferula_sargassicola_AB372856
Haloferula_luteola_FJ032193
Haloferula_phyci_AB372854
Haloferula_helveola_AB372855
Haloferula_harenae_AB372852
Haloferula_chungangensis_JN001489
Haloferula_rosea_AB372853
Rhodothermus_marinus_X80994
Rhodothermus_profundi_FJ624399
Rubrivirga_marina_JQ906088
Rubricoccus_marinus_AB545808
Salisaeta_longa_EU426570
Salinibacter_ruber_CP000159
Salinibacter_iranicus_HQ197982
Salinibacter_luteus_HQ197983
Ignavibacterium_album_AB478415
Melioribacter_roseus_JQ292917
Chloroherpeton_thalassium_AF170103
Prosthecochloris_vibrioformis_M62791
Prosthecochloris_aestuarii_CP001108
Chlorobium_phaeovibrioides_Y08105
Chlorobium_limicola_CP001097
Chlorobium_phaeobacteroides_CP000492
Chlorobium_clathratiforme_Y08108
Chlorobium_luteolum_CP000096
Chlorobium_chlorovibrioides_Y10649
Chlorobaculum_thiosulfatiphilum_Y08102
Chlorobaculum_tepidum_M58468
Fodinibius_salinus_HM153810
Aliifodinibius_roseus_JQ923475
Aliifodinibius_sediminis_JQ923476
Balneola_alkaliphila_EU008564
Balneola_vulgaris_AY576749
Gracilimonas_mengyeensis_KC561141

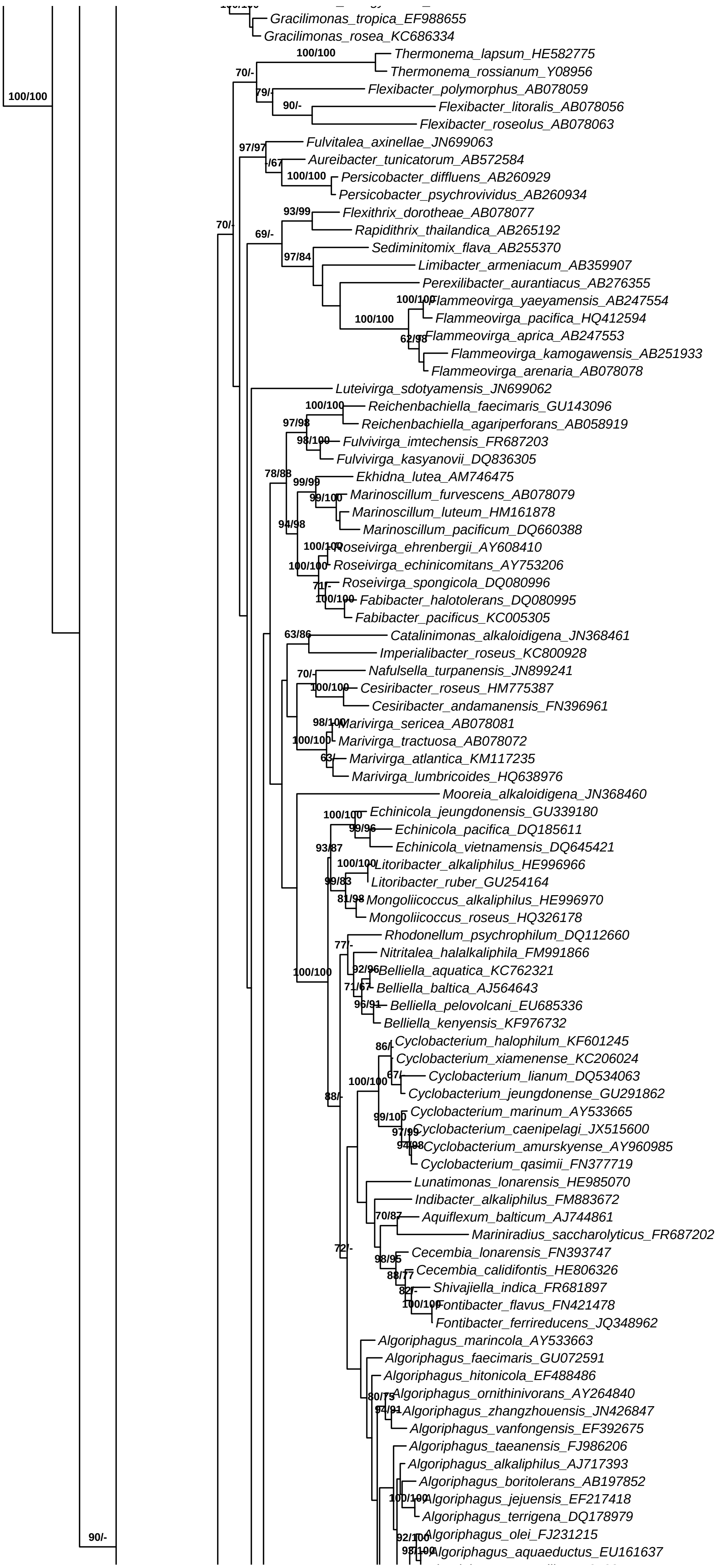
Gracilimonas_tropica_EF988655
Gracilimonas_rosea_KC686334
Thermonema_lapsum_HE582775
Thermonema_rossianum_Y08956
Flexibacter_polymorphus_AB078059
Flexibacter_litoralis_AB078056
Flexibacter_roseolus_AB078063
Fulvitalea_axinellae_JN699063
Aureibacter_tunicatorum_AB572584
Persicobacter_diffluens_AB260929
Persicobacter_psychrovividus_AB260934
Flexithrix_dorotheae_AB078077
Rapidithrix_thailandica_AB265192
Sediminitomix_flava_AB255370
Limibacter_armeniacum_AB359907
Perexilibacter_aurantiacus_AB276355
Flammeovirga_yaeyamensis_AB247554
Flammeovirga_pacifica_HQ412594
Flammeovirga_aprica_AB247553
Flammeovirga_kamogawensis_AB251933
Flammeovirga_arenaria_AB078078
Luteivirga_sdotyamensis_JN699062
Reichenbachiella_faecimaris_GU143096
Reichenbachiella_agariperforans_AB058919
Fulvivirga_imtechensis_FR687203
Fulvivirga_kasyanovii_DQ836305
Ekhidna_lutea_AM746475
Marinoscillum_furvescens_AB078079
Marinoscillum_luteum_HM161878
Marinoscillum_pacificum_DQ660388
Roseivirga_ehrenbergii_AY608410
Roseivirga_echinicomitans_AY753206
Roseivirga_spongicola_DQ080996
Fabibacter_halotolerans_DQ080995
Fabibacter_pacificus_KC005305
Catalinimonas_alkaloidigena_JN368461
Imperialibacter_roseus_KC800928
Nafulsella_turpanensis_JN899241
Cesiribacter_roseus_HM775387
Cesiribacter_andamanensis_FN396961
Marivirga_sericea_AB078081
Marivirga_tractuosa_AB078072
Marivirga_atlantica_KM117235
Marivirga_lumbricoides_HQ638976
Mooreia_alkaloidigena_JN368460
Echinicola_jeungdonensis_GU339180
Echinicola_pacifica_DQ185611
Echinicola_vietnamensis_DQ645421
Litoribacter_alkaliphilus_HE996966
Litoribacter_ruber_GU254164
Mongoliicoccus_alkaliphilus_HE996970
Mongoliicoccus_roseus_HQ326178
Rhodonellum_psychrophilum_DQ112660
Nitritalea_halalkaliphila_FM991866
Belliella_aquatica_KC762321
Belliella_baltica_AJ564643
Belliella_pelovolcani_EU685336
Belliella_kenyensis_KF976732
Cyclobacterium_halophilum_KF601245
Cyclobacterium_xiamenense_KC206024
Cyclobacterium_lianum_DQ534063
Cyclobacterium_jeungdonense_GU291862
Cyclobacterium_marinum_AY533665
Cyclobacterium_caenipelagi_JX515600
Cyclobacterium_amurskyense_AY960985
Cyclobacterium_qasimii_FN377719
Lunatimonas_lonarensis_HE985070
Indibacter_alkaliphilus_FM883672
Aquiflexum_balticum_AJ744861
Mariniradius_saccharolyticus_FR687202
Cecembia_lonarensis_FN393747
Cecembia_calidifontis_HE806326
Shivajiella_indica_FR681897
Fontibacter_flavus_FN421478
Fontibacter_ferrireducens_JQ348962
Algoriphagus_marincola_AY533663
Algoriphagus_faecimaris_GU072591
Algoriphagus_hitonicola_EF488486
Algoriphagus_ornithinivorans_AY264840
Algoriphagus_zhangzhouensis_JN426847
Algoriphagus_vanfongensis_EF392675
Algoriphagus_taeanensis_FJ986206
Algoriphagus_alkaliphilus_AJ717393
Algoriphagus_boritolerans_AB197852
Algoriphagus_jejuensis_EF217418
Algoriphagus_terrigena_DQ178979
Algoriphagus_olei_FJ231215
Algoriphagus_aquaeductus_EU161637

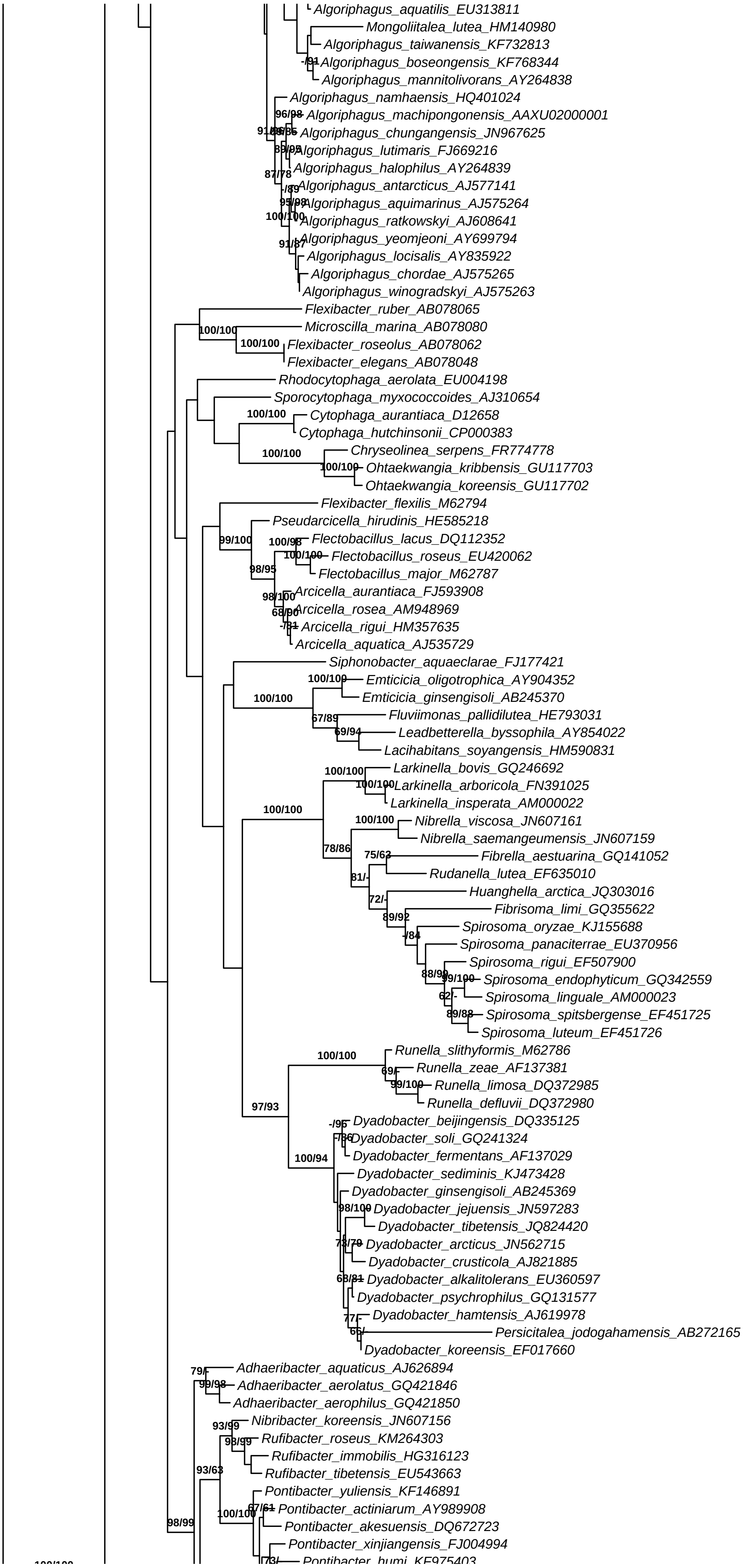
Algoriphagus_aquatilis_EU313811
Mongoliitalea_lutea_HM140980
Algoriphagus_taiwanensis_KF732813
Algoriphagus_boseongensis_KF768344
Algoriphagus_mannitolivorans_AY264838
Algoriphagus_namhaensis_HQ401024
Algoriphagus_machipongonensis_AAXU02000001
Algoriphagus_chungangensis_JN967625
Algoriphagus_lutimaris_FJ669216
Algoriphagus_halophilus_AY264839
Algoriphagus_antarcticus_AJ577141
Algoriphagus_aquimarinus_AJ575264
Algoriphagus_ratkowskyi_AJ608641
Algoriphagus_yeomjeoni_AY699794
Algoriphagus_locisalis_AY835922
Algoriphagus_chordae_AJ575265
Algoriphagus_winogradskyi_AJ575263
Flexibacter_ruber_AB078065
Microscilla_marina_AB078080
Flexibacter_roseolus_AB078062
Flexibacter_elegans_AB078048
Rhodocytophaga_aerolata_EU004198
Sporocytophaga_myxococcoides_AJ310654
Cytophaga_aurantiaca_D12658
Cytophaga_hutchinsonii_CP000383
Chryseolinea_serpens_FR774778
Ohtaekwangia_kribbensis_GU117703
Ohtaekwangia_koreensis_GU117702
Flexibacter_flexilis_M62794
Pseudarcicella_hirudinis_HE585218
Flectobacillus_lacus_DQ112352
Flectobacillus_roseus_EU420062
Flectobacillus_major_M62787
Arcicella_aurantiaca_FJ593908
Arcicella_rosea_AM948969
Arcicella_rigui_HM357635
Arcicella_aquatica_AJ535729
Siphonobacter_aquaeclarae_FJ177421
Emticicia_oligotrophica_AY904352
Emticicia_ginsengisoli_AB245370
Fluviimonas_pallidilutea_HE793031
Leadbetterella_byssophila_AY854022
Lacihabitans_soyangensis_HM590831
Larkinella_bovis_GQ246692
Larkinella_arboricola_FN391025
Larkinella_insperata_AM000022
Nibrella_viscosa_JN607161
Nibrella_saemangeumensis_JN607159
Fibrella_aestuarina_GQ141052
Rudanella_lutea_EF635010
Huanghella_arctica_JQ303016
Fibrisoma_limi_GQ355622
Spirosoma_oryzae_KJ155688
Spirosoma_panaciterrae_EU370956
Spirosoma_rigui_EF507900
Spirosoma_endophyticum_GQ342559
Spirosoma_linguale_AM000023
Spirosoma_spitsbergense_EF451725
Spirosoma_luteum_EF451726
Runella_slithyformis_M62786
Runella_zeae_AF137381
Runella_limosa_DQ372985
Runella_defluvii_DQ372980
Dyadobacter_beijingensis_DQ335125
Dyadobacter_soli_GQ241324
Dyadobacter_fermentans_AF137029
Dyadobacter_sediminis_KJ473428
Dyadobacter_ginsengisoli_AB245369
Dyadobacter_jejuensis_JN597283
Dyadobacter_tibetensis_JQ824420
Dyadobacter_arcticus_JN562715
Dyadobacter_crusticola_AJ821885
Dyadobacter_alkalitolerans_EU360597
Dyadobacter_psychrophilus_GQ131577
Dyadobacter_hamtensis_AJ619978
Persicitalea_jodogahamensis_AB272165
Dyadobacter_koreensis_EF017660
Adhaeribacter_aquaticus_AJ626894
Adhaeribacter_aerolatus_GQ421846
Adhaeribacter_aerophilus_GQ421850
Nibribacter_koreensis_JN607156
Rufibacter_roseus_KM264303
Rufibacter_immobilis_HG316123
Rufibacter_tibetensis_EU543663
Pontibacter_yuliensis_KF146891
Pontibacter_actiniarum_AY989908
Pontibacter_akesuensis_DQ672723
Pontibacter_xinjiangensis_FJ004994
Pontibacter_humi_KF975403

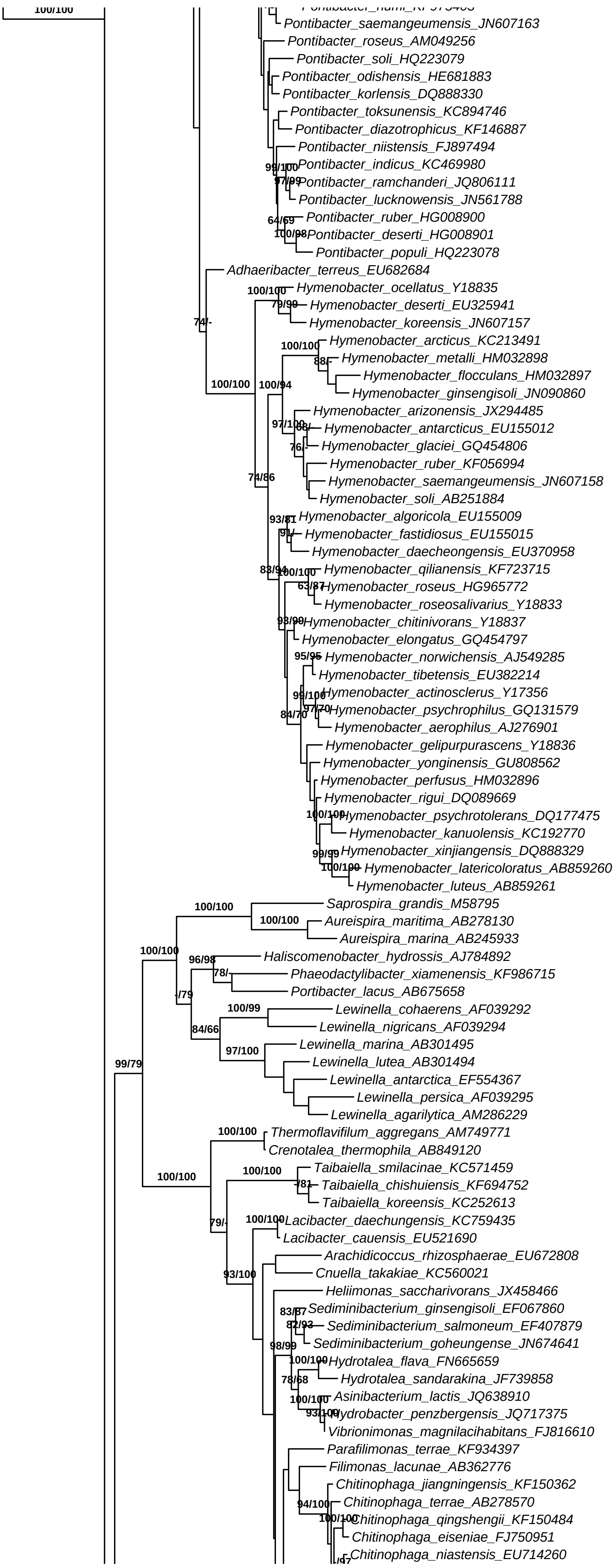

Pontibacter_saemangeumensis_JN607163
Pontibacter_roseus_AM049256
Pontibacter_soli_HQ223079
Pontibacter_odishensis_HE681883
Pontibacter_korlensis_DQ888330
Pontibacter_toksunensis_KC894746
Pontibacter_diazotrophicus_KF146887
Pontibacter_niistensis_FJ897494
Pontibacter_indicus_KC469980
Pontibacter_ramchanderi_JQ806111
Pontibacter_lucknowensis_JN561788
Pontibacter_ruber_HG008900
Pontibacter_deserti_HG008901
Pontibacter_populi_HQ223078
Adhaeribacter_terreus_EU682684
Hymenobacter_ocellatus_Y18835
Hymenobacter_deserti_EU325941
Hymenobacter_koreensis_JN607157
Hymenobacter_arcticus_KC213491
Hymenobacter_metalli_HM032898
Hymenobacter_flocculans_HM032897
Hymenobacter_ginsengisoli_JN090860
Hymenobacter_arizonensis_JX294485
Hymenobacter_antarcticus_EU155012
Hymenobacter_glaciei_GQ454806
Hymenobacter_ruber_KF056994
Hymenobacter_saemangeumensis_JN607158
Hymenobacter_soli_AB251884
Hymenobacter_algoricola_EU155009
Hymenobacter_fastidiosus_EU155015
Hymenobacter_daecheongensis_EU370958
Hymenobacter_qilianensis_KF723715
Hymenobacter_roseus_HG965772
Hymenobacter_roseosalivarius_Y18833
Hymenobacter_chitinivorans_Y18837
Hymenobacter_elongatus_GQ454797
Hymenobacter_norwichensis_AJ549285
Hymenobacter_tibetensis_EU382214
Hymenobacter_actinosclerus_Y17356
Hymenobacter_psychrophilus_GQ131579
Hymenobacter_aerophilus_AJ276901
Hymenobacter_gelipurpurascens_Y18836
Hymenobacter_yonginensis_GU808562
Hymenobacter_perfusus_HM032896
Hymenobacter_rigui_DQ089669
Hymenobacter_psychrotolerans_DQ177475
Hymenobacter_kanuolensis_KC192770
Hymenobacter_xinjiangensis_DQ888329
Hymenobacter_latericoloratus_AB859260
Hymenobacter_luteus_AB859261
Saprospira_grandis_M58795
Aureispira_maritima_AB278130
Aureispira_marina_AB245933
Haliscomenobacter_hydrossis_AJ784892
Phaeodactylibacter_xiamenensis_KF986715
Portibacter_lacus_AB675658
Lewinella_cohaerens_AF039292
Lewinella_nigricans_AF039294
Lewinella_marina_AB301495
Lewinella_lutea_AB301494
Lewinella_antarctica_EF554367
Lewinella_persica_AF039295
Lewinella_agarilytica_AM286229
Thermoflavifilum_aggregans_AM749771
Crenotalea_thermophila_AB849120
Taibaiella_smilacinae_KC571459
Taibaiella_chishuiensis_KF694752
Taibaiella_koreensis_KC252613
Lacibacter_daechungensis_KC759435
Lacibacter_cauensis_EU521690
Arachidicoccus_rhizosphaerae_EU672808
Cnuella_takakiae_KC560021
Heliimonas_saccharivorans_JX458466
Sediminibacterium_ginsengisoli_EF067860
Sediminibacterium_salmoneum_EF407879
Sediminibacterium_goheungense_JN674641
Hydrotalea_flava_FN665659
Hydrotalea_sandarakina_JF739858
Asinibacterium_lactis_JQ638910
Hydrobacter_penzbergensis_JQ717375
Vibrionimonas_magnilacihabitans_FJ816610
Parafilimonas_terrae_KF934397
Filimonas_lacunae_AB362776
Chitinophaga_jiangningensis_KF150362
Chitinophaga_terrae_AB278570
Chitinophaga_qingshengii_KF150484
Chitinophaga_eiseniae_FJ750951
Chitinophaga_niastensis_EU714260

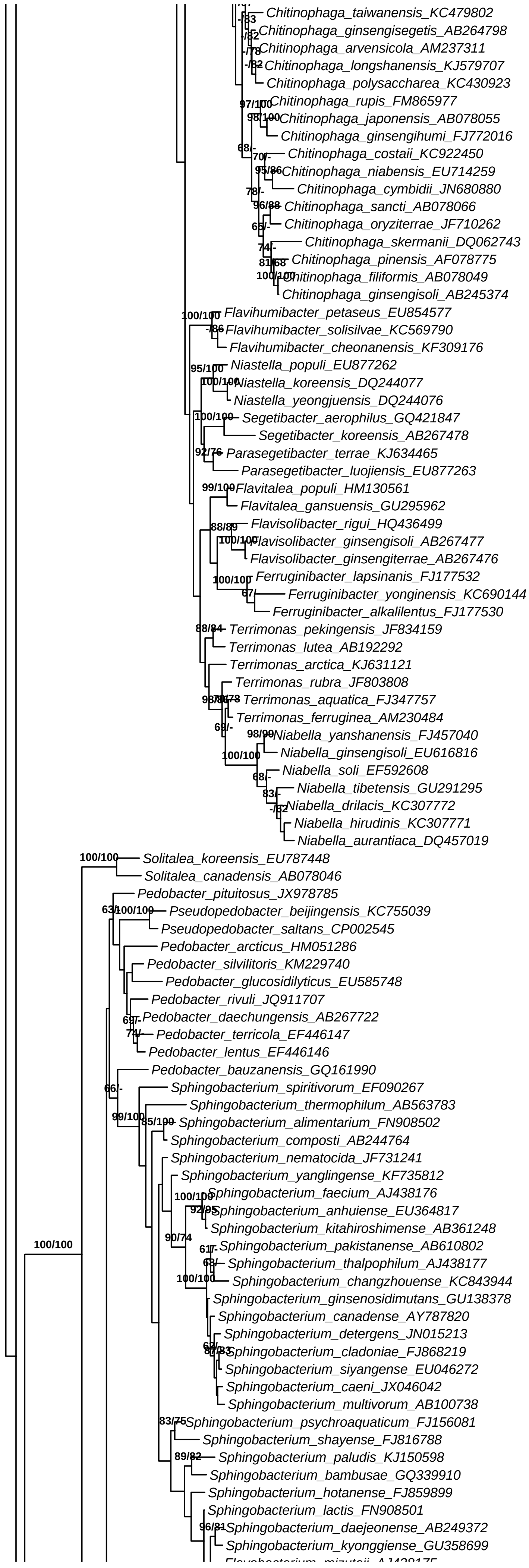

Chitinophaga_taiwanensis_KC479802
Chitinophaga_ginsengisegetis_AB264798
Chitinophaga_arvensicola_AM237311
Chitinophaga_longshanensis_KJ579707
Chitinophaga_polysaccharea_KC430923
Chitinophaga_rupis_FM865977
Chitinophaga_japonensis_AB078055
Chitinophaga_ginsengihumi_FJ772016
Chitinophaga_costaii_KC922450
Chitinophaga_niabensis_EU714259
Chitinophaga_cymbidii_JN680880
Chitinophaga_sancti_AB078066
Chitinophaga_oryziterrae_JF710262
Chitinophaga_skermanii_DQ062743
Chitinophaga_pinensis_AF078775
Chitinophaga_filiformis_AB078049
Chitinophaga_ginsengisoli_AB245374
Flavihumibacter_petaseus_EU854577
Flavihumibacter_solisilvae_KC569790
Flavihumibacter_cheonanensis_KF309176
Niastella_populi_EU877262
Niastella_koreensis_DQ244077
Niastella_yeongjuensis_DQ244076
Segetibacter_aerophilus_GQ421847
Segetibacter_koreensis_AB267478
Parasegetibacter_terrae_KJ634465
Parasegetibacter_luojiensis_EU877263
Flavitalea_populi_HM130561
Flavitalea_gansuensis_GU295962
Flavisolibacter_rigui_HQ436499
Flavisolibacter_ginsengisoli_AB267477
Flavisolibacter_ginsengiterrae_AB267476
Ferruginibacter_lapsinanis_FJ177532
Ferruginibacter_yonginensis_KC690144
Ferruginibacter_alkalilentus_FJ177530
Terrimonas_pekingensis_JF834159
Terrimonas_lutea_AB192292
Terrimonas_arctica_KJ631121
Terrimonas_rubra_JF803808
Terrimonas_aquatica_FJ347757
Terrimonas_ferruginea_AM230484
Niabella_yanshanensis_FJ457040
Niabella_ginsengisoli_EU616816
Niabella_soli_EF592608
Niabella_tibetensis_GU291295
Niabella_drilacis_KC307772
Niabella_hirudinis_KC307771
Niabella_aurantiaca_DQ457019
Solitalea_koreensis_EU787448
Solitalea_canadensis_AB078046
Pedobacter_pituitosus_JX978785
Pseudopedobacter_beijingensis_KC755039
Pseudopedobacter_saltans_CP002545
Pedobacter_arcticus_HM051286
Pedobacter_silvilitoris_KM229740
Pedobacter_glucosidilyticus_EU585748
Pedobacter_rivuli_JQ911707
Pedobacter_daechungensis_AB267722
Pedobacter_terricola_EF446147
Pedobacter_lentus_EF446146
Pedobacter_bauzanensis_GQ161990
Sphingobacterium_spiritivorum_EF090267
Sphingobacterium_thermophilum_AB563783
Sphingobacterium_alimentarium_FN908502
Sphingobacterium_composti_AB244764
Sphingobacterium_nematocida_JF731241
Sphingobacterium_yanglingense_KF735812
Sphingobacterium_faecium_AJ438176
Sphingobacterium_anhuiense_EU364817
Sphingobacterium_kitahiroshimense_AB361248
Sphingobacterium_pakistanense_AB610802
Sphingobacterium_thalpophilum_AJ438177
Sphingobacterium_changzhouense_KC843944
Sphingobacterium_ginsenosidimutans_GU138378
Sphingobacterium_canadense_AY787820
Sphingobacterium_detergens_JN015213
Sphingobacterium_cladoniae_FJ868219
Sphingobacterium_siyangense_EU046272
Sphingobacterium_caeni_JX046042
Sphingobacterium_multivorum_AB100738
Sphingobacterium_psychroaquaticum_FJ156081
Sphingobacterium_shayense_FJ816788
Sphingobacterium_paludis_KJ150598
Sphingobacterium_bambusae_GQ339910
Sphingobacterium_hotanense_FJ859899
Sphingobacterium_lactis_FN908501
Sphingobacterium_daejeonense_AB249372
Sphingobacterium_kyonggiense_GU358699

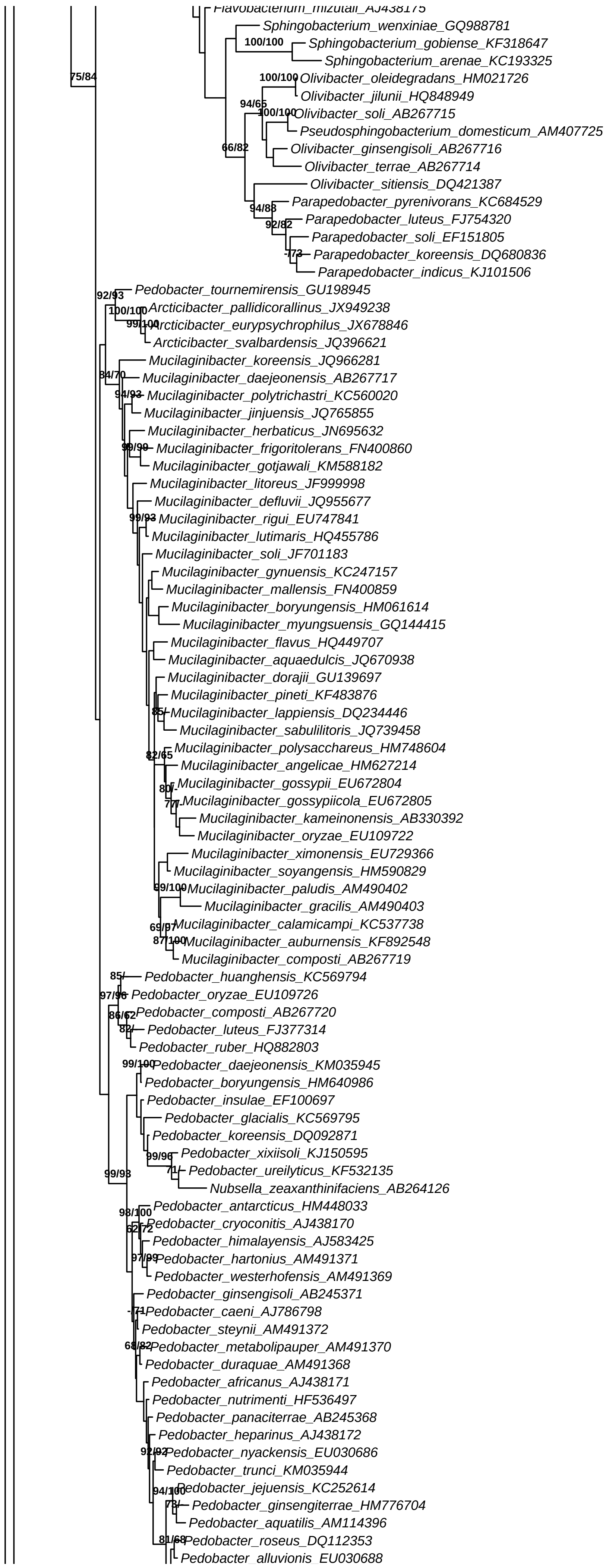

Sphingobacterium_wenxiniae_GQ988781
100/100
Sphingobacterium_gobiense_KF318647
Sphingobacterium_arenae_KC193325
75/84
100/100
Olivibacter_oleidegradans_HM021726
Olivibacter_jilunii_HQ848949
94/66
100/100
Olivibacter_soli_AB267715
Pseudosphingobacterium_domesticum_AM407725
66/82
Olivibacter_ginsengisoli_AB267716
Olivibacter_terrae_AB267714
Olivibacter_sitiensis_DQ421387
94/88
Parapedobacter_pyrenivorans_KC684529
92/82
Parapedobacter_luteus_FJ754320
Parapedobacter_soli_EF151805
-/73
Parapedobacter_koreensis_DQ680836
Parapedobacter_indicus_KJ101506
92/93
Pedobacter_tournemirensis_GU198945
100/100
Arcticibacter_pallidicorallinus_JX949238
99/100
Arcticibacter_eurypsychrophilus_JX678846
Arcticibacter_svalbardensis_JQ396621
Mucilaginibacter_koreensis_JQ966281
84/70
Mucilaginibacter_daejeonensis_AB267717
94/93
Mucilaginibacter_polytrichastri_KC560020
Mucilaginibacter_jinjuensis_JQ765855
Mucilaginibacter_herbaticus_JN695632
99/99
Mucilaginibacter_frigoritolerans_FN400860
Mucilaginibacter_gotjawali_KM588182
Mucilaginibacter_litoreus_JF999998
Mucilaginibacter_defluvii_JQ955677
99/93
Mucilaginibacter_rigui_EU747841
Mucilaginibacter_lutimaris_HQ455786
Mucilaginibacter_soli_JF701183
Mucilaginibacter_gynuensis_KC247157
Mucilaginibacter_mallensis_FN400859
Mucilaginibacter_boryungensis_HM061614
Mucilaginibacter_myungsuensis_GQ144415
Mucilaginibacter_flavus_HQ449707
Mucilaginibacter_aquaedulcis_JQ670938
Mucilaginibacter_dorajii_GU139697
Mucilaginibacter_pineti_KF483876
85/-
Mucilaginibacter_lappiensis_DQ234446
Mucilaginibacter_sabulilitoris_JQ739458
82/65
Mucilaginibacter_polysacchareus_HM748604
Mucilaginibacter_angelicae_HM627214
Mucilaginibacter_gossypii_EU672804
80/-
77/-
Mucilaginibacter_gossypiicola_EU672805
Mucilaginibacter_kameinonensis_AB330392
Mucilaginibacter_oryzae_EU109722
Mucilaginibacter_ximonensis_EU729366
Mucilaginibacter_soyangensis_HM590829
99/100
Mucilaginibacter_paludis_AM490402
Mucilaginibacter_gracilis_AM490403
69/97
Mucilaginibacter_calamicampi_KC537738
87/100
Mucilaginibacter_auburnensis_KF892548
Mucilaginibacter_composti_AB267719
85/-
Pedobacter_huanghensis_KC569794
97/96
Pedobacter_oryzae_EU109726
86/62
Pedobacter_composti_AB267720
82/-
Pedobacter_luteus_FJ377314
Pedobacter_ruber_HQ882803
99/100
Pedobacter_daejeonensis_KM035945
Pedobacter_boryungensis_HM640986
Pedobacter_insulae_EF100697
Pedobacter_glacialis_KC569795
Pedobacter_koreensis_DQ092871
99/96
Pedobacter_xixiisoli_KJ150595
71/-
Pedobacter_ureilyticus_KF532135
99/93
Nubsella_zeaxanthinifaciens_AB264126
98/100
Pedobacter_antarcticus_HM448033
62/72
Pedobacter_cryoconitis_AJ438170
Pedobacter_himalayensis_AJ583425
97/99
Pedobacter_hartonius_AM491371
Pedobacter_westerhofensis_AM491369
Pedobacter_ginsengisoli_AB245371
-/71
Pedobacter_caeni_AJ786798
Pedobacter_steynii_AM491372
68/82
Pedobacter_metabolipauper_AM491370
Pedobacter_duraquae_AM491368
Pedobacter_africanus_AJ438171
Pedobacter_nutrimenti_HF536497
Pedobacter_panaciterrae_AB245368
Pedobacter_heparinus_AJ438172
92/92
Pedobacter_nyackensis_EU030686
Pedobacter_trunci_KM035944
94/100
Pedobacter_jejuensis_KC252614
73/-
Pedobacter_ginsengiterrae_HM776704
Pedobacter_aquatilis_AM114396
81/68
Pedobacter_roseus_DQ112353
Pedobacter_alluvionis_EU030688

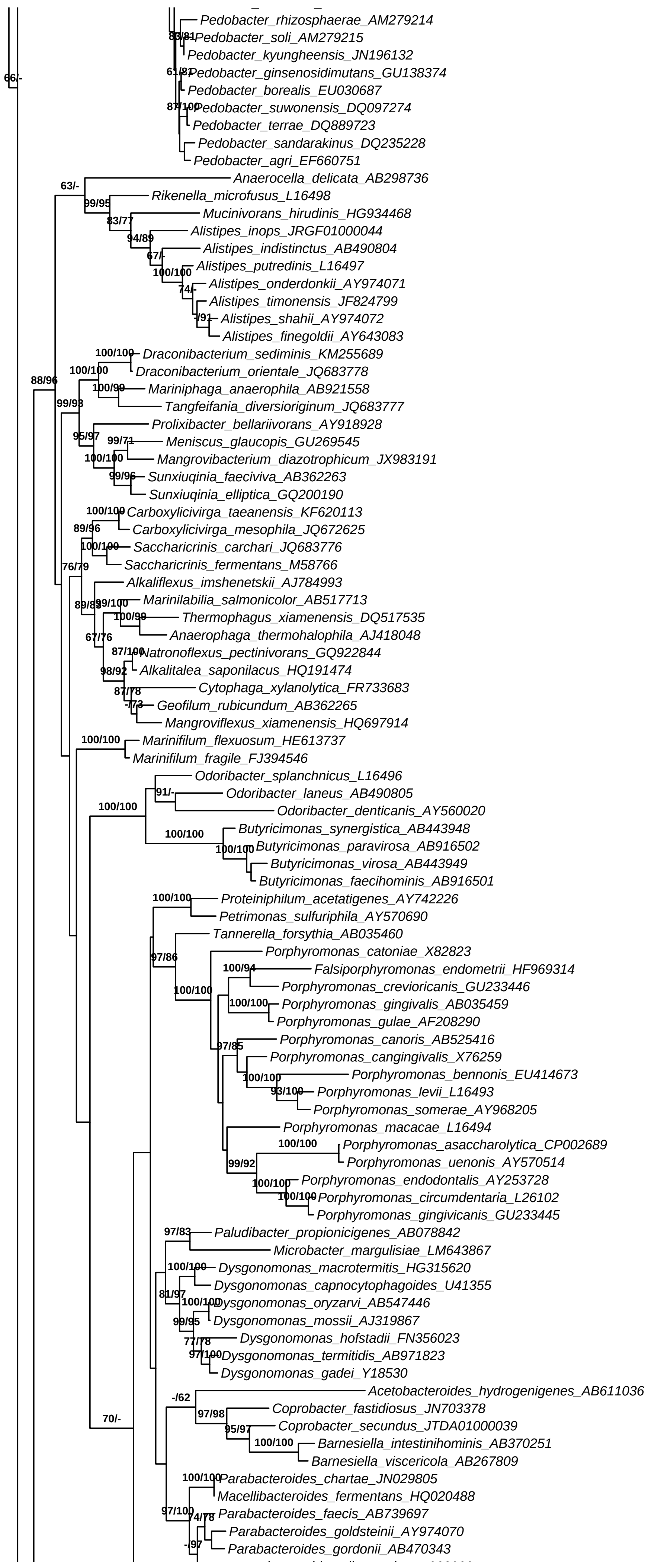

66/-
Pedobacter_rhizosphaerae_AM279214
83/81
Pedobacter_soli_AM279215
Pedobacter_kyungheensis_JN196132
61/81
Pedobacter_ginsenosidimutans_GU138374
Pedobacter_borealis_EU030687
87/100
Pedobacter_suwonensis_DQ097274
Pedobacter_terrae_DQ889723
Pedobacter_sandarakinus_DQ235228
Pedobacter_agri_EF660751
63/-
Anaerocella_delicata_AB298736
99/95
Rikenella_microfusus_L16498
83/77
Mucinivorans_hirudinis_HG934468
94/89
Alistipes_inops_JRGF01000044
67/-
Alistipes_indistinctus_AB490804
100/100
Alistipes_putredinis_L16497
74/-
Alistipes_onderdonkii_AY974071
Alistipes_timonensis_JF824799
-/91
Alistipes_shahii_AY974072
Alistipes_finegoldii_AY643083
88/96
100/100
Draconibacterium_sediminis_KM255689
100/100
Draconibacterium_orientale_JQ683778
99/93
100/99
Mariniphaga_anaerophila_AB921558
Tangfeifania_diversioriginum_JQ683777
Prolixibacter_bellariivorans_AY918928
95/97
99/71
Meniscus_glaucopis_GU269545
Mangrovibacterium_diazotrophicum_JX983191
100/100
99/96
Sunxiuqinia_faeciviva_AB362263
Sunxiuqinia_elliptica_GQ200190
100/100
Carboxylicivirga_taeanensis_KF620113
89/96
Carboxylicivirga_mesophila_JQ672625
100/100
Saccharicrinis_carchari_JQ683776
76/79
Saccharicrinis_fermentans_M58766
Alkaliflexus_imshenetskii_AJ784993
89/83
99/100
Marinilabilia_salmonicolor_AB517713
100/99
Thermophagus_xiamenensis_DQ517535
67/76
Anaerophaga_thermohalophila_AJ418048
87/100
Natronoflexus_pectinivorans_GQ922844
98/92
Alkalitalea_saponilacus_HQ191474
87/78
Cytophaga_xylanolytica_FR733683
-/73
Geofilum_rubicundum_AB362265
Mangroviflexus_xiamenensis_HQ697914
100/100
Marinifilum_flexuosum_HE613737
Marinifilum_fragile_FJ394546
Odoribacter_splanchnicus_L16496
91/-
Odoribacter_laneus_AB490805
100/100
Odoribacter_denticanis_AY560020
100/100
Butyricimonas_synergistica_AB443948
100/100
Butyricimonas_paravirosa_AB916502
Butyricimonas_virosa_AB443949
Butyricimonas_faecihominis_AB916501
100/100
Proteiniphilum_acetatigenes_AY742226
Petrimonas_sulfuriphila_AY570690
Tannerella_forsythia_AB035460
97/86
Porphyromonas_catoniae_X82823
100/94
Falsiporphyromonas_endometrii_HF969314
100/100
Porphyromonas_crevioricanis_GU233446
100/100
Porphyromonas_gingivalis_AB035459
Porphyromonas_gulae_AF208290
97/85
Porphyromonas_canoris_AB525416
Porphyromonas_cangingivalis_X76259
100/100
Porphyromonas_bennonis_EU414673
93/100
Porphyromonas_levii_L16493
Porphyromonas_somerae_AY968205
Porphyromonas_macacae_L16494
100/100
Porphyromonas_asaccharolytica_CP002689
99/92
Porphyromonas_uenonis_AY570514
100/100
Porphyromonas_endodontalis_AY253728
100/100
Porphyromonas_circumdentaria_L26102
Porphyromonas_gingivicanis_GU233445
97/83
Paludibacter_propionicigenes_AB078842
Microbacter_margulisiae_LM643867
100/100
Dysgonomonas_macrotermitis_HG315620
81/97
Dysgonomonas_capnocytophagoides_U41355
100/100
Dysgonomonas_oryzarvi_AB547446
99/95
Dysgonomonas_mossii_AJ319867
77/78
Dysgonomonas_hofstadii_FN356023
97/100
Dysgonomonas_termitidis_AB971823
Dysgonomonas_gadei_Y18530
-/62
Acetobacteroides_hydrogenigenes_AB611036
97/98
Coprobacter_fastidiosus_JN703378
95/97
Coprobacter_secundus_JTDA01000039
100/100
Barnesiella_intestinihominis_AB370251
70/-
Barnesiella_viscericola_AB267809
100/100
Parabacteroides_chartae_JN029805
Macellibacteroides_fermentans_HQ020488
97/100
Parabacteroides_faecis_AB739697
74/78
Parabacteroides_goldsteinii_AY974070
-/97
Parabacteroides_gordonii_AB470343

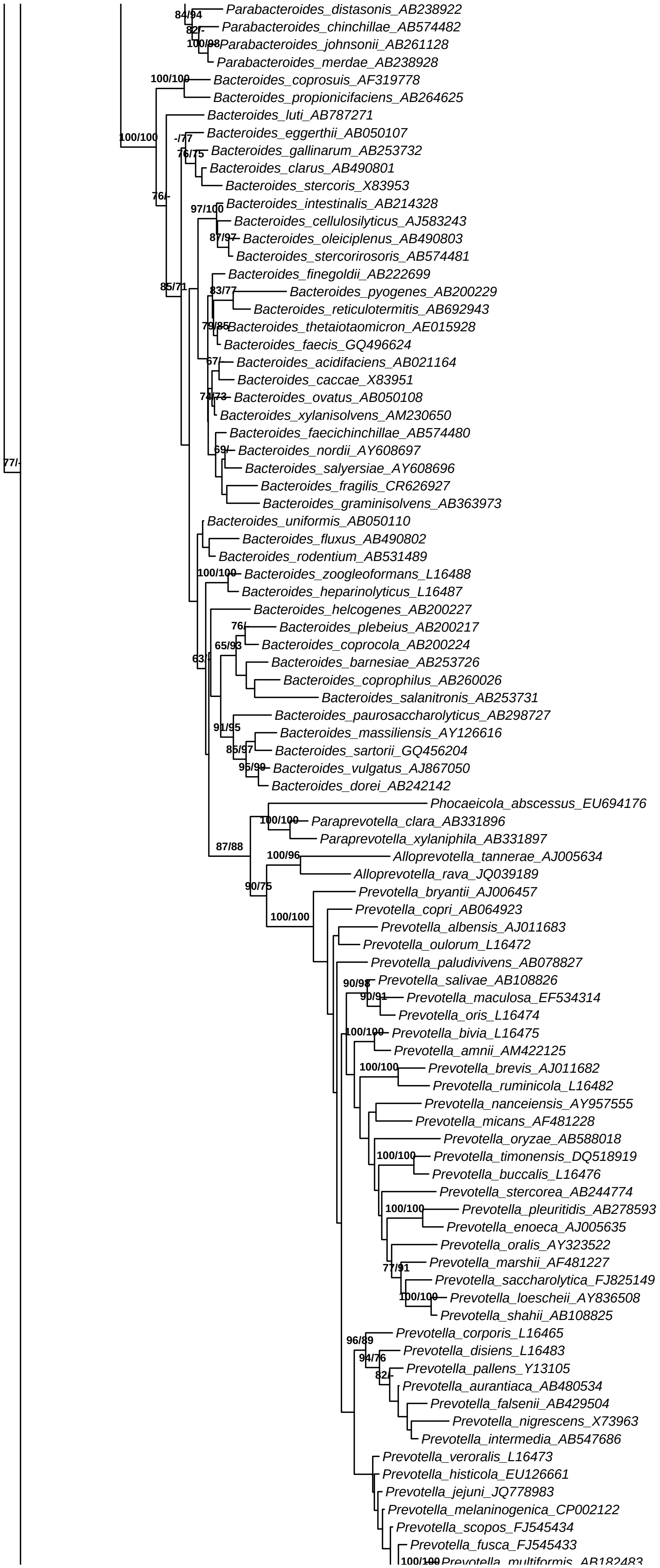
Parabacteroides_distasonis_AB238922
Parabacteroides_chinchillae_AB574482
Parabacteroides_johnsonii_AB261128
Parabacteroides_merdae_AB238928
Bacteroides_coprosuis_AF319778
Bacteroides_propionicifaciens_AB264625
Bacteroides_luti_AB787271
Bacteroides_eggerthii_AB050107
Bacteroides_gallinarum_AB253732
Bacteroides_clarus_AB490801
Bacteroides_stercoris_X83953
Bacteroides_intestinalis_AB214328
Bacteroides_cellulosilyticus_AJ583243
Bacteroides_oleiciplenus_AB490803
Bacteroides_stercorirosoris_AB574481
Bacteroides_finegoldii_AB222699
Bacteroides_pyogenes_AB200229
Bacteroides_reticulotermitis_AB692943
Bacteroides_thetaiotaomicron_AE015928
Bacteroides_faecis_GQ496624
Bacteroides_acidifaciens_AB021164
Bacteroides_caccae_X83951
Bacteroides_ovatus_AB050108
Bacteroides_xylanisolvens_AM230650
Bacteroides_faecichinchillae_AB574480
Bacteroides_nordii_AY608697
Bacteroides_salyersiae_AY608696
Bacteroides_fragilis_CR626927
Bacteroides_graminisolvens_AB363973
Bacteroides_uniformis_AB050110
Bacteroides_fluxus_AB490802
Bacteroides_rodentium_AB531489
Bacteroides_zoogleoformans_L16488
Bacteroides_heparinolyticus_L16487
Bacteroides_helcogenes_AB200227
Bacteroides_plebeius_AB200217
Bacteroides_coprocola_AB200224
Bacteroides_barnesiae_AB253726
Bacteroides_coprophilus_AB260026
Bacteroides_salanitronis_AB253731
Bacteroides_paurosaccharolyticus_AB298727
Bacteroides_massiliensis_AY126616
Bacteroides_sartorii_GQ456204
Bacteroides_vulgatus_AJ867050
Bacteroides_dorei_AB242142
Phocaeicola_abscessus_EU694176
Paraprevotella_clara_AB331896
Paraprevotella_xylaniphila_AB331897
Alloprevotella_tannerae_AJ005634
Alloprevotella_rava_JQ039189
Prevotella_bryantii_AJ006457
Prevotella_copri_AB064923
Prevotella_albensis_AJ011683
Prevotella_oulorum_L16472
Prevotella_paludivivens_AB078827
Prevotella_salivae_AB108826
Prevotella_maculosa_EF534314
Prevotella_oris_L16474
Prevotella_bivia_L16475
Prevotella_amnii_AM422125
Prevotella_brevis_AJ011682
Prevotella_ruminicola_L16482
Prevotella_nanceiensis_AY957555
Prevotella_micans_AF481228
Prevotella_oryzae_AB588018
Prevotella_timonensis_DQ518919
Prevotella_buccalis_L16476
Prevotella_stercorea_AB244774
Prevotella_pleuritidis_AB278593
Prevotella_enoeca_AJ005635
Prevotella_oralis_AY323522
Prevotella_marshii_AF481227
Prevotella_saccharolytica_FJ825149
Prevotella_loescheii_AY836508
Prevotella_shahii_AB108825
Prevotella_corporis_L16465
Prevotella_disiens_L16483
Prevotella_pallens_Y13105
Prevotella_aurantiaca_AB480534
Prevotella_falsenii_AB429504
Prevotella_nigrescens_X73963
Prevotella_intermedia_AB547686
Prevotella_veroralis_L16473
Prevotella_histicola_EU126661
Prevotella_jejuni_JQ778983
Prevotella_melaninogenica_CP002122
Prevotella_scopos_FJ545434
Prevotella_fusca_FJ545433
Prevotella_multiformis_AB182483

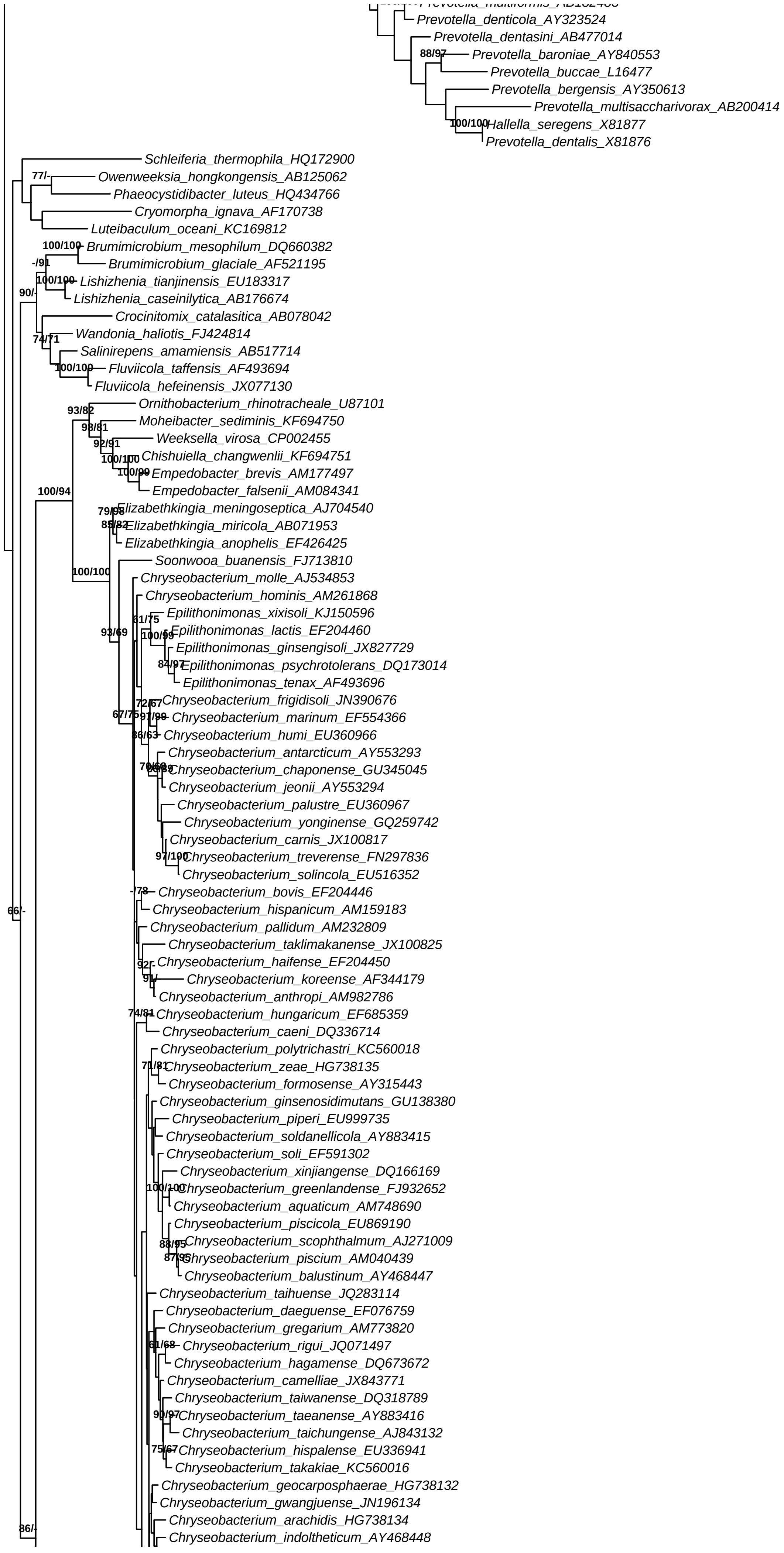
Prevotella_denticola_AY323524
Prevotella_dentasini_AB477014
88/97
Prevotella_baroniae_AY840553
Prevotella_buccae_L16477
Prevotella_bergensis_AY350613
Prevotella_multisaccharivorax_AB200414
100/100
Hallella_seregens_X81877
Prevotella_dentalis_X81876
Schleiferia_thermophila_HQ172900
77/-
Owenweeksia_hongkongensis_AB125062
Phaeocystidibacter_luteus_HQ434766
Cryomorpha_ignava_AF170738
Luteibaculum_oceani_KC169812
100/100
Brumimicrobium_mesophilum_DQ660382
-/91
Brumimicrobium_glaciale_AF521195
100/100
Lishizhenia_tianjinensis_EU183317
90/-
Lishizhenia_caseinilytica_AB176674
Crocinitomix_catalasitica_AB078042
Wandonia_haliotis_FJ424814
74/71
Salinirepens_amamiensis_AB517714
100/100
Fluviicola_taffensis_AF493694
Fluviicola_hefeinensis_JX077130
Ornithobacterium_rhinotracheale_U87101
93/82
Moheibacter_sediminis_KF694750
98/81
Weeksella_virosa_CP002455
92/91
Chishuiella_changwenlii_KF694751
100/100
Empedobacter_brevis_AM177497
100/99
Empedobacter_falsenii_AM084341
100/94
Elizabethkingia_meningoseptica_AJ704540
79/98
Elizabethkingia_miricola_AB071953
85/82
Elizabethkingia_anophelis_EF426425
Soonwooa_buanensis_FJ713810
100/100
Chryseobacterium_molle_AJ534853
Chryseobacterium_hominis_AM261868
Epilithonimonas_xixisoli_KJ150596
61/75
Epilithonimonas_lactis_EF204460
100/99
93/69
Epilithonimonas_ginsengisoli_JX827729
84/97
Epilithonimonas_psychrotolerans_DQ173014
Epilithonimonas_tenax_AF493696
Chryseobacterium_frigidisoli_JN390676
72/67
67/75
Chryseobacterium_marinum_EF554366
97/99
Chryseobacterium_humi_EU360966
86/63
Chryseobacterium_antarcticum_AY553293
Chryseobacterium_chaponense_GU345045
Chryseobacterium_jeonii_AY553294
Chryseobacterium_palustre_EU360967
Chryseobacterium_yonginense_GQ259742
Chryseobacterium_carnis_JX100817
97/100
Chryseobacterium_treverense_FN297836
Chryseobacterium_solincola_EU516352
-/78
Chryseobacterium_bovis_EF204446
Chryseobacterium_hispanicum_AM159183
66/-
Chryseobacterium_pallidum_AM232809
Chryseobacterium_taklimakanense_JX100825
Chryseobacterium_haifense_EF204450
Chryseobacterium_koreense_AF344179
Chryseobacterium_anthropi_AM982786
74/81
Chryseobacterium_hungaricum_EF685359
Chryseobacterium_caeni_DQ336714
Chryseobacterium_polytrichastri_KC560018
71/81
Chryseobacterium_zeae_HG738135
Chryseobacterium_formosense_AY315443
Chryseobacterium_ginsenosidimutans_GU138380
Chryseobacterium_piperi_EU999735
Chryseobacterium_soldanellicola_AY883415
Chryseobacterium_soli_EF591302
Chryseobacterium_xinjiangense_DQ166169
100/100
Chryseobacterium_greenlandense_FJ932652
Chryseobacterium_aquaticum_AM748690
Chryseobacterium_piscicola_EU869190
88/95
Chryseobacterium_scophthalmum_AJ271009
87/95
Chryseobacterium_piscium_AM040439
Chryseobacterium_balustinum_AY468447
Chryseobacterium_taihuense_JQ283114
Chryseobacterium_daeguense_EF076759
Chryseobacterium_gregarium_AM773820
61/68
Chryseobacterium_rigui_JQ071497
Chryseobacterium_hagamense_DQ673672
Chryseobacterium_camelliae_JX843771
Chryseobacterium_taiwanense_DQ318789
90/97
Chryseobacterium_taeanense_AY883416
Chryseobacterium_taichungense_AJ843132
75/67
Chryseobacterium_hispalense_EU336941
Chryseobacterium_takakiae_KC560016
Chryseobacterium_geocarposphaerae_HG738132
Chryseobacterium_gwangjuense_JN196134
Chryseobacterium_arachidis_HG738134
86/-
Chryseobacterium_indoltheticum_AY468448

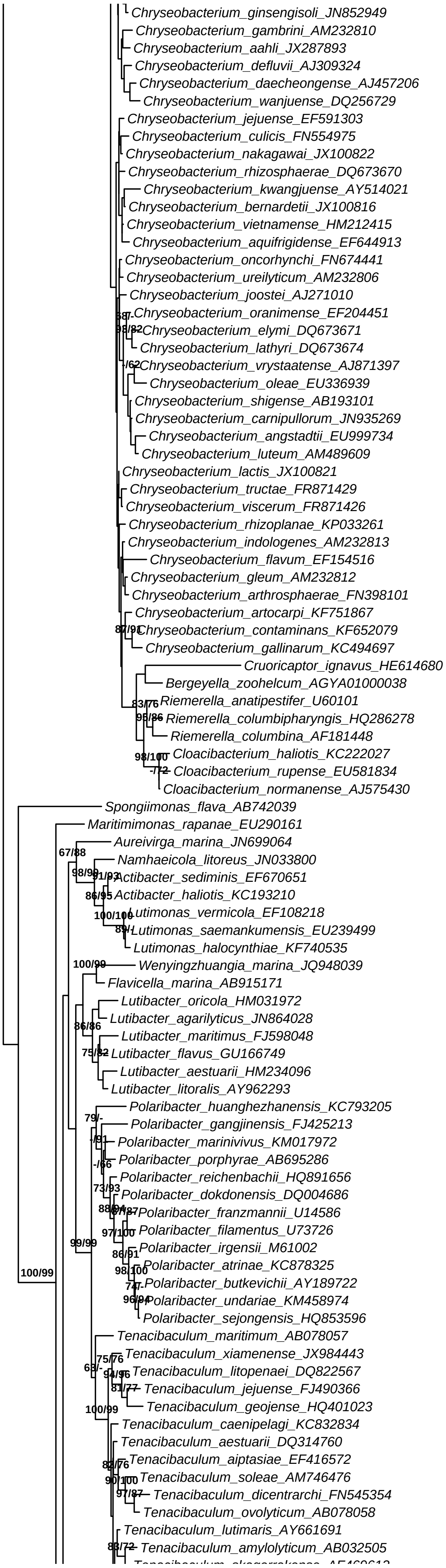

Chryseobacterium_ginsengisoli_JN852949
Chryseobacterium_gambrini_AM232810
Chryseobacterium_aahli_JX287893
Chryseobacterium_defluvii_AJ309324
Chryseobacterium_daecheongense_AJ457206
Chryseobacterium_wanjuense_DQ256729
Chryseobacterium_jejuense_EF591303
Chryseobacterium_culicis_FN554975
Chryseobacterium_nakagawai_JX100822
Chryseobacterium_rhizosphaerae_DQ673670
Chryseobacterium_kwangjuense_AY514021
Chryseobacterium_bernardetii_JX100816
Chryseobacterium_vietnamense_HM212415
Chryseobacterium_aquifrigidense_EF644913
Chryseobacterium_oncorhynchi_FN674441
Chryseobacterium_ureilyticum_AM232806
Chryseobacterium_joostei_AJ271010
Chryseobacterium_oranimense_EF204451
Chryseobacterium_elymi_DQ673671
Chryseobacterium_lathyri_DQ673674
Chryseobacterium_vrystaatense_AJ871397
Chryseobacterium_oleae_EU336939
Chryseobacterium_shigense_AB193101
Chryseobacterium_carnipullorum_JN935269
Chryseobacterium_angstadtii_EU999734
Chryseobacterium_luteum_AM489609
Chryseobacterium_lactis_JX100821
Chryseobacterium_tructae_FR871429
Chryseobacterium_viscerum_FR871426
Chryseobacterium_rhizoplanae_KP033261
Chryseobacterium_indologenes_AM232813
Chryseobacterium_flavum_EF154516
Chryseobacterium_gleum_AM232812
Chryseobacterium_arthrosphaerae_FN398101
Chryseobacterium_artocarpi_KF751867
Chryseobacterium_contaminans_KF652079
Chryseobacterium_gallinarum_KC494697
Cruoricaptor_ignavus_HE614680
Bergeyella_zoohelcum_AGYA01000038
Riemerella_anatipestifer_U60101
Riemerella_columbipharyngis_HQ286278
Riemerella_columbina_AF181448
Cloacibacterium_haliotis_KC222027
Cloacibacterium_rupense_EU581834
Cloacibacterium_normanense_AJ575430
Spongiimonas_flava_AB742039
Maritimimonas_rapanae_EU290161
Aureivirga_marina_JN699064
Namhaeicola_litoreus_JN033800
Actibacter_sediminis_EF670651
Actibacter_haliotis_KC193210
Lutimonas_vermicola_EF108218
Lutimonas_saemankumensis_EU239499
Lutimonas_halocynthiae_KF740535
Wenyingzhuangia_marina_JQ948039
Flavicella_marina_AB915171
Lutibacter_oricola_HM031972
Lutibacter_agarilyticus_JN864028
Lutibacter_maritimus_FJ598048
Lutibacter_flavus_GU166749
Lutibacter_aestuarii_HM234096
Lutibacter_litoralis_AY962293
Polaribacter_huanghezhanensis_KC793205
Polaribacter_gangjinensis_FJ425213
Polaribacter_marinivivus_KM017972
Polaribacter_porphyrae_AB695286
Polaribacter_reichenbachii_HQ891656
Polaribacter_dokdonensis_DQ004686
Polaribacter_franzmannii_U14586
Polaribacter_filamentus_U73726
Polaribacter_irgensii_M61002
Polaribacter_atrinae_KC878325
Polaribacter_butkevichii_AY189722
Polaribacter_undariae_KM458974
Polaribacter_sejongensis_HQ853596
Tenacibaculum_maritimum_AB078057
Tenacibaculum_xiamenense_JX984443
Tenacibaculum_litopenaei_DQ822567
Tenacibaculum_jejuense_FJ490366
Tenacibaculum_geojense_HQ401023
Tenacibaculum_caenipelagi_KC832834
Tenacibaculum_aestuarii_DQ314760
Tenacibaculum_aiptasiae_EF416572
Tenacibaculum_soleae_AM746476
Tenacibaculum_dicentrarchi_FN545354
Tenacibaculum_ovolyticum_AB078058
Tenacibaculum_lutimaris_AY661691
Tenacibaculum_amylolyticum_AB032505

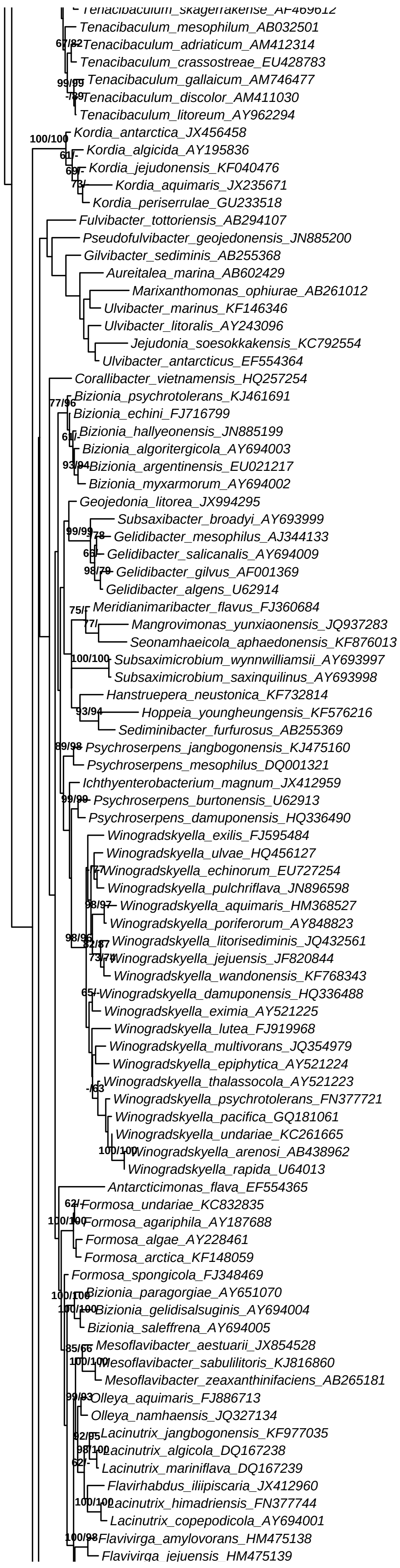
Tenacibaculum_skagerrakense_AF469612
Tenacibaculum_mesophilum_AB032501
Tenacibaculum_adriaticum_AM412314
Tenacibaculum_crassostreae_EU428783
Tenacibaculum_gallaicum_AM746477
Tenacibaculum_discolor_AM411030
Tenacibaculum_litoreum_AY962294
Kordia_antarctica_JX456458
Kordia_algicida_AY195836
Kordia_jejudonensis_KF040476
Kordia_aquimaris_JX235671
Kordia_periserrulae_GU233518
Fulvibacter_tottoriensis_AB294107
Pseudofulvibacter_geojedonensis_JN885200
Gilvibacter_sediminis_AB255368
Aureitalea_marina_AB602429
Marixanthomonas_ophiurae_AB261012
Ulvibacter_marinus_KF146346
Ulvibacter_litoralis_AY243096
Jejudonia_soesokkakensis_KC792554
Ulvibacter_antarcticus_EF554364
Corallibacter_vietnamensis_HQ257254
Bizionia_psychrotolerans_KJ461691
Bizionia_echini_FJ716799
Bizionia_hallyeonensis_JN885199
Bizionia_algoritergicola_AY694003
Bizionia_argentinensis_EU021217
Bizionia_myxarmorum_AY694002
Geojedonia_litorea_JX994295
Subsaxibacter_broadyi_AY693999
Gelidibacter_mesophilus_AJ344133
Gelidibacter_salicanalis_AY694009
Gelidibacter_gilvus_AF001369
Gelidibacter_algens_U62914
Meridianimaribacter_flavus_FJ360684
Mangrovimonas_yunxiaonensis_JQ937283
Seonamhaeicola_aphaedonensis_KF876013
Subsaximicrobium_wynnwilliamsii_AY693997
Subsaximicrobium_saxinquilinus_AY693998
Hanstruepera_neustonica_KF732814
Hoppeia_youngheungensis_KF576216
Sediminibacter_furfurosus_AB255369
Psychroserpens_jangbogonensis_KJ475160
Psychroserpens_mesophilus_DQ001321
Ichthyenterobacterium_magnum_JX412959
Psychroserpens_burtonensis_U62913
Psychroserpens_damuponensis_HQ336490
Winogradskyella_exilis_FJ595484
Winogradskyella_ulvae_HQ456127
Winogradskyella_echinorum_EU727254
Winogradskyella_pulchriflava_JN896598
Winogradskyella_aquimaris_HM368527
Winogradskyella_poriferorum_AY848823
Winogradskyella_litorisediminis_JQ432561
Winogradskyella_jejuensis_JF820844
Winogradskyella_wandonensis_KF768343
Winogradskyella_damuponensis_HQ336488
Winogradskyella_eximia_AY521225
Winogradskyella_lutea_FJ919968
Winogradskyella_multivorans_JQ354979
Winogradskyella_epiphytica_AY521224
Winogradskyella_thalassocola_AY521223
Winogradskyella_psychrotolerans_FN377721
Winogradskyella_pacifica_GQ181061
Winogradskyella_undariae_KC261665
Winogradskyella_arenosi_AB438962
Winogradskyella_rapida_U64013
Antarcticimonas_flava_EF554365
Formosa_undariae_KC832835
Formosa_agariphila_AY187688
Formosa_algae_AY228461
Formosa_arctica_KF148059
Formosa_spongicola_FJ348469
Bizionia_paragorgiae_AY651070
Bizionia_gelidisalsuginis_AY694004
Bizionia_saleffrena_AY694005
Mesoflavibacter_aestuarii_JX854528
Mesoflavibacter_sabulilitoris_KJ816860
Mesoflavibacter_zeaxanthinifaciens_AB265181
Olleya_aquimaris_FJ886713
Olleya_namhaensis_JQ327134
Lacinutrix_jangbogonensis_KF977035
Lacinutrix_algicola_DQ167238
Lacinutrix_mariniflava_DQ167239
Flavirhabdus_iliipiscaria_JX412960
Lacinutrix_himadriensis_FN377744
Lacinutrix_copepodicola_AY694001
Flavivirga_amylovorans_HM475138
Flavivirga_jejuensis_HM475139

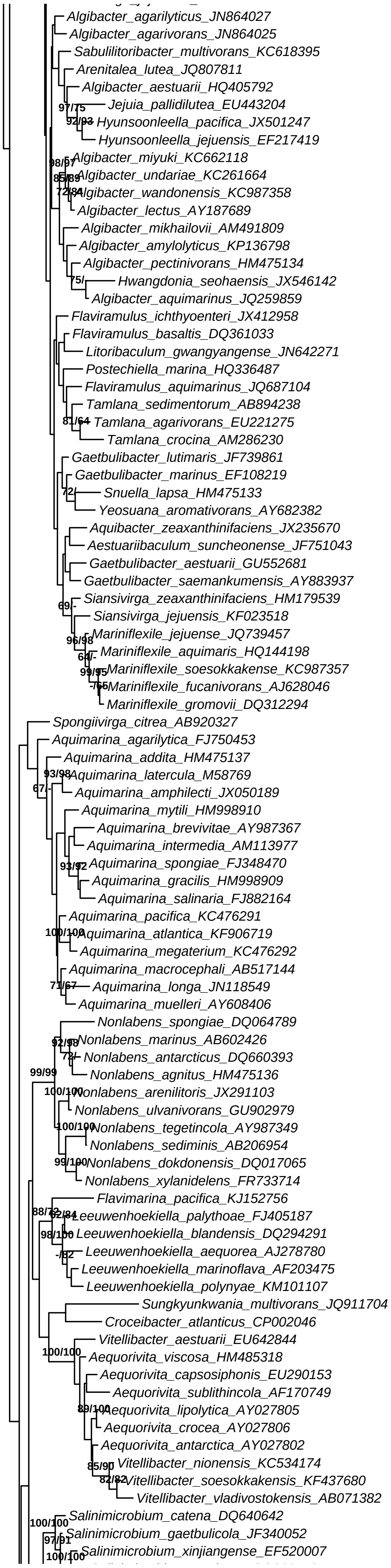

Algibacter_agarilyticus_JN864027
Algibacter_agarivorans_JN864025
Sabulilitoribacter_multivorans_KC618395
Arenitalea_lutea_JQ807811
Algibacter_aestuarii_HQ405792
Jejuia_pallidilutea_EU443204
Hyunsoonleella_pacifica_JX501247
Hyunsoonleella_jejuensis_EF217419
Algibacter_miyuki_KC662118
Algibacter_undariae_KC261664
Algibacter_wandonensis_KC987358
Algibacter_lectus_AY187689
Algibacter_mikhailovii_AM491809
Algibacter_amylolyticus_KP136798
Algibacter_pectinivorans_HM475134
Hwangdonia_seohaensis_JX546142
Algibacter_aquimarinus_JQ259859
Flaviramulus_ichthyoenteri_JX412958
Flaviramulus_basaltis_DQ361033
Litoribaculum_gwangyangense_JN642271
Postechiella_marina_HQ336487
Flaviramulus_aquimarinus_JQ687104
Tamlana_sedimentorum_AB894238
Tamlana_agarivorans_EU221275
Tamlana_crocina_AM286230
Gaetbulibacter_lutimaris_JF739861
Gaetbulibacter_marinus_EF108219
Snuella_lapsa_HM475133
Yeosuana_aromativorans_AY682382
Aquibacter_zeaxanthinifaciens_JX235670
Aestuariibaculum_suncheonense_JF751043
Gaetbulibacter_aestuarii_GU552681
Gaetbulibacter_saemankumensis_AY883937
Siansivirga_zeaxanthinifaciens_HM179539
Siansivirga_jejuensis_KF023518
Mariniflexile_jejuense_JQ739457
Mariniflexile_aquimaris_HQ144198
Mariniflexile_soesokkakense_KC987357
Mariniflexile_fucanivorans_AJ628046
Mariniflexile_gromovii_DQ312294
Spongiivirga_citrea_AB920327
Aquimarina_agarilytica_FJ750453
Aquimarina_addita_HM475137
Aquimarina_latercula_M58769
Aquimarina_amphilecti_JX050189
Aquimarina_mytili_HM998910
Aquimarina_brevivitae_AY987367
Aquimarina_intermedia_AM113977
Aquimarina_spongiae_FJ348470
Aquimarina_gracilis_HM998909
Aquimarina_salinaria_FJ882164
Aquimarina_pacifica_KC476291
Aquimarina_atlantica_KF906719
Aquimarina_megaterium_KC476292
Aquimarina_macrocephali_AB517144
Aquimarina_longa_JN118549
Aquimarina_muelleri_AY608406
Nonlabens_spongiae_DQ064789
Nonlabens_marinus_AB602426
Nonlabens_antarcticus_DQ660393
Nonlabens_agnitus_HM475136
Nonlabens_arenilitoris_JX291103
Nonlabens_ulvanivorans_GU902979
Nonlabens_tegetincola_AY987349
Nonlabens_sediminis_AB206954
Nonlabens_dokdonensis_DQ017065
Nonlabens_xylanidelens_FR733714
Flavimarina_pacifica_KJ152756
Leeuwenhoekiella_palythoae_FJ405187
Leeuwenhoekiella_blandensis_DQ294291
Leeuwenhoekiella_aequorea_AJ278780
Leeuwenhoekiella_marinoflava_AF203475
Leeuwenhoekiella_polynyae_KM101107
Sungkyunkwania_multivorans_JQ911704
Croceibacter_atlanticus_CP002046
Vitellibacter_aestuarii_EU642844
Aequorivita_viscosa_HM485318
Aequorivita_capsosiphonis_EU290153
Aequorivita_sublithincola_AF170749
Aequorivita_lipolytica_AY027805
Aequorivita_crocea_AY027806
Aequorivita_antarctica_AY027802
Vitellibacter_nionensis_KC534174
Vitellibacter_soesokkakensis_KF437680
Vitellibacter_vladivostokensis_AB071382
Salinimicrobium_catena_DQ640642
Salinimicrobium_gaetbulicola_JF340052
Salinimicrobium_xinjiangense_EF520007

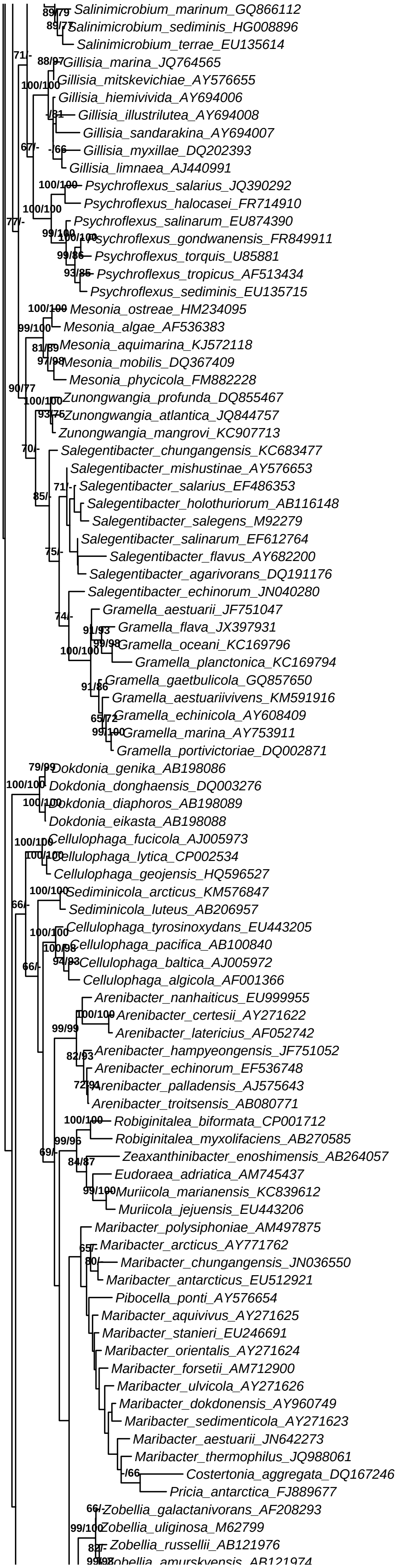
Salinimicrobium_marinum_GQ866112
Salinimicrobium_sediminis_HG008896
Salinimicrobium_terrae_EU135614
Gillisia_marina_JQ764565
Gillisia_mitskevichiae_AY576655
Gillisia_hiemivivida_AY694006
Gillisia_illustrilutea_AY694008
Gillisia_sandarakina_AY694007
Gillisia_myxillae_DQ202393
Gillisia_limnaea_AJ440991
Psychroflexus_salarius_JQ390292
Psychroflexus_halocasei_FR714910
Psychroflexus_salinarum_EU874390
Psychroflexus_gondwanensis_FR849911
Psychroflexus_torquis_U85881
Psychroflexus_tropicus_AF513434
Psychroflexus_sediminis_EU135715
Mesonia_ostreae_HM234095
Mesonia_algae_AF536383
Mesonia_aquimarina_KJ572118
Mesonia_mobilis_DQ367409
Mesonia_phycicola_FM882228
Zunongwangia_profunda_DQ855467
Zunongwangia_atlantica_JQ844757
Zunongwangia_mangrovi_KC907713
Salegentibacter_chungangensis_KC683477
Salegentibacter_mishustinae_AY576653
Salegentibacter_salarius_EF486353
Salegentibacter_holothuriorum_AB116148
Salegentibacter_salegens_M92279
Salegentibacter_salinarum_EF612764
Salegentibacter_flavus_AY682200
Salegentibacter_agarivorans_DQ191176
Salegentibacter_echinorum_JN040280
Gramella_aestuarii_JF751047
Gramella_flava_JX397931
Gramella_oceani_KC169796
Gramella_planctonica_KC169794
Gramella_gaetbulicola_GQ857650
Gramella_aestuariivivens_KM591916
Gramella_echinicola_AY608409
Gramella_marina_AY753911
Gramella_portivictoriae_DQ002871
Dokdonia_genika_AB198086
Dokdonia_donghaensis_DQ003276
Dokdonia_diaphoros_AB198089
Dokdonia_eikasta_AB198088
Cellulophaga_fucicola_AJ005973
Cellulophaga_lytica_CP002534
Cellulophaga_geojensis_HQ596527
Sediminicola_arcticus_KM576847
Sediminicola_luteus_AB206957
Cellulophaga_tyrosinoxydans_EU443205
Cellulophaga_pacifica_AB100840
Cellulophaga_baltica_AJ005972
Cellulophaga_algicola_AF001366
Arenibacter_nanhaiticus_EU999955
Arenibacter_certesii_AY271622
Arenibacter_latericius_AF052742
Arenibacter_hampyeongensis_JF751052
Arenibacter_echinorum_EF536748
Arenibacter_palladensis_AJ575643
Arenibacter_troitsensis_AB080771
Robiginitalea_biformata_CP001712
Robiginitalea_myxolifaciens_AB270585
Zeaxanthinibacter_enoshimensis_AB264057
Eudoraea_adriatica_AM745437
Muriicola_marianensis_KC839612
Muriicola_jejuensis_EU443206
Maribacter_polysiphoniae_AM497875
Maribacter_arcticus_AY771762
Maribacter_chungangensis_JN036550
Maribacter_antarcticus_EU512921
Pibocella_ponti_AY576654
Maribacter_aquivivus_AY271625
Maribacter_stanieri_EU246691
Maribacter_orientalis_AY271624
Maribacter_forsetii_AM712900
Maribacter_ulvicola_AY271626
Maribacter_dokdonensis_AY960749
Maribacter_sedimenticola_AY271623
Maribacter_aestuarii_JN642273
Maribacter_thermophilus_JQ988061
Costertonia_aggregata_DQ167246
Pricia_antarctica_FJ889677
Zobellia_galactanivorans_AF208293
Zobellia_uliginosa_M62799
Zobellia_russellii_AB121976

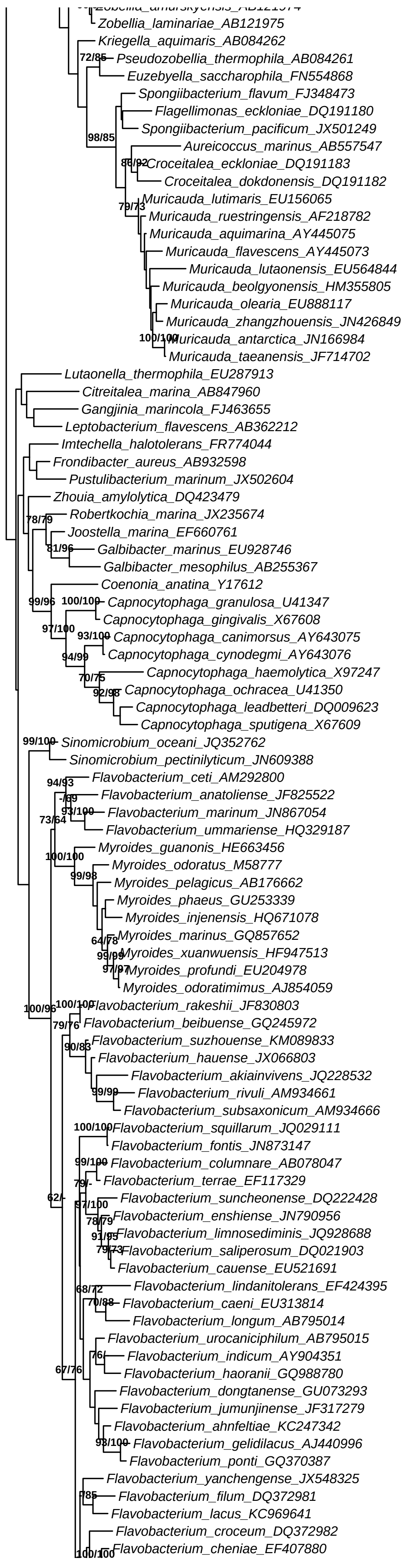

Zobellia_laminariae_AB121975
Kriegella_aquimaris_AB084262
72/85
Pseudozobellia_thermophila_AB084261
Euzebyella_saccharophila_FN554868
Spongiibacterium_flavum_FJ348473
Flagellimonas_eckloniae_DQ191180
Spongiibacterium_pacificum_JX501249
98/85
Aureicoccus_marinus_AB557547
86/92
Croceitalea_eckloniae_DQ191183
Croceitalea_dokdonensis_DQ191182
Muricauda_lutimaris_EU156065
79/73
Muricauda_ruestringensis_AF218782
Muricauda_aquimarina_AY445075
Muricauda_flavescens_AY445073
Muricauda_lutaonensis_EU564844
Muricauda_beolgyonensis_HM355805
Muricauda_olearia_EU888117
Muricauda_zhangzhouensis_JN426849
100/100
Muricauda_antarctica_JN166984
Muricauda_taeanensis_JF714702
Lutaonella_thermophila_EU287913
Citreitalea_marina_AB847960
Gangjinia_marincola_FJ463655
Leptobacterium_flavescens_AB362212
Imtechella_halotolerans_FR774044
Frondibacter_aureus_AB932598
Pustulibacterium_marinum_JX502604
Zhouia_amylolytica_DQ423479
78/79
Robertkochia_marina_JX235674
Joostella_marina_EF660761
81/96
Galbibacter_marinus_EU928746
Galbibacter_mesophilus_AB255367
Coenonia_anatina_Y17612
99/96
100/100
Capnocytophaga_granulosa_U41347
Capnocytophaga_gingivalis_X67608
97/100
93/100
Capnocytophaga_canimorsus_AY643075
94/99
Capnocytophaga_cynodegmi_AY643076
70/75
Capnocytophaga_haemolytica_X97247
92/98
Capnocytophaga_ochracea_U41350
Capnocytophaga_leadbetteri_DQ009623
Capnocytophaga_sputigena_X67609
99/100
Sinomicrobium_oceani_JQ352762
Sinomicrobium_pectinilyticum_JN609388
94/93
Flavobacterium_ceti_AM292800
-/89
Flavobacterium_anatoliense_JF825522
93/100
Flavobacterium_marinum_JN867054
73/64
Flavobacterium_ummariense_HQ329187
Myroides_guanonis_HE663456
100/100
Myroides_odoratus_M58777
99/98
Myroides_pelagicus_AB176662
Myroides_phaeus_GU253339
Myroides_injenensis_HQ671078
64/78
Myroides_marinus_GQ857652
99/99
Myroides_xuanwuensis_HF947513
97/97
Myroides_profundi_EU204978
Myroides_odoratimimus_AJ854059
100/96
100/100
Flavobacterium_rakeshii_JF830803
79/76
Flavobacterium_beibuense_GQ245972
Flavobacterium_suzhouense_KM089833
90/83
Flavobacterium_hauense_JX066803
Flavobacterium_akiainvivens_JQ228532
99/99
Flavobacterium_rivuli_AM934661
Flavobacterium_subsaxonicum_AM934666
100/100
Flavobacterium_squillarum_JQ029111
Flavobacterium_fontis_JN873147
99/100
Flavobacterium_columnare_AB078047
79/-
Flavobacterium_terrae_EF117329
62/-
97/100
Flavobacterium_suncheonense_DQ222428
78/79
Flavobacterium_enshiense_JN790956
91/95
Flavobacterium_limnosediminis_JQ928688
79/73
Flavobacterium_saliperosum_DQ021903
Flavobacterium_cauense_EU521691
68/72
Flavobacterium_lindanitolerans_EF424395
70/88
Flavobacterium_caeni_EU313814
Flavobacterium_longum_AB795014
Flavobacterium_urocaniciphilum_AB795015
76/-
Flavobacterium_indicum_AY904351
67/76
Flavobacterium_haoranii_GQ988780
Flavobacterium_dongtanense_GU073293
Flavobacterium_jumunjinense_JF317279
Flavobacterium_ahnfeltiae_KC247342
93/100
Flavobacterium_gelidilacus_AJ440996
Flavobacterium_ponti_GQ370387
Flavobacterium_yanchengense_JX548325
-/85
Flavobacterium_filum_DQ372981
Flavobacterium_lacus_KC969641
Flavobacterium_croceum_DQ372982
100/100
Flavobacterium_cheniae_EF407880

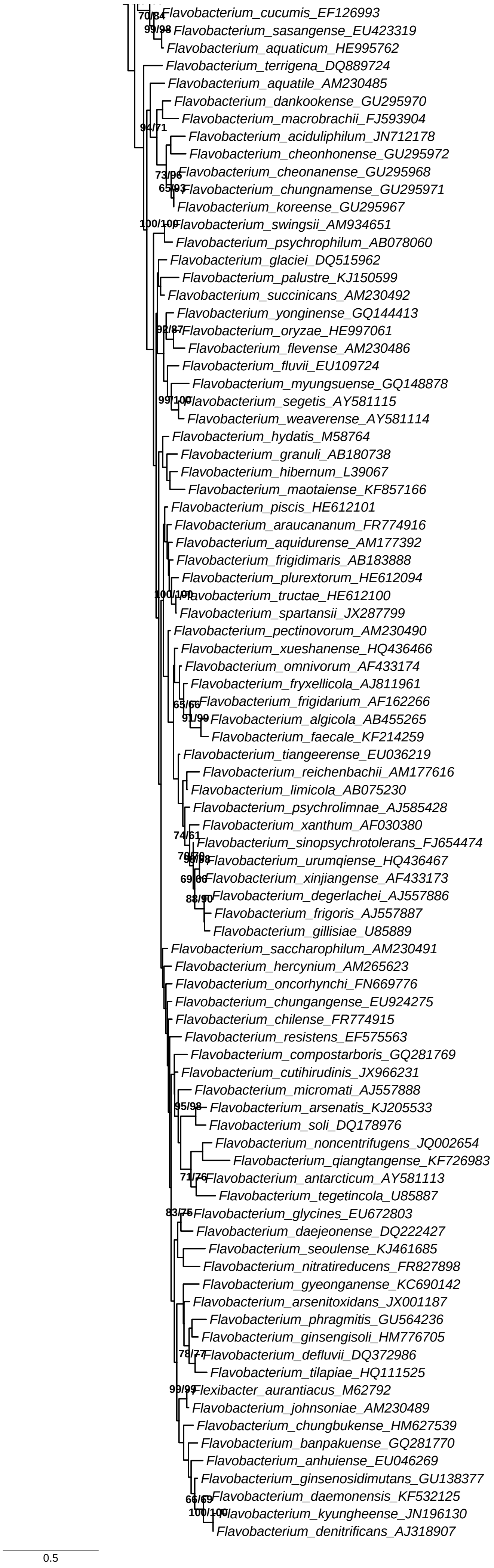
Flavobacterium_cucumis_EF126993
70/84
99/98
Flavobacterium_sasangense_EU423319
Flavobacterium_aquaticum_HE995762
Flavobacterium_terrigena_DQ889724
Flavobacterium_aquatile_AM230485
Flavobacterium_dankookense_GU295970
Flavobacterium_macrobrachii_FJ593904
94/71
Flavobacterium_aciduliphilum_JN712178
Flavobacterium_cheonhonense_GU295972
73/96
Flavobacterium_cheonanense_GU295968
65/93
Flavobacterium_chungnamense_GU295971
Flavobacterium_koreense_GU295967
100/100
Flavobacterium_swingsii_AM934651
Flavobacterium_psychrophilum_AB078060
Flavobacterium_glaciei_DQ515962
Flavobacterium_palustre_KJ150599
Flavobacterium_succinicans_AM230492
Flavobacterium_yonginense_GQ144413
92/87
Flavobacterium_oryzae_HE997061
Flavobacterium_flevense_AM230486
Flavobacterium_fluvii_EU109724
Flavobacterium_myungsuense_GQ148878
99/100
Flavobacterium_segetis_AY581115
Flavobacterium_weaverense_AY581114
Flavobacterium_hydatis_M58764
Flavobacterium_granuli_AB180738
Flavobacterium_hibernum_L39067
Flavobacterium_maotaiense_KF857166
Flavobacterium_piscis_HE612101
Flavobacterium_araucananum_FR774916
Flavobacterium_aquidurense_AM177392
Flavobacterium_frigidimaris_AB183888
Flavobacterium_plurextorum_HE612094
100/100
Flavobacterium_tructae_HE612100
Flavobacterium_spartansii_JX287799
Flavobacterium_pectinovorum_AM230490
Flavobacterium_xueshanense_HQ436466
Flavobacterium_omnivorum_AF433174
Flavobacterium_fryxellicola_AJ811961
65/66
Flavobacterium_frigidarium_AF162266
91/99
Flavobacterium_algicola_AB455265
Flavobacterium_faecale_KF214259
Flavobacterium_tiangeerense_EU036219
Flavobacterium_reichenbachii_AM177616
Flavobacterium_limicola_AB075230
Flavobacterium_psychrolimnae_AJ585428
Flavobacterium_xanthum_AF030380
74/51
Flavobacterium_sinopsychrotolerans_FJ654474
Flavobacterium_urumqiense_HQ436467
69/66
Flavobacterium_xinjiangense_AF433173
88/90
Flavobacterium_degerlachei_AJ557886
Flavobacterium_frigoris_AJ557887
Flavobacterium_gillisiae_U85889
Flavobacterium_saccharophilum_AM230491
Flavobacterium_hercynium_AM265623
Flavobacterium_oncorhynchi_FN669776
Flavobacterium_chungangense_EU924275
Flavobacterium_chilense_FR774915
Flavobacterium_resistens_EF575563
Flavobacterium_compostarboris_GQ281769
Flavobacterium_cutihirudinis_JX966231
Flavobacterium_micromati_AJ557888
95/98
Flavobacterium_arsenatis_KJ205533
Flavobacterium_soli_DQ178976
Flavobacterium_noncentrifugens_JQ002654
Flavobacterium_qiangtangense_KF726983
71/76
Flavobacterium_antarcticum_AY581113
Flavobacterium_tegetincola_U85887
83/75
Flavobacterium_glycines_EU672803
Flavobacterium_daejeonense_DQ222427
Flavobacterium_seoulense_KJ461685
Flavobacterium_nitratireducens_FR827898
Flavobacterium_gyeonganense_KC690142
Flavobacterium_arsenitoxidans_JX001187
Flavobacterium_phragmitis_GU564236
Flavobacterium_ginsengisoli_HM776705
78/77
Flavobacterium_defluvii_DQ372986
Flavobacterium_tilapiae_HQ111525
99/99
Flexibacter_aurantiacus_M62792
Flavobacterium_johnsoniae_AM230489
Flavobacterium_chungbukense_HM627539
Flavobacterium_banpakuense_GQ281770
Flavobacterium_anhuiense_EU046269
Flavobacterium_ginsenosidimutans_GU138377
66/69
Flavobacterium_daemonensis_KF532125
100/100
Flavobacterium_kyungheense_JN196130
Flavobacterium_denitrificans_AJ318907
0.5

Figure 2 - backbone-constrained comprehensive 16S rRNA gene ML and MP tree (CCT)

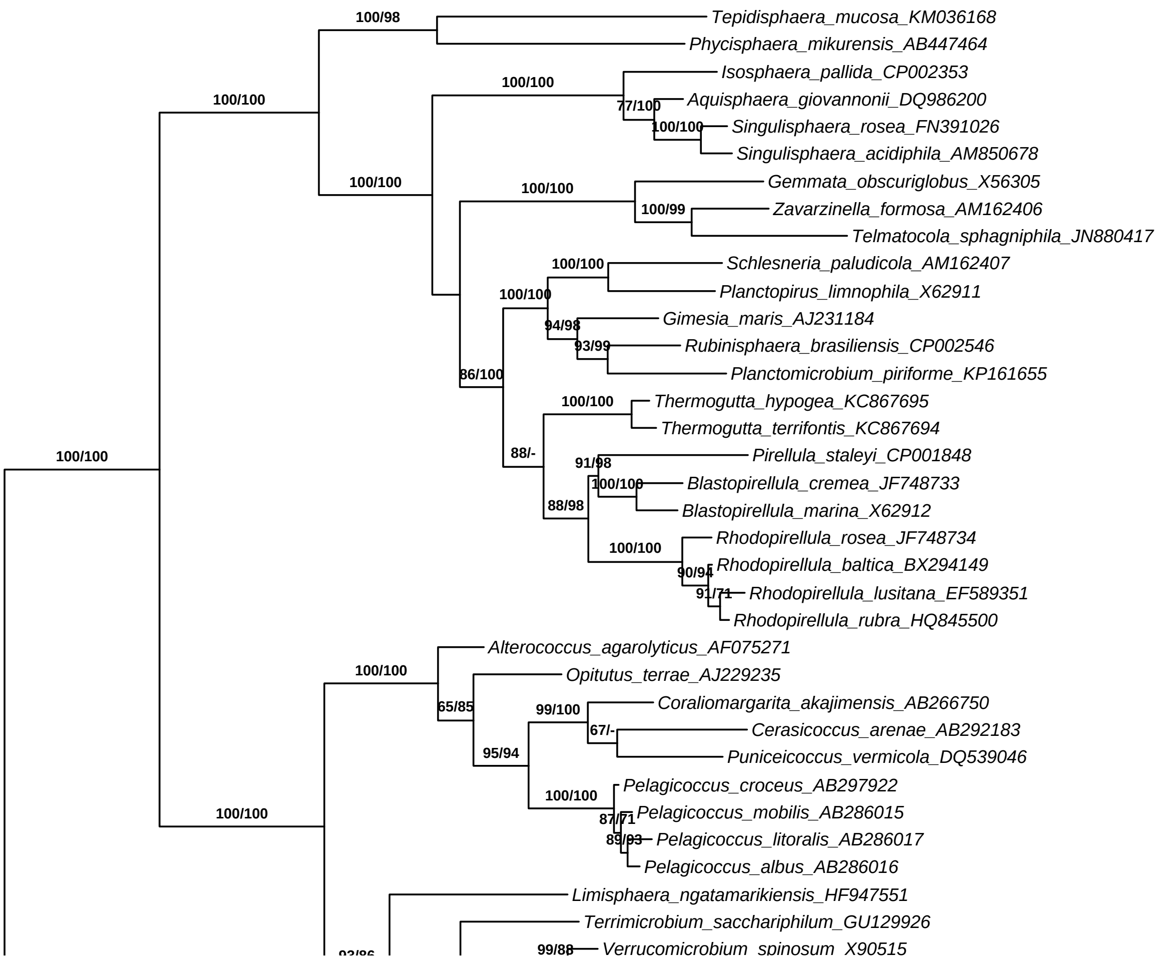

100/98
Tepidisphaera_mucosa_KM036168
Phycisphaera_mikurensis_AB447464
100/100
100/100
Isosphaera_pallida_CP002353
77/100
Aquisphaera_giovannonii_DQ986200
100/100
Singulisphaera_rosea_FN391026
Singulisphaera_acidiphila_AM850678
100/100
100/100
Gemmata_obscuriglobus_X56305
100/99
Zavarzinella_formosa_AM162406
Telmatocola_sphagniphila_JN880417
100/100
Schlesneria_paludicola_AM162407
Planctopirus_limnophila_X62911
100/100
94/98
Gimesia_maris_AJ231184
93/99
Rubinisphaera_brasiliensis_CP002546
Planctomicrobium_piriforme_KP161655
86/100
100/100
Thermogutta_hypogea_KC867695
Thermogutta_terrifontis_KC867694
100/100
88/-
91/98
Pirellula_staleyi_CP001848
100/100
Blastopirellula_cremea_JF748733
Blastopirellula_marina_X62912
88/98
100/100
Rhodopirellula_rosea_JF748734
90/94
Rhodopirellula_baltica_BX294149
91/71
Rhodopirellula_lusitana_EF589351
Rhodopirellula_rubra_HQ845500
Alterococcus_agarolyticus_AF075271
100/100
Opitutus_terrae_AJ229235
65/85
99/100
Coraliomargarita_akajimensis_AB266750
67/-
Cerasicoccus_arenae_AB292183
95/94
Puniceicoccus_vermicola_DQ539046
Pelagicoccus_croceus_AB297922
100/100
Pelagicoccus_mobilis_AB286015
87/71
89/93
Pelagicoccus_litoralis_AB286017
Pelagicoccus_albus_AB286016
100/100
Limisphaera_ngatamarikiensis_HF947551
Terrimicrobium_sacchariphilum_GU129926
93/86
99/88
Verrucomicrobium_spinosum_X90515

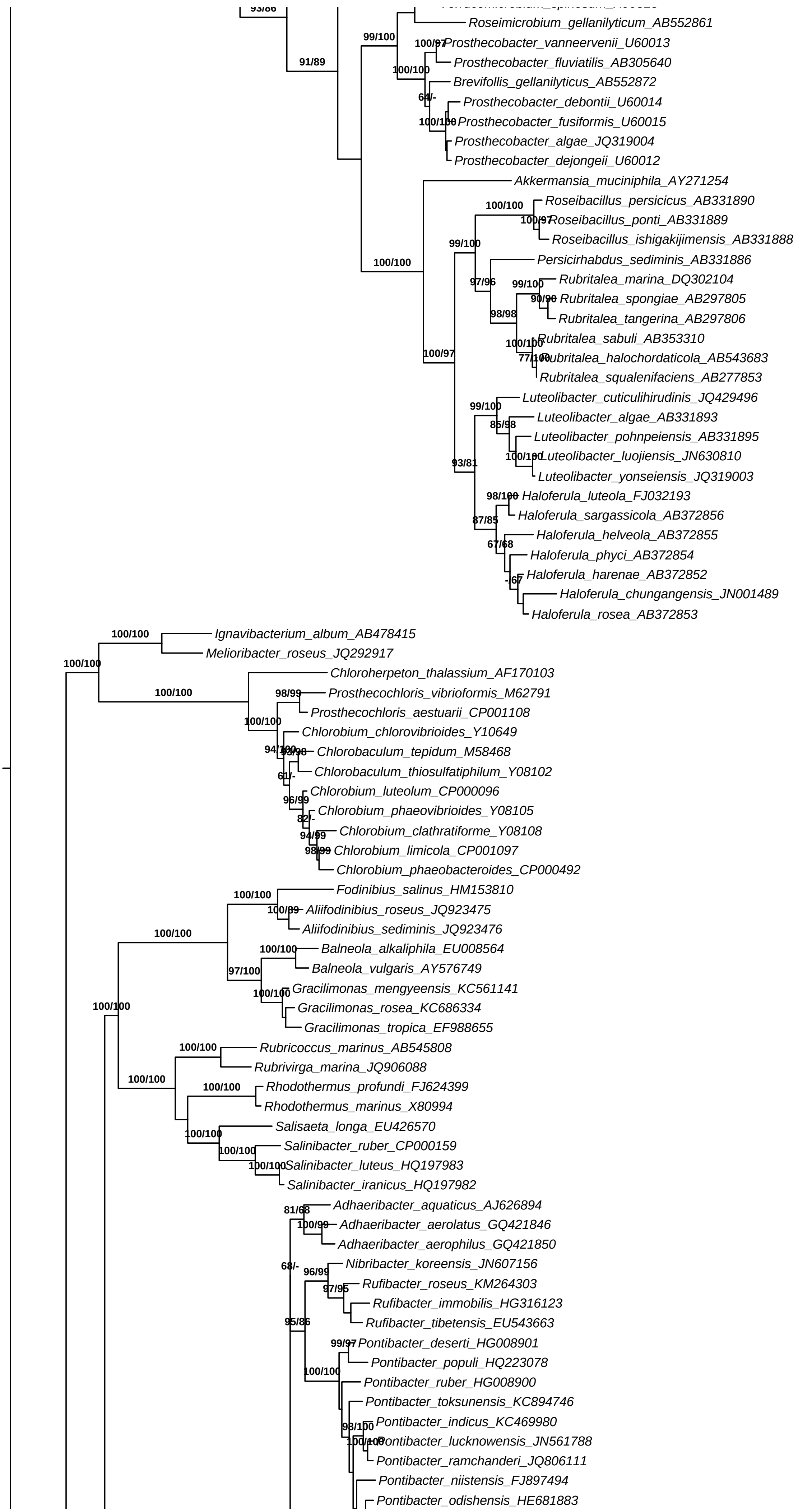
93/86
Roseimicrobium_gellanilyticum_AB552861
99/100
100/97 Prosthecobacter_vanneervenii_U60013
91/89
Prosthecobacter_fluviatilis_AB305640
100/100
Brevifollis_gellanilyticus_AB552872
64/-
Prosthecobacter_debontii_U60014
100/100 Prosthecobacter_fusiformis_U60015
Prosthecobacter_algae_JQ319004
Prosthecobacter_dejongeii_U60012
Akkermansia_muciniphila_AY271254
100/100
Roseibacillus_persicicus_AB331890
100/97 Roseibacillus_ponti_AB331889
99/100
Roseibacillus_ishigakijimensis_AB331888
100/100
Persicirhabdus_sediminis_AB331886
97/96
99/100
Rubritalea_marina_DQ302104
90/90 Rubritalea_spongiae_AB297805
98/98
Rubritalea_tangerina_AB297806
100/100
Rubritalea_sabuli_AB353310
100/97
77/100 Rubritalea_halochordaticola_AB543683
Rubritalea_squalenifaciens_AB277853
Luteolibacter_cuticulihirudinis_JQ429496
99/100
Luteolibacter_algae_AB331893
85/98
Luteolibacter_pohnpeiensis_AB331895
100/100 Luteolibacter_luojiensis_JN630810
93/81
Luteolibacter_yonseiensis_JQ319003
98/100 Haloferula_luteola_FJ032193
Haloferula_sargassicola_AB372856
87/85
Haloferula_helveola_AB372855
67/68
Haloferula_phyci_AB372854
Haloferula_harenae_AB372852
-/67
Haloferula_chungangensis_JN001489
Haloferula_rosea_AB372853
100/100
Ignavibacterium_album_AB478415
100/100
Melioribacter_roseus_JQ292917
Chloroherpeton_thalassium_AF170103
100/100
98/99
Prosthecochloris_vibrioformis_M62791
Prosthecochloris_aestuarii_CP001108
100/100
Chlorobium_chlorovibrioides_Y10649
94/100
93/98
Chlorobaculum_tepidum_M58468
61/-
Chlorobaculum_thiosulfatiphilum_Y08102
Chlorobium_luteolum_CP000096
96/99
Chlorobium_phaeovibrioides_Y08105
82/-
94/99
Chlorobium_clathratiforme_Y08108
98/99 Chlorobium_limicola_CP001097
Chlorobium_phaeobacteroides_CP000492
100/100
Fodinibius_salinus_HM153810
100/89 Aliifodinibius_roseus_JQ923475
100/100
Aliifodinibius_sediminis_JQ923476
100/100
Balneola_alkaliphila_EU008564
97/100
Balneola_vulgaris_AY576749
100/100
Gracilimonas_mengyeensis_KC561141
Gracilimonas_rosea_KC686334
100/100
Gracilimonas_tropica_EF988655
100/100
Rubricoccus_marinus_AB545808
Rubrivirga_marina_JQ906088
100/100
100/100
Rhodothermus_profundi_FJ624399
Rhodothermus_marinus_X80994
Salisaeta_longa_EU426570
100/100
100/100
Salinibacter_ruber_CP000159
100/100 Salinibacter_luteus_HQ197983
Salinibacter_iranicus_HQ197982
81/68
Adhaeribacter_aquaticus_AJ626894
100/99
Adhaeribacter_aerolatus_GQ421846
Adhaeribacter_aerophilus_GQ421850
68/-
Nibribacter_koreensis_JN607156
96/99
Rufibacter_roseus_KM264303
97/95
Rufibacter_immobilis_HG316123
95/86
Rufibacter_tibetensis_EU543663
99/97 Pontibacter_deserti_HG008901
Pontibacter_populi_HQ223078
100/100
Pontibacter_ruber_HG008900
Pontibacter_toksunensis_KC894746
98/100
Pontibacter_indicus_KC469980
100/100 Pontibacter_lucknowensis_JN561788
Pontibacter_ramchanderi_JQ806111
Pontibacter_niistensis_FJ897494
Pontibacter_odishensis_HE681883

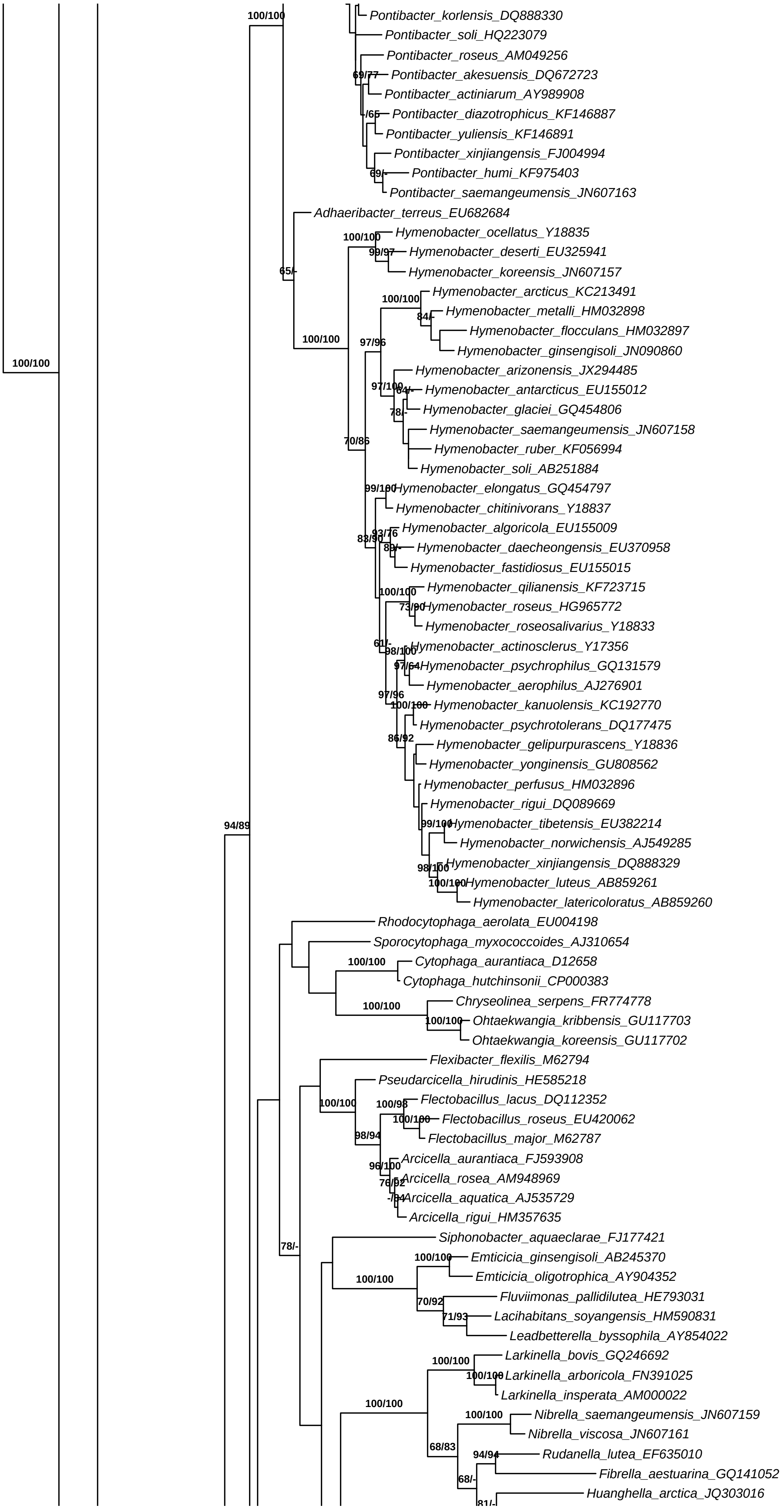
Pontibacter_korlensis_DQ888330
Pontibacter_soli_HQ223079
Pontibacter_roseus_AM049256
Pontibacter_akesuensis_DQ672723
Pontibacter_actiniarum_AY989908
Pontibacter_diazotrophicus_KF146887
Pontibacter_yuliensis_KF146891
Pontibacter_xinjiangensis_FJ004994
Pontibacter_humi_KF975403
Pontibacter_saemangeumensis_JN607163
Adhaeribacter_terreus_EU682684
Hymenobacter_ocellatus_Y18835
Hymenobacter_deserti_EU325941
Hymenobacter_koreensis_JN607157
Hymenobacter_arcticus_KC213491
Hymenobacter_metalli_HM032898
Hymenobacter_flocculans_HM032897
Hymenobacter_ginsengisoli_JN090860
Hymenobacter_arizonensis_JX294485
Hymenobacter_antarcticus_EU155012
Hymenobacter_glaciei_GQ454806
Hymenobacter_saemangeumensis_JN607158
Hymenobacter_ruber_KF056994
Hymenobacter_soli_AB251884
Hymenobacter_elongatus_GQ454797
Hymenobacter_chitinivorans_Y18837
Hymenobacter_algoricola_EU155009
Hymenobacter_daecheongensis_EU370958
Hymenobacter_fastidiosus_EU155015
Hymenobacter_qilianensis_KF723715
Hymenobacter_roseus_HG965772
Hymenobacter_roseosalivarius_Y18833
Hymenobacter_actinosclerus_Y17356
Hymenobacter_psychrophilus_GQ131579
Hymenobacter_aerophilus_AJ276901
Hymenobacter_kanuolensis_KC192770
Hymenobacter_psychrotolerans_DQ177475
Hymenobacter_gelipurpurascens_Y18836
Hymenobacter_yonginensis_GU808562
Hymenobacter_perfusus_HM032896
Hymenobacter_rigui_DQ089669
Hymenobacter_tibetensis_EU382214
Hymenobacter_norwichensis_AJ549285
Hymenobacter_xinjiangensis_DQ888329
Hymenobacter_luteus_AB859261
Hymenobacter_latericoloratus_AB859260
Rhodocytophaga_aerolata_EU004198
Sporocytophaga_myxococcoides_AJ310654
Cytophaga_aurantiaca_D12658
Cytophaga_hutchinsonii_CP000383
Chryseolinea_serpens_FR774778
Ohtaekwangia_kribbensis_GU117703
Ohtaekwangia_koreensis_GU117702
Flexibacter_flexilis_M62794
Pseudarcicella_hirudinis_HE585218
Flectobacillus_lacus_DQ112352
Flectobacillus_roseus_EU420062
Flectobacillus_major_M62787
Arcicella_aurantiaca_FJ593908
Arcicella_rosea_AM948969
Arcicella_aquatica_AJ535729
Arcicella_rigui_HM357635
Siphonobacter_aquaeclarae_FJ177421
Emticicia_ginsengisoli_AB245370
Emticicia_oligotrophica_AY904352
Fluviimonas_pallidilutea_HE793031
Lacihabitans_soyangensis_HM590831
Leadbetterella_byssophila_AY854022
Larkinella_bovis_GQ246692
Larkinella_arboricola_FN391025
Larkinella_insperata_AM000022
Nibrella_saemangeumensis_JN607159
Nibrella_viscosa_JN607161
Rudanella_lutea_EF635010
Fibrella_aestuarina_GQ141052
Huanghella_arctica_JQ303016

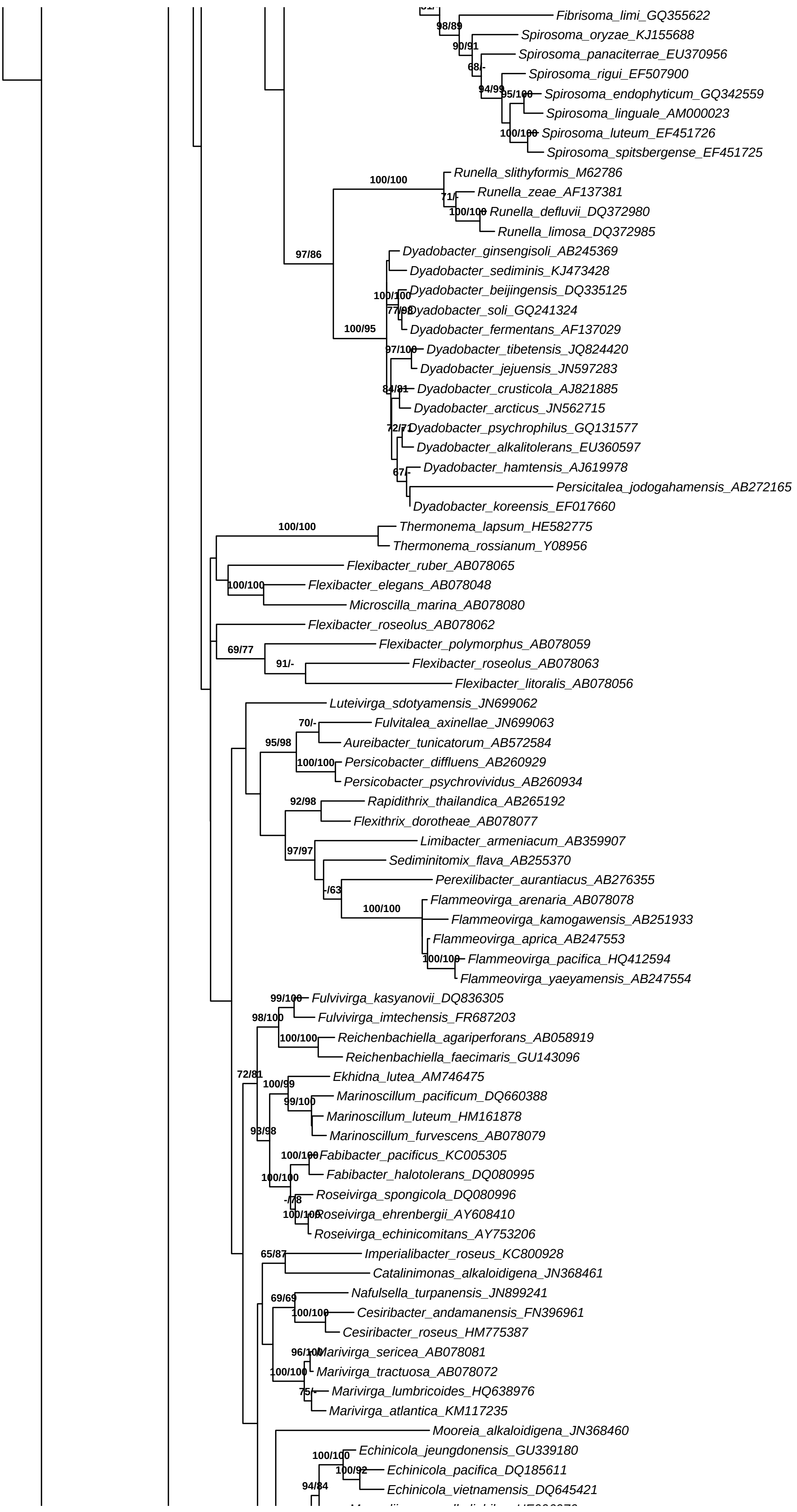

98/89
Fibrisoma_limi_GQ355622
90/91
Spirosoma_oryzae_KJ155688
68/-
Spirosoma_panaciterrae_EU370956
94/99
Spirosoma_rigui_EF507900
95/100
Spirosoma_endophyticum_GQ342559
Spirosoma_linguale_AM000023
100/100
Spirosoma_luteum_EF451726
Spirosoma_spitsbergense_EF451725
100/100
Runella_slithyformis_M62786
71/-
Runella_zeae_AF137381
100/100
Runella_defluvii_DQ372980
Runella_limosa_DQ372985
97/86
Dyadobacter_ginsengisoli_AB245369
Dyadobacter_sediminis_KJ473428
100/100
Dyadobacter_beijingensis_DQ335125
77/98
Dyadobacter_soli_GQ241324
100/95
Dyadobacter_fermentans_AF137029
97/100
Dyadobacter_tibetensis_JQ824420
Dyadobacter_jejuensis_JN597283
84/81
Dyadobacter_crusticola_AJ821885
Dyadobacter_arcticus_JN562715
72/71
Dyadobacter_psychrophilus_GQ131577
Dyadobacter_alkalitolerans_EU360597
67/-
Dyadobacter_hamtensis_AJ619978
Persicitalea_jodogahamensis_AB272165
Dyadobacter_koreensis_EF017660
100/100
Thermonema_lapsum_HE582775
Thermonema_rossianum_Y08956
Flexibacter_ruber_AB078065
100/100
Flexibacter_elegans_AB078048
Microscilla_marina_AB078080
Flexibacter_roseolus_AB078062
69/77
Flexibacter_polymorphus_AB078059
91/-
Flexibacter_roseolus_AB078063
Flexibacter_litoralis_AB078056
Luteivirga_sdotyamensis_JN699062
70/-
Fulvitalea_axinellae_JN699063
95/98
Aureibacter_tunicatorum_AB572584
100/100
Persicobacter_diffluens_AB260929
Persicobacter_psychrovividus_AB260934
92/98
Rapidithrix_thailandica_AB265192
Flexithrix_dorotheae_AB078077
97/97
Limibacter_armeniacum_AB359907
Sediminitomix_flava_AB255370
-/63
Perexilibacter_aurantiacus_AB276355
100/100
Flammeovirga_arenaria_AB078078
Flammeovirga_kamogawensis_AB251933
Flammeovirga_aprica_AB247553
100/100
Flammeovirga_pacifica_HQ412594
Flammeovirga_yaeyamensis_AB247554
99/100
Fulvivirga_kasyanovii_DQ836305
98/100
Fulvivirga_imtechensis_FR687203
100/100
Reichenbachiella_agariperforans_AB058919
Reichenbachiella_faecimaris_GU143096
72/81
100/99
Ekhidna_lutea_AM746475
99/100
Marinoscillum_pacificum_DQ660388
Marinoscillum_luteum_HM161878
98/98
Marinoscillum_furvescens_AB078079
100/100
Fabibacter_pacificus_KC005305
100/100
Fabibacter_halotolerans_DQ080995
-/78
Roseivirga_spongicola_DQ080996
100/100
Roseivirga_ehrenbergii_AY608410
Roseivirga_echinicomitans_AY753206
65/87
Imperialibacter_roseus_KC800928
Catalinimonas_alkaloidigena_JN368461
69/69
Nafulsella_turpanensis_JN899241
100/100
Cesiribacter_andamanensis_FN396961
Cesiribacter_roseus_HM775387
96/100
Marivirga_sericea_AB078081
100/100
Marivirga_tractuosa_AB078072
75/-
Marivirga_lumbricoides_HQ638976
Marivirga_atlantica_KM117235
Mooreia_alkaloidigena_JN368460
100/100
Echinicola_jeungdonensis_GU339180
100/92
Echinicola_pacifica_DQ185611
94/84
Echinicola_vietnamensis_DQ645421

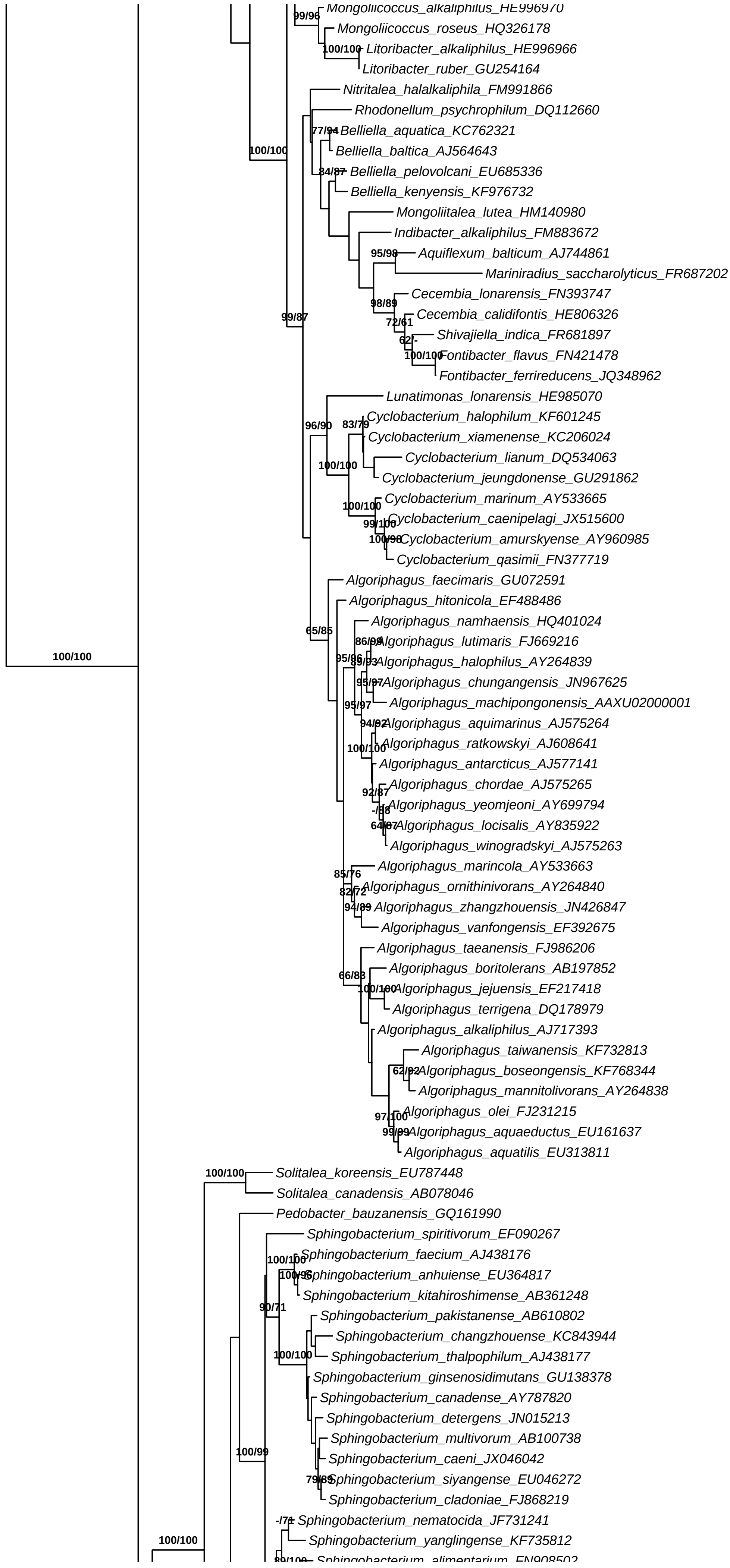
Mongoliicoccus_alkaliphilus_HE996970
99/96
Mongoliicoccus_roseus_HQ326178
100/100
Litoribacter_alkaliphilus_HE996966
Litoribacter_ruber_GU254164
Nitritalea_halalkaliphila_FM991866
Rhodonellum_psychrophilum_DQ112660
77/94
Belliella_aquatica_KC762321
Belliella_baltica_AJ564643
100/100
Belliella_pelovolcani_EU685336
84/87
Belliella_kenyensis_KF976732
Mongoliitalea_lutea_HM140980
Indibacter_alkaliphilus_FM883672
95/98
Aquiflexum_balticum_AJ744861
Mariniradius_saccharolyticus_FR687202
Cecembia_lonarensis_FN393747
98/89
Cecembia_calidifontis_HE806326
99/87
72/61
Shivajiella_indica_FR681897
62/-
100/100
Fontibacter_flavus_FN421478
Fontibacter_ferrireducens_JQ348962
Lunatimonas_lonarensis_HE985070
Cyclobacterium_halophilum_KF601245
96/90
83/79
Cyclobacterium_xiamenense_KC206024
Cyclobacterium_lianum_DQ534063
100/100
Cyclobacterium_jeungdonense_GU291862
Cyclobacterium_marinum_AY533665
100/100
Cyclobacterium_caenipelagi_JX515600
99/100
100/98
Cyclobacterium_amurskyense_AY960985
Cyclobacterium_qasimii_FN377719
Algoriphagus_faecimaris_GU072591
Algoriphagus_hitonicola_EF488486
Algoriphagus_namhaensis_HQ401024
65/85
86/99
Algoriphagus_lutimaris_FJ669216
100/100
95/96
89/93
Algoriphagus_halophilus_AY264839
95/97
Algoriphagus_chungangensis_JN967625
Algoriphagus_machipongonensis_AAXU02000001
95/97
94/92
Algoriphagus_aquimarinus_AJ575264
Algoriphagus_ratkowskyi_AJ608641
100/100
Algoriphagus_antarcticus_AJ577141
Algoriphagus_chordae_AJ575265
92/87
Algoriphagus_yeomjeoni_AY699794
-/88
Algoriphagus_locisalis_AY835922
64/87
Algoriphagus_winogradskyi_AJ575263
Algoriphagus_marincola_AY533663
85/76
Algoriphagus_ornithinivorans_AY264840
82/72
Algoriphagus_zhangzhouensis_JN426847
94/89
Algoriphagus_vanfongensis_EF392675
Algoriphagus_taeanensis_FJ986206
Algoriphagus_boritolerans_AB197852
66/83
100/100
Algoriphagus_jejuensis_EF217418
Algoriphagus_terrigena_DQ178979
Algoriphagus_alkaliphilus_AJ717393
Algoriphagus_taiwanensis_KF732813
62/92
Algoriphagus_boseongensis_KF768344
Algoriphagus_mannitolivorans_AY264838
Algoriphagus_olei_FJ231215
97/100
99/99
Algoriphagus_aquaeductus_EU161637
Algoriphagus_aquatilis_EU313811
100/100
Solitalea_koreensis_EU787448
Solitalea_canadensis_AB078046
Pedobacter_bauzanensis_GQ161990
Sphingobacterium_spiritivorum_EF090267
Sphingobacterium_faecium_AJ438176
100/100
100/96
Sphingobacterium_anhuiense_EU364817
Sphingobacterium_kitahiroshimense_AB361248
90/71
Sphingobacterium_pakistanense_AB610802
Sphingobacterium_changzhouense_KC843944
100/100
Sphingobacterium_thalpophilum_AJ438177
Sphingobacterium_ginsenosidimutans_GU138378
Sphingobacterium_canadense_AY787820
Sphingobacterium_detergens_JN015213
Sphingobacterium_multivorum_AB100738
100/99
Sphingobacterium_caeni_JX046042
79/89
Sphingobacterium_siyangense_EU046272
Sphingobacterium_cladoniae_FJ868219
-/71
Sphingobacterium_nematocida_JF731241
Sphingobacterium_yanglingense_KF735812
100/100
89/100
Sphingobacterium_alimentarium_FN908502

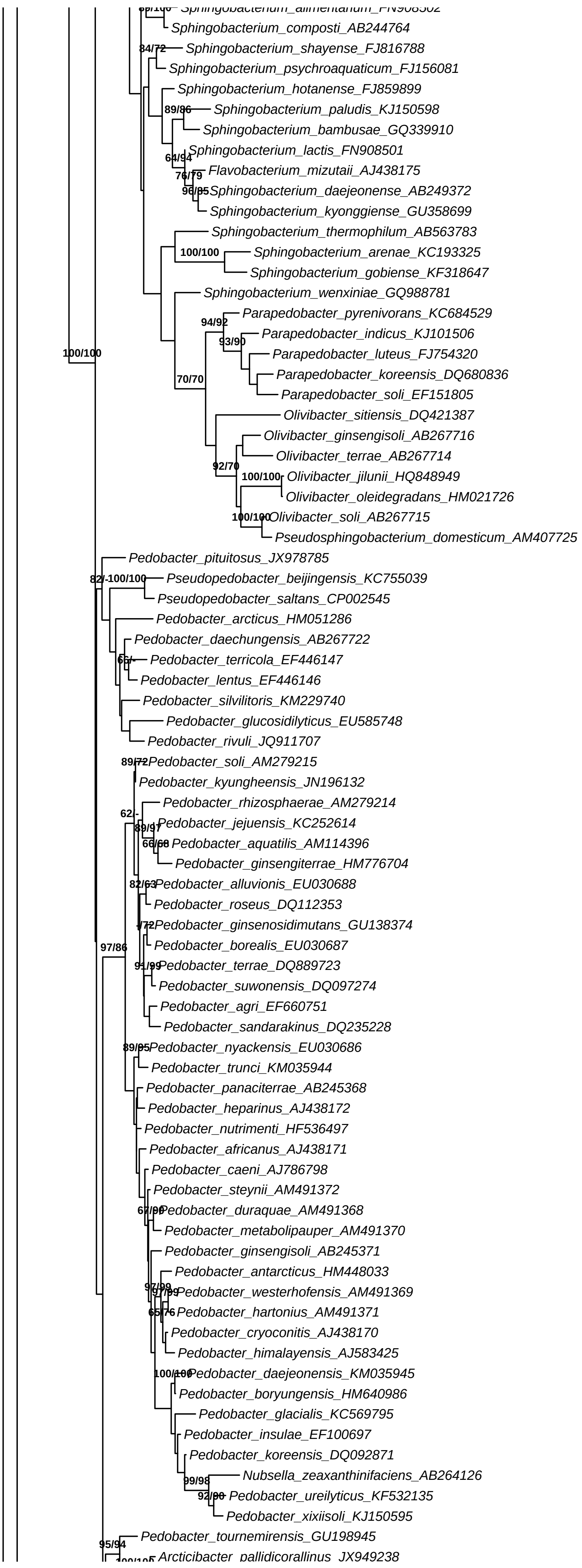

Sphingobacterium_composti_AB244764
84/72
Sphingobacterium_shayense_FJ816788
Sphingobacterium_psychroaquaticum_FJ156081
Sphingobacterium_hotanense_FJ859899
89/86
Sphingobacterium_paludis_KJ150598
Sphingobacterium_bambusae_GQ339910
Sphingobacterium_lactis_FN908501
64/94
Flavobacterium_mizutaii_AJ438175
76/79
Sphingobacterium_daejeonense_AB249372
Sphingobacterium_kyonggiense_GU358699
Sphingobacterium_thermophilum_AB563783
100/100
Sphingobacterium_arenae_KC193325
Sphingobacterium_gobiense_KF318647
Sphingobacterium_wenxiniae_GQ988781
Parapedobacter_pyrenivorans_KC684529
94/92
Parapedobacter_indicus_KJ101506
93/90
Parapedobacter_luteus_FJ754320
100/100
Parapedobacter_koreensis_DQ680836
Parapedobacter_soli_EF151805
70/70
Olivibacter_sitiensis_DQ421387
Olivibacter_ginsengisoli_AB267716
Olivibacter_terrae_AB267714
92/70
100/100
Olivibacter_jilunii_HQ848949
Olivibacter_oleidegradans_HM021726
100/100
Olivibacter_soli_AB267715
Pseudosphingobacterium_domesticum_AM407725
Pedobacter_pituitosus_JX978785
82/-
100/100
Pseudopedobacter_beijingensis_KC755039
Pseudopedobacter_saltans_CP002545
Pedobacter_arcticus_HM051286
Pedobacter_daechungensis_AB267722
Pedobacter_terricola_EF446147
Pedobacter_lentus_EF446146
Pedobacter_silvilitoris_KM229740
Pedobacter_glucosidilyticus_EU585748
Pedobacter_rivuli_JQ911707
89/72
Pedobacter_soli_AM279215
Pedobacter_kyungheensis_JN196132
Pedobacter_rhizosphaerae_AM279214
62/-
Pedobacter_jejuensis_KC252614
89/97
Pedobacter_aquatilis_AM114396
Pedobacter_ginsengiterrae_HM776704
82/63
Pedobacter_alluvionis_EU030688
Pedobacter_roseus_DQ112353
Pedobacter_ginsenosidimutans_GU138374
Pedobacter_borealis_EU030687
97/86
Pedobacter_terrae_DQ889723
Pedobacter_suwonensis_DQ097274
Pedobacter_agri_EF660751
Pedobacter_sandarakinus_DQ235228
Pedobacter_nyackensis_EU030686
Pedobacter_trunci_KM035944
Pedobacter_panaciterrae_AB245368
Pedobacter_heparinus_AJ438172
Pedobacter_nutrimenti_HF536497
Pedobacter_africanus_AJ438171
Pedobacter_caeni_AJ786798
Pedobacter_steynii_AM491372
Pedobacter_duraquae_AM491368
Pedobacter_metabolipauper_AM491370
Pedobacter_ginsengisoli_AB245371
Pedobacter_antarcticus_HM448033
97/99
Pedobacter_westerhofensis_AM491369
65/76
Pedobacter_hartonius_AM491371
Pedobacter_cryoconitis_AJ438170
Pedobacter_himalayensis_AJ583425
Pedobacter_daejeonensis_KM035945
Pedobacter_boryungensis_HM640986
Pedobacter_glacialis_KC569795
Pedobacter_insulae_EF100697
Pedobacter_koreensis_DQ092871
Nubsella_zeaxanthinifaciens_AB264126
99/98
Pedobacter_ureilyticus_KF532135
92/90
Pedobacter_xixiisoli_KJ150595
Pedobacter_tournemirensis_GU198945
95/94
Arcticibacter_pallidicorallinus_JX949238

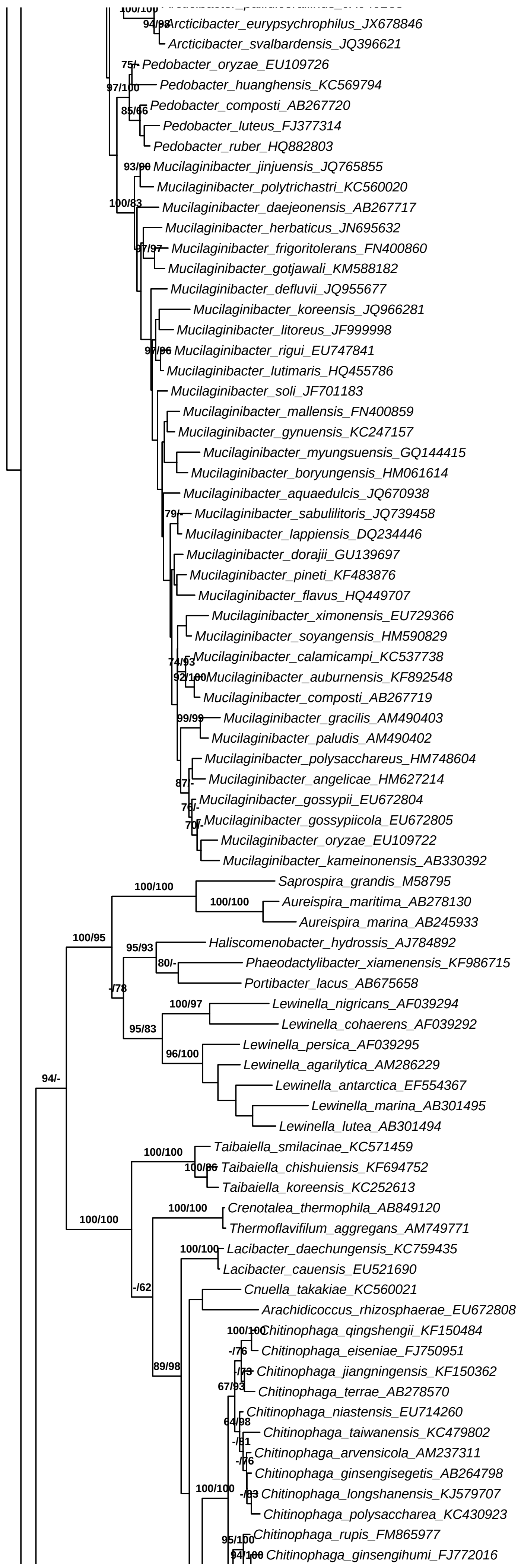
Arcticibacter_eurypsychrophilus_JX678846
Arcticibacter_svalbardensis_JQ396621
Pedobacter_oryzae_EU109726
Pedobacter_huanghensis_KC569794
Pedobacter_composti_AB267720
Pedobacter_luteus_FJ377314
Pedobacter_ruber_HQ882803
Mucilaginibacter_jinjuensis_JQ765855
Mucilaginibacter_polytrichastri_KC560020
Mucilaginibacter_daejeonensis_AB267717
Mucilaginibacter_herbaticus_JN695632
Mucilaginibacter_frigoritolerans_FN400860
Mucilaginibacter_gotjawali_KM588182
Mucilaginibacter_defluvii_JQ955677
Mucilaginibacter_koreensis_JQ966281
Mucilaginibacter_litoreus_JF999998
Mucilaginibacter_rigui_EU747841
Mucilaginibacter_lutimaris_HQ455786
Mucilaginibacter_soli_JF701183
Mucilaginibacter_mallensis_FN400859
Mucilaginibacter_gynuensis_KC247157
Mucilaginibacter_myungsuensis_GQ144415
Mucilaginibacter_boryungensis_HM061614
Mucilaginibacter_aquaedulcis_JQ670938
Mucilaginibacter_sabulilitoris_JQ739458
Mucilaginibacter_lappiensis_DQ234446
Mucilaginibacter_dorajii_GU139697
Mucilaginibacter_pineti_KF483876
Mucilaginibacter_flavus_HQ449707
Mucilaginibacter_ximonensis_EU729366
Mucilaginibacter_soyangensis_HM590829
Mucilaginibacter_calamicampi_KC537738
Mucilaginibacter_auburnensis_KF892548
Mucilaginibacter_composti_AB267719
Mucilaginibacter_gracilis_AM490403
Mucilaginibacter_paludis_AM490402
Mucilaginibacter_polysacchareus_HM748604
Mucilaginibacter_angelicae_HM627214
Mucilaginibacter_gossypii_EU672804
Mucilaginibacter_gossypiicola_EU672805
Mucilaginibacter_oryzae_EU109722
Mucilaginibacter_kameinonensis_AB330392
Saprospira_grandis_M58795
Aureispira_maritima_AB278130
Aureispira_marina_AB245933
Haliscomenobacter_hydrossis_AJ784892
Phaeodactylibacter_xiamenensis_KF986715
Portibacter_lacus_AB675658
Lewinella_nigricans_AF039294
Lewinella_cohaerens_AF039292
Lewinella_persica_AF039295
Lewinella_agarilytica_AM286229
Lewinella_antarctica_EF554367
Lewinella_marina_AB301495
Lewinella_lutea_AB301494
Taibaiella_smilacinae_KC571459
Taibaiella_chishuiensis_KF694752
Taibaiella_koreensis_KC252613
Crenotalea_thermophila_AB849120
Thermoflavifilum_aggregans_AM749771
Lacibacter_daechungensis_KC759435
Lacibacter_cauensis_EU521690
Cnuella_takakiae_KC560021
Arachidicoccus_rhizosphaerae_EU672808
Chitinophaga_qingshengii_KF150484
Chitinophaga_eiseniae_FJ750951
Chitinophaga_jiangningensis_KF150362
Chitinophaga_terrae_AB278570
Chitinophaga_niastensis_EU714260
Chitinophaga_taiwanensis_KC479802
Chitinophaga_arvensicola_AM237311
Chitinophaga_ginsengisegetis_AB264798
Chitinophaga_longshanensis_KJ579707
Chitinophaga_polysaccharea_KC430923
Chitinophaga_rupis_FM865977
Chitinophaga_ginsengihumi_FJ772016

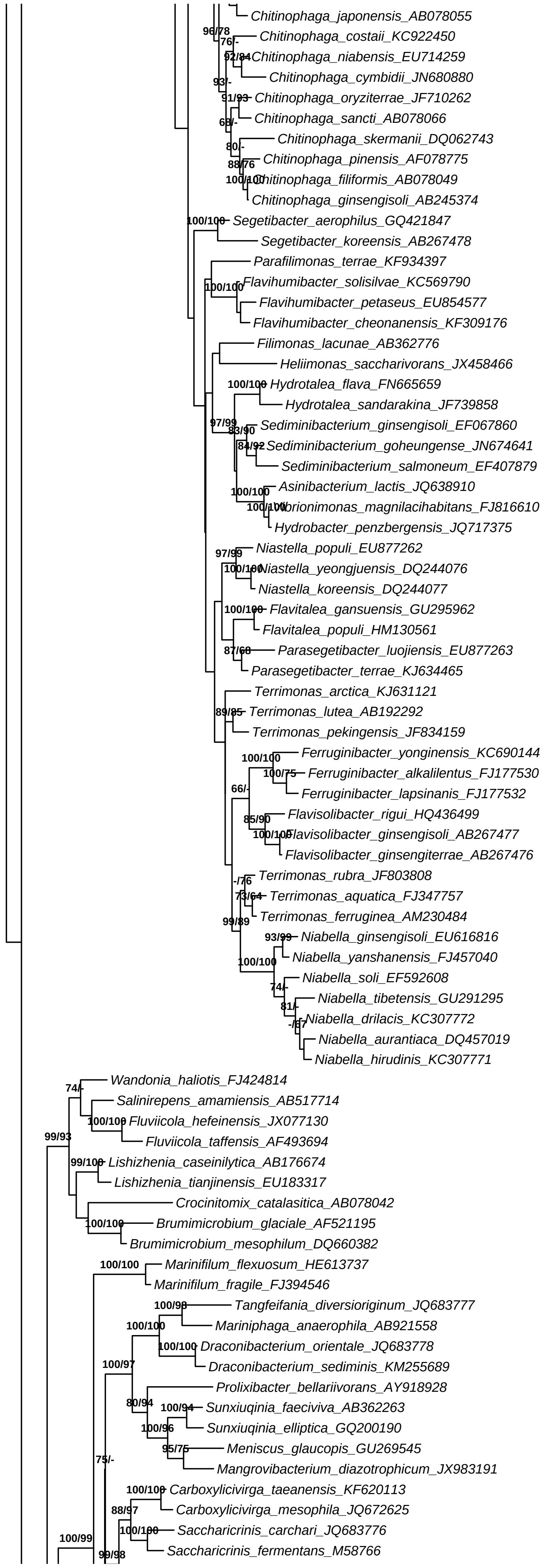
Chitinophaga_japonensis_AB078055
96/78
76/-
Chitinophaga_costaii_KC922450
92/84
Chitinophaga_niabensis_EU714259
Chitinophaga_cymbidii_JN680880
93/-
91/93
Chitinophaga_oryziterrae_JF710262
68/-
Chitinophaga_sancti_AB078066
Chitinophaga_skermanii_DQ062743
80/-
88/76
Chitinophaga_pinensis_AF078775
100/100
Chitinophaga_filiformis_AB078049
Chitinophaga_ginsengisoli_AB245374
100/100
Segetibacter_aerophilus_GQ421847
Segetibacter_koreensis_AB267478
Parafilimonas_terrae_KF934397
100/100
Flavihumibacter_solisilvae_KC569790
Flavihumibacter_petaseus_EU854577
Flavihumibacter_cheonanensis_KF309176
Filimonas_lacunae_AB362776
Heliimonas_saccharivorans_JX458466
100/100
Hydrotalea_flava_FN665659
Hydrotalea_sandarakina_JF739858
97/99
83/90
Sediminibacterium_ginsengisoli_EF067860
84/92
Sediminibacterium_goheungense_JN674641
Sediminibacterium_salmoneum_EF407879
100/100
Asinibacterium_lactis_JQ638910
100/100
Vibrionimonas_magnilacihabitans_FJ816610
Hydrobacter_penzbergensis_JQ717375
97/99
Niastella_populi_EU877262
100/100
Niastella_yeongjuensis_DQ244076
Niastella_koreensis_DQ244077
100/100
Flavitalea_gansuensis_GU295962
Flavitalea_populi_HM130561
87/68
Parasegetibacter_luojiensis_EU877263
Parasegetibacter_terrae_KJ634465
Terrimonas_arctica_KJ631121
89/85
Terrimonas_lutea_AB192292
Terrimonas_pekingensis_JF834159
100/100
Ferruginibacter_yonginensis_KC690144
100/75
Ferruginibacter_alkalilentus_FJ177530
66/-
Ferruginibacter_lapsinanis_FJ177532
85/90
Flavisolibacter_rigui_HQ436499
100/100
Flavisolibacter_ginsengisoli_AB267477
Flavisolibacter_ginsengiterrae_AB267476
-/76
Terrimonas_rubra_JF803808
73/64
Terrimonas_aquatica_FJ347757
99/89
Terrimonas_ferruginea_AM230484
93/99
Niabella_ginsengisoli_EU616816
100/100
Niabella_yanshanensis_FJ457040
74/-
Niabella_soli_EF592608
81/-
Niabella_tibetensis_GU291295
-/87
Niabella_drilacis_KC307772
Niabella_aurantiaca_DQ457019
Niabella_hirudinis_KC307771
74/-
Wandonia_haliotis_FJ424814
Salinirepens_amamiensis_AB517714
100/100
Fluviicola_hefeinensis_JX077130
99/93
Fluviicola_taffensis_AF493694
99/100
Lishizhenia_caseinilytica_AB176674
Lishizhenia_tianjinensis_EU183317
Crocinitomix_catalasitica_AB078042
100/100
Brumimicrobium_glaciale_AF521195
Brumimicrobium_mesophilum_DQ660382
100/100
Marinifilum_flexuosum_HE613737
Marinifilum_fragile_FJ394546
100/98
Tangfeifania_diversioriginum_JQ683777
100/100
Mariniphaga_anaerophila_AB921558
100/100
Draconibacterium_orientale_JQ683778
100/97
Draconibacterium_sediminis_KM255689
Prolixibacter_bellariivorans_AY918928
80/94
100/94
Sunxiuqinia_faeciviva_AB362263
100/96
Sunxiuqinia_elliptica_GQ200190
95/75
Meniscus_glaucopis_GU269545
75/-
Mangrovibacterium_diazotrophicum_JX983191
100/100
Carboxylicivirga_taeanensis_KF620113
88/97
Carboxylicivirga_mesophila_JQ672625
100/100
Saccharicrinis_carchari_JQ683776
100/99
99/98
Saccharicrinis_fermentans_M58766

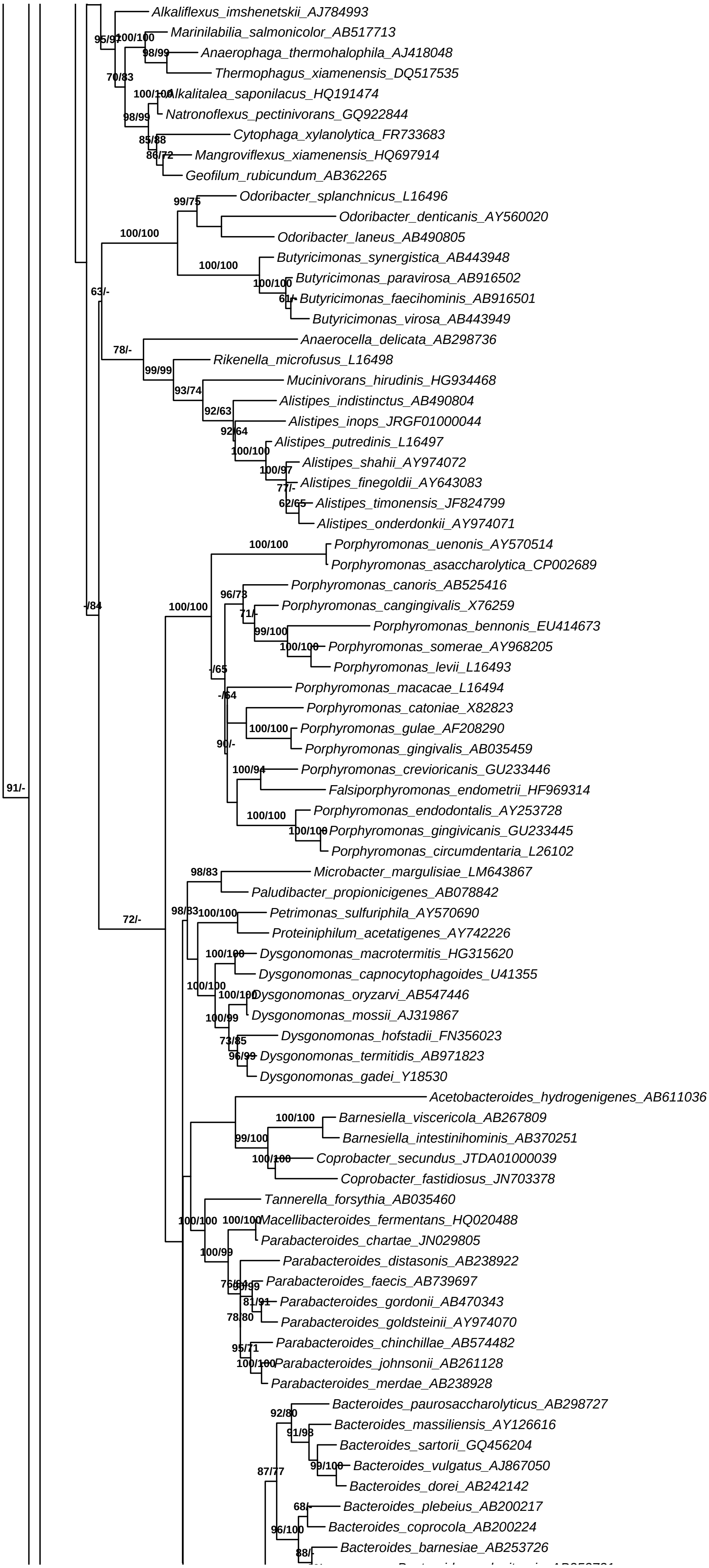
Alkaliflexus_imshenetskii_AJ784993
Marinilabilia_salmonicolor_AB517713
Anaerophaga_thermohalophila_AJ418048
Thermophagus_xiamenensis_DQ517535
Alkalitalea_saponilacus_HQ191474
Natronoflexus_pectinivorans_GQ922844
Cytophaga_xylanolytica_FR733683
Mangroviflexus_xiamenensis_HQ697914
Geofilum_rubicundum_AB362265
Odoribacter_splanchnicus_L16496
Odoribacter_denticanis_AY560020
Odoribacter_laneus_AB490805
Butyricimonas_synergistica_AB443948
Butyricimonas_paravirosa_AB916502
Butyricimonas_faecihominis_AB916501
Butyricimonas_virosa_AB443949
Anaerocella_delicata_AB298736
Rikenella_microfusus_L16498
Mucinivorans_hirudinis_HG934468
Alistipes_indistinctus_AB490804
Alistipes_inops_JRGF01000044
Alistipes_putredinis_L16497
Alistipes_shahii_AY974072
Alistipes_finegoldii_AY643083
Alistipes_timonensis_JF824799
Alistipes_onderdonkii_AY974071
Porphyromonas_uenonis_AY570514
Porphyromonas_asaccharolytica_CP002689
Porphyromonas_canoris_AB525416
Porphyromonas_cangingivalis_X76259
Porphyromonas_bennonis_EU414673
Porphyromonas_somerae_AY968205
Porphyromonas_levii_L16493
Porphyromonas_macacae_L16494
Porphyromonas_catoniae_X82823
Porphyromonas_gulae_AF208290
Porphyromonas_gingivalis_AB035459
Porphyromonas_crevioricanis_GU233446
Falsiporphyromonas_endometrii_HF969314
Porphyromonas_endodontalis_AY253728
Porphyromonas_gingivicanis_GU233445
Porphyromonas_circumdentaria_L26102
Microbacter_margulisiae_LM643867
Paludibacter_propionicigenes_AB078842
Petrimonas_sulfuriphila_AY570690
Proteiniphilum_acetatigenes_AY742226
Dysgonomonas_macrotermitis_HG315620
Dysgonomonas_capnocytophagoides_U41355
Dysgonomonas_oryzarvi_AB547446
Dysgonomonas_mossii_AJ319867
Dysgonomonas_hofstadii_FN356023
Dysgonomonas_termitidis_AB971823
Dysgonomonas_gadei_Y18530
Acetobacteroides_hydrogenigenes_AB611036
Barnesiella_viscericola_AB267809
Barnesiella_intestinihominis_AB370251
Coprobacter_secundus_JTDA01000039
Coprobacter_fastidiosus_JN703378
Tannerella_forsythia_AB035460
Macellibacteroides_fermentans_HQ020488
Parabacteroides_chartae_JN029805
Parabacteroides_distasonis_AB238922
Parabacteroides_faecis_AB739697
Parabacteroides_gordonii_AB470343
Parabacteroides_goldsteinii_AY974070
Parabacteroides_chinchillae_AB574482
Parabacteroides_johnsonii_AB261128
Parabacteroides_merdae_AB238928
Bacteroides_paurosaccharolyticus_AB298727
Bacteroides_massiliensis_AY126616
Bacteroides_sartorii_GQ456204
Bacteroides_vulgatus_AJ867050
Bacteroides_dorei_AB242142
Bacteroides_plebeius_AB200217
Bacteroides_coprocola_AB200224
Bacteroides_barnesiae_AB253726
95/97
100/100
98/99
70/83
100/100
98/99
85/88
86/72
99/75
100/100
100/100
100/100
61/-
63/-
78/-
99/99
93/74
92/63
92/64
100/100
100/97
77/-
62/65
100/100
96/73
100/100
71/-
99/100
100/100
-/65
-/84
-/64
100/100
90/-
100/94
100/100
100/100
91/-
98/83
98/83
100/100
72/-
100/100
100/100
100/100
100/99
73/85
96/99
100/100
99/100
100/100
100/100
100/100
100/99
76/64
90/99
81/91
78/80
95/71
100/100
92/80
91/93
99/100
87/77
68/-
96/100
88/-

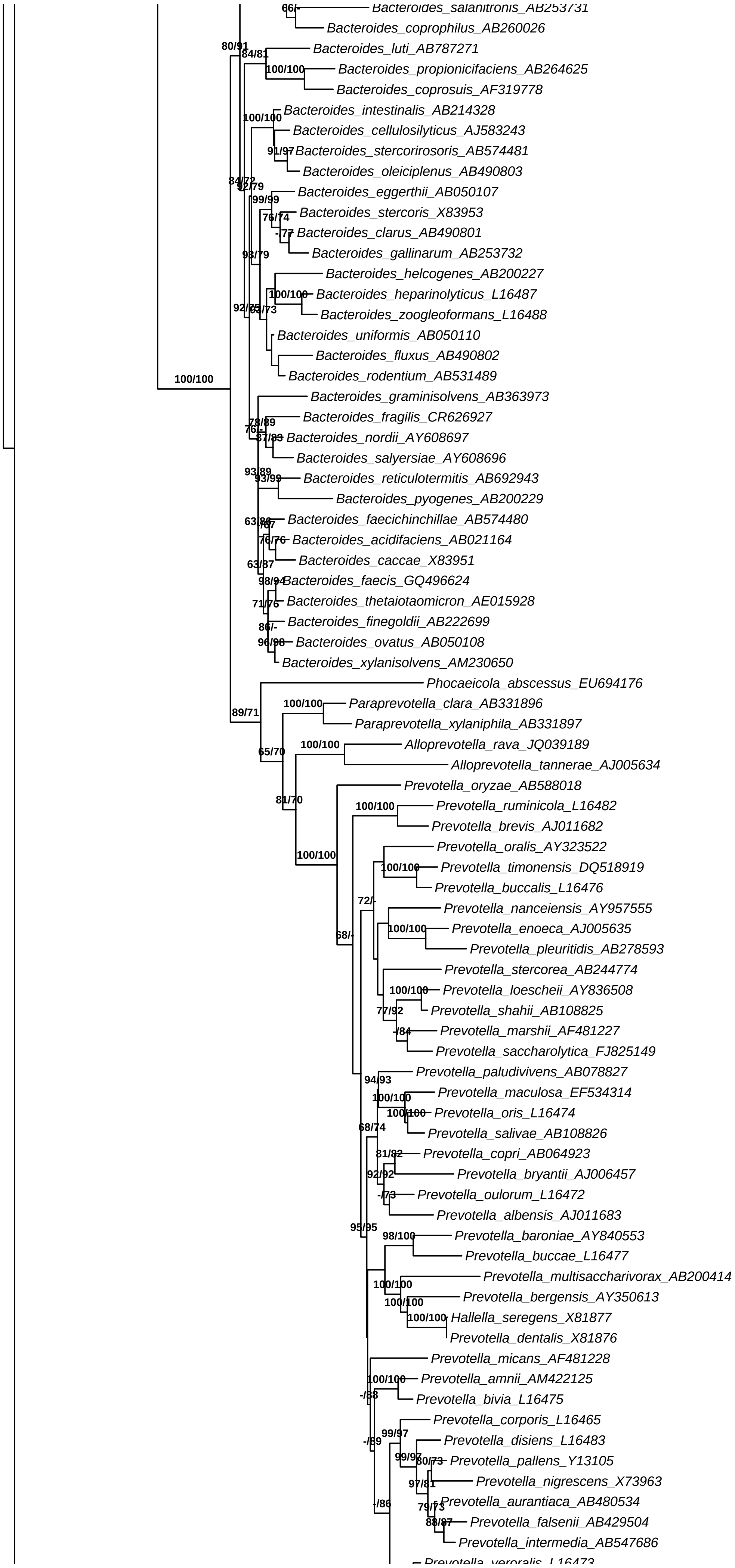
Bacteroides_salanitronis_AB253731
Bacteroides_coprophilus_AB260026
Bacteroides_luti_AB787271
Bacteroides_propionicifaciens_AB264625
Bacteroides_coprosuis_AF319778
Bacteroides_intestinalis_AB214328
Bacteroides_cellulosilyticus_AJ583243
Bacteroides_stercorirosoris_AB574481
Bacteroides_oleiciplenus_AB490803
Bacteroides_eggerthii_AB050107
Bacteroides_stercoris_X83953
Bacteroides_clarus_AB490801
Bacteroides_gallinarum_AB253732
Bacteroides_helcogenes_AB200227
Bacteroides_heparinolyticus_L16487
Bacteroides_zoogleoformans_L16488
Bacteroides_uniformis_AB050110
Bacteroides_fluxus_AB490802
Bacteroides_rodentium_AB531489
Bacteroides_graminisolvens_AB363973
Bacteroides_fragilis_CR626927
Bacteroides_nordii_AY608697
Bacteroides_salyersiae_AY608696
Bacteroides_reticulotermitis_AB692943
Bacteroides_pyogenes_AB200229
Bacteroides_faecichinchillae_AB574480
Bacteroides_acidifaciens_AB021164
Bacteroides_caccae_X83951
Bacteroides_faecis_GQ496624
Bacteroides_thetaiotaomicron_AE015928
Bacteroides_finegoldii_AB222699
Bacteroides_ovatus_AB050108
Bacteroides_xylanisolvens_AM230650
Phocaeicola_abscessus_EU694176
Paraprevotella_clara_AB331896
Paraprevotella_xylaniphila_AB331897
Alloprevotella_rava_JQ039189
Alloprevotella_tannerae_AJ005634
Prevotella_oryzae_AB588018
Prevotella_ruminicola_L16482
Prevotella_brevis_AJ011682
Prevotella_oralis_AY323522
Prevotella_timonensis_DQ518919
Prevotella_buccalis_L16476
Prevotella_nanceiensis_AY957555
Prevotella_enoeca_AJ005635
Prevotella_pleuritidis_AB278593
Prevotella_stercorea_AB244774
Prevotella_loescheii_AY836508
Prevotella_shahii_AB108825
Prevotella_marshii_AF481227
Prevotella_saccharolytica_FJ825149
Prevotella_paludivivens_AB078827
Prevotella_maculosa_EF534314
Prevotella_oris_L16474
Prevotella_salivae_AB108826
Prevotella_copri_AB064923
Prevotella_bryantii_AJ006457
Prevotella_oulorum_L16472
Prevotella_albensis_AJ011683
Prevotella_baroniae_AY840553
Prevotella_buccae_L16477
Prevotella_multisaccharivorax_AB200414
Prevotella_bergensis_AY350613
Hallella_seregens_X81877
Prevotella_dentalis_X81876
Prevotella_micans_AF481228
Prevotella_amnii_AM422125
Prevotella_bivia_L16475
Prevotella_corporis_L16465
Prevotella_disiens_L16483
Prevotella_pallens_Y13105
Prevotella_nigrescens_X73963
Prevotella_aurantiaca_AB480534
Prevotella_falsenii_AB429504
Prevotella_intermedia_AB547686
Prevotella_veroralis_L16473

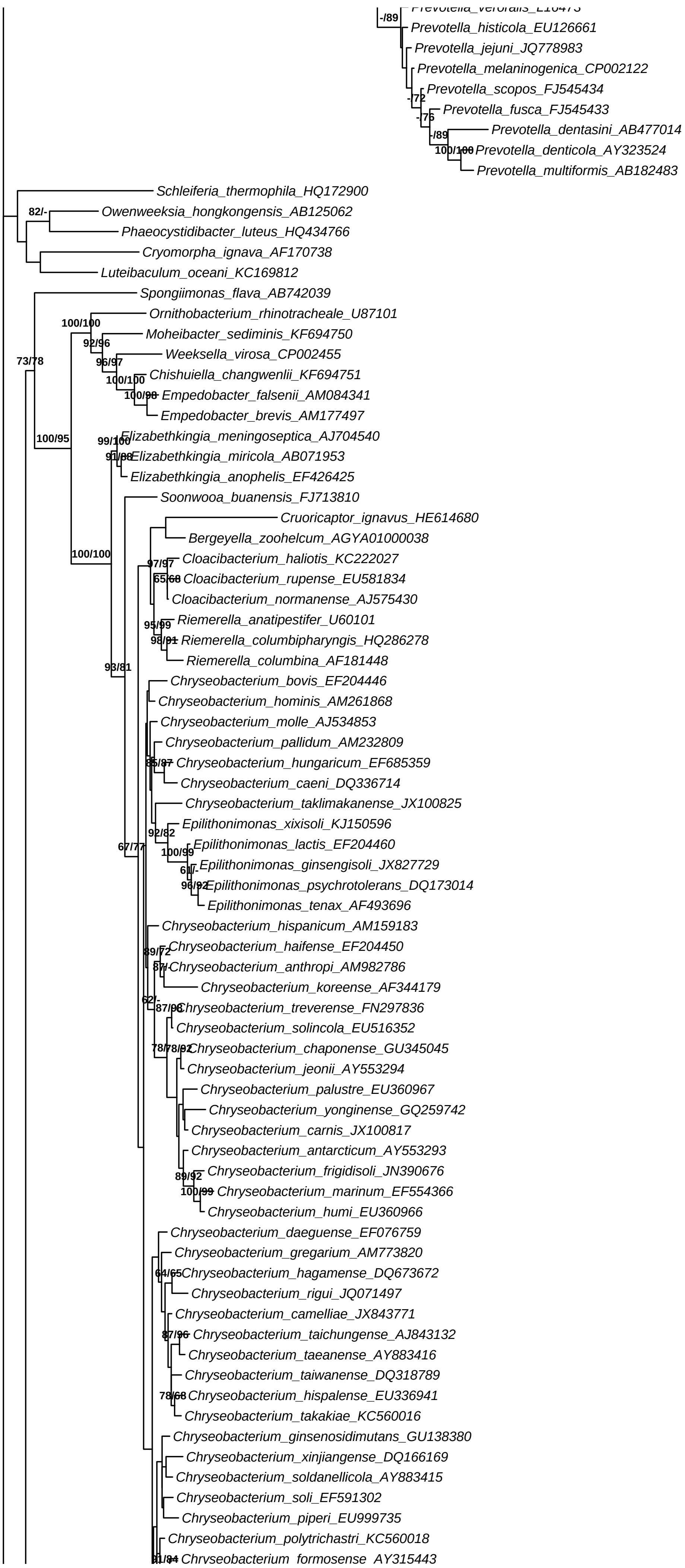

Prevotella_histicola_EU126661
Prevotella_jejuni_JQ778983
Prevotella_melaninogenica_CP002122
Prevotella_scopos_FJ545434
Prevotella_fusca_FJ545433
Prevotella_dentasini_AB477014
Prevotella_denticola_AY323524
Prevotella_multiformis_AB182483
Schleiferia_thermophila_HQ172900
Owenweeksia_hongkongensis_AB125062
Phaeocystidibacter_luteus_HQ434766
Cryomorpha_ignava_AF170738
Luteibaculum_oceani_KC169812
Spongiimonas_flava_AB742039
Ornithobacterium_rhinotracheale_U87101
Moheibacter_sediminis_KF694750
Weeksella_virosa_CP002455
Chishuiella_changwenlii_KF694751
Empedobacter_falsenii_AM084341
Empedobacter_brevis_AM177497
Elizabethkingia_meningoseptica_AJ704540
Elizabethkingia_miricola_AB071953
Elizabethkingia_anophelis_EF426425
Soonwooa_buanensis_FJ713810
Cruoricaptor_ignavus_HE614680
Bergeyella_zoohelcum_AGYA01000038
Cloacibacterium_haliotis_KC222027
Cloacibacterium_rupense_EU581834
Cloacibacterium_normanense_AJ575430
Riemerella_anatipestifer_U60101
Riemerella_columbipharyngis_HQ286278
Riemerella_columbina_AF181448
Chryseobacterium_bovis_EF204446
Chryseobacterium_hominis_AM261868
Chryseobacterium_molle_AJ534853
Chryseobacterium_pallidum_AM232809
Chryseobacterium_hungaricum_EF685359
Chryseobacterium_caeni_DQ336714
Chryseobacterium_taklimakanense_JX100825
Epilithonimonas_xixisoli_KJ150596
Epilithonimonas_lactis_EF204460
Epilithonimonas_ginsengisoli_JX827729
Epilithonimonas_psychrotolerans_DQ173014
Epilithonimonas_tenax_AF493696
Chryseobacterium_hispanicum_AM159183
Chryseobacterium_haifense_EF204450
Chryseobacterium_anthropi_AM982786
Chryseobacterium_koreense_AF344179
Chryseobacterium_treverense_FN297836
Chryseobacterium_solincola_EU516352
Chryseobacterium_chaponense_GU345045
Chryseobacterium_jeonii_AY553294
Chryseobacterium_palustre_EU360967
Chryseobacterium_yonginense_GQ259742
Chryseobacterium_carnis_JX100817
Chryseobacterium_antarcticum_AY553293
Chryseobacterium_frigidisoli_JN390676
Chryseobacterium_marinum_EF554366
Chryseobacterium_humi_EU360966
Chryseobacterium_daeguense_EF076759
Chryseobacterium_gregarium_AM773820
Chryseobacterium_hagamense_DQ673672
Chryseobacterium_rigui_JQ071497
Chryseobacterium_camelliae_JX843771
Chryseobacterium_taichungense_AJ843132
Chryseobacterium_taeanense_AY883416
Chryseobacterium_taiwanense_DQ318789
Chryseobacterium_hispalense_EU336941
Chryseobacterium_takakiae_KC560016
Chryseobacterium_ginsenosidimutans_GU138380
Chryseobacterium_xinjiangense_DQ166169
Chryseobacterium_soldanellicola_AY883415
Chryseobacterium_soli_EF591302
Chryseobacterium_piperi_EU999735
Chryseobacterium_polytrichastri_KC560018
Chryseobacterium_formosense_AY315443

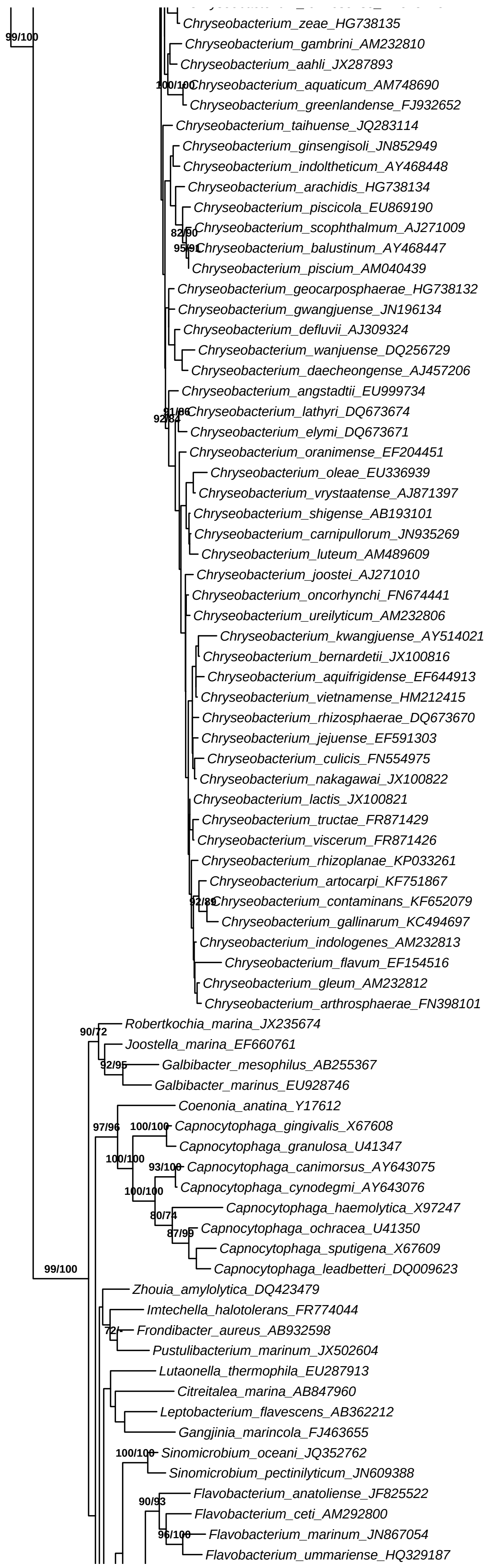

99/100
Chryseobacterium_zeae_HG738135
Chryseobacterium_gambrini_AM232810
Chryseobacterium_aahli_JX287893
100/100
Chryseobacterium_aquaticum_AM748690
Chryseobacterium_greenlandense_FJ932652
Chryseobacterium_taihuense_JQ283114
Chryseobacterium_ginsengisoli_JN852949
Chryseobacterium_indoltheticum_AY468448
Chryseobacterium_arachidis_HG738134
Chryseobacterium_piscicola_EU869190
82/90
Chryseobacterium_scophthalmum_AJ271009
95/91
Chryseobacterium_balustinum_AY468447
Chryseobacterium_piscium_AM040439
Chryseobacterium_geocarposphaerae_HG738132
Chryseobacterium_gwangjuense_JN196134
Chryseobacterium_defluvii_AJ309324
Chryseobacterium_wanjuense_DQ256729
Chryseobacterium_daecheongense_AJ457206
Chryseobacterium_angstadtii_EU999734
91/86
92/84
Chryseobacterium_lathyri_DQ673674
Chryseobacterium_elymi_DQ673671
Chryseobacterium_oranimense_EF204451
Chryseobacterium_oleae_EU336939
Chryseobacterium_vrystaatense_AJ871397
Chryseobacterium_shigense_AB193101
Chryseobacterium_carnipullorum_JN935269
Chryseobacterium_luteum_AM489609
Chryseobacterium_joostei_AJ271010
Chryseobacterium_oncorhynchi_FN674441
Chryseobacterium_ureilyticum_AM232806
Chryseobacterium_kwangjuense_AY514021
Chryseobacterium_bernardetii_JX100816
Chryseobacterium_aquifrigidense_EF644913
Chryseobacterium_vietnamense_HM212415
Chryseobacterium_rhizosphaerae_DQ673670
Chryseobacterium_jejuense_EF591303
Chryseobacterium_culicis_FN554975
Chryseobacterium_nakagawai_JX100822
Chryseobacterium_lactis_JX100821
Chryseobacterium_tructae_FR871429
Chryseobacterium_viscerum_FR871426
Chryseobacterium_rhizoplanae_KP033261
Chryseobacterium_artocarpi_KF751867
92/89
Chryseobacterium_contaminans_KF652079
Chryseobacterium_gallinarum_KC494697
Chryseobacterium_indologenes_AM232813
Chryseobacterium_flavum_EF154516
Chryseobacterium_gleum_AM232812
Chryseobacterium_arthrosphaerae_FN398101
Robertkochia_marina_JX235674
90/72
Joostella_marina_EF660761
92/95
Galbibacter_mesophilus_AB255367
Galbibacter_marinus_EU928746
Coenonia_anatina_Y17612
97/96
100/100
Capnocytophaga_gingivalis_X67608
Capnocytophaga_granulosa_U41347
100/100
93/100
Capnocytophaga_canimorsus_AY643075
Capnocytophaga_cynodegmi_AY643076
100/100
Capnocytophaga_haemolytica_X97247
80/74
87/95
Capnocytophaga_ochracea_U41350
Capnocytophaga_sputigena_X67609
Capnocytophaga_leadbetteri_DQ009623
99/100
Zhouia_amylolytica_DQ423479
Imtechella_halotolerans_FR774044
72/-
Frondibacter_aureus_AB932598
Pustulibacterium_marinum_JX502604
Lutaonella_thermophila_EU287913
Citreitalea_marina_AB847960
Leptobacterium_flavescens_AB362212
Gangjinia_marincola_FJ463655
100/100
Sinomicrobium_oceani_JQ352762
Sinomicrobium_pectinilyticum_JN609388
Flavobacterium_anatoliense_JF825522
90/93
Flavobacterium_ceti_AM292800
96/100
Flavobacterium_marinum_JN867054
Flavobacterium_ummariense_HQ329187

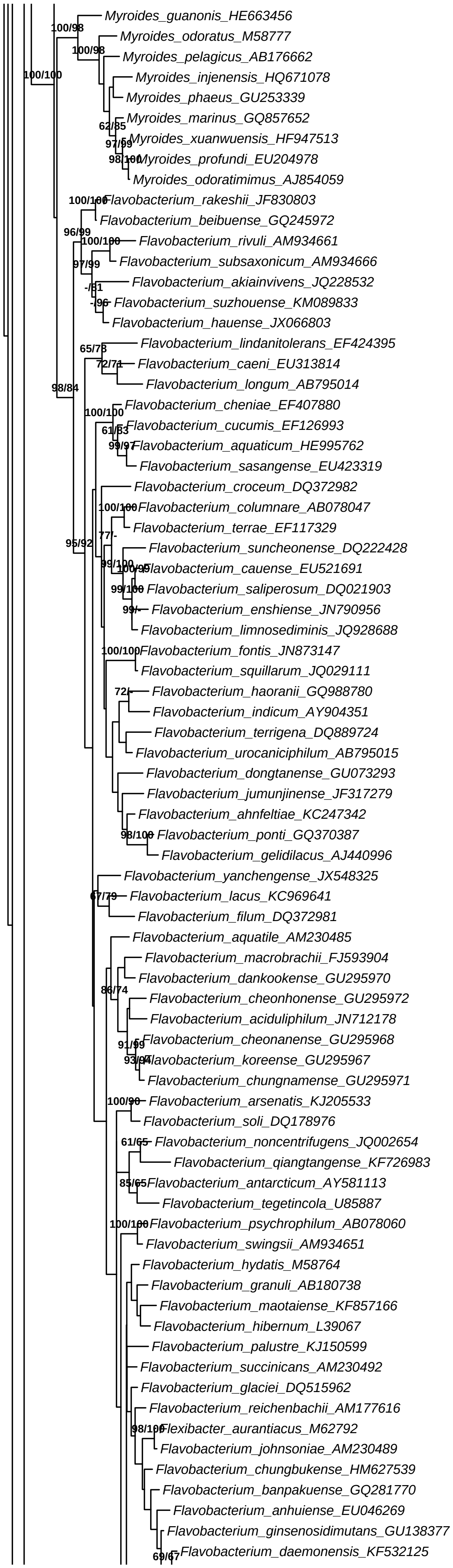

Myroides_guanonis_HE663456
100/98
Myroides_odoratus_M58777
100/98
Myroides_pelagicus_AB176662
100/100
Myroides_injenensis_HQ671078
Myroides_phaeus_GU253339
Myroides_marinus_GQ857652
62/85
Myroides_xuanwuensis_HF947513
97/99
98/100
Myroides_profundi_EU204978
Myroides_odoratimimus_AJ854059
100/100
Flavobacterium_rakeshii_JF830803
Flavobacterium_beibuense_GQ245972
96/99
100/100
Flavobacterium_rivuli_AM934661
97/99
Flavobacterium_subsaxonicum_AM934666
Flavobacterium_akiainvivens_JQ228532
-/81
-/96
Flavobacterium_suzhouense_KM089833
Flavobacterium_hauense_JX066803
65/78
Flavobacterium_lindanitolerans_EF424395
72/71
Flavobacterium_caeni_EU313814
98/84
Flavobacterium_longum_AB795014
Flavobacterium_cheniae_EF407880
100/100
Flavobacterium_cucumis_EF126993
61/83
99/97
Flavobacterium_aquaticum_HE995762
Flavobacterium_sasangense_EU423319
Flavobacterium_croceum_DQ372982
100/100
Flavobacterium_columnare_AB078047
Flavobacterium_terrae_EF117329
77/-
95/92
Flavobacterium_suncheonense_DQ222428
99/100
100/95
Flavobacterium_cauense_EU521691
99/100
Flavobacterium_saliperosum_DQ021903
99/-
Flavobacterium_enshiense_JN790956
Flavobacterium_limnosediminis_JQ928688
100/100
Flavobacterium_fontis_JN873147
Flavobacterium_squillarum_JQ029111
72/-
Flavobacterium_haoranii_GQ988780
Flavobacterium_indicum_AY904351
Flavobacterium_terrigena_DQ889724
Flavobacterium_urocaniciphilum_AB795015
Flavobacterium_dongtanense_GU073293
Flavobacterium_jumunjinense_JF317279
Flavobacterium_ahnfeltiae_KC247342
98/100
Flavobacterium_ponti_GQ370387
Flavobacterium_gelidilacus_AJ440996
Flavobacterium_yanchengense_JX548325
67/79
Flavobacterium_lacus_KC969641
Flavobacterium_filum_DQ372981
Flavobacterium_aquatile_AM230485
Flavobacterium_macrobrachii_FJ593904
Flavobacterium_dankookense_GU295970
86/74
Flavobacterium_cheonhonense_GU295972
Flavobacterium_aciduliphilum_JN712178
91/99
Flavobacterium_cheonanense_GU295968
93/94
Flavobacterium_koreense_GU295967
Flavobacterium_chungnamense_GU295971
100/90
Flavobacterium_arsenatis_KJ205533
Flavobacterium_soli_DQ178976
61/65
Flavobacterium_noncentrifugens_JQ002654
Flavobacterium_qiangtangense_KF726983
85/65
Flavobacterium_antarcticum_AY581113
Flavobacterium_tegetincola_U85887
100/100
Flavobacterium_psychrophilum_AB078060
Flavobacterium_swingsii_AM934651
Flavobacterium_hydatis_M58764
Flavobacterium_granuli_AB180738
Flavobacterium_maotaiense_KF857166
Flavobacterium_hibernum_L39067
Flavobacterium_palustre_KJ150599
Flavobacterium_succinicans_AM230492
Flavobacterium_glaciei_DQ515962
Flavobacterium_reichenbachii_AM177616
98/100
Flexibacter_aurantiacus_M62792
Flavobacterium_johnsoniae_AM230489
Flavobacterium_chungbukense_HM627539
Flavobacterium_banpakuense_GQ281770
Flavobacterium_anhuiense_EU046269
Flavobacterium_ginsenosidimutans_GU138377
69/67
Flavobacterium_daemonensis_KF532125

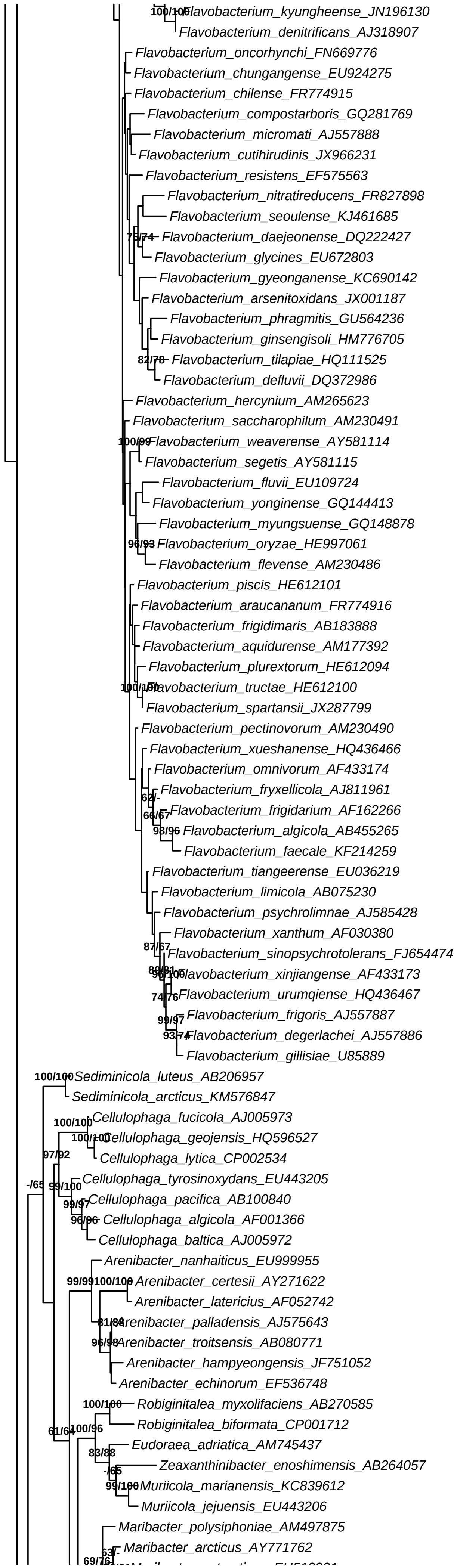
Flavobacterium_kyungheense_JN196130
Flavobacterium_denitrificans_AJ318907
Flavobacterium_oncorhynchi_FN669776
Flavobacterium_chungangense_EU924275
Flavobacterium_chilense_FR774915
Flavobacterium_compostarboris_GQ281769
Flavobacterium_micromati_AJ557888
Flavobacterium_cutihirudinis_JX966231
Flavobacterium_resistens_EF575563
Flavobacterium_nitratireducens_FR827898
Flavobacterium_seoulense_KJ461685
Flavobacterium_daejeonense_DQ222427
Flavobacterium_glycines_EU672803
Flavobacterium_gyeonganense_KC690142
Flavobacterium_arsenitoxidans_JX001187
Flavobacterium_phragmitis_GU564236
Flavobacterium_ginsengisoli_HM776705
82/78
Flavobacterium_tilapiae_HQ111525
Flavobacterium_defluvii_DQ372986
Flavobacterium_hercynium_AM265623
Flavobacterium_saccharophilum_AM230491
100/99
Flavobacterium_weaverense_AY581114
Flavobacterium_segetis_AY581115
Flavobacterium_fluvii_EU109724
Flavobacterium_yonginense_GQ144413
Flavobacterium_myungsuense_GQ148878
96/93
Flavobacterium_oryzae_HE997061
Flavobacterium_flevense_AM230486
Flavobacterium_piscis_HE612101
Flavobacterium_araucananum_FR774916
Flavobacterium_frigidimaris_AB183888
Flavobacterium_aquidurense_AM177392
Flavobacterium_plurextorum_HE612094
Flavobacterium_tructae_HE612100
Flavobacterium_spartansii_JX287799
Flavobacterium_pectinovorum_AM230490
Flavobacterium_xueshanense_HQ436466
Flavobacterium_omnivorum_AF433174
62/-
Flavobacterium_fryxellicola_AJ811961
66/67
Flavobacterium_frigidarium_AF162266
Flavobacterium_algicola_AB455265
Flavobacterium_faecale_KF214259
Flavobacterium_tiangeerense_EU036219
Flavobacterium_limicola_AB075230
Flavobacterium_psychrolimnae_AJ585428
Flavobacterium_xanthum_AF030380
87/67
Flavobacterium_sinopsychrotolerans_FJ654474
Flavobacterium_xinjiangense_AF433173
74/76
Flavobacterium_urumqiense_HQ436467
99/97
Flavobacterium_frigoris_AJ557887
Flavobacterium_degerlachei_AJ557886
Flavobacterium_gillisiae_U85889
100/100
Sediminicola_luteus_AB206957
Sediminicola_arcticus_KM576847
100/100
Cellulophaga_fucicola_AJ005973
Cellulophaga_geojensis_HQ596527
97/92
Cellulophaga_lytica_CP002534
-/65
99/100
Cellulophaga_tyrosinoxydans_EU443205
99/97
Cellulophaga_pacifica_AB100840
Cellulophaga_algicola_AF001366
Cellulophaga_baltica_AJ005972
Arenibacter_nanhaiticus_EU999955
99/99
Arenibacter_certesii_AY271622
Arenibacter_latericius_AF052742
Arenibacter_palladensis_AJ575643
96/98
Arenibacter_troitsensis_AB080771
Arenibacter_hampyeongensis_JF751052
Arenibacter_echinorum_EF536748
Robiginitalea_myxolifaciens_AB270585
Robiginitalea_biformata_CP001712
61/64
100/96
Eudoraea_adriatica_AM745437
83/88
Zeaxanthinibacter_enoshimensis_AB264057
Muriicola_marianensis_KC839612
Muriicola_jejuensis_EU443206
Maribacter_polysiphoniae_AM497875
Maribacter_arcticus_AY771762
69/76

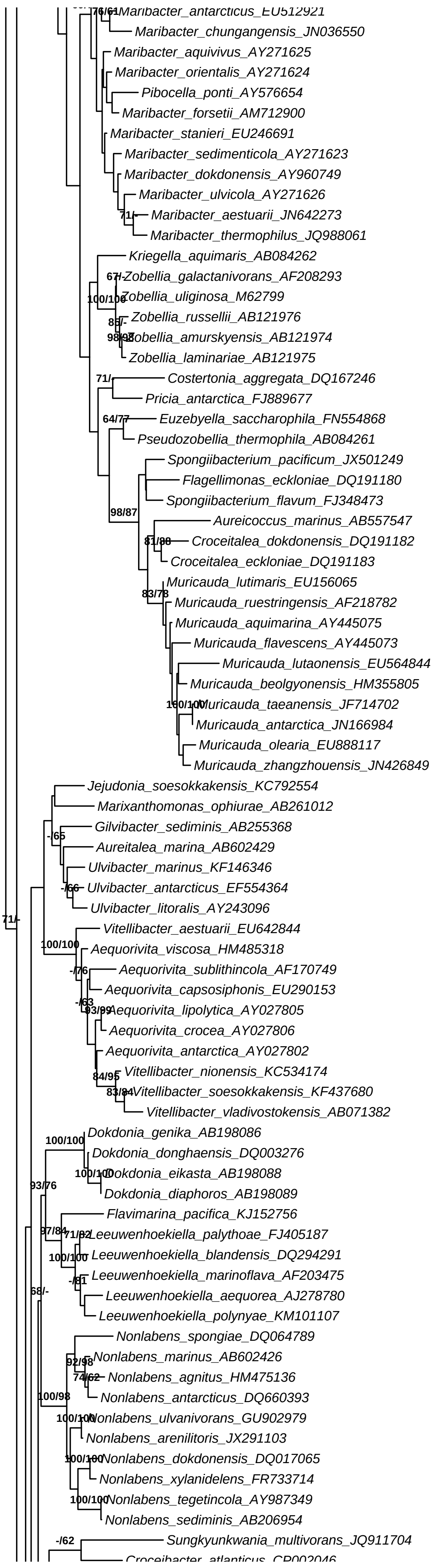

76/61 Maribacter_antarcticus_EU512921
Maribacter_chungangensis_JN036550
Maribacter_aquivivus_AY271625
Maribacter_orientalis_AY271624
Pibocella_ponti_AY576654
Maribacter_forsetii_AM712900
Maribacter_stanieri_EU246691
Maribacter_sedimenticola_AY271623
Maribacter_dokdonensis_AY960749
Maribacter_ulvicola_AY271626
71/- Maribacter_aestuarii_JN642273
Maribacter_thermophilus_JQ988061
Kriegella_aquimaris_AB084262
67/- Zobellia_galactanivorans_AF208293
100/100 Zobellia_uliginosa_M62799
Zobellia_russellii_AB121976
Zobellia_amurskyensis_AB121974
Zobellia_laminariae_AB121975
71/- Costertonia_aggregata_DQ167246
Pricia_antarctica_FJ889677
64/77 Euzebyella_saccharophila_FN554868
Pseudozobellia_thermophila_AB084261
Spongiibacterium_pacificum_JX501249
Flagellimonas_eckloniae_DQ191180
Spongiibacterium_flavum_FJ348473
98/87
Aureicoccus_marinus_AB557547
81/88 Croceitalea_dokdonensis_DQ191182
Croceitalea_eckloniae_DQ191183
Muricauda_lutimaris_EU156065
83/78 Muricauda_ruestringensis_AF218782
Muricauda_aquimarina_AY445075
Muricauda_flavescens_AY445073
Muricauda_lutaonensis_EU564844
Muricauda_beolgyonensis_HM355805
100/100 Muricauda_taeanensis_JF714702
Muricauda_antarctica_JN166984
Muricauda_olearia_EU888117
Muricauda_zhangzhouensis_JN426849
Jejudonia_soesokkakensis_KC792554
Marixanthomonas_ophiurae_AB261012
Gilvibacter_sediminis_AB255368
-/65
Aureitalea_marina_AB602429
Ulvibacter_marinus_KF146346
-/66 Ulvibacter_antarcticus_EF554364
Ulvibacter_litoralis_AY243096
71/-
Vitellibacter_aestuarii_EU642844
100/100 Aequorivita_viscosa_HM485318
-/76 Aequorivita_sublithincola_AF170749
Aequorivita_capsosiphonis_EU290153
-/63
93/99 Aequorivita_lipolytica_AY027805
Aequorivita_crocea_AY027806
Aequorivita_antarctica_AY027802
Vitellibacter_nionensis_KC534174
84/95
83/94 Vitellibacter_soesokkakensis_KF437680
Vitellibacter_vladivostokensis_AB071382
100/100 Dokdonia_genika_AB198086
Dokdonia_donghaensis_DQ003276
100/100 Dokdonia_eikasta_AB198088
93/76 Dokdonia_diaphoros_AB198089
Flavimarina_pacifica_KJ152756
97/84 71/82 Leeuwenhoekiella_palythoae_FJ405187
100/100 Leeuwenhoekiella_blandensis_DQ294291
Leeuwenhoekiella_marinoflava_AF203475
-/81
68/- Leeuwenhoekiella_aequorea_AJ278780
Leeuwenhoekiella_polynyae_KM101107
Nonlabens_spongiae_DQ064789
Nonlabens_marinus_AB602426
92/98
74/62 Nonlabens_agnitus_HM475136
Nonlabens_antarcticus_DQ660393
100/98
100/100 Nonlabens_ulvanivorans_GU902979
Nonlabens_arenilitoris_JX291103
100/100 Nonlabens_dokdonensis_DQ017065
Nonlabens_xylanidelens_FR733714
100/100 Nonlabens_tegetincola_AY987349
Nonlabens_sediminis_AB206954
-/62 Sungkyunkwania_multivorans_JQ911704

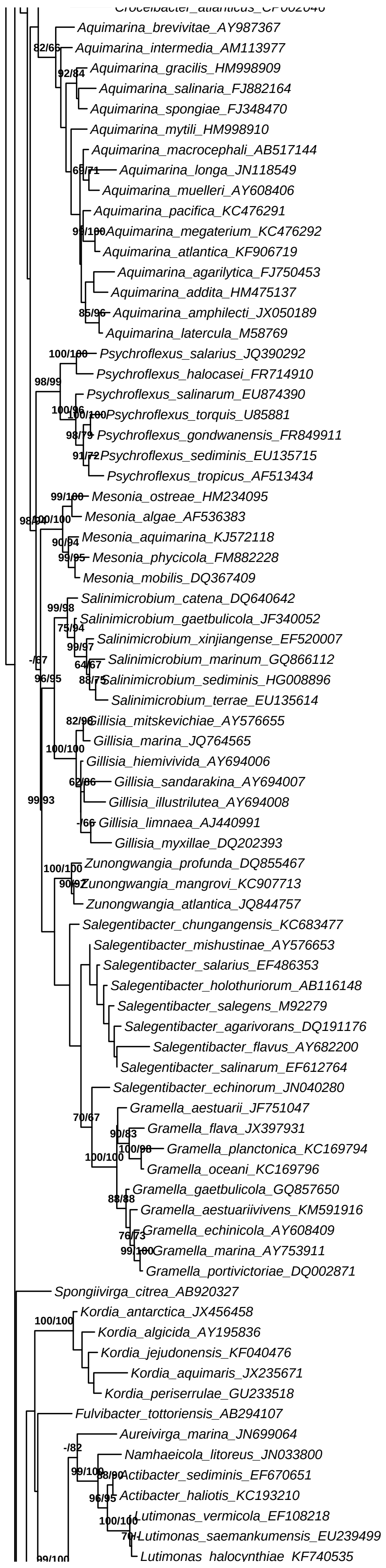

Aquimarina_brevivitae_AY987367
82/66
Aquimarina_intermedia_AM113977
Aquimarina_gracilis_HM998909
92/84
Aquimarina_salinaria_FJ882164
Aquimarina_spongiae_FJ348470
Aquimarina_mytili_HM998910
Aquimarina_macrocephali_AB517144
Aquimarina_longa_JN118549
Aquimarina_muelleri_AY608406
Aquimarina_pacifica_KC476291
Aquimarina_megaterium_KC476292
Aquimarina_atlantica_KF906719
Aquimarina_agarilytica_FJ750453
Aquimarina_addita_HM475137
85/96
Aquimarina_amphilecti_JX050189
Aquimarina_latercula_M58769
Psychroflexus_salarius_JQ390292
Psychroflexus_halocasei_FR714910
98/99
Psychroflexus_salinarum_EU874390
100/96
Psychroflexus_torquis_U85881
98/79
Psychroflexus_gondwanensis_FR849911
Psychroflexus_sediminis_EU135715
Psychroflexus_tropicus_AF513434
Mesonia_ostreae_HM234095
Mesonia_algae_AF536383
Mesonia_aquimarina_KJ572118
90/94
Mesonia_phycicola_FM882228
99/95
Mesonia_mobilis_DQ367409
Salinimicrobium_catena_DQ640642
99/98
Salinimicrobium_gaetbulicola_JF340052
75/94
Salinimicrobium_xinjiangense_EF520007
99/97
Salinimicrobium_marinum_GQ866112
64/67
-/67
96/95
Salinimicrobium_sediminis_HG008896
Salinimicrobium_terrae_EU135614
Gillisia_mitskevichiae_AY576655
Gillisia_marina_JQ764565
100/100
Gillisia_hiemivivida_AY694006
62/86
Gillisia_sandarakina_AY694007
Gillisia_illustrilutea_AY694008
99/93
Gillisia_limnaea_AJ440991
Gillisia_myxillae_DQ202393
Zunongwangia_profunda_DQ855467
100/100
Zunongwangia_mangrovi_KC907713
Zunongwangia_atlantica_JQ844757
Salegentibacter_chungangensis_KC683477
Salegentibacter_mishustinae_AY576653
Salegentibacter_salarius_EF486353
Salegentibacter_holothuriorum_AB116148
Salegentibacter_salegens_M92279
Salegentibacter_agarivorans_DQ191176
Salegentibacter_flavus_AY682200
Salegentibacter_salinarum_EF612764
Salegentibacter_echinorum_JN040280
Gramella_aestuarii_JF751047
70/67
Gramella_flava_JX397931
90/83
Gramella_planctonica_KC169794
100/98
100/100
Gramella_oceani_KC169796
Gramella_gaetbulicola_GQ857650
88/88
Gramella_aestuariivivens_KM591916
Gramella_echinicola_AY608409
76/73
Gramella_marina_AY753911
Gramella_portivictoriae_DQ002871
Spongiivirga_citrea_AB920327
Kordia_antarctica_JX456458
100/100
Kordia_algicida_AY195836
Kordia_jejudonensis_KF040476
Kordia_aquimaris_JX235671
Kordia_periserrulae_GU233518
Fulvibacter_tottoriensis_AB294107
Aureivirga_marina_JN699064
-/82
Namhaeicola_litoreus_JN033800
Actibacter_sediminis_EF670651
Actibacter_haliotis_KC193210
96/95
Lutimonas_vermicola_EF108218
100/100
Lutimonas_saemankumensis_EU239499
Lutimonas_halocynthiae_KF740535
99/100

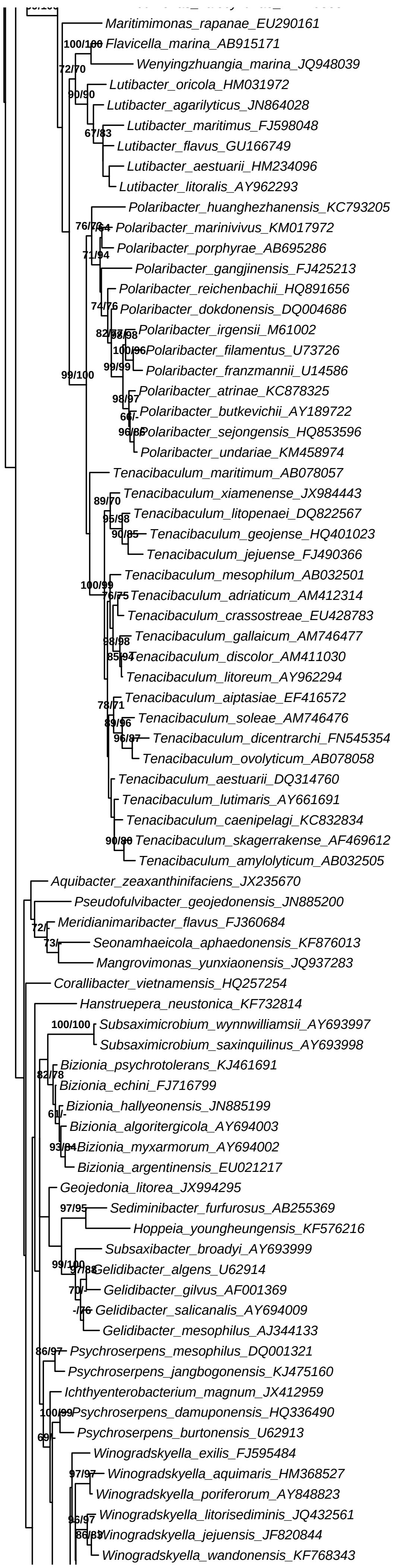

Maritimimonas_rapanae_EU290161
Flavicella_marina_AB915171
Wenyingzhuangia_marina_JQ948039
Lutibacter_oricola_HM031972
Lutibacter_agarilyticus_JN864028
Lutibacter_maritimus_FJ598048
Lutibacter_flavus_GU166749
Lutibacter_aestuarii_HM234096
Lutibacter_litoralis_AY962293
Polaribacter_huanghezhanensis_KC793205
Polaribacter_marinivivus_KM017972
Polaribacter_porphyrae_AB695286
Polaribacter_gangjinensis_FJ425213
Polaribacter_reichenbachii_HQ891656
Polaribacter_dokdonensis_DQ004686
Polaribacter_irgensii_M61002
Polaribacter_filamentus_U73726
Polaribacter_franzmannii_U14586
Polaribacter_atrinae_KC878325
Polaribacter_butkevichii_AY189722
Polaribacter_sejongensis_HQ853596
Polaribacter_undariae_KM458974
Tenacibaculum_maritimum_AB078057
Tenacibaculum_xiamenense_JX984443
Tenacibaculum_litopenaei_DQ822567
Tenacibaculum_geojense_HQ401023
Tenacibaculum_jejuense_FJ490366
Tenacibaculum_mesophilum_AB032501
Tenacibaculum_adriaticum_AM412314
Tenacibaculum_crassostreae_EU428783
Tenacibaculum_gallaicum_AM746477
Tenacibaculum_discolor_AM411030
Tenacibaculum_litoreum_AY962294
Tenacibaculum_aiptasiae_EF416572
Tenacibaculum_soleae_AM746476
Tenacibaculum_dicentrarchi_FN545354
Tenacibaculum_ovolyticum_AB078058
Tenacibaculum_aestuarii_DQ314760
Tenacibaculum_lutimaris_AY661691
Tenacibaculum_caenipelagi_KC832834
Tenacibaculum_skagerrakense_AF469612
Tenacibaculum_amylolyticum_AB032505
Aquibacter_zeaxanthinifaciens_JX235670
Pseudofulvibacter_geojedonensis_JN885200
Meridianimaribacter_flavus_FJ360684
Seonamhaeicola_aphaedonensis_KF876013
Mangrovimonas_yunxiaonensis_JQ937283
Corallibacter_vietnamensis_HQ257254
Hanstruepera_neustonica_KF732814
Subsaximicrobium_wynnwilliamsii_AY693997
Subsaximicrobium_saxinquilinus_AY693998
Bizionia_psychrotolerans_KJ461691
Bizionia_echini_FJ716799
Bizionia_hallyeonensis_JN885199
Bizionia_algoritergicola_AY694003
Bizionia_myxarmorum_AY694002
Bizionia_argentinensis_EU021217
Geojedonia_litorea_JX994295
Sediminibacter_furfurosus_AB255369
Hoppeia_youngheungensis_KF576216
Subsaxibacter_broadyi_AY693999
Gelidibacter_algens_U62914
Gelidibacter_gilvus_AF001369
Gelidibacter_salicanalis_AY694009
Gelidibacter_mesophilus_AJ344133
Psychroserpens_mesophilus_DQ001321
Psychroserpens_jangbogonensis_KJ475160
Ichthyenterobacterium_magnum_JX412959
Psychroserpens_damuponensis_HQ336490
Psychroserpens_burtonensis_U62913
Winogradskyella_exilis_FJ595484
Winogradskyella_aquimaris_HM368527
Winogradskyella_poriferorum_AY848823
Winogradskyella_litorisediminis_JQ432561
Winogradskyella_jejuensis_JF820844
Winogradskyella_wandonensis_KF768343

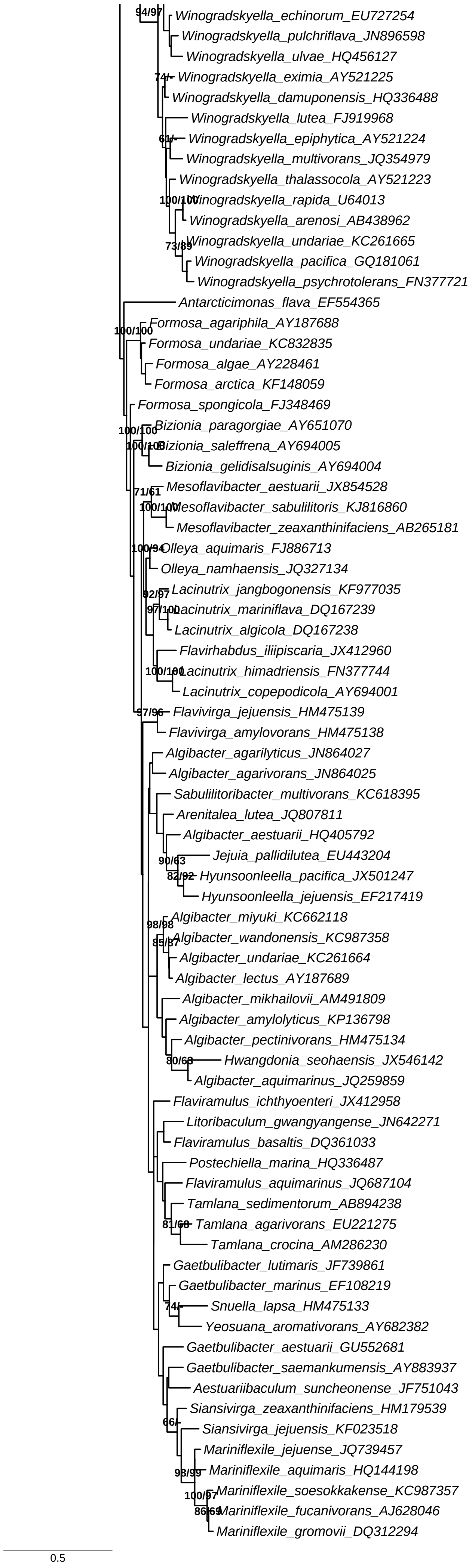
94/97
Winogradskyella_echinorum_EU727254
Winogradskyella_pulchriflava_JN896598
Winogradskyella_ulvae_HQ456127
74/-
Winogradskyella_eximia_AY521225
Winogradskyella_damuponensis_HQ336488
Winogradskyella_lutea_FJ919968
61/-
Winogradskyella_epiphytica_AY521224
Winogradskyella_multivorans_JQ354979
Winogradskyella_thalassocola_AY521223
100/100
Winogradskyella_rapida_U64013
Winogradskyella_arenosi_AB438962
73/89
Winogradskyella_undariae_KC261665
Winogradskyella_pacifica_GQ181061
Winogradskyella_psychrotolerans_FN377721
Antarcticimonas_flava_EF554365
100/100
Formosa_agariphila_AY187688
Formosa_undariae_KC832835
Formosa_algae_AY228461
Formosa_arctica_KF148059
Formosa_spongicola_FJ348469
100/100
Bizionia_paragorgiae_AY651070
100/100
Bizionia_saleffrena_AY694005
Bizionia_gelidisalsuginis_AY694004
71/61
Mesoflavibacter_aestuarii_JX854528
100/100
Mesoflavibacter_sabulilitoris_KJ816860
Mesoflavibacter_zeaxanthinifaciens_AB265181
100/94
Olleya_aquimaris_FJ886713
Olleya_namhaensis_JQ327134
92/97
Lacinutrix_jangbogonensis_KF977035
97/100
Lacinutrix_mariniflava_DQ167239
Lacinutrix_algicola_DQ167238
Flavirhabdus_iliipiscaria_JX412960
100/100
Lacinutrix_himadriensis_FN377744
Lacinutrix_copepodicola_AY694001
97/96
Flavivirga_jejuensis_HM475139
Flavivirga_amylovorans_HM475138
Algibacter_agarilyticus_JN864027
Algibacter_agarivorans_JN864025
Sabulilitoribacter_multivorans_KC618395
Arenitalea_lutea_JQ807811
Algibacter_aestuarii_HQ405792
90/63
Jejuia_pallidilutea_EU443204
82/92
Hyunsoonleella_pacifica_JX501247
Hyunsoonleella_jejuensis_EF217419
98/98
Algibacter_miyuki_KC662118
85/87
Algibacter_wandonensis_KC987358
Algibacter_undariae_KC261664
Algibacter_lectus_AY187689
Algibacter_mikhailovii_AM491809
Algibacter_amylolyticus_KP136798
Algibacter_pectinivorans_HM475134
80/63
Hwangdonia_seohaensis_JX546142
Algibacter_aquimarinus_JQ259859
Flaviramulus_ichthyoenteri_JX412958
Litoribaculum_gwangyangense_JN642271
Flaviramulus_basaltis_DQ361033
Postechiella_marina_HQ336487
Flaviramulus_aquimarinus_JQ687104
Tamlana_sedimentorum_AB894238
81/68
Tamlana_agarivorans_EU221275
Tamlana_crocina_AM286230
Gaetbulibacter_lutimaris_JF739861
Gaetbulibacter_marinus_EF108219
74/-
Snuella_lapsa_HM475133
Yeosuana_aromativorans_AY682382
Gaetbulibacter_aestuarii_GU552681
Gaetbulibacter_saemankumensis_AY883937
Aestuariibaculum_suncheonense_JF751043
Siansivirga_zeaxanthinifaciens_HM179539
66/-
Siansivirga_jejuensis_KF023518
Mariniflexile_jejuense_JQ739457
98/99
Mariniflexile_aquimaris_HQ144198
100/97
Mariniflexile_soesokkakense_KC987357
86/69
Mariniflexile_fucanivorans_AJ628046
Mariniflexile_gromovii_DQ312294
0.5

Figure 3 - 16S rRNA gene ML and MP tree using the sampling from the GBDP tree

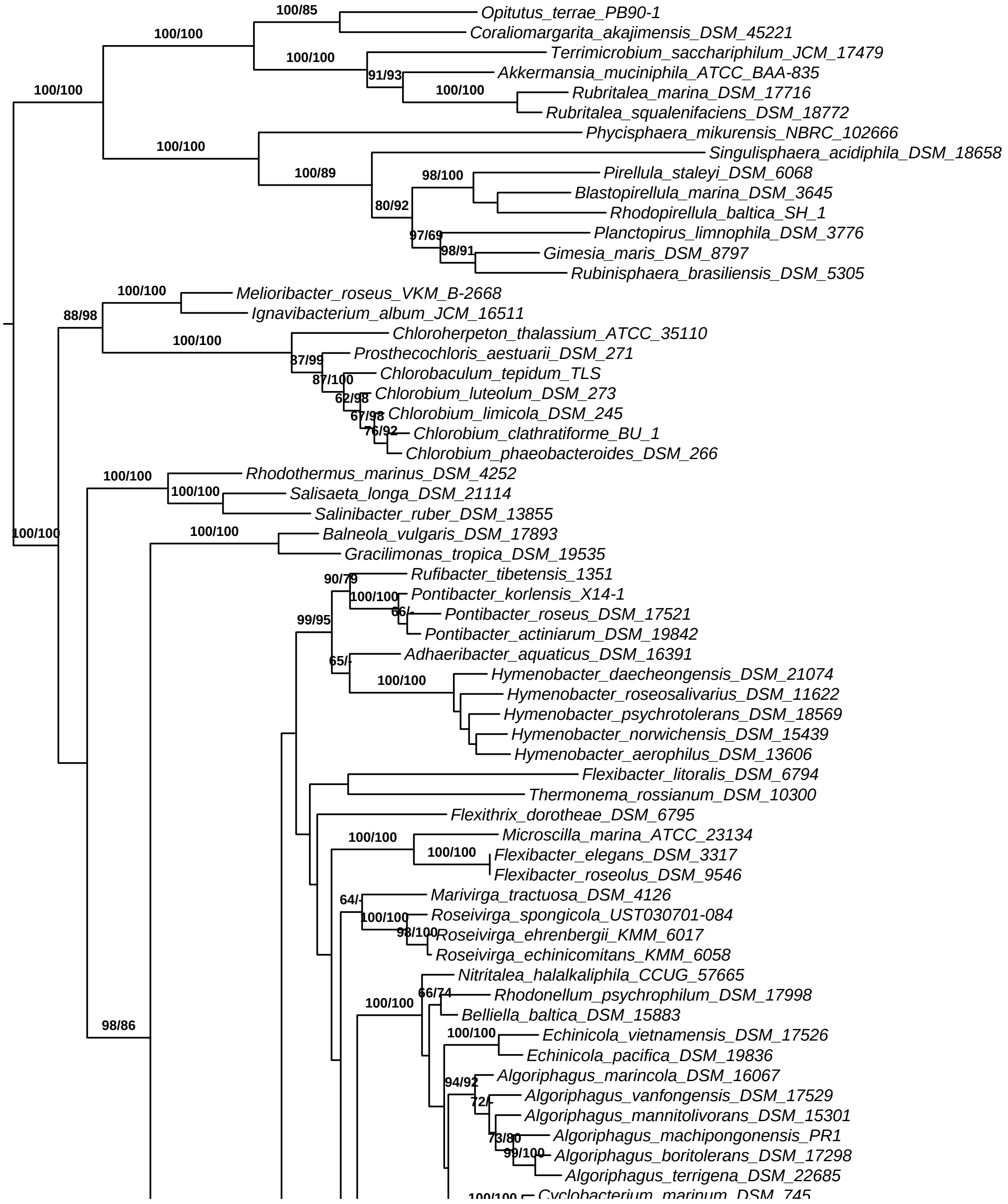
Opitutus_terrae_PB90-1
Coraliomargarita_akajimensis_DSM_45221
Terrimicrobium_sacchariphilum_JCM_17479
Akkermansia_muciniphila_ATCC_BAA-835
Rubritalea_marina_DSM_17716
Rubritalea_squalenifaciens_DSM_18772
Phycisphaera_mikurensis_NBRC_102666
Singulisphaera_acidiphila_DSM_18658
Pirellula_staleyi_DSM_6068
Blastopirellula_marina_DSM_3645
Rhodopirellula_baltica_SH_1
Planctopirus_limnophila_DSM_3776
Gimesia_maris_DSM_8797
Rubinisphaera_brasiliensis_DSM_5305
Melioribacter_roseus_VKM_B-2668
Ignavibacterium_album_JCM_16511
Chloroherpeton_thalassium_ATCC_35110
Prosthecochloris_aestuarii_DSM_271
Chlorobaculum_tepidum_TLS
Chlorobium_luteolum_DSM_273
Chlorobium_limicola_DSM_245
Chlorobium_clathratiforme_BU_1
Chlorobium_phaeobacteroides_DSM_266
Rhodothermus_marinus_DSM_4252
Salisaeta_longa_DSM_21114
Salinibacter_ruber_DSM_13855
Balneola_vulgaris_DSM_17893
Gracilimonas_tropica_DSM_19535
Rufibacter_tibetensis_1351
Pontibacter_korlensis_X14-1
Pontibacter_roseus_DSM_17521
Pontibacter_actiniarum_DSM_19842
Adhaeribacter_aquaticus_DSM_16391
Hymenobacter_daecheongensis_DSM_21074
Hymenobacter_roseosalivarius_DSM_11622
Hymenobacter_psychrotolerans_DSM_18569
Hymenobacter_norwichensis_DSM_15439
Hymenobacter_aerophilus_DSM_13606
Flexibacter_litoralis_DSM_6794
Thermonema_rossianum_DSM_10300
Flexithrix_dorotheae_DSM_6795
Microscilla_marina_ATCC_23134
Flexibacter_elegans_DSM_3317
Flexibacter_roseolus_DSM_9546
Marivirga_tractuosa_DSM_4126
Roseivirga_spongicola_UST030701-084
Roseivirga_ehrenbergii_KMM_6017
Roseivirga_echinicomitans_KMM_6058
Nitritalea_halalkaliphila_CCUG_57665
Rhodonellum_psychrophilum_DSM_17998
Belliella_baltica_DSM_15883
Echinicola_vietnamensis_DSM_17526
Echinicola_pacifica_DSM_19836
Algoriphagus_marincola_DSM_16067
Algoriphagus_vanfongensis_DSM_17529
Algoriphagus_mannitolivorans_DSM_15301
Algoriphagus_machipongonensis_PR1
Algoriphagus_boritolerans_DSM_17298
Algoriphagus_terrigena_DSM_22685

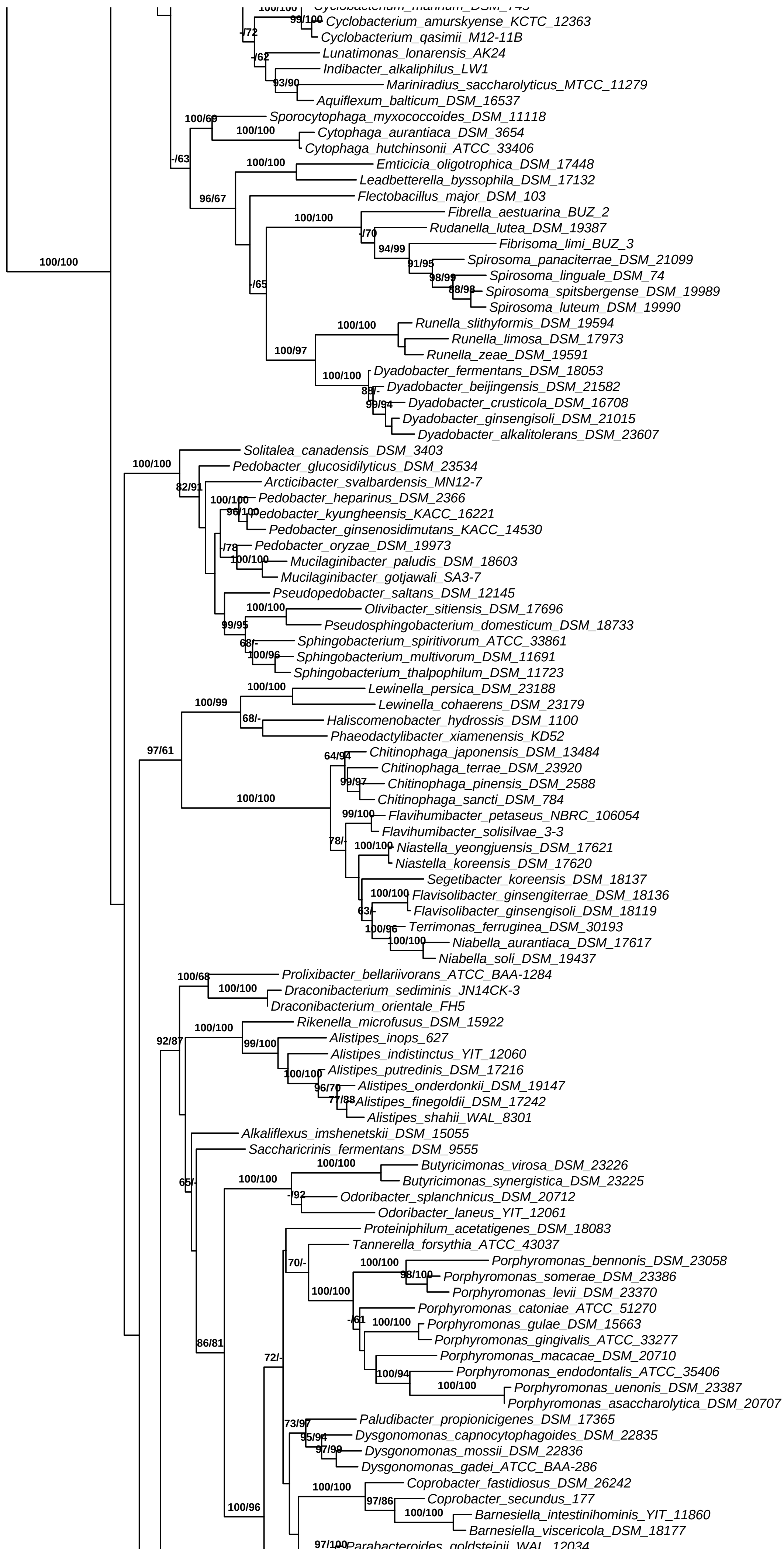

99/100
Cyclobacterium_amurskyense_KCTC_12363
-/72
Cyclobacterium_qasimii_M12-11B
Lunatimonas_lonarensis_AK24
-/62
Indibacter_alkaliphilus_LW1
93/90
Mariniradius_saccharolyticus_MTCC_11279
Aquiflexum_balticum_DSM_16537
100/69
Sporocytophaga_myxococcoides_DSM_11118
100/100
Cytophaga_aurantiaca_DSM_3654
Cytophaga_hutchinsonii_ATCC_33406
-/63
100/100
Emticicia_oligotrophica_DSM_17448
Leadbetterella_byssophila_DSM_17132
96/67
Flectobacillus_major_DSM_103
100/100
Fibrella_aestuarina_BUZ_2
-/70
Rudanella_lutea_DSM_19387
94/99
Fibrisoma_limi_BUZ_3
100/100
91/95
Spirosoma_panaciterrae_DSM_21099
98/99
Spirosoma_linguale_DSM_74
88/98
Spirosoma_spitsbergense_DSM_19989
-/65
Spirosoma_luteum_DSM_19990
100/100
Runella_slithyformis_DSM_19594
Runella_limosa_DSM_17973
100/97
Runella_zeae_DSM_19591
100/100
Dyadobacter_fermentans_DSM_18053
88/-
Dyadobacter_beijingensis_DSM_21582
99/94
Dyadobacter_crusticola_DSM_16708
Dyadobacter_ginsengisoli_DSM_21015
Dyadobacter_alkalitolerans_DSM_23607
Solitalea_canadensis_DSM_3403
100/100
Pedobacter_glucosidilyticus_DSM_23534
82/91
Arcticibacter_svalbardensis_MN12-7
100/100
Pedobacter_heparinus_DSM_2366
96/100
Pedobacter_kyungheensis_KACC_16221
Pedobacter_ginsenosidimutans_KACC_14530
-/78
Pedobacter_oryzae_DSM_19973
100/100
Mucilaginibacter_paludis_DSM_18603
Mucilaginibacter_gotjawali_SA3-7
Pseudopedobacter_saltans_DSM_12145
100/100
Olivibacter_sitiensis_DSM_17696
99/95
Pseudosphingobacterium_domesticum_DSM_18733
68/-
Sphingobacterium_spiritivorum_ATCC_33861
100/96
Sphingobacterium_multivorum_DSM_11691
Sphingobacterium_thalpophilum_DSM_11723
100/100
Lewinella_persica_DSM_23188
100/99
Lewinella_cohaerens_DSM_23179
68/-
Haliscomenobacter_hydrossis_DSM_1100
Phaeodactylibacter_xiamenensis_KD52
97/61
64/94
Chitinophaga_japonensis_DSM_13484
Chitinophaga_terrae_DSM_23920
99/97
Chitinophaga_pinensis_DSM_2588
100/100
Chitinophaga_sancti_DSM_784
99/100
Flavihumibacter_petaseus_NBRC_106054
Flavihumibacter_solisilvae_3-3
78/-
100/100
Niastella_yeongjuensis_DSM_17621
Niastella_koreensis_DSM_17620
Segetibacter_koreensis_DSM_18137
100/100
Flavisolibacter_ginsengiterrae_DSM_18136
63/-
Flavisolibacter_ginsengisoli_DSM_18119
100/96
Terrimonas_ferruginea_DSM_30193
100/100
Niabella_aurantiaca_DSM_17617
Niabella_soli_DSM_19437
100/68
Prolixibacter_bellariivorans_ATCC_BAA-1284
100/100
Draconibacterium_sediminis_JN14CK-3
Draconibacterium_orientale_FH5
100/100
Rikenella_microfusus_DSM_15922
92/87
99/100
Alistipes_inops_627
Alistipes_indistinctus_YIT_12060
100/100
Alistipes_putredinis_DSM_17216
96/70
Alistipes_onderdonkii_DSM_19147
77/88
Alistipes_finegoldii_DSM_17242
Alistipes_shahii_WAL_8301
Alkaliflexus_imshenetskii_DSM_15055
Saccharicrinis_fermentans_DSM_9555
100/100
Butyricimonas_virosa_DSM_23226
100/100
Butyricimonas_synergistica_DSM_23225
65/-
-/92
Odoribacter_splanchnicus_DSM_20712
Odoribacter_laneus_YIT_12061
Proteiniphilum_acetatigenes_DSM_18083
Tannerella_forsythia_ATCC_43037
70/-
100/100
Porphyromonas_bennonis_DSM_23058
98/100
Porphyromonas_somerae_DSM_23386
100/100
Porphyromonas_levii_DSM_23370
Porphyromonas_catoniae_ATCC_51270
-/61
100/100
Porphyromonas_gulae_DSM_15663
Porphyromonas_gingivalis_ATCC_33277
86/81
72/-
Porphyromonas_macacae_DSM_20710
100/94
Porphyromonas_endodontalis_ATCC_35406
100/100
Porphyromonas_uenonis_DSM_23387
Porphyromonas_asaccharolytica_DSM_20707
73/97
Paludibacter_propionicigenes_DSM_17365
95/94
Dysgonomonas_capnocytophagoides_DSM_22835
97/99
Dysgonomonas_mossii_DSM_22836
Dysgonomonas_gadei_ATCC_BAA-286
100/100
Coprobacter_fastidiosus_DSM_26242
97/86
Coprobacter_secundus_177
100/100
Barnesiella_intestinihominis_YIT_11860
100/96
Barnesiella_viscericola_DSM_18177
97/100
Parabacteroides_goldsteinii_WAL_12034
100/100

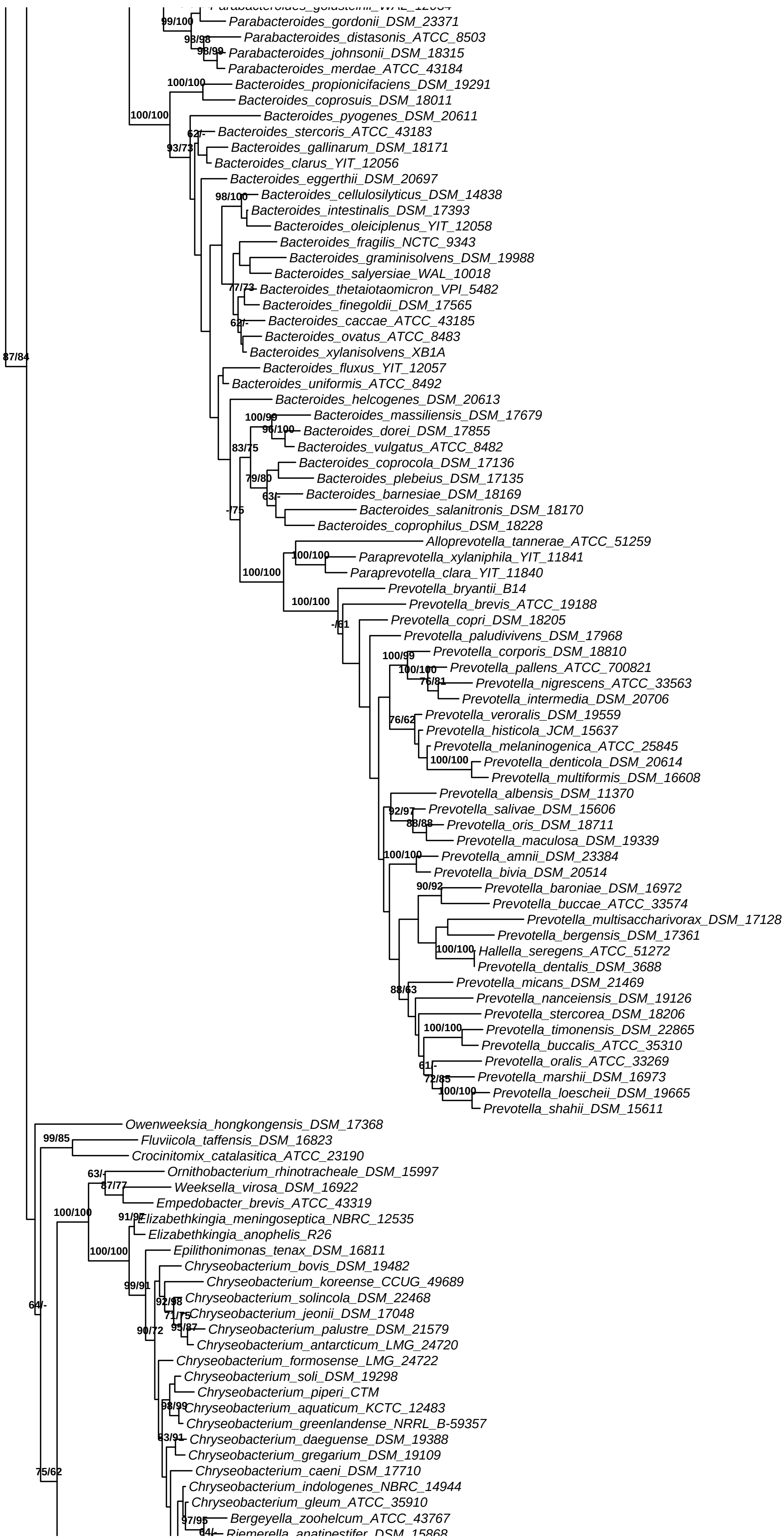

Parabacteroides_goldsteinii_WAL_12034
99/100
Parabacteroides_gordonii_DSM_23371
98/98
Parabacteroides_distasonis_ATCC_8503
98/99
Parabacteroides_johnsonii_DSM_18315
Parabacteroides_merdae_ATCC_43184
100/100
Bacteroides_propionicifaciens_DSM_19291
Bacteroides_coprosuis_DSM_18011
100/100
Bacteroides_pyogenes_DSM_20611
62/-
Bacteroides_stercoris_ATCC_43183
93/73
Bacteroides_gallinarum_DSM_18171
Bacteroides_clarus_YIT_12056
Bacteroides_eggerthii_DSM_20697
98/100
Bacteroides_cellulosilyticus_DSM_14838
Bacteroides_intestinalis_DSM_17393
Bacteroides_oleiciplenus_YIT_12058
Bacteroides_fragilis_NCTC_9343
Bacteroides_graminisolvens_DSM_19988
Bacteroides_salyersiae_WAL_10018
77/73
Bacteroides_thetaiotaomicron_VPI_5482
Bacteroides_finegoldii_DSM_17565
62/-
Bacteroides_caccae_ATCC_43185
Bacteroides_ovatus_ATCC_8483
Bacteroides_xylanisolvens_XB1A
87/84
Bacteroides_fluxus_YIT_12057
Bacteroides_uniformis_ATCC_8492
Bacteroides_helcogenes_DSM_20613
100/99
Bacteroides_massiliensis_DSM_17679
96/100
Bacteroides_dorei_DSM_17855
83/75
Bacteroides_vulgatus_ATCC_8482
Bacteroides_coprocola_DSM_17136
79/80
Bacteroides_plebeius_DSM_17135
63/-
Bacteroides_barnesiae_DSM_18169
-/75
Bacteroides_salanitronis_DSM_18170
Bacteroides_coprophilus_DSM_18228
Alloprevotella_tannerae_ATCC_51259
100/100
Paraprevotella_xylaniphila_YIT_11841
100/100
Paraprevotella_clara_YIT_11840
Prevotella_bryantii_B14
100/100
Prevotella_brevis_ATCC_19188
-/61
Prevotella_copri_DSM_18205
Prevotella_paludivivens_DSM_17968
100/99
Prevotella_corporis_DSM_18810
100/100
Prevotella_pallens_ATCC_700821
76/81
Prevotella_nigrescens_ATCC_33563
Prevotella_intermedia_DSM_20706
76/62
Prevotella_veroralis_DSM_19559
Prevotella_histicola_JCM_15637
Prevotella_melaninogenica_ATCC_25845
100/100
Prevotella_denticola_DSM_20614
Prevotella_multiformis_DSM_16608
Prevotella_albensis_DSM_11370
92/97
Prevotella_salivae_DSM_15606
88/88
Prevotella_oris_DSM_18711
Prevotella_maculosa_DSM_19339
100/100
Prevotella_amnii_DSM_23384
Prevotella_bivia_DSM_20514
90/92
Prevotella_baroniae_DSM_16972
Prevotella_buccae_ATCC_33574
Prevotella_multisaccharivorax_DSM_17128
Prevotella_bergensis_DSM_17361
100/100
Hallella_seregens_ATCC_51272
Prevotella_dentalis_DSM_3688
88/63
Prevotella_micans_DSM_21469
Prevotella_nanceiensis_DSM_19126
Prevotella_stercorea_DSM_18206
100/100
Prevotella_timonensis_DSM_22865
Prevotella_buccalis_ATCC_35310
61/-
Prevotella_oralis_ATCC_33269
72/85
Prevotella_marshii_DSM_16973
100/100
Prevotella_loescheii_DSM_19665
Prevotella_shahii_DSM_15611
Owenweeksia_hongkongensis_DSM_17368
99/85
Fluviicola_taffensis_DSM_16823
Crocinitomix_catalasitica_ATCC_23190
63/-
Ornithobacterium_rhinotracheale_DSM_15997
87/77
Weeksella_virosa_DSM_16922
100/100
Empedobacter_brevis_ATCC_43319
91/97
Elizabethkingia_meningoseptica_NBRC_12535
Elizabethkingia_anophelis_R26
100/100
Epilithonimonas_tenax_DSM_16811
Chryseobacterium_bovis_DSM_19482
99/91
Chryseobacterium_koreense_CCUG_49689
92/98
Chryseobacterium_solincola_DSM_22468
64/-
71/75
Chryseobacterium_jeonii_DSM_17048
95/87
Chryseobacterium_palustre_DSM_21579
90/72
Chryseobacterium_antarcticum_LMG_24720
Chryseobacterium_formosense_LMG_24722
Chryseobacterium_soli_DSM_19298
Chryseobacterium_piperi_CTM
98/99
Chryseobacterium_aquaticum_KCTC_12483
Chryseobacterium_greenlandense_NRRL_B-59357
63/91
Chryseobacterium_daeguense_DSM_19388
Chryseobacterium_gregarium_DSM_19109
75/62
Chryseobacterium_caeni_DSM_17710
Chryseobacterium_indologenes_NBRC_14944
Chryseobacterium_gleum_ATCC_35910
97/95
Bergeyella_zoohelcum_ATCC_43767
64/-
Riemerella_anatipestifer_DSM_15868

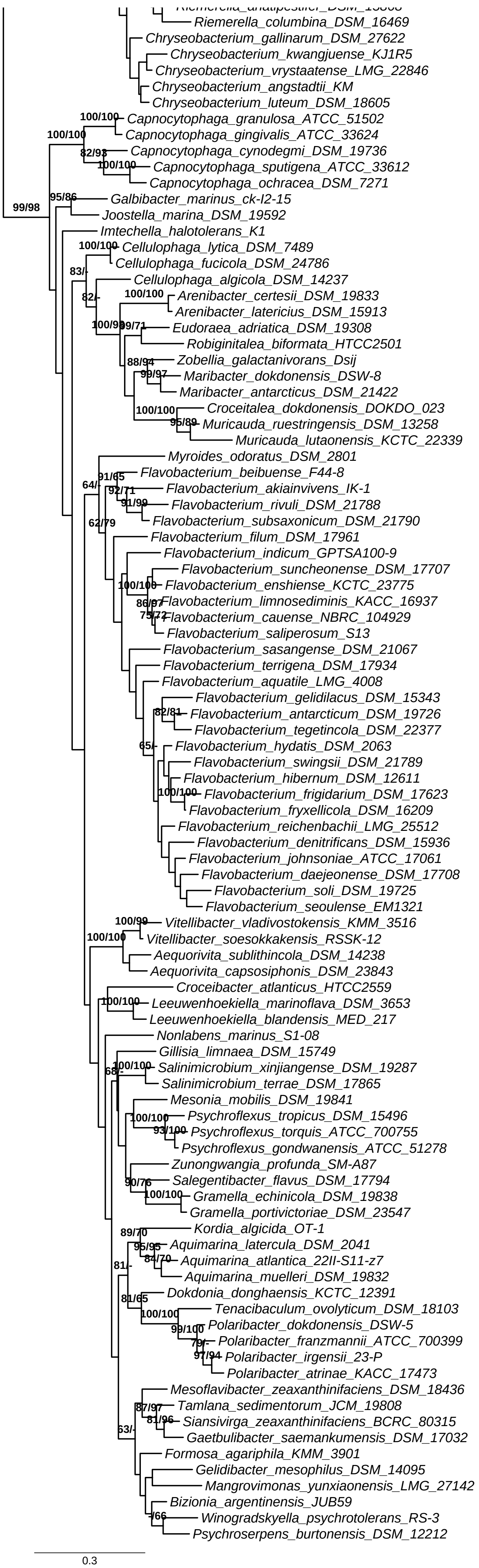
Riemerella_columbina_DSM_16469
Chryseobacterium_gallinarum_DSM_27622
Chryseobacterium_kwangjuense_KJ1R5
Chryseobacterium_vrystaatense_LMG_22846
Chryseobacterium_angstadtii_KM
Chryseobacterium_luteum_DSM_18605
Capnocytophaga_granulosa_ATCC_51502
Capnocytophaga_gingivalis_ATCC_33624
Capnocytophaga_cynodegmi_DSM_19736
Capnocytophaga_sputigena_ATCC_33612
Capnocytophaga_ochracea_DSM_7271
Galbibacter_marinus_ck-I2-15
Joostella_marina_DSM_19592
Imtechella_halotolerans_K1
Cellulophaga_lytica_DSM_7489
Cellulophaga_fucicola_DSM_24786
Cellulophaga_algicola_DSM_14237
Arenibacter_certesii_DSM_19833
Arenibacter_latericius_DSM_15913
Eudoraea_adriatica_DSM_19308
Robiginitalea_biformata_HTCC2501
Zobellia_galactanivorans_Dsij
Maribacter_dokdonensis_DSW-8
Maribacter_antarcticus_DSM_21422
Croceitalea_dokdonensis_DOKDO_023
Muricauda_ruestringensis_DSM_13258
Muricauda_lutaonensis_KCTC_22339
Myroides_odoratus_DSM_2801
Flavobacterium_beibuense_F44-8
Flavobacterium_akiainvivens_IK-1
Flavobacterium_rivuli_DSM_21788
Flavobacterium_subsaxonicum_DSM_21790
Flavobacterium_filum_DSM_17961
Flavobacterium_indicum_GPTSA100-9
Flavobacterium_suncheonense_DSM_17707
Flavobacterium_enshiense_KCTC_23775
Flavobacterium_limnosediminis_KACC_16937
Flavobacterium_cauense_NBRC_104929
Flavobacterium_saliperosum_S13
Flavobacterium_sasangense_DSM_21067
Flavobacterium_terrigena_DSM_17934
Flavobacterium_aquatile_LMG_4008
Flavobacterium_gelidilacus_DSM_15343
Flavobacterium_antarcticum_DSM_19726
Flavobacterium_tegetincola_DSM_22377
Flavobacterium_hydatis_DSM_2063
Flavobacterium_swingsii_DSM_21789
Flavobacterium_hibernum_DSM_12611
Flavobacterium_frigidarium_DSM_17623
Flavobacterium_fryxellicola_DSM_16209
Flavobacterium_reichenbachii_LMG_25512
Flavobacterium_denitrificans_DSM_15936
Flavobacterium_johnsoniae_ATCC_17061
Flavobacterium_daejeonense_DSM_17708
Flavobacterium_soli_DSM_19725
Flavobacterium_seoulense_EM1321
Vitellibacter_vladivostokensis_KMM_3516
Vitellibacter_soesokkakensis_RSSK-12
Aequorivita_sublithincola_DSM_14238
Aequorivita_capsosiphonis_DSM_23843
Croceibacter_atlanticus_HTCC2559
Leeuwenhoekiella_marinoflava_DSM_3653
Leeuwenhoekiella_blandensis_MED_217
Nonlabens_marinus_S1-08
Gillisia_limnaea_DSM_15749
Salinimicrobium_xinjiangense_DSM_19287
Salinimicrobium_terrae_DSM_17865
Mesonia_mobilis_DSM_19841
Psychroflexus_tropicus_DSM_15496
Psychroflexus_torquis_ATCC_700755
Psychroflexus_gondwanensis_ATCC_51278
Zunongwangia_profunda_SM-A87
Salegentibacter_flavus_DSM_17794
Gramella_echinicola_DSM_19838
Gramella_portivictoriae_DSM_23547
Kordia_algicida_OT-1
Aquimarina_latercula_DSM_2041
Aquimarina_atlantica_22II-S11-z7
Aquimarina_muelleri_DSM_19832
Dokdonia_donghaensis_KCTC_12391
Tenacibaculum_ovolyticum_DSM_18103
Polaribacter_dokdonensis_DSW-5
Polaribacter_franzmannii_ATCC_700399
Polaribacter_irgensii_23-P
Polaribacter_atrinae_KACC_17473
Mesoflavibacter_zeaxanthinifaciens_DSM_18436
Tamlana_sedimentorum_JCM_19808
Siansivirga_zeaxanthinifaciens_BCRC_80315
Gaetbulibacter_saemankumensis_DSM_17032
Formosa_agariphila_KMM_3901
Gelidibacter_mesophilus_DSM_14095
Mangrovimonas_yunxiaonensis_LMG_27142
Bizionia_argentinensis_JUB59
Winogradskyella_psychrotolerans_RS-3
Psychroserpens_burtonensis_DSM_12212
0.3

Figure 4 - phylogenomic GBDP tree without collapsed branches

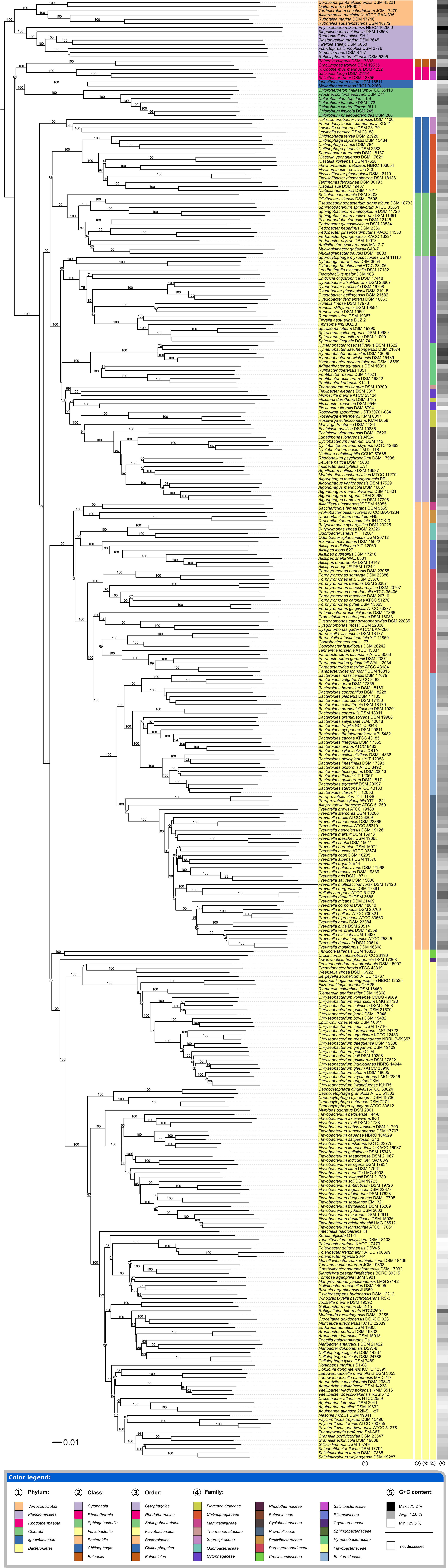
0.01
Color legend:
① Phylum:
Verrucomicrobia
Planctomycetes
Rhodothermaeota
Chlorobi
Ignavibacteriae
Bacteroidetes
② Class:
Cytophagia
Rhodothermia
Sphingobacteriia
Flavobacteriia
Bacteroidia
Chitinophagia
Balneolia
③ Order:
Cytophagales
Rhodothermales
Sphingobacteriales
Flavobacteriales
Bacteroidales
Chitinophagales
Balneolales
④ Family:
Flammeovirgaceae
Chitinophagaceae
Marinilabiliaceae
Thermonemataceae
Saprospiraceae
Odoribacteraceae
Cytophagaceae
Rhodothermaceae
Balneolaceae
Cyclobacteriaceae
Prevotellaceae
Prolixibacteraceae
Porphyromonadaceae
Crocinitomicaceae
Salinibacteraceae
Rikenellaceae
Cryomorphaceae
Sphingobacteriaceae
Hymenobacteraceae
Flavobacteriaceae
Bacteroidaceae
⑤ G+C content:
Max.: 73.2 %
Avg.: 42.6 %
Min.: 29.5 %
not discussed
